# Genome-wide equine preimplantation genetic testing enabled by simultaneous haplotyping and copy number detection

**DOI:** 10.1038/s41598-023-48103-7

**Published:** 2024-01-23

**Authors:** T. De Coster, Y. Zhao, O. Tšuiko, S. Demyda-Peyrás, A. Van Soom, J. R. Vermeesch, K. Smits

**Affiliations:** 1 Department of Internal Medicine, Reproduction and Population Medicine, Ghent University, Merelbeke, Belgium; 2 Department of Human Genetics, KU Leuven, Leuven, Belgium; 3 Department of Genetics, University of Córdoba, Córdoba, Spain; 4 Department of Animal Production, Veterinary School, National University of La Plata, La Plata, Argentina

**Keywords:** Developmental biology, Genetics

## Abstract

In different species, embryonic aneuploidies and genome-wide errors are a major cause of developmental failure. The increasing number of equine embryos being produced worldwide provides the opportunity to characterize and rank or select embryos based on their genetic profile prior to transfer. Here, we explored the possibility of generic, genome-wide preimplantation genetic testing concurrently for aneuploidies (PGT-A) and monogenic (PGT-M) traits and diseases in the horse, meanwhile assessing the incidence and spectrum of chromosomal and genome-wide errors in in vitro*-*produced equine embryos. To this end, over 70,000 single nucleotide polymorphism (SNP) positions were genotyped in 14 trophectoderm biopsies and corresponding biopsied blastocysts, and in 26 individual blastomeres from six arrested cleavage-stage embryos. Subsequently, concurrent genome-wide copy number detection and haplotyping by haplarithmisis was performed and the presence of aneuploidies and genome-wide errors and the inherited parental haplotypes for four common disease-associated genes with high carrier frequency in different horse breeds (*GBE1, PLOD1, B3GALNT2, MUTYH*), and for one color coat-associated gene (*STX17*) were compared in biopsy-blastocyst combinations. The euploid (n = 12) or fully aneuploid (n = 2) state and the inherited parental haplotypes for 42/45 loci of interest of the biopsied blastocysts were predicted by the biopsy samples in all successfully analyzed biopsy-blastocyst combinations (n = 9). Two biopsies showed a loss of maternal chromosome 28 and 31, respectively, which were confirmed in the corresponding blastocysts. In one of those biopsies, additional complex aneuploidies not present in the blastocyst were found. Five out of six arrested embryos contained chromosomal and/or genome-wide errors in most of their blastomeres, demonstrating their contribution to equine embryonic arrest in vitro. The application of the described PGT strategy would allow to select equine embryos devoid of genetic errors and pathogenetic variants, and with the variants of interest, which will improve foaling rate and horse quality. We believe this approach will be a gamechanger in horse breeding.

## Introduction

While traditional breeding in the horse was based on natural mating, modern breeding strategies are influenced by the rapid development of assisted reproduction technologies (ART). The introduction of artificial insemination at the end of the nineteenth century allowed storage and distribution of male genetics^1^. Nowadays, also valuable female individuals can produce more than one foal per year through the collection of oocytes and the production of embryos, both in vivo and in vitro. The number of equine embryos being produced is increasing annually, with 37,094 embryos produced in 2021, an increase of 10% as compared to 2020^2^. This increase is mainly caused by the rapid growth of equine in vitro embryo production (IVEP) and recent progress in embryo cryopreservation^2,3^. The popularity of IVEP results from the possibility to increase the genetic gain from high quality and/or subfertile mares and from economic benefits related to the full exploitation of expensive straws of semen from high quality stallions. Moreover, in contrast to flushed embryos, in vitro-produced embryos can be generated outside the reproductive season and are routinely cryopreserved and traded. As a consequence, modern breeding strategies gave rise to a new market in which equine embryos are sold based upon pedigree’s phenotypes or performances. On the 4th of October 2022, a record price of € 124,000 euro was paid for an equine embryo with a promising pedigree^4^. However, it remains unknown if transfer of such an embryo will result in a healthy foal with the aspired phenotypical characteristics.

Embryonic and fetal loss remains one of the greatest challenges in equine breeding. Indeed, only ~ 18 to 26% of injected oocytes reach the transferable blastocyst stage during IVEP^3,5^. Moreover, only 76 to 83% of the artificial inseminations with fresh semen^6,7^ and ~ 85% of fresh in vivo and ~ 70% of frozen-thawed day 6 to 9 in vitro-produced embryo transfers^3,8–10^ generate a clinical pregnancy. When a pregnancy is clinically detected after in vivo fertilization, 5 to 10% of day 14- and 5 to 10% of day 70-pregnancies additionally fail to produce a viable foal (reviewed elsewhere^11^). Following IVEP, a doubled incidence of early pregnancy losses^9^, of which a higher rate seems to present as anembryonic vesicles^12,13^ has been reported. Due to the seasonal breeding and the formation of endometrial cup cells, pregnancy losses in horses are complicated, as those occurring after day 35 will usually leave the mare barren for that season, which has economic repercussions.

Similar to what has been reported in humans and other species^14–19^, chromosomal gains or losses, also called aneuploidies, are a major cause of equine pregnancy loss, occurring at a rate of 22% in early lost conceptuses^20^. In humans and cattle, aneuploidy occurs frequently throughout early embryo development in vivo^21^ and in vitro^22–29^, and contributes also to pregnancy loss before the clinical detection of pregnancy. The latter is known from transfers of aneuploid bovine and human blastocysts^26,30–32^ and the analysis of in vitro-produced human embryos that arrested before reaching the blastocyst stage^33,34^. In addition, aneuploidy may present itself also on a genome-wide level, resulting in ploidy errors and IVEP may increase the incidence of genomic instability^23–25,27,28,31,32,35,36^. In horses, genomic instability throughout embryo development has only been assessed in few studies. Two studies showed the presence of chromosome-containing micronuclei in cleavage- and blastocyst- stage in vitro-produced embryos, suggesting chromosome segregation errors^37,38^. Another one described embryonic aneuploidies on chromosome two and four only, in both in vitro*-* and in vivo*-* produced morulas and blastocysts and suggested that IVEP increases the likelihood of aneuploidy, although data were not significant^39^. Adding to this suggestion, a study investigating the effect of in vitro maturation (IVM) on the rate of aneuploidy by immunostaining found that equine in vitro-matured oocytes were significantly more affected by aneuploidy than in vivo matured oocytes (45.5% vs 0%, respectively)^40^. In all equine studies thus far, genome-wide errors have not been recovered due to technical limitations and embryos were sacrificed for the analysis and thus, not transferred. By consequence, the incidence and nature of aneuploidies and genome-wide errors throughout early embryonic development, and the effect on early embryo viability remains undetermined in the horse.

Besides aneuploidies or genome-wide errors, also the inherited alleles determine the viability of the conceptus, and additionally blueprint the phenotype of the foal. Domestication and centuries of horse breeding, selecting (related) mares and stallions with the desired traits, have led to the evolution of modern horses. These traits used to include phenotypical characteristics facilitating transportation, farming, and warfare purposes, but breeding goals have shifted since the industrialization towards traits related to the exterior of the specific breed standards and sports performances. Intensive human selection during the last 250 years has resulted in homogeneity within and a substantial variation among different horse breeds^41^. This resulted in the accumulation of both alleles for the desired traits and deleterious mutations in today’s horse genome^42,43^. As a consequence, several hereditary diseases have come up alongside desirable traits characterizing these horse breeds and some of them additionally contribute to pregnancy loss or lethality at or close to birth. One of those, occurring with a carrier frequency of 7 and 8% in quarter and paint horses, respectively^44^, is the glycogen branching enzyme deficiency (GBED). This autosomal recessive disease is caused by a mutation in the glycogen branching enzyme 1 (*GBE1)* gene that results in non-functional glycogen storage^45,46^. It is responsible for 3% of spontaneous abortions, and cardiac or respiratory failure, seizures, muscle weakness and death of foals within 18 weeks of age following homozygous inheritance^47^. Another one, called warmblood fragile foal syndrome (WFFS) is caused by a mutation in the procollagen-lysine, 2-oxoglutarate 5-dioxygenase 1* (PLOD1)* gene, and is important to warmblood horses in which it occurs with a frequency of up to 17%, depending on the specific breed^48,49^. The *PLOD1* mutation causes deviant collagen formation, and homozygous inheritance presents as severe skin lesions in foals, which require euthanasia just after birth^50^. In turn, 5–9% of Arabian horses are burdened by the occurrence of cerebellar abiotrophy (CA), a neurodegenerative, autosomal recessive disease caused by a mutation of mutY DNA glycosylase* (MUTYH)* gene that results in the loss of Purkinje neurons and causes ataxia in foals^51–53^. Also in Friesian horses, 13.3–17.3% are carrying a mutation of the beta-1,3-N-acetylgalactosaminyltransferase 2 (*B3GALNT2)* gene, responsible for the autosomal recessive congenital hydrocephalus (CH), which results in stillbirth, dystocia or postnatal euthanasia^54^. In closed studbooks, carriers of known recessive diseases are excluded from breeding, giving rise to a further increase of inbreeding in these small populations.

The production of equine embryos provides the opportunity for characterization of embryos by preimplantation genetic testing (PGT), which involves the sampling of embryonic material for genetic profiling prior to embryo transfer. Application of PGT for aneuploidies (PGT-A) would allow to prioritize embryos with a normal chromosomal and genome-wide content for transfer, potentially increasing pregnancy and foaling rates. On the other hand, PGT for monogenic (PGT-M), or polygenic (PGT-P) traits and diseases and structural chromosomal errors (PGT-SR) would provide the possibility to select only healthy embryos and prioritize those with desirable traits for transfer. While testing of the mating stallion and mare for monogenic traits and diseases can currently be used for informing mating choices when breeding with animals carrying recessive alleles allowing for non-affected offspring, implementation of PGT-M would allow to include animals carrying both recessive and dominant alleles in breeding programs and to prevent the birth of both carrier and affected foals by selective transfer of embryos to recipient mares, thus avoiding inbreeding while eliminating unwanted alleles from the population. In addition, both PGT-M and PGT-P reduce the generation interval, allowing for improving selective breeding in horses, with PGT-P having the further ability to increase the accuracy of selection for polygenic traits and diseases. In general, applying PGT woud also increase our understanding of the origin of aneuploidy and genome-wide errors in horses and the contribution of aneuploidy, genome-wide errors and mutations to pregnancy loss before and after clinical pregnancy detection. However, PGT for horses remains underdeveloped as compared to humans or cattle with no publications on PGT-A, and few studies on PGT-M, limited to the interrogation of a maximum of 33 loci related to diseases or traits, including sex, ID markers, coat color, based on (multiplex) targeted PCR^3,55–58^. A generic method allowing for concurrent, genome-wide PGT-A, PGT-M and PGT-SR with the potential for PGT-P^59–61^, as successfully applied for humans^25,32,62^ and cattle^27,31^, would increase the potential of PGT for both clinical and research-based equine applications.

Here, we explored the possibility of concurrent, generic, genome-wide PGT-A and PGT-M in horses using the ‘single-cell haplotyping and imputation of linked disease variants’ (siCHILD)/haplarithmisis technique previously applied for humans and cattle^25,36,59,63^. Simultaneously, we assessed the incidence and spectrum of chromosomal and genome-wide aberrations in equine preimplantation development in vitro by analysis of arrested and blastocyst-stage embryos.

## Results

**Figure 1:**
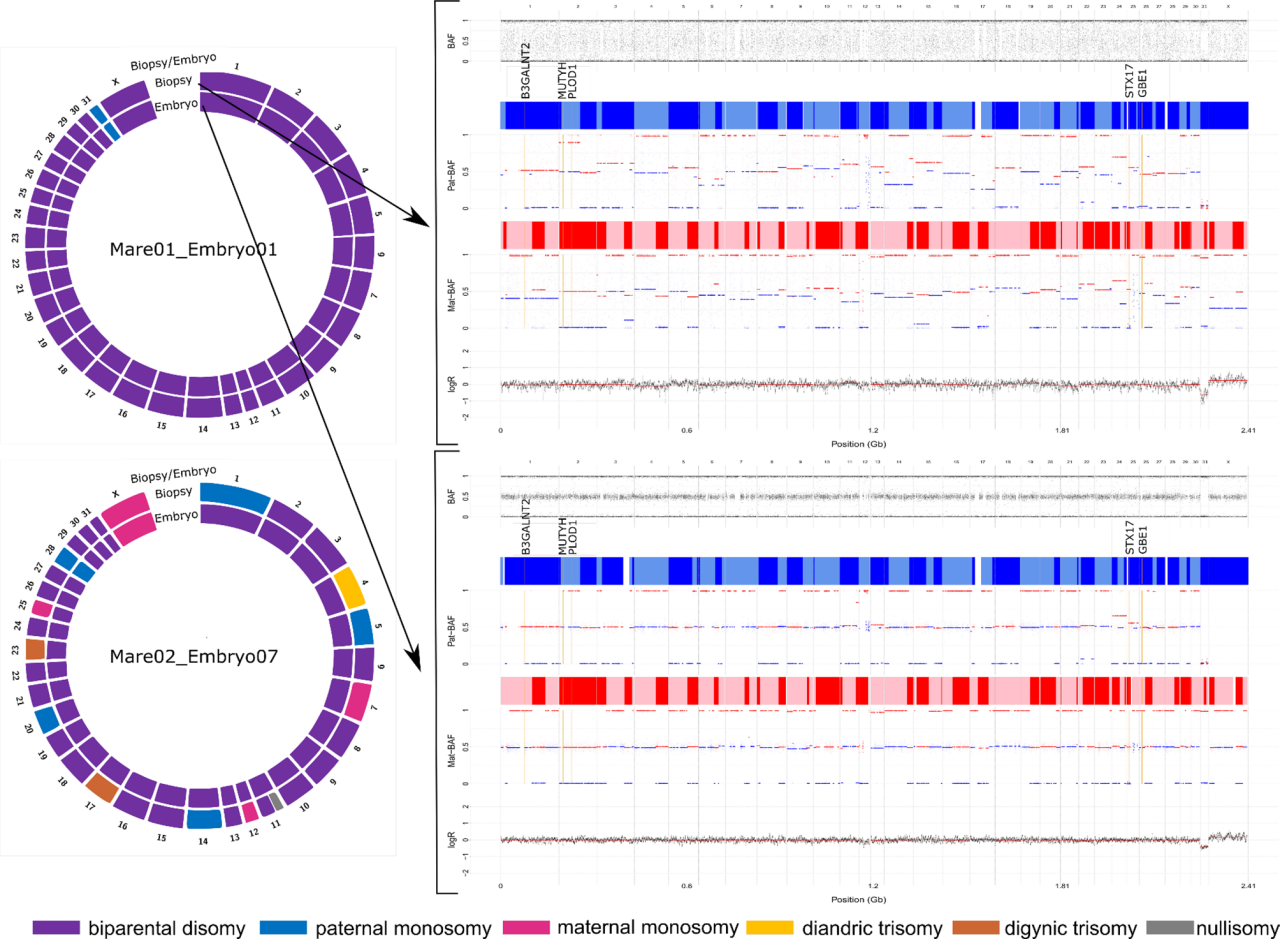
PGT analysis of blastocyst-stage equine embryos. On the left side, the circos plots are depicted for two out of 14 blastocyst-stage equine embryos that were aneuploid. The outer and inner circles represent the genome constitution per chromosome (1-X) of the biopsy sample and the corresponding biopsied blastocyst sample, respectively. Different colors represent different types of aneuploidies. On the right, the parental haplarithm plots of the biopsy (upper plot) and the corresponding embryo sample (lower plot) of Mare01_Embryo01 are depicted containing the genome-wide LogR value (measure for the chromosome copy number), the maternal B-allele-frequency (Mat-BAF), the maternally inherited haplotype blocks (pink/red colors represent regions inherited from maternal homologue 1 or 2), the paternal B-allele-frequency (Pat-BAF) and the paternally inherited haplotype blocks (light/dark blue colors represent regions inherited from maternal homologue 1 or 2) from the bottom to the top, respectively. The inherited parental haplotypes of four disease-causing genes (*B3GALNT2* for congenital hydrocephalus, *MUTYH* for cerebellar abiotrophy, *PLOD1* for warmblood fragile foal syndrome, *GBE1* for glycogen branching enzyme deficiency) and the gene causative for grey color coat (*STX17*) are indicated by the yellow line. Genome-wide haplarithm plots and detailed chromosome-wise haplarithm plots of the chromosomes carrying a gene of interest of all samples can be retrieved in Additional file 1, Figs. 1 and 2, respectively.

Following IVEP, fourteen trophectoderm (TE) biopsies and corresponding biopsied whole blastocysts and 26 individual blastomeres from six arrested cleavage-stage embryos were analyzed for their genome-wide copy number and haplotype. All sample characteristics, results of the genetic analysis, and genome-wide haplarithm plots obtained from this study are provided in Additional file 1, Table 1 and Fig. 1 (TE biopsies and corresponding biopsied blastocysts), Additional file 2, Table 1 and Fig. 1 (blastomeres from arrested embryos) and Additional file 3 (all samples). The number of informative SNPs was around 20,000 for samples for which parental genotypes were phased with sibling embryos and 7229 for samples of Mare03, for which parental genotypes were phased with paternal grandparents. Five biopsies and seven single blastomeres failed to give a result. The average coverage rate of successfully analyzed biopsied blastocysts, biopsies and single blastomeres was 70.54, 55.83 and 45.19%, respectively (Additional file 1, Table 1 and Additional file 2, Table 1). The average rate of Mendelian inconsistency of the called SNP genotypes in successfully analyzed blastocysts, biopsies and single blastomeres was 0.91, 3.14 and 18.94%, respectively (Additional file 3). Nine and five of the blastocyst-stage embryos and one and five of the arrested embryos were found to be male and female, respectively, which was confirmed by qPCR.

### Trophectoderm biopsies and corresponding biopsied whole blastocysts

#### PGT-A

As a proof of principle for PGT-A, the presence of aneuploidies and genome-wide errors was compared in biopsy-blastocyst combinations. The euploid or fully aneuploid state of the biopsied blastocysts was predicted by the analysis of the corresponding TE biopsy samples in all nine successfully analyzed biopsy-blastocyst combinations. Two out of 14 analyzed biopsied blastocysts were aneuploid. Among them, Mare01_ Embryo01 contained a maternal chromosomal loss of chromosome 31 and Mare02_Embryo07 contained a maternal chromosomal loss of chromosome 28 (Fig. 1). The presence of a maternal chromosomal loss in both the biopsies and the biopsied blastocysts indicates the presence of a chromosomal error in all cells (full aneuploidy) and by consequence, the occurrence of a chromosomal error of meiotic origin. For Mare02_Embryo07, additional aneuploidies, not detected in the blastocyst were found in the TE biopsy. These included maternal losses of chromosomes 1, 5, 14, 20 and 28 and paternal losses of chromosomes 7, 12 and 25. Additionally, paternal trisomy was noted on chromosome 4 and maternal trisomy on chromosome 17 and 23. Furthermore, a partial nullisomy was found in chromosome 11. Because of the complex aneuploidy, the collection of multiple cells and fluctuations of the LogR and parental BAF plots, deviating from values expected for full chromosome aberrations in one cell (i.e. LogR should be at − 1, 0.58 and 1 for a monosomy, trisomy or tetrasomy, respectively (LogR = Log2(rate detected/rate expected)), and parental BAF values should be at 0, and 0 and 1 for a monosomy; 0 and 0.33, and 1 and 0.67 for a trisomy; and 0 and 0.25, and 1 and 0.75 for a tetrasomy (BAF = number of B-alleles/ number of total alleles)), the described additional aberrations in the TE biopsy sample of Mare02_Embryo07 seemed to be present in a mosaic state (i.e. differently in different cells). The presence of additional aneuploidies in a mosaic state in the biopsy sample indicate the presence of distinct chromosomal errors in the blastocyst too. However, as these were not detected, they were likely present only in a small fraction of cells (low grade mosaic aneuploidy), resulting from chromosomal segregation errors during the late embryonic divisions.

For the TE biopsy of Mare2_Embryo5 no clear evidence of a full chromosomal loss exists. Yet, the LogR and the distance between the parental BAF values fluctuate between 0 and -1 and deviate from 0.5 respectively, which can be caused by the sampling of mosaic cells. However, also the plot quality could be affected by the lower coverage of SNPs (50.90%) compared to the average SNP coverage of TE biopsy samples (55.83%).

#### PGT-M

As a proof of principle for PGT-M, we compared the inherited maternal and paternal haplotypes for four common disease-associated genes with an autosomal recessive inheritance pattern and a high carrier frequency in different horse breeds as described in the introduction, and for one phenotypical trait with an autosomal dominant inheritance pattern in all successfully analyzed biopsy-blastocyst combinations. These included the *B3GALNT2* gene on chromosome 1, mutated in case of CH, the *PLOD1* and the *MUTYH* genes on chromosome 2, mutated in case of WFFS and CA, respectively, the *GBE1* gene on chromosome 26, mutated in case of GBED and the syntaxin 17(*STX17)* gene on chromosome 25, causative for grey color coat. An example for Mare01_ Embryo01 can be found in Fig. 1. Detailed haplarithm plots of the chromosomes containing the genes of interest in all analyzed samples can be found in Additional file 1, Fig. 2. The parental haplotypes were inconclusive for four out of 90 analyzed loci, all of which involved samples of Mare02_Embryo07. The first two included the paternal haplotype of *MUTYH* in both the biopsy and embryo sample due to the presence of a recombination site and absence of informative SNPs at the gene location. The other two included the maternal haplotype of *B3GALNT2* and the paternal haplotype of *STX17* in the biopsy sample due to the loss of the maternal homologue and paternal homologue, respectively. At the other loci, the parental haplotype in the biopsy sample was confirmed by the analysis of the corresponding biopsied embryo. The length (bp) and the number of informative SNPs to the closest upstream and downstream homologous recombination site and the rate of Mendelian inconsistency are summarized in Additional file 3.

### Arrested cleavage-stage embryos

**Figure 2:**
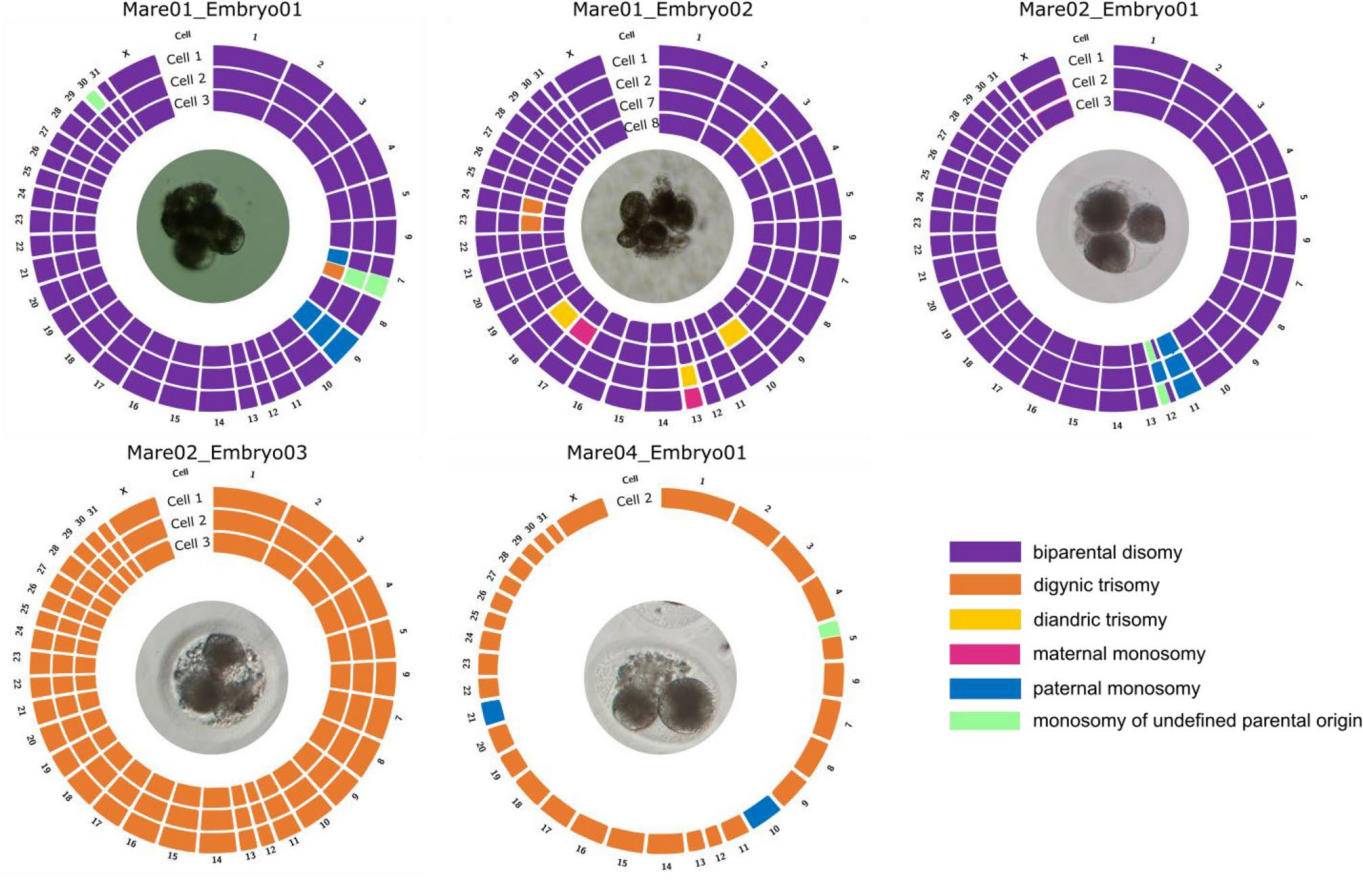
Circos plots of the five arrested equine embryos that contained chromosomal or genome-wide errors, with each circle representing the genome constitution per chromosome (1-X) of a single blastomere. Different colors represent different types of aneuploidies. Signatures of digynic trisomy across (almost all) chromosomes in Mare02_Embryo03 and Mare04_Embryo01 indicate digynic triploidy. Chromosomal errors of undefined parental origin (green) represent those that could be seen in the LogR plot, but were not confirmed by the parental haplarithm plots. Mare02_ Embryo02 was the only embryo that contained a normal, biparental diploid profile in all blastomeres and is not depicted. Lost blastomeres or blastomeres for which genetic analysis failed are not included, Also blastomere 5 of Mare01_Embryo02 was not included due to a low genome coverage and a complex aneuploid profile. Haplarithm plots of all blastomeres can be retrieved in Additional file 2, Fig. 1. In the middle of each circus plot, a picture of each embryo prior to dissociation is shown.

From six arrested embryos, only Mare02_ Embryo02 contained a normal diploid profile in all four blastomeres. All other five contained chromosomal and/or genome-wide errors in most of their blastomeres (Fig. 2). A minority of chromosomal losses were only seen in the LogR plot, but not confirmed by the parental haplarithm plots. For these errors, depicted with a green color in Fig. 2, the parental origin remained undetermined.

For Mare01_Embryo01, one blastomere was lost during the collection. The four remaining blastomeres all contained a maternal loss of chromosome nine, which thus most likely resulted from a meiotic error. In addition, a variety of other chromosomal errors was retrieved in the distinct blastomeres, pointing to errors of mitotic origin. Chromosome seven was affected differently in all blastomeres. In blastomere three, a partial chromosomal loss of the first half and a partial chromosomal gain of the second half of maternal chromosome 7 was found. Reciprocal to what was seen in blastomere three, blastomeres one and two presented with a partial, chromosomal loss of unknown parental origin of the last half of chromosome 7. Blastomere one additionally presented with a loss of chromosome 30 of unknown parental origin. For blastomere five, a lower number of SNPs were analyzed (coverage of 35.80%) and by consequence, the quality of the haplarithm plot was poorer. Nonetheless, a complex aneuploid profile was observed, with (partial) chromosomal losses and gains of both paternal and maternal origin including at least chromosomes 7, 9, 24, 27, 28 and 30.

For Mare01_ Embryo02, one blastomere was lost during collection and three other blastomeres failed analysis. From the remaining four blastomeres, blastomere eight was normal diploid and three other blastomeres were found with distinct chromosomal errors. By consequence, these are of mitotic origin. Blastomere one and two contained a paternal chromosomal loss and the reciprocal gain on chromosome 13, respectively. Blastomere seven showed a complex aneuploid profile with paternal chromosomal gains on chromosome 3, 10 and 18, a paternal chromosomal loss on chromosome 17 and maternal chromosomal gains on chromosomes 23 and 24.

In Mare02_ Embryo01, all three blastomeres presented with a maternal loss of chromosome 11, pointing to a meiotic origin. Maternal chromosome 12 was lost in blastomere two and lost partially in blastomeres one and three. For the last two blastomeres, a loss of the second half of chromosome 12 of unknown parental origin was found.

In Mare02_ Embryo03, three blastomeres failed analysis and three blastomeres showed an identical triploid profile with an additional maternal genome copy. Genome-wide regions of heterodisomy point out the presence of two distinct maternal genomes which must have resulted from a genome-wide meiotic error.

Finally, Mare04_ Embryo01, contained one blastomere which failed analysis and one blastomere with a digynic triploid profile with only one maternal genotype, pointing to a genome-wide mitotic error. Additionally, two out of three maternal copies were lost on chromosomes 10 and 21 and a partial chromosomal loss of chromosome five of unknown parental origin occurred.

## Discussion

Here, we provide proof of concept for concurrent genome-wide copy number profiling and haplotyping in the horse enabling combined PGT-A and PGT-M in a generic way. Like in humans and cattle, a similar analysis could additionally enable PGT-SR and PGT-P in the horse. Research-based application of this strategy has the potential to determine the contribution of genetic aberrations to embryonic or fetal arrest before or after the clinical detection of pregnancy. Clinically, this method allows the selection of embryos devoid of both aneuploidies and pathogenic variants, and with the genetic variants of interest. We envision this application will revolutionize horse breeding.

The simultaneous detection of the genome-wide copy number and the inherited parental haplotypes allows to determine the presence, the type (gain/loss) and the origin (maternal/paternal and meiotic/mitotic) of (segmental) aneuploidies and genome-wide errors, also called ‘ploidy’ aberrations^25,36,59^. Ploidy aberrations include triploidy and haploidy, in which case a whole parental genome of maternal or paternal origin is additionally present (digynic and diandric triploidy, respectively) or missing (androgenetic or gynogenetic haploidy, respectively), or androgenetic/gynogenetic genome-wide uniparental disomy when two genomes derived from the same parent are present. This method enabled to chart for the first time the full extent and spectrum of chromosomal and genome-wide aberrations in arrested cleavage-stage and transferable blastocyst-stage in vitro-produced equine embryos. As such, we could provide the first solid evidence of the occurrence of genome-wide errors in equine embryos. These included two digynic triploidies, one of meiotic and one of mitotic origin. Considering the almost exclusive application of intracytoplasmic sperm injection (ICSI) for in vitro fertilization (IVF) in horses, the occurrence of predominantly maternal genome-wide errors can be expected as diandry via dispermic fertilization is prevented. Furthermore, a staggering five out of six (83%) arrested cleavage-stage embryos were found presenting with chromosomal and genome-wide errors. Considering this is a bit lower compared to the frequencies of human arrested cleavage-stage embryos (93–94% in Ref.^33,34^), this could represent a realistic estimate. At the blastocyst-stage, errors were detected in two out of 14 samples (14%). This is lower as compared to the incidence reported in biopsies from blastocyst-stage embryos in humans (25–69% in Ref.^25,29,30,33^) and cattle (14–31% in Ref.^27,31^). A higher incidence was also reported in the one study on in vitro-produced equine samples based on cytogenetic evaluation (40% in blastocysts^39^). This discrepancy might be related to either sample size or species-specific differences in gene content, effect of aneuploidies on embryonic viability and/or IVEP conditions. The high incidence of chromosomal and genome-wide errors in arrested equine cleavage-stage embryos as compared to transferable blastocyst-stage embryos supports the view that these errors are a cause of in vitro embryo arrest in mammals.

With the exception of one, all arrested cleavage-stage embryos were characterized by (complex) aneuploidy and/or digynic triploidy of mitotic origin affecting all or most of their blastomeres, sometimes in combination with meiotic aneuploidy of one of the larger chromosomes or, meiotic triploidy, affecting all cells per definition. In contrast, the two aneuploid blastocysts contained errors of meiotic origin on smaller chromosomes, and one of them additionally contained mitotic aneuploidies in a small proportion of the embryo, as they were detected in the TE biopsy only. These findings corroborate the human observation that especially genomic aberrations that affect a large part of the genome in a large proportion of the embryo, such as (complex) aneuploidy resulting from mitotic errors at the earliest embryonic divisions, combinations of meiotic and mitotic aneuploidies, or ploidy aberrations seem detrimental to early embryonic development while meiotic errors or low-grade mosaicism do not necessarily prohibit embryo development to the blastocyst stage^25,29,33,34,64,65^. In horses, meiotic monosomies of smaller autosomal chromosomes have been reported in aborted equine specimens (e.g. chromosome 26, 27 and 31), but never in live born^11,20^. Here, we demonstrate the presence of meiotic monosomies of larger chromosomes (e.g. chromosome 9 in Mare01_Embryo01 and chromosome 11 in Mare02_Embryo03) in arrested embryos but not in blastocysts. Together, these data point out that monosomies of the larger chromosomes affecting all embryonic cells cause gestational loss before clinical pregnancy detection^11^, which can be due to gene-dosage imbalances resulting in embryo arrest before embryonic genome activation. In general, it should be taken into consideration that only a limited number of embryos from a limited number of mare-stallion combinations were analyzed here and therefore, analysis of more samples will be required to draw valid and generalizable conclusions.

When successful, the analysis of the TE biopsy could predict the euploid or full aneuploid state of the embryo, highlighting its potential for PGT-A. Monosomies on small chromosomes, here detected in two embryos on chromosome 28 and 31, are associated with fetal demise by 65 days of gestation^11^. Therefore, clinical application of PGT-A and selection against such embryos could increase success rates per embryo transfer as well as the live-birth rates, as was shown for in vitro-produced bovine embryos^31^. Yet, such application remains controversial. In humans for example, over 70 retrospective single and multi-center studies attest to the efficacy of PGT-A^66^, but a lack of evidence for its beneficial effect in randomized-controlled multicenter studies trials remains, except in older age groups^67^. The risk of PGT-A being performed on a few cells obtained by a biopsy procedure, is that the analysis result not necessarily reflects the status of the whole embryo in case of mosaic aneuploidy, the latter of which is not necessarily incompatible with healthy live birth^68–70^. In the study presented here, additional aneuploidies detected in the TE biopsy of one embryo, but not in the corresponding biopsied blastocyst, demonstrated the presence of low grade mosaicism. Although mosaicism was indicated by the genetic profile of the TE biopsy of that embryo and also of one other embryo, the presence and extent cannot be accurately predicted by a TE biopsy alone. By consequence, whilst fully aneuploid embryos are unviable or severely diseased, the application of PGT-A may lead to misdiagnosis of mosaic embryos^71^ and additionally, to damage to the embryo due to the biopsy procedure, leading to wastage of viable embryos. In regard of the biopsy procedure, both the needle aspiration biopsy of in vitro- and in vivo-generated embryos and the collection of herniating cells from in vitro-generated embryos have shown similar early pregnancy results as compared to non-biopsied controls^3,72^.

In contrast to cattle but similar to humans, horse embryos have a high individual value, from both an emotional and economic perspective. For that reason, the clinical application of PGT-A in horses is expected to result rather in the selection against embryos with a meiotic trisomy (which are unviable and can be discriminated by haplarithmisis) and the further ranking of euploid embryos and embryos presenting with other chromosomal errors, genome-wide errors or traces of mosaicism based on the type and number of chromosomes affected than in the selection against all abnormal embryos, as performed in cows^31^. Such ranking could impact both pregnancy rates and embryo prizes. The clinical application of PGT-A may particularly benefit the subpopulation of slowly developing in vitro-produced equine embryos. These are currently discarded by some centers since transfer results in decreased pregnancy and foaling rates^3,38,73–75^. Yet, since female embryos develop slower^73^, such practice may also underly an increased disposal of desired female embryos resulting in the observed skewed sex ratio following transfer of equine in vitro-produced embryos^8,73^. PGT-A by haplarithmisis has the potential to select against fully aneuploid embryos while salvaging the euploid and potential or presumed mosaic embryos, which might increase embryo viability and (partly) reinstate a more balanced sex ratio within this cohort. A subset of TE biopsies analyzed here, failed to give a result following haplarithmisis, which may be due to failed tubing or low fidelity of the whole genome amplification (WGA) process due to inhibition^72^. The same samples also failed analysis when analyzed by qPCR to verify the sex of the embryo, which may thus provide a method for quality assessment of the TE biopsy. The chance of a successful analysis could be increased by dividing the initial TE biopsy of 10 to 20 cells into two samples, generating a spare sample, and the upfront selection of the best sample for analysis by haplarithmisis.

We also demonstrate the ability to detect the inherited parental haplotypes at the location of five genes related to traits and diseases based on a biopsy sample, enabling PGT-M when real-case pedigree information regarding the inheritance of the allele of interest is available. Haplotyping-based PGT-M has a number of advantages as compared to other types of PGT-M. First, it minimizes misdiagnosis due to upfront WGA, which is a necessary evil to obtain sufficient input DNA when genotyping multiple targets or performing multiple analysis on one or a few cells, but results in erroneous and biased amplification. PCR-based PGT-M methods rely on the direct genotyping of the allele of interest and therefore, they are at risk to pick up the bias created by the WGA process. Especially the misdiagnosis of heterozygous embryos in horses^55–57^ and humans^76,77^ occurs frequently in PCR-based PGT-M, because of allele drop-out, resulting from the failure WGA process to amplify one of both alleles, to which especially at GC-rich locations, like the *GBE1* locus, are prone. Haplotyping-based PGT-M counters this problem, because the presence of the alleles of interest is not determined directly but instead, inferred from parental haplotypes determined from polymorphic markers flanking the loci of interest, most of which will not undergo allele drop-out. Second, the analysis is generic and performed on a genome-wide scale, making the simultaneous screening for many Mendelian or polygenic disorders and traits possible. This is in contrast to PCR-based methods, which can only detect a limited number of alleles of interest and have to be designed per family and/or location or of interest. Whilst this study looked for the inherited parental haplotypes limited to five loci related to color coat and diseases, haplotype-based PGT-M can in the short term be applied for each pedigree to determine the presence of all alleles for which the causative mutation is currently known. In horses, Mendelian inheritance has been detected for 62 equine traits and diseases, but only for 46, the (likely) causal variants is known (https://www.omia.org/home/). Further research will continue to identify the underlying genetic mutation of other Mendelian disorders and traits.

Concurrent genome-wide copy number detection and haplotyping of embryos requires pedigree genotypes to enable phasing of the genotypes into haplotypes. These include genotypes from both parents, together with a sibling (affected or unaffected for the monogenic disease or trait) or at least one of the grandparents (from the parental side(s) at risk of transmitting the allele of interest in case of PGT-M). For PGT-M, pedigree information regarding the inheritance pattern of the allele of interest is additionally required. These requirements bring along opportunities and hurdles. On the one hand, clinical application of haplotype-based PGT would enable the development of large genomic databases. On a longer term, these databases may lead to the annotation of genes for multifactorial/polygenic traits or disorders and additionally enable future ranking of embryos for traits based on polygenic risk analysis. While the initiation of human embryo selection based on PGT-P poses several ethical and technical problems^78–82^, the development of PGT-P in horses can be valuable for assessing important equine polygenic disorders like osteochondrosis dissecans^83–85^ or insect bite hypersensitivity^86^ and traits like performance^87,88^, parasite susceptibility^89^ or fertility^90,91^, for which genome-wide association studies in horses are increasing the knowledge on the heritability and associated SNPs. On the other hand, obtaining materials from family members is straightforward in humans but poses a hurdle for the application of haplotyping-based PGT in the horse industry. While DNA from the mare can be obtained via a blood sample collected during ovum-pick up or embryo transfer, DNA collection from the stallion is less straightforward as the sale of stallions’ semen is exploited by external stud farms which send expensive small doses of semen for which DNA extraction is inherently more challenging as compared to blood. As such, the enrollment of equine PGT by the method presented here in the current setting would require the collaboration of stud farms. The necessity of grandparental or sibling genotype information from live horses can be avoided by genotyping an arrested or blastocyst-stage sibling embryo from the same mare-stallion combination, as we demonstrated here too. On the longer term, the development of a large equine genotype database would enable to reuse genotype information obtained from previous PGT sessions, especially because of the popular use of certain stallions in the equine breeding programs. Such database would also allow the increased use of parental siblings for phasing of the parental haplotypes^92^.

The resolution of haplotyping via haplarithmisis depends on the number and distribution of total analysed and fraction of the informative SNPs. Here, a medium density, 70 K SNP array was applied, analyzing only a limited number of SNPs. As a consequence, some of the chromosomal copy number errors shown by the LogR plot were not confirmed by the parental haplarithm plots, resulting in the failure to determine the parental origin. Furthermore, the paternal haplotype at one locus of interest in one biopsy-blastocyst combination could not be conclusively determined due to the co-occurrence of a recombination site and lack of informative SNPs. In human haplarithmisis-based PGT, parameters based on call rate and Mendelian inconsistency are applied for general quality assessment of the samples and for PGT-M specifically, the distance to the flanking homologous recombination site and number and accuracy of informative SNPs within this area are applied for conclusive determination of the parental haplotypes at loci of interest^93^. The determination of the resolution and threshold values for equine haplotype calling requires further research, including genomic samples with known haplotypes. Applying a higher density SNP array would result in more data points for subsequent genome-wide haplotyping and thus higher resolution PGT-A and PGT-M. However, for horses, only a 671 K SNP array for concurrent analysis of 96 samples is commercially available (Axiom MNEC670; Thermo Fisher Scientific, Waltham, MA, USA^94^), which is not cost-efficient for application of PGT in horses. Alternatively, the rapid development and increased cost-efficiency of sequencing-based technologies may provide a solution to increase the number of data points. Genotyping-by-sequencing, for example, scalable in terms of both data points and sample throughput, can provide a cost-efficient alternative for arrays to provide input genotypes with an increased resolution for haplarithmisis, as demonstrated for human and bovine samples^63^.

The number of equine embryos being produced and traded worldwide is expected to grow further, especially since the recent development of repeatable regular IVF in horses^95^. Similar to cattle and humans, we postulate that the selection and ranking of these embryos by a reliable method for PGT as demonstrated here, will enhance and predict embryo transfer success rates, and will enable selection for traits and against diseases, accelerating breeding programs. In cattle, the use of SNP-arrays for genomic selection of liveborn and embryos via a biopsy has revolutionized breeding. The integration of genomic selection has doubled the rate of genetic progress for important economic traits and increased selection accuracy, while decreasing the generation interval and avoiding undesirable recessive conditions^96,97^. While we generated new insights in the incidence and nature of chromosomal and genome-wide errors in equine in vitro*-*produced embryos until the blastocyst stage and their contribution to developmental arrest, further clinical application of PGT will determine the contribution of chromosomal errors, genome-wide errors and mutations to pregnancy failure before or after clinical pregnancy detection following transfer of those blastocysts. Furthermore, since horses offer the opportunity to study embryos from a population not necessarily biased by high reproductive age or infertility like humans seeking fertility treatment, and because in vivo-generated blastocysts are frequently flushed from the uterus and transferred to a recipient mare, they represent an excellent model to determine the contribution of chromosomal an genome-wide errors to pregnancy loss before or after the clinical detection of pregnancy, and to study the in vivo and the in vitro situation (both regular IVF and ICSI) and assess and optimize in vitro culture conditions with regard to genome instability.

## Material and methods

### Equine in vitro embryo production

Embryos were produced in vitro as described previously^98,99^, separating oocytes and embryos per donor. Briefly, oocytes retrieved from slaughterhouse-derived ovaries from four mares of unknown identity were kept for ~ 15 to 18 h in holding medium (Emcare, Manhattan, KS, USA) at room temperature (22 °C) prior to IVM. Maturation of cumulus-oocyte-complexes was performed in a humidified atmosphere per donor for 31–33 h at 38.5˚C in 5% CO_2_ in 500 µL of maturation medium consisting of tissue culture medium (TCM)-199 with Earle’s salts containing 10% fetal bovine serum (FBS) (31150-022 and 10082147; Thermo Fisher Scientific, Waltham, MA, USA), 9.4 μg/mL follicle-stimulating hormone, 1.88 μg/mL luteinizing hormone (BE-V157997; Stimufol; Reprobiol, Ouffet, Belgium) and 50 μg/mL gentamycin (15710049; Thermo Fisher Scientific, Waltham, MA, USA). A tissue sample was collected for each donor animal from the ovary and stored at − 80 °C. Following maturation, oocytes were denuded by successive pipetting in 50 µL droplets of 0.1% bovine hyaluronidase (H3506-1G; Sigma-Aldrich, Saint Louis, MO, USA) in TCM-199 with Hank’s salts (22350–029; Thermo Fisher Scientific, Waltham, MA, USA) with 10% FBS and 50 μg/mL gentamycin (15710049; Thermo Fisher Scientific, Waltham, MA, USA) and TCM-199 with Hank’s salts with 10% FBS using a STRIPPER pipettor and 170 μm and 135 µm capillaries (MXL3-STR-CGR, MXL3-175 and MXL3-135; Cooper Surgical, Trumbull, CT, USA). Frozen-thawed semen from one stallion was used for fertilization and prepared as described previously^98^. Mature oocytes were fertilized by ICSI as described previously^99^ and presumptive zygotes were cultured at 38.2 °C in a humidified atmosphere of 5% O_2_, 5% CO_2_, and 90% N_2_ in 20 µL droplets of Dulbecco’s Modified Eagle Medium Nutrient Mixture F-12 (DMEM/F-12; 21331-020; Thermo Fisher Scientific, Waltham, MA, USA) with 10% FBS and 50 μg/mL gentamycin under oil (ART-4008-5PA; Cooper Surgical, Trumbull, CT, USA). Blastocyst development was monitored daily from day six onwards until day 12.

### Embryo biopsy and single-cell collection

All but two embryos reaching the blastocyst stage were biopsied at the day of TE delineation and the remaining biopsied embryos were collected immediately afterwards. Embryos not collected included two embryos of Mare 4, one of which presented as an apparent twin embryo and the other of which developed at day 13. Collection of the TE biopsies was performed as described in detail by De Coster et al.^72^. Briefly, embryos were placed in 5 µL droplets of TCM-199 with Hank’s salts containing 10% FBS under oil and immobilized by suction of a holding pipette (MPH-MED-35; Cooper Surgical, Trumbull, CT, USA) on a heated stage (39 °C) of an inverted microscope (Olympus IX73; Olympus Shinjuku, Tokyo, Japan) equipped with a Research Instruments micromanipulation system (Cooper Surgical, Trumbull, CT, USA). To avoid the collection of presumably degenerated cells, embryos were positioned such way that visual cell debris was not aspirated. Ten to 20 cells were aspirated from the TE layer following piercing of the embryo with a beveled biopsy micropipette (MPB-BS-30; Cooper Surgical, Trumbull, CT, USA) and placed in a 2.5 µL droplet of Ca^2+^/Mg^2+^-free phosphate buffered saline (PBS; 14190-144; Thermo Fisher Scientific, Waltham, MA, USA) with 1% polyvinylpyrrolidone (PVP; ART-4005; Cooper Surgical, Trumbull, CT, USA), of which 2 µL containing the biopsy was subsequently transferred to a DNase-free 0.2 mL tube on ice using a 135 µm capillary (MXL3-STR-CGR; MXL3-135; Cooper Surgical, Trumbull, CT, USA). The remaining biopsied embryos were individually removed from their zona pellucida, by incubation in warm 0.1% pronase (P8811; Merck KGaA, Darmstadt, Germany; with Hank’s salts) dissolved in TCM-199 with Hank’s salts, and subsequent washing in TCM-199 with Hank’s salts containing 10% FBS and Ca^2+^/Mg^2+^-PBS with 1% PVP, while removing the zona by pipetting with a 200 µm capillary. Zona-free embryos were subsequently transferred individually to a DNase-free 0.2 mL tube on ice containing 2 µL Ca^2+^/Mg^2+^-free PBS% with 1%PVP using a new 200 µm capillary. For embryos that arrested at the cleavage-stage, individual blastomeres were isolated and collected at day 11 and 12 of embryo culture as described elsewhere^23,36^. When individual blastomeres could not be discriminated due to degeneration and/or fragmentation, embryos were not analyzed. All samples were immediately stored at − 80 °C. To avoid cross-contamination, every embryo was washed in TCM-199 with Hank’s salts with 10% FBS before sample collection and manipulated with a new set of micropipettes and/or capillaries.

### Genetic analysis

#### Whole-genome amplification and DNA extraction

DNA of single blastomeres from arrested embryos, TE biopsies and their corresponding biopsied embryos was extracted and amplified by multiple displacement WGA using the REPLI-g Single Cell Kit (150345; Qiagen, Hilden, Germany) according to the manufacturer’s instructions with minor modifications. Full (TE biopsies and biopsied whole blastocysts) or half (single blastomeres from arrested embryos) reaction volumes were applied and the incubation reaction was reduced to 3 h. The concentration of WGA DNA was determined by Qubit Broad Range Assay (Q33266; Thermo Fisher Scientific, Waltham, MA, USA), according to the manufacturer’s protocol. Ovarian tissue from the donor mares (i.e. mothers of the respective embryos), semen from the stallion (i.e. fathers of the respective embryos) and blood from the parents of the stallion (i.e. paternal grandparents of the respective embryos) were used to extract bulk DNA (DNeasy Blood and Tissue kit; 69504; Qiagen, Hilden, Germany; User-developed protocol for semen extraction). Purity and concentration of the bulk DNA was measured with Nanodrop (Isogen, Utrecht, The Netherlands), according to the manufacturer’s protocol.

#### SNP genotyping and haplarithmisis

Over 70,000 single nucleotide polymorphisms (SNPs) were genotyped in all samples on GeneSeek Genomic Profiler (GGP) equine SNP arrays (outsourced to Neogen, Ayrshire, UK). Subsequently, discrete genotypes, B-allele frequency (BAF) values, and LogR values were exported using the GenomeStudio software Genotyping Module v. 2.0.5 (Illumina, San Diego, USA). SNP genotypes were called by setting the GenCall score at 0.75. Next the computational workflow siCHILD, which includes haplarithmisis^59^, modified for analysis of equine samples according to equine reference genome EquCab3.0 was applied. Briefly, haplarithmisis uses the phased parental genotypes and SNP BAF-values of the embryo sample to determine genome-wide haplotypes, copy-number of the haplotypes, as well as the parental and segregational origin of anomalies. Parental genotypes were phased with the SNP genotype calls derived from the paternal grandparents, when no sibling blastocyst was available (Mare03_Embryo01) or from a sibling biopsied whole blastocyst (other embryos). Next, for specific combinations of phased parental genotypes, corresponding SNP BAF values of the embryo sample were retrieved. Consequently, these values were plotted on paternal and maternal haplarithms. Visualization of genome-wide raw BAF values, paternal and maternal haplarithms, and LogR values using siCHILD rendered genome-wide haplarithm plots. A visual overview on the interpretation of the haplarithm plots on chromosomal and genome-wide level can be consulted in Refs.^59^ and^36^, respectively. Failed samples were those samples resulting in inconclusive haplarithm plots. The sex of the embryo was determined by the copy number of the X-chromosome as determined by the combined interpretation of the parental haplarithm plots and the LogR value, and verified by qPCR, using 1 µL of 1 ng/µL diluted WGA material as a template^72^.

#### PGT-M

The coordinates of the five selected genes on the equine reference genome EquCab3.0 were extracted from Online Mendelian Inheritance in Animals (OMIA) (https://omia.org/). The gene regions were then indicated by orange lines on whole genome haplarithm plots. Maternally inherited haplotype blocks (regions inherited from homologue 1 or 2 of the mother) were visualized through pink/red colors and paternally inherited haplotype blocks (regions inherited from homologue 1 or 2 of the father) were visualized with light/dark blue colors. Haplotype inheritance patterns for the five gene regions were inferred according to the color of haplotype block. The haplotype block at loci of interest were called inconclusive in the absence of heterozygosity (e.g. maternal or paternal monosomy), or informative SNP positions or, when co-localized with a meiotic recombination site.

#### Data visualization

Circos plots were drawn using the Circos software^100^.

### Ethical approval

Ethical approval was waived for this study since slaughterhouse-obtained material and embryonic samples are no subject of ethical approval.

### Supplementary Information

Supplementary Information 1.

Supplementary Information 2.

Supplementary Information 3.

## Data Availability

All SNP-array data will be available on EGA https://ega-archive.org/.
