# Supplementary material for: Genome-wide equine preimplantation genetic testing enabled by simultaneous haplotyping and copy number detection
Source: Sci Rep. 2024 Jan 23;14:2003. doi: 10.1038/s41598-023-48103-7 (PMC10805710; doi:10.1038/s41598-023-48103-7)

ADDITIONAL FILE 1: DATA OVERVIEW OF THE TROPHECTODERM BIOPSIES AND CORRESPONDING BIOPSIED BLASTOCYSTS

Table 1. Characteristics of the blastocyst samples, concentration after WGA and coverage rate.

Figure 1. Pictures and genome-wide haplarithm plots of the trophectoderm biopsies and corresponding biopsied blastocysts.

Figure 2. Haplarithm plots of chromosomes containing the five genes of interest of successfully analyzed trophectoderm biopsies and corresponding biopsied blastocyst combinations.

Table 1. Characteristics of the blastocyst samples, concentration after WGA and coverage rate.

Sample	Time of collection (day post ICSI)	Concentration after WGA (ng/µL)		Coverage of analyzed SNP genotypes (%)		Sex
		Trophectoderm biopsy	Embryo	Trophectoderm biopsy	Embryo	
Mare01_Embryo01	8	644	772	55.38*	70.72*	♀
Mare01_Embryo02	9	622	640	53.34*	70.85*	♀
Mare01_Embryo03	9	770	580	58.22*	70.86*	♀
Mare01_Embryo04	9	590	674	58.84*	70.95*	♂
Mare02_Embryo01	8	586	742	26.40	70.99*	♀
Mare02_Embryo02	8	904	728	57.52*	70.96*	♂
Mare02_Embryo03	8	870	728	57.35*	70.61*	♂
Mare02_Embryo04	9	520	668	47.88	70.67*	♂
Mare02_Embryo05	9	882	632	50.90*	70.90*	♂
Mare02_Embryo06	9	356	678	47.61	67.01*	♂
Mare02_Embryo07	9	852	712	54.10*	70.63*	♂
Mare03_Embryo01	12	702	346	56.81*	70.62*	♀
Mare04_Embryo01	9	502	780	14.56	70.80*	♂
Mare04_Embryo02	9	436	1100	50.10	70.99*	♂
**Average coverage (%)**				49.22	70.54	
**Average coverage successful (*) samples (%)**				55.83	70.54	

*Samples successfully analyzed by haplarithmisis. ICSI: intracytoplasmic sperm injection; SNP: single nucleotide polymorphism; WGA: whole genome amplification.

Figure 1. Pictures and genome-wide haplarithm plots of the trophectoderm biopsies and corresponding biopsied blastocysts.

Mare01_Embryo01_Biopsy

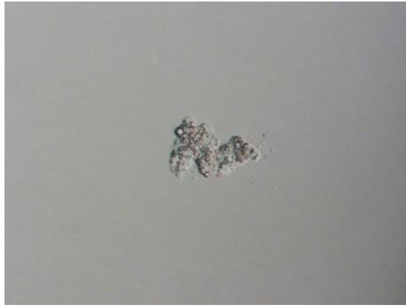

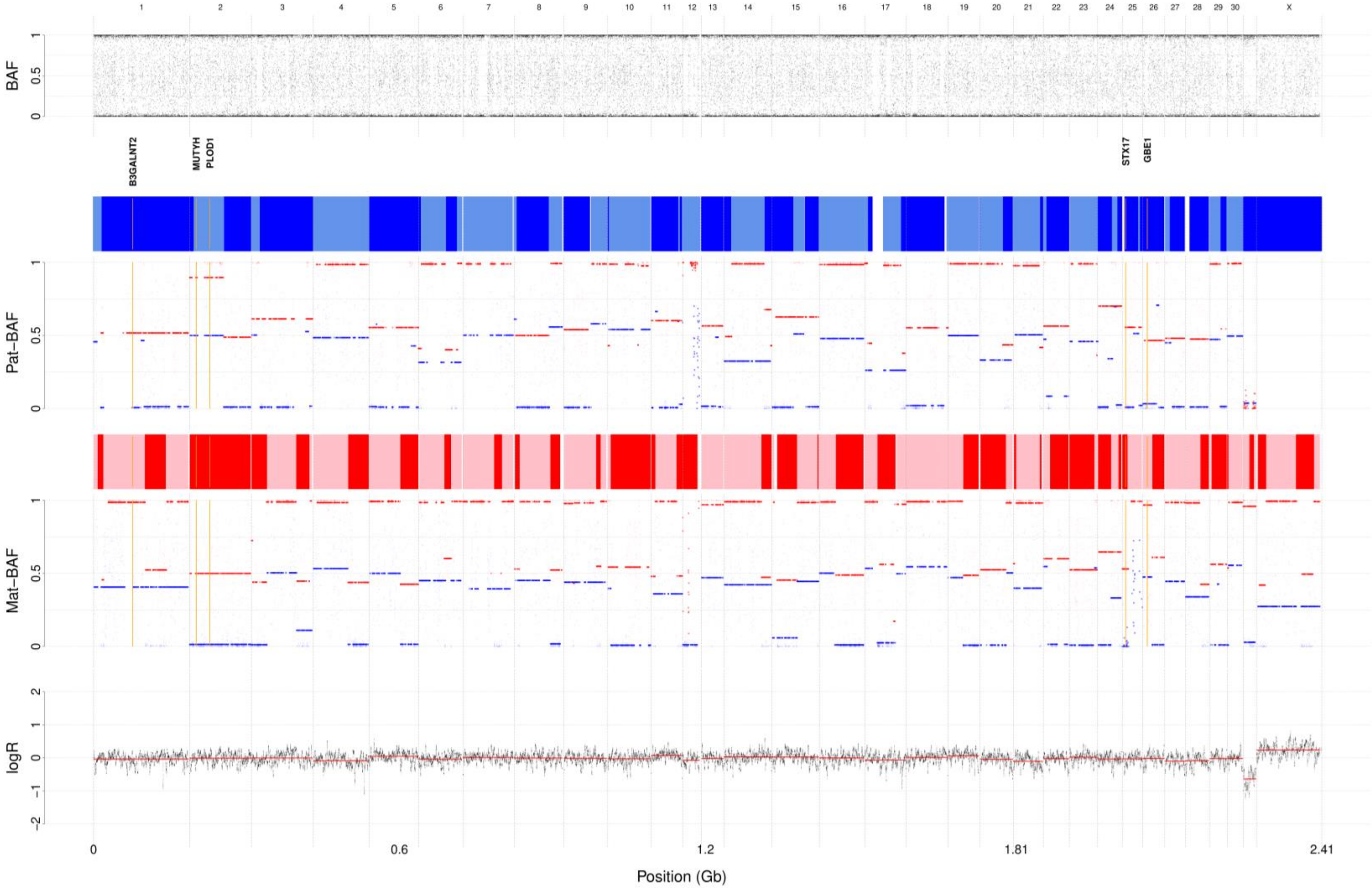

Mare01_Embryo01_Embryo

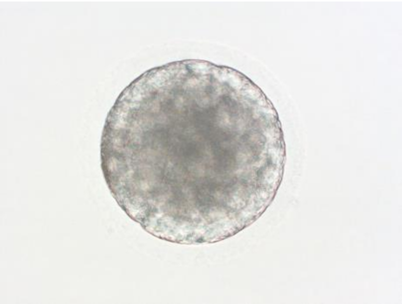

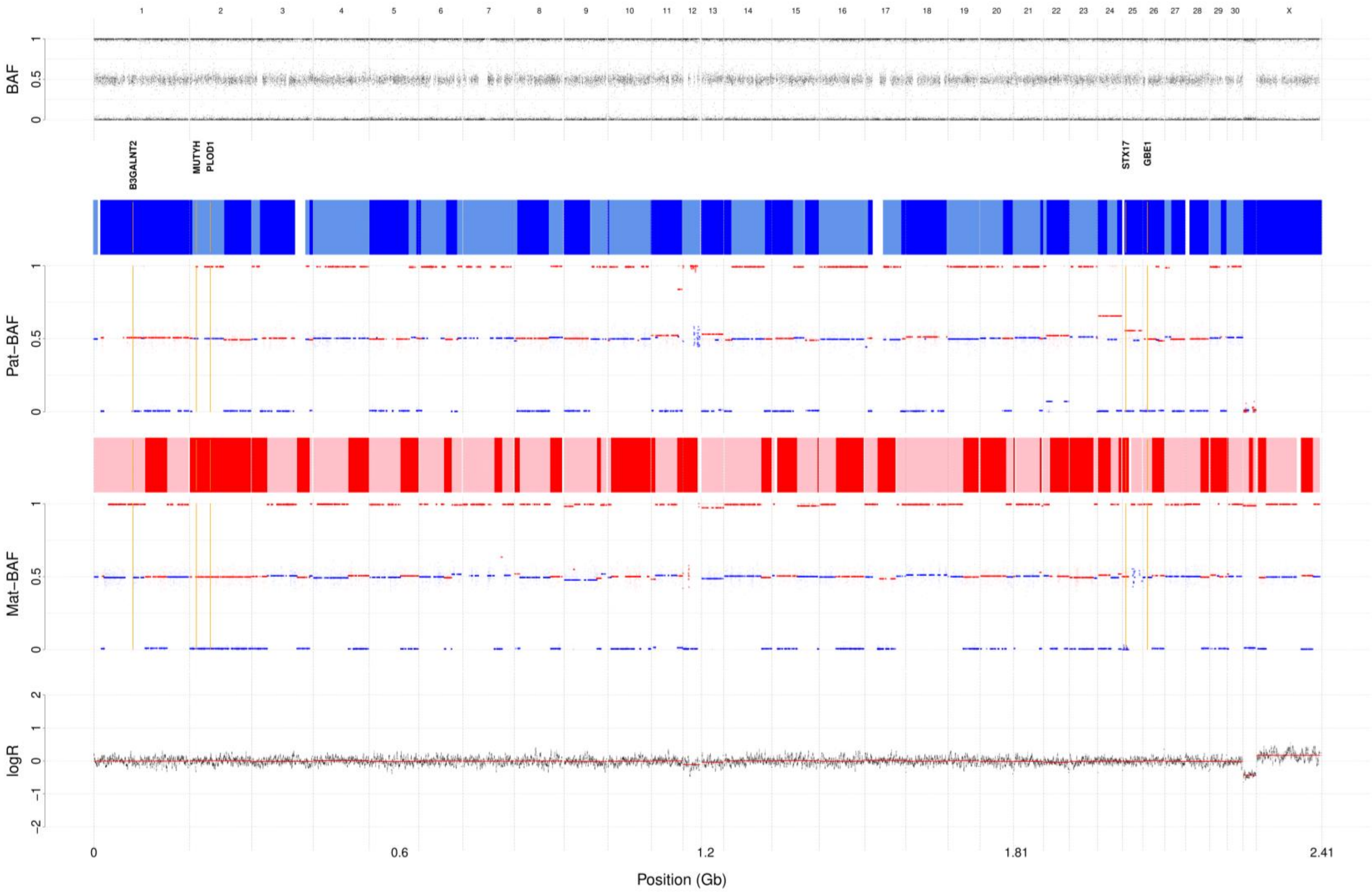

Mare01_Embryo02_Biopsy

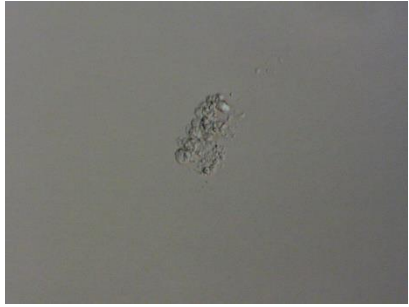

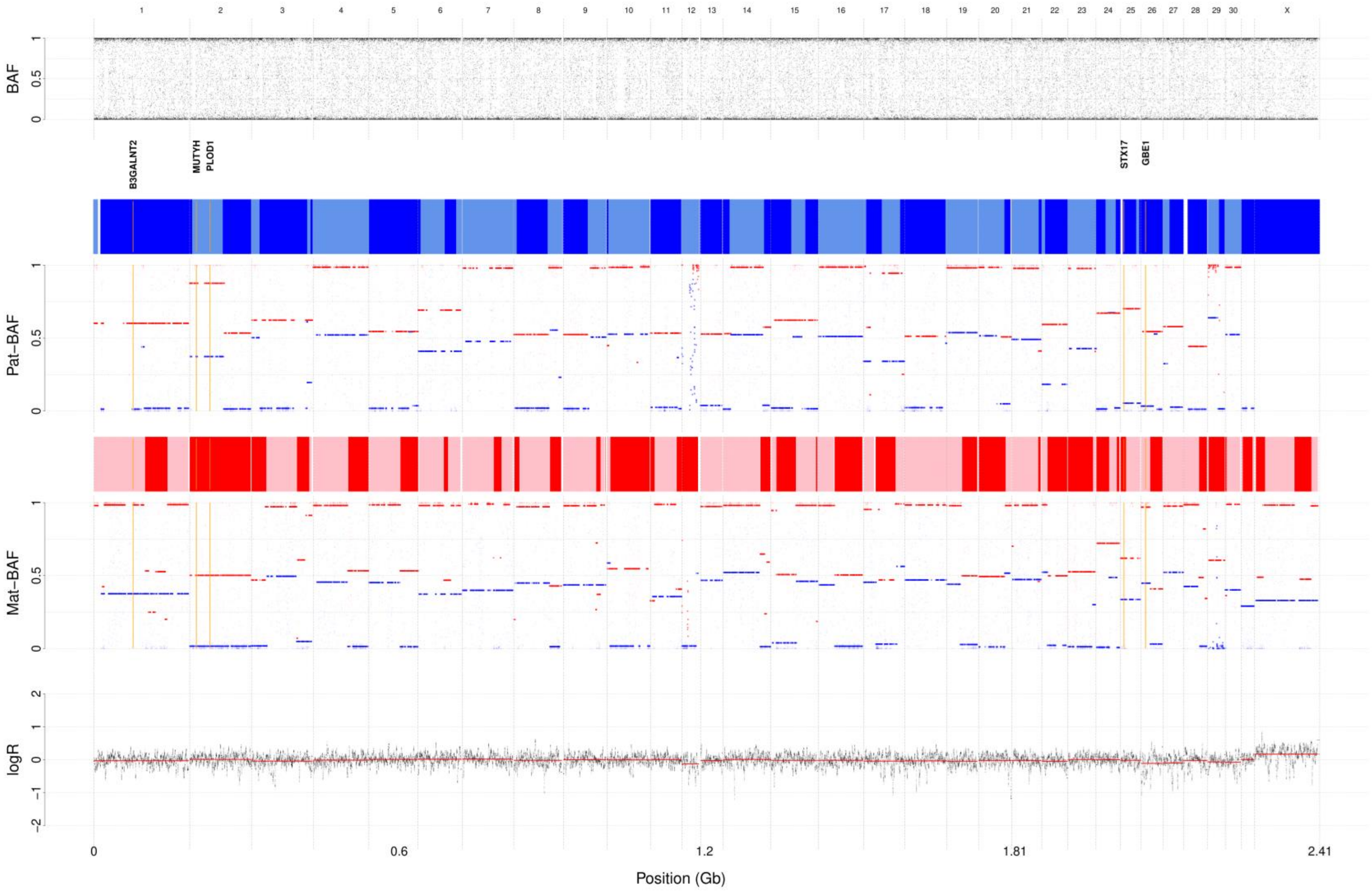

Mare01_Embryo02_Embryo

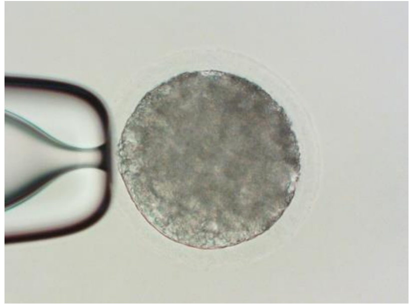

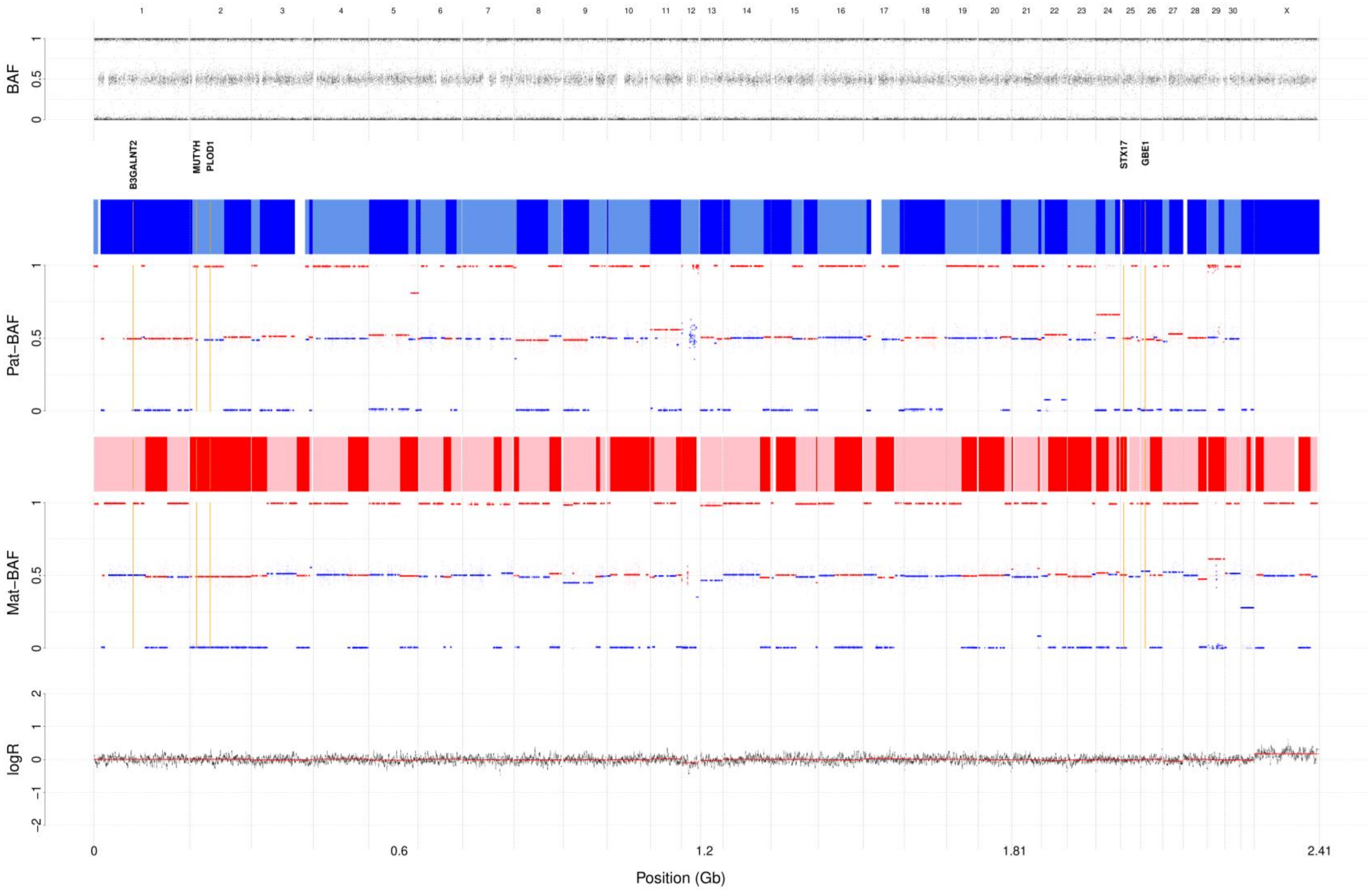

Mare01_Embryo03_Biopsy

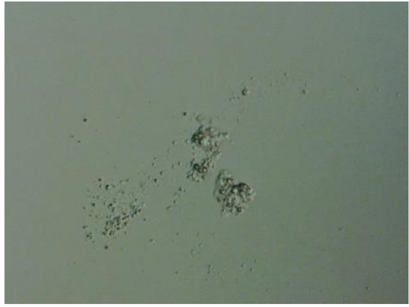

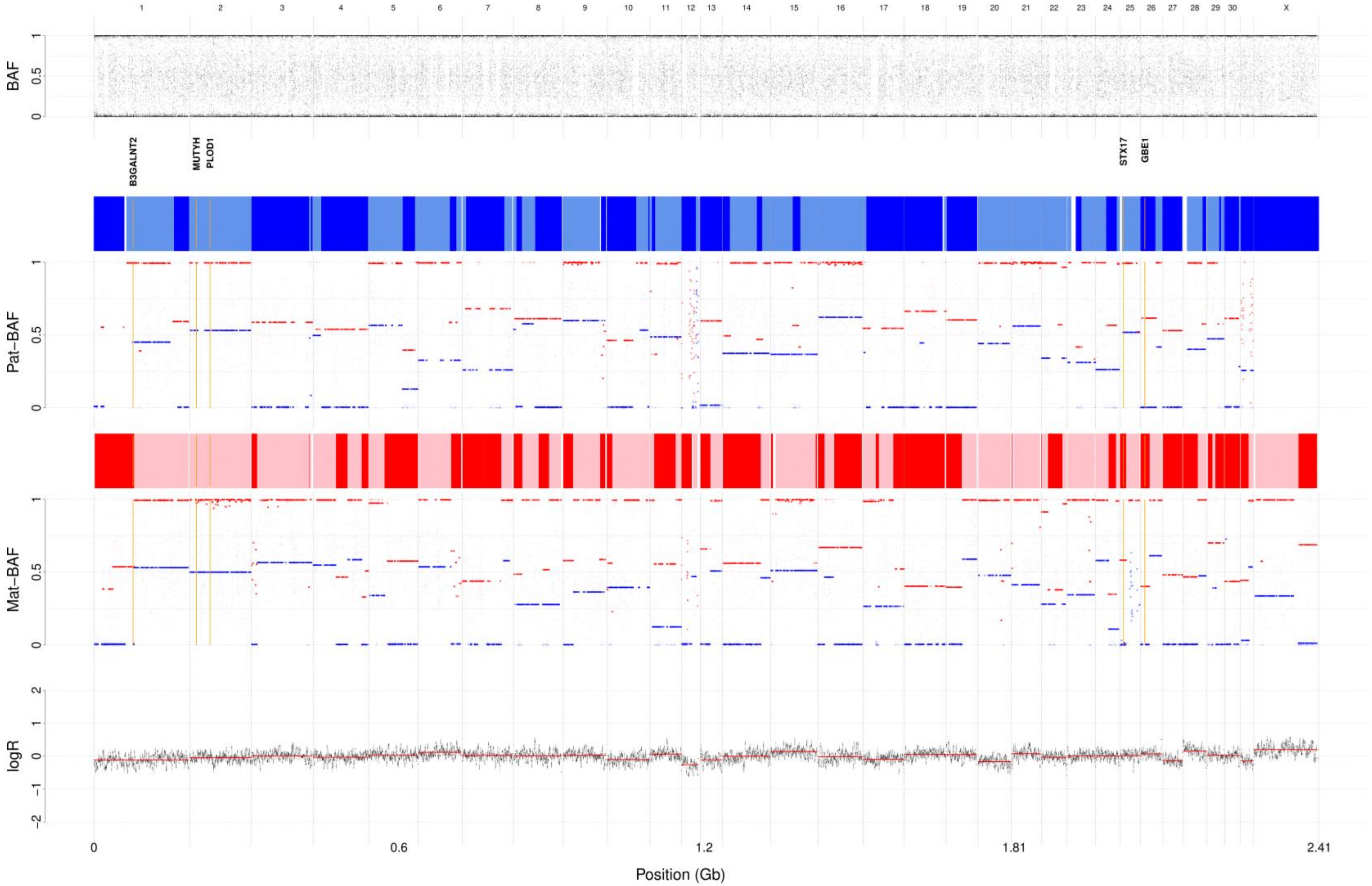

Mare01_Embryo03_Embryo

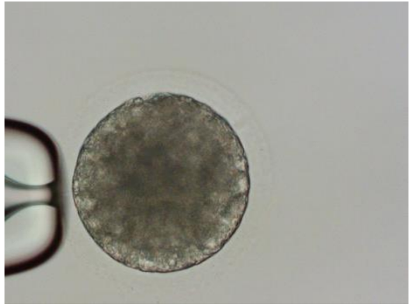

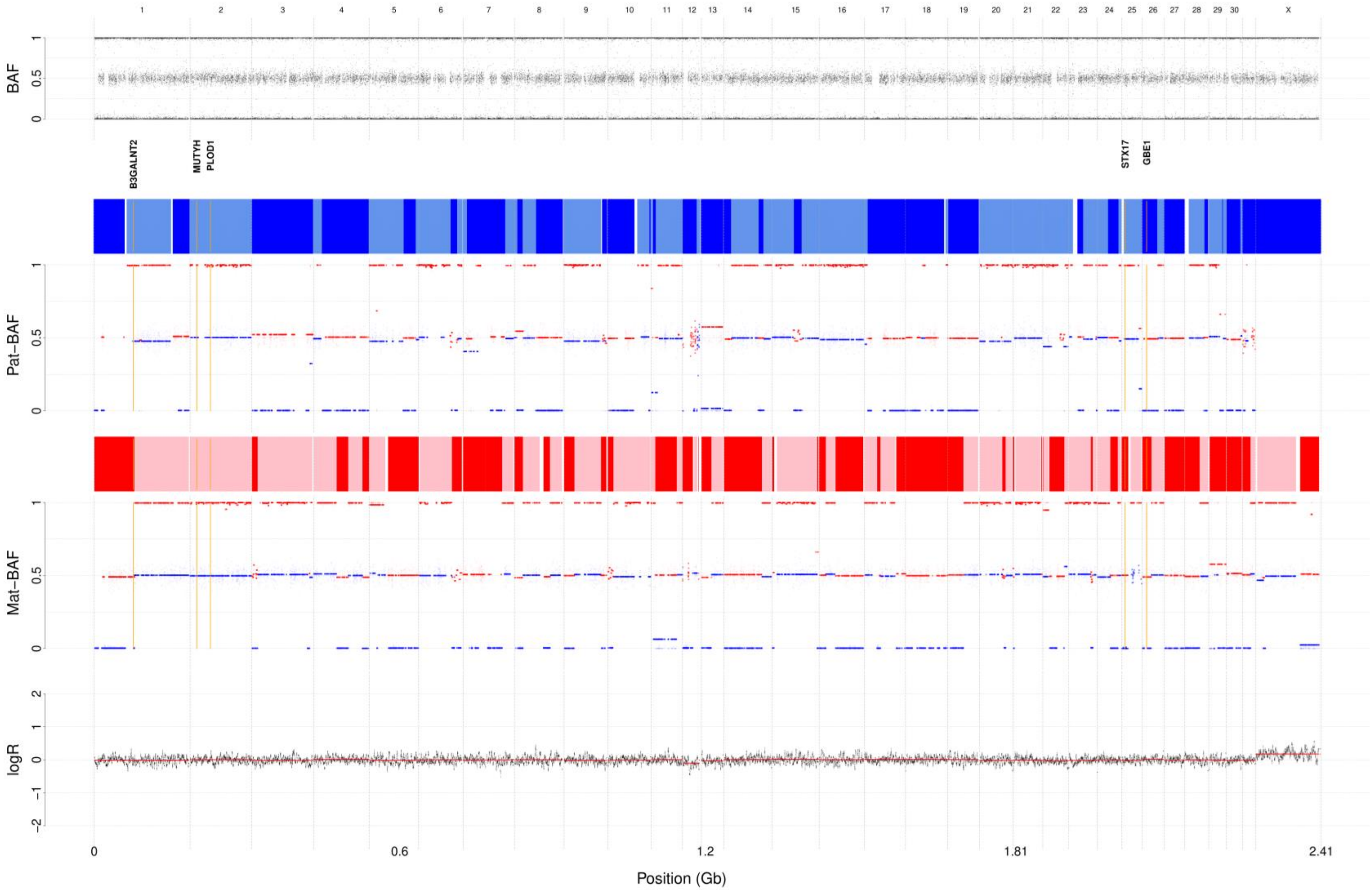

Mare01_Embryo04_Biopsy

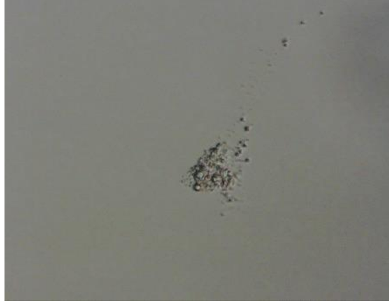

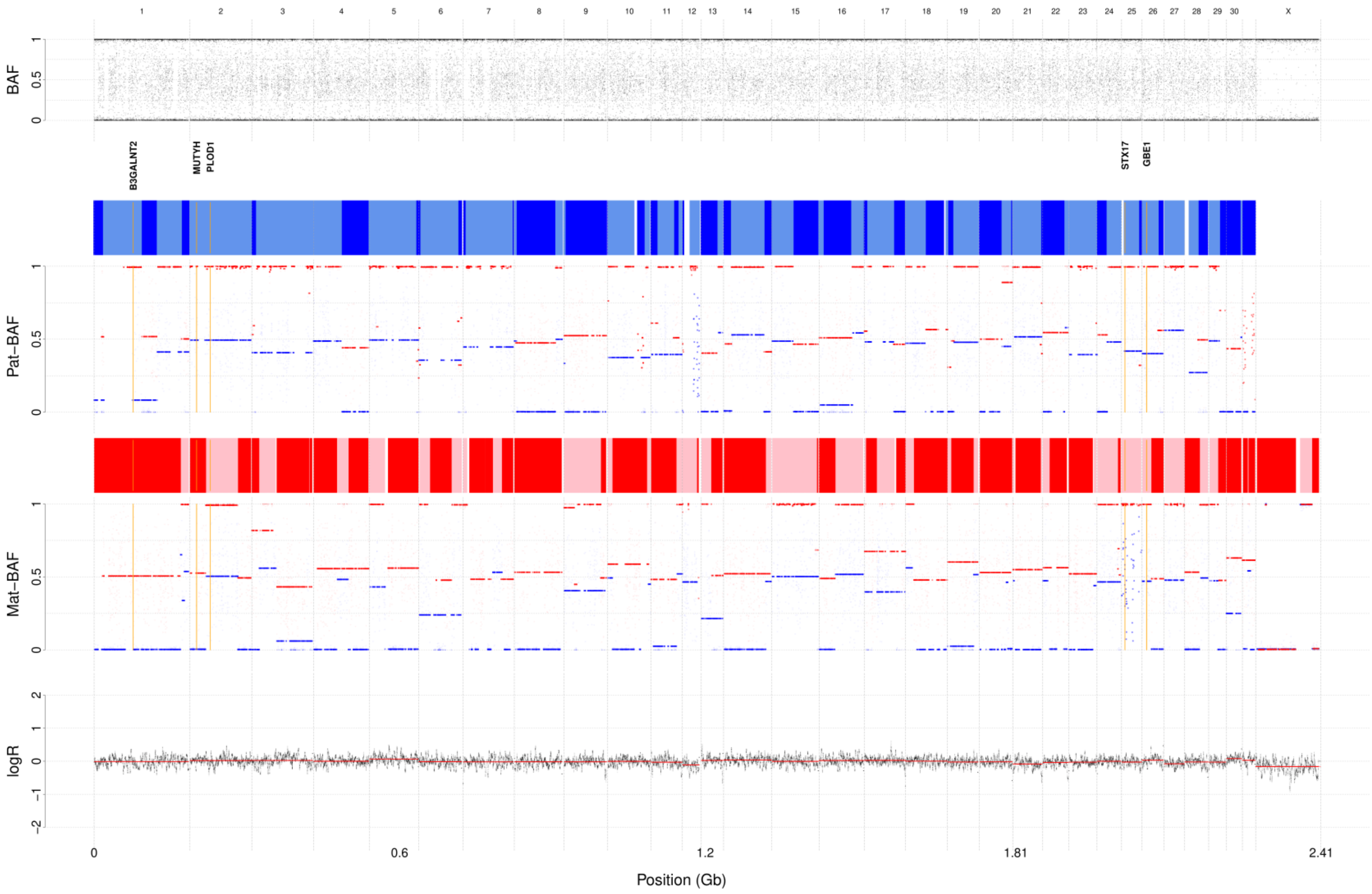

Mare01_Embryo04_Embryo

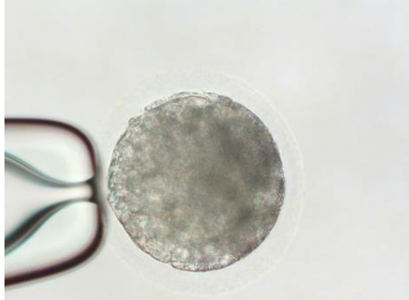

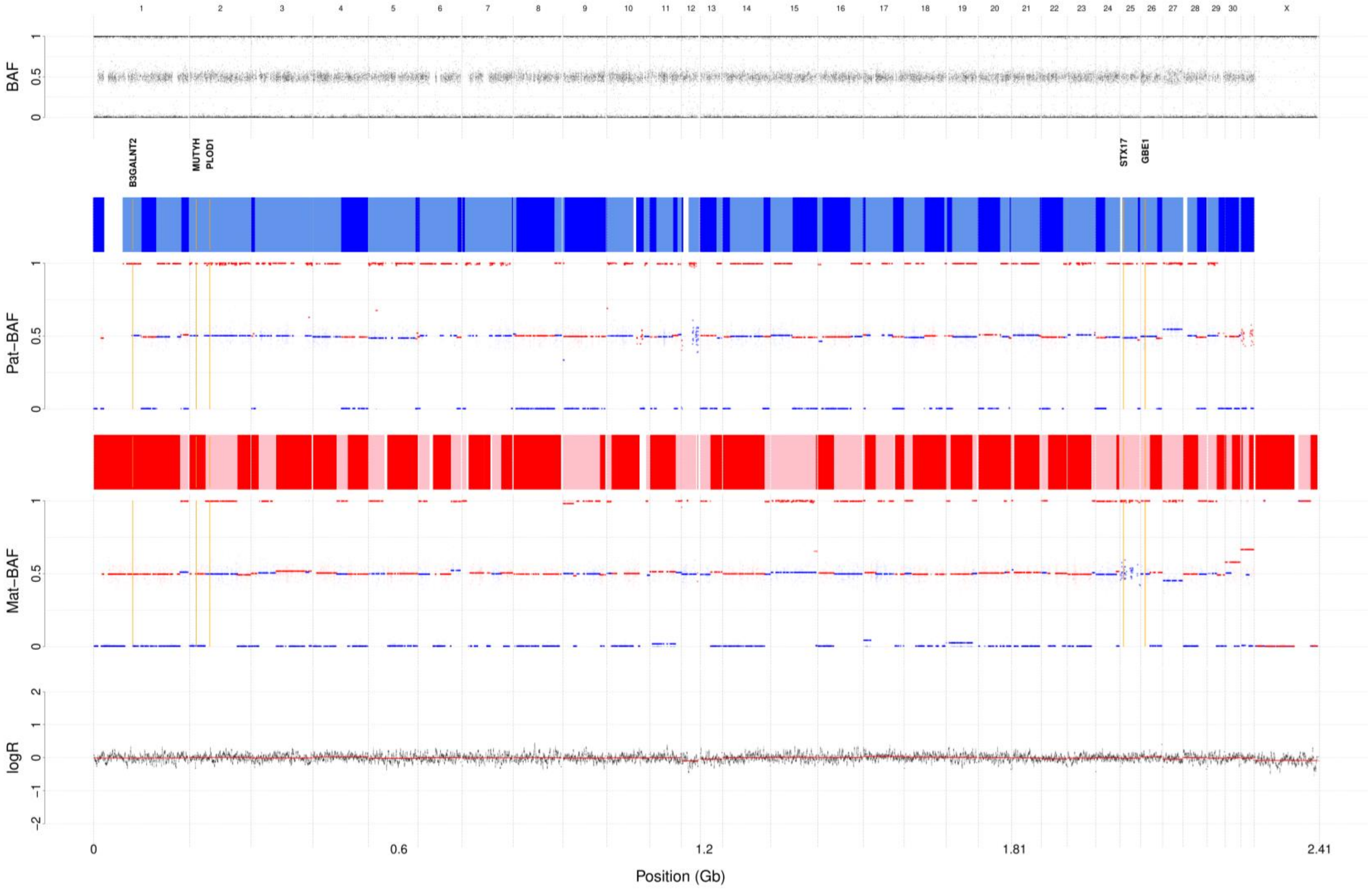

Mare02_Embryo01_Biopsy

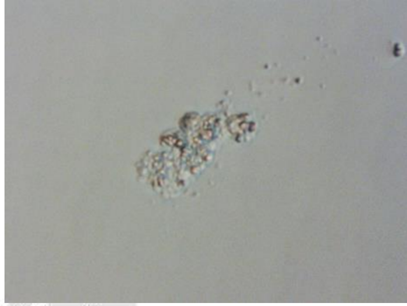

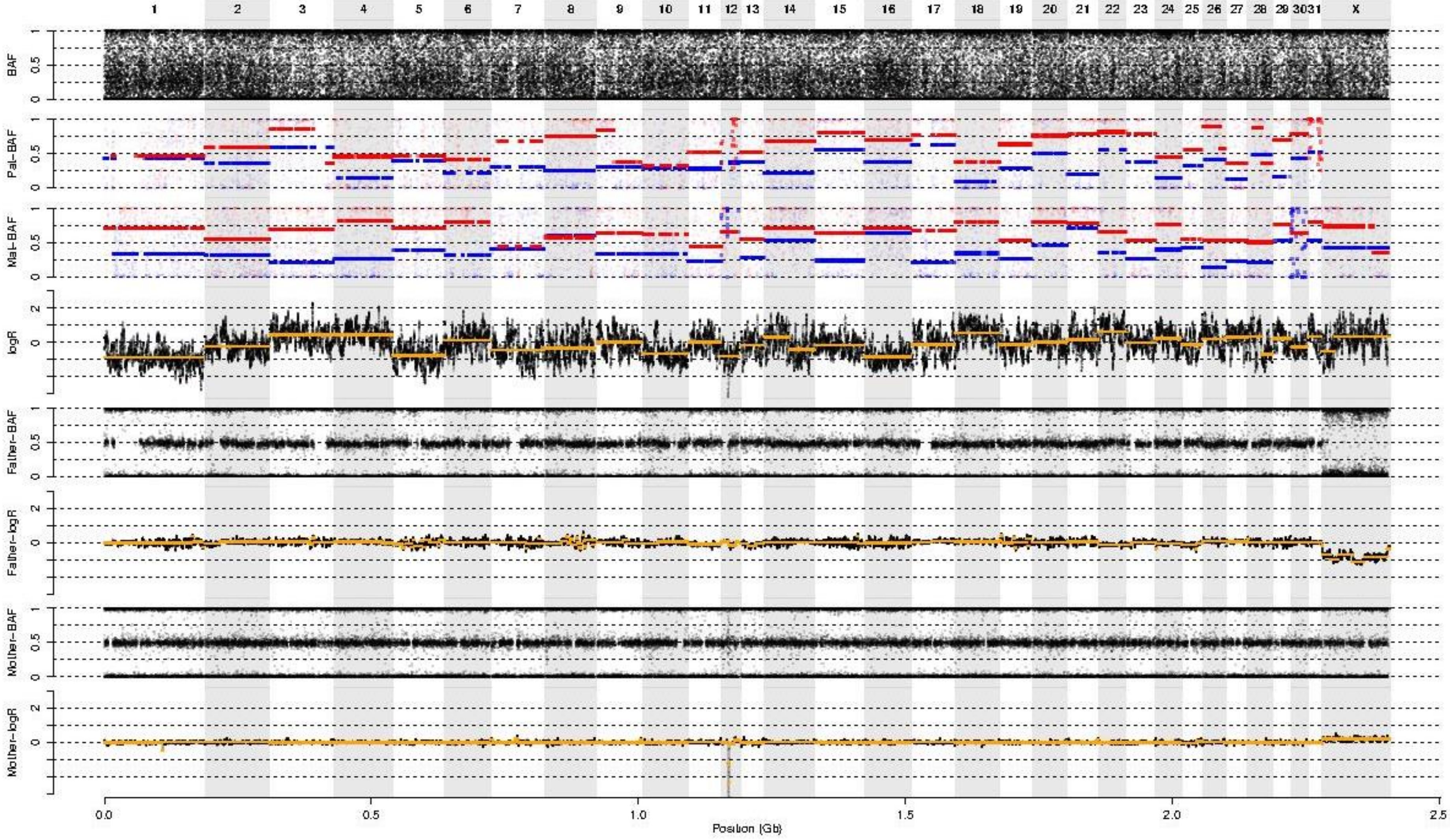

Mare02_Embryo01_Embryo

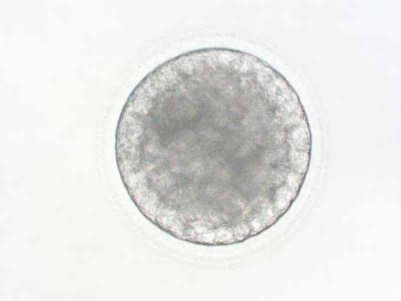

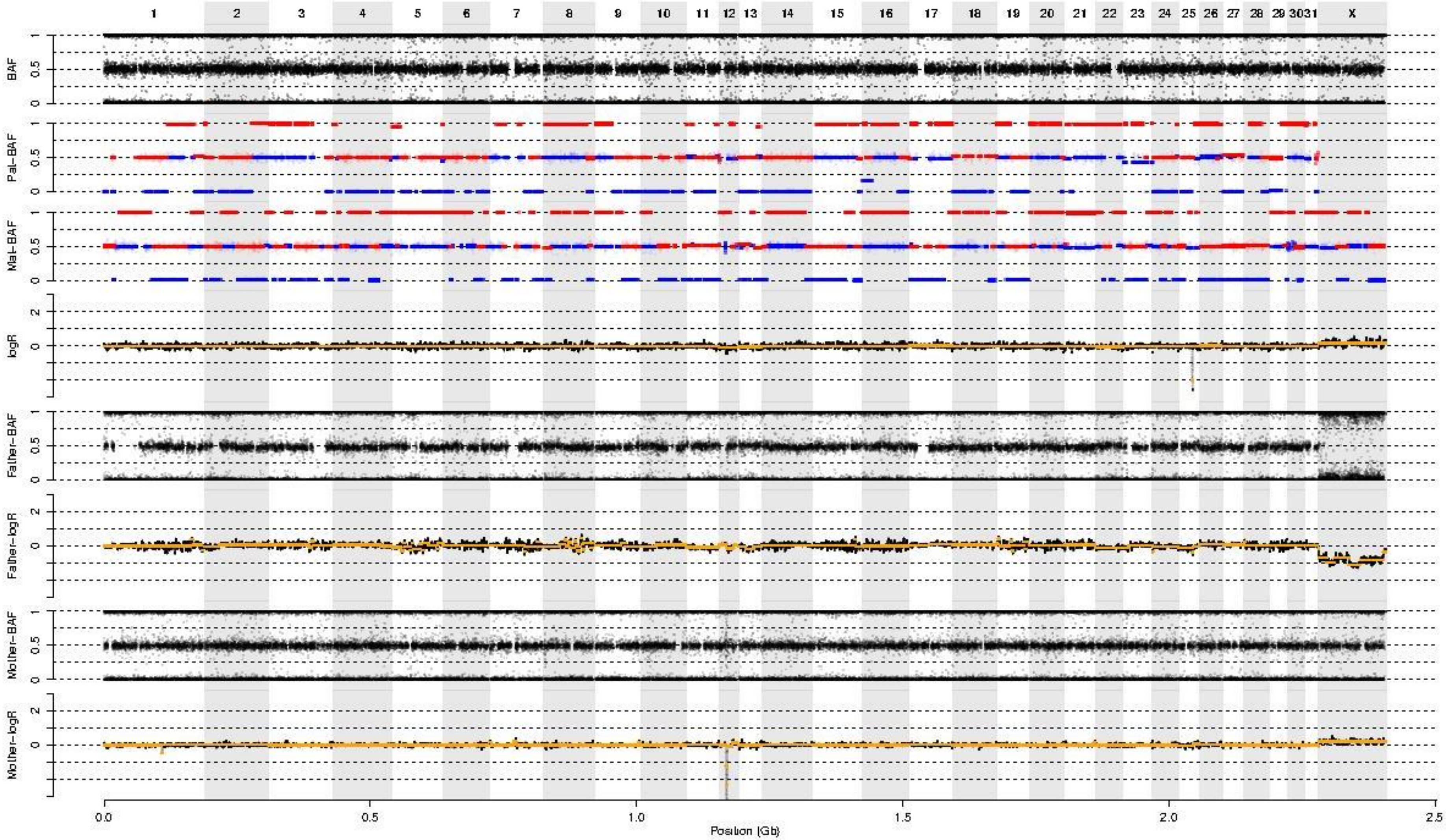

Mare02_Embryo02_Biopsy

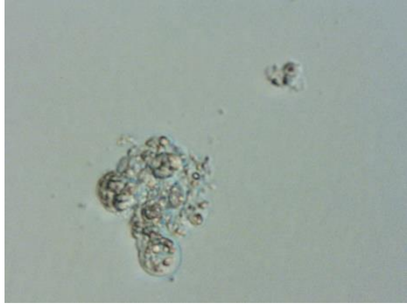

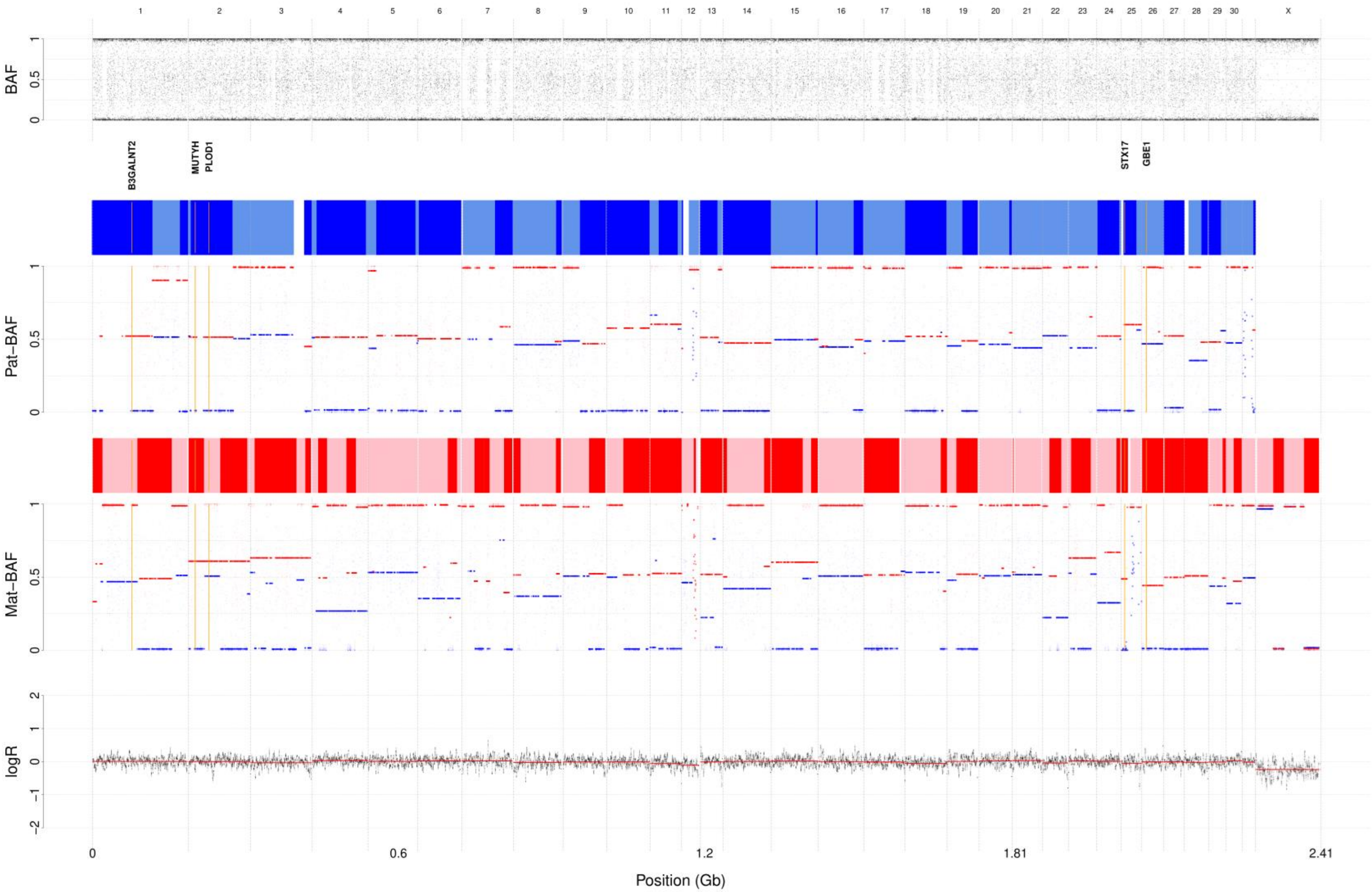

Mare02_Embryo02_Embryo

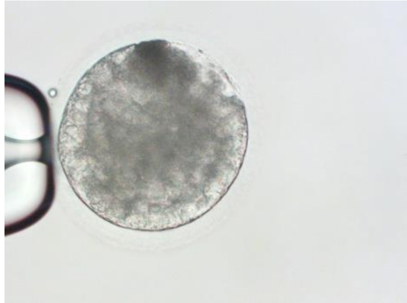

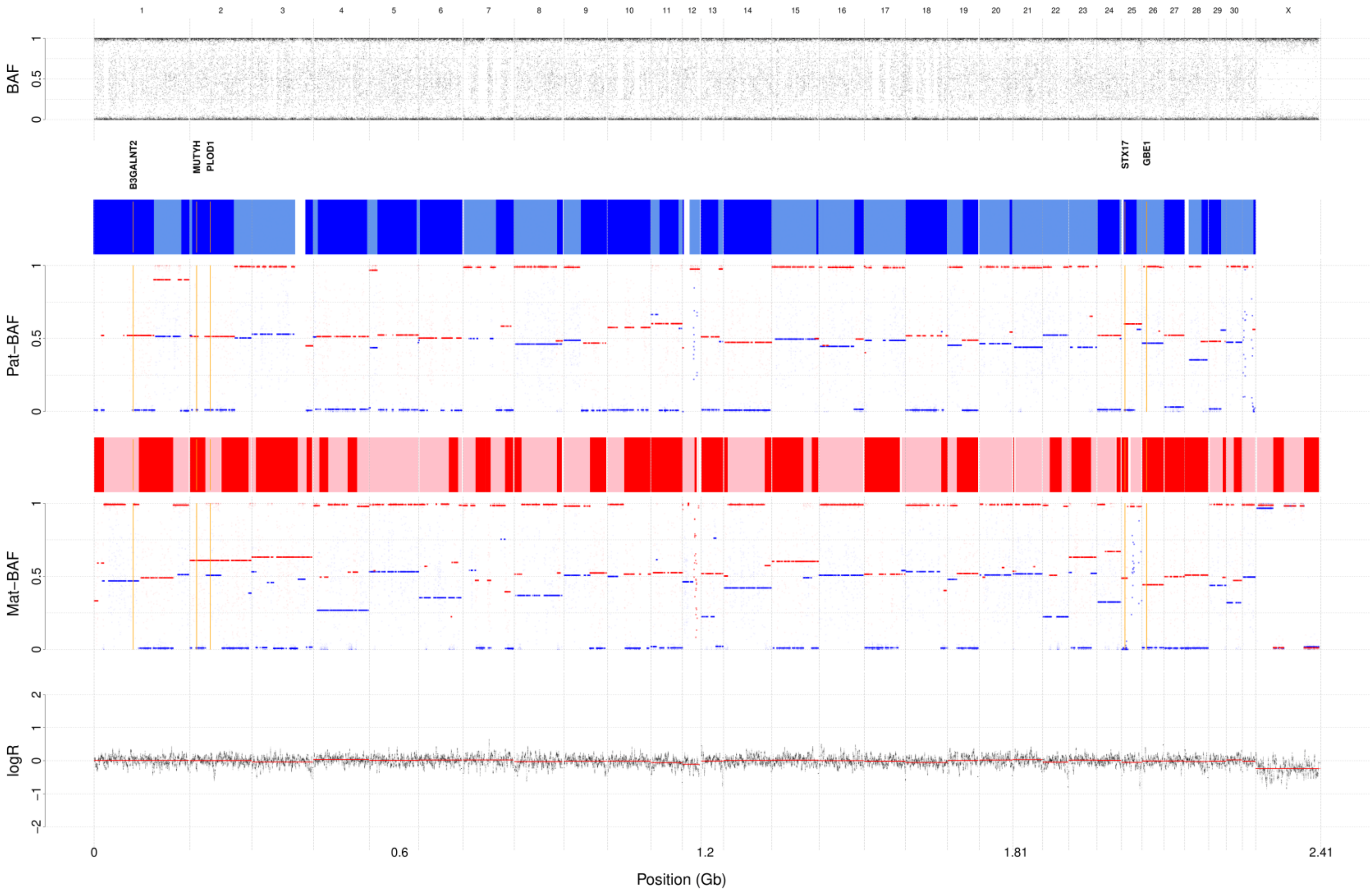

Mare02_Embryo03_Biopsy

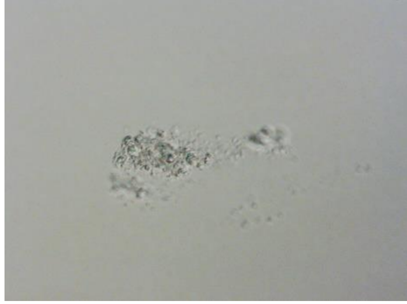

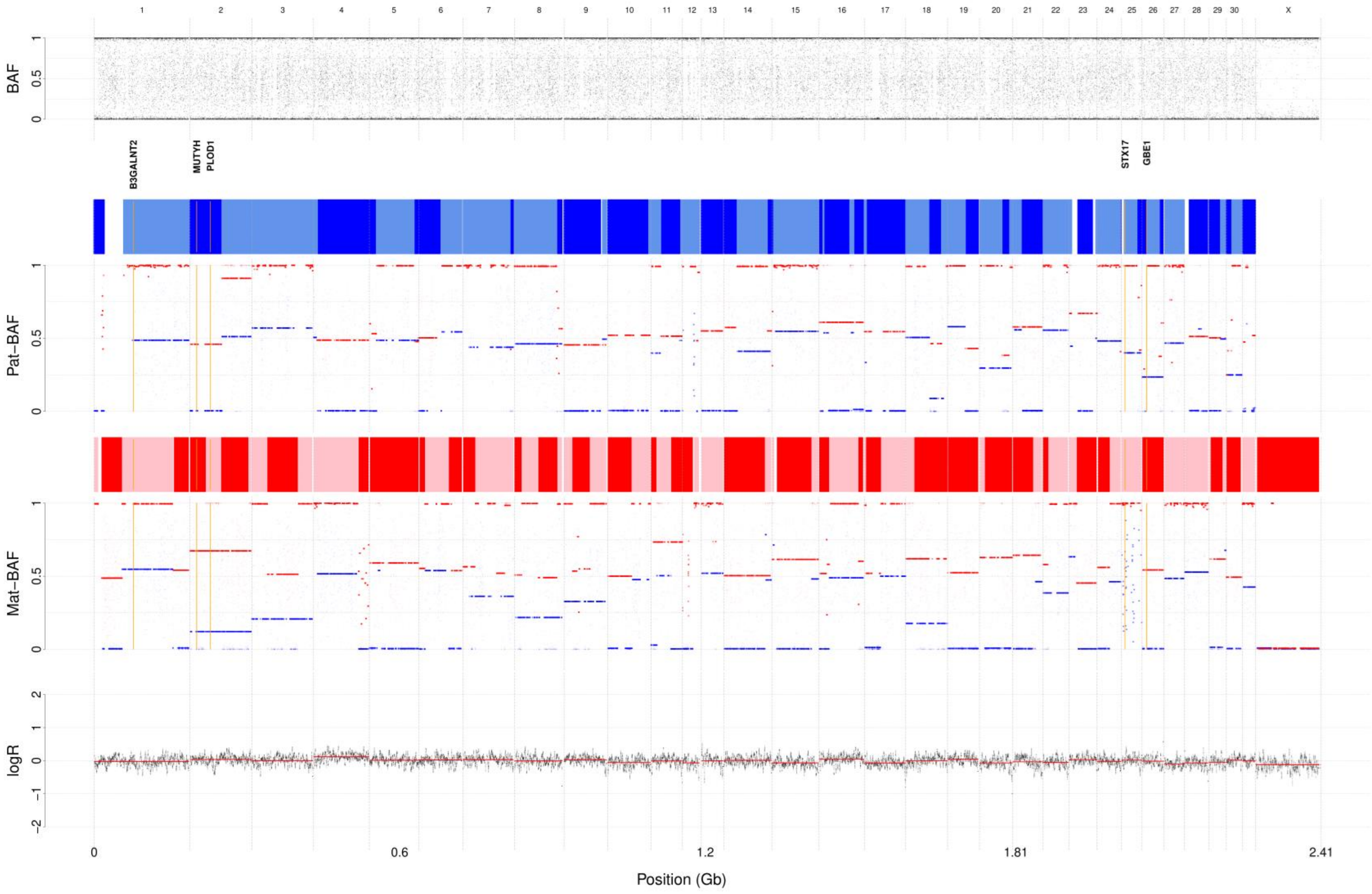

Mare02_Embryo03_Embryo

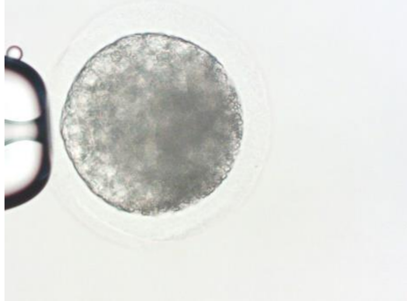

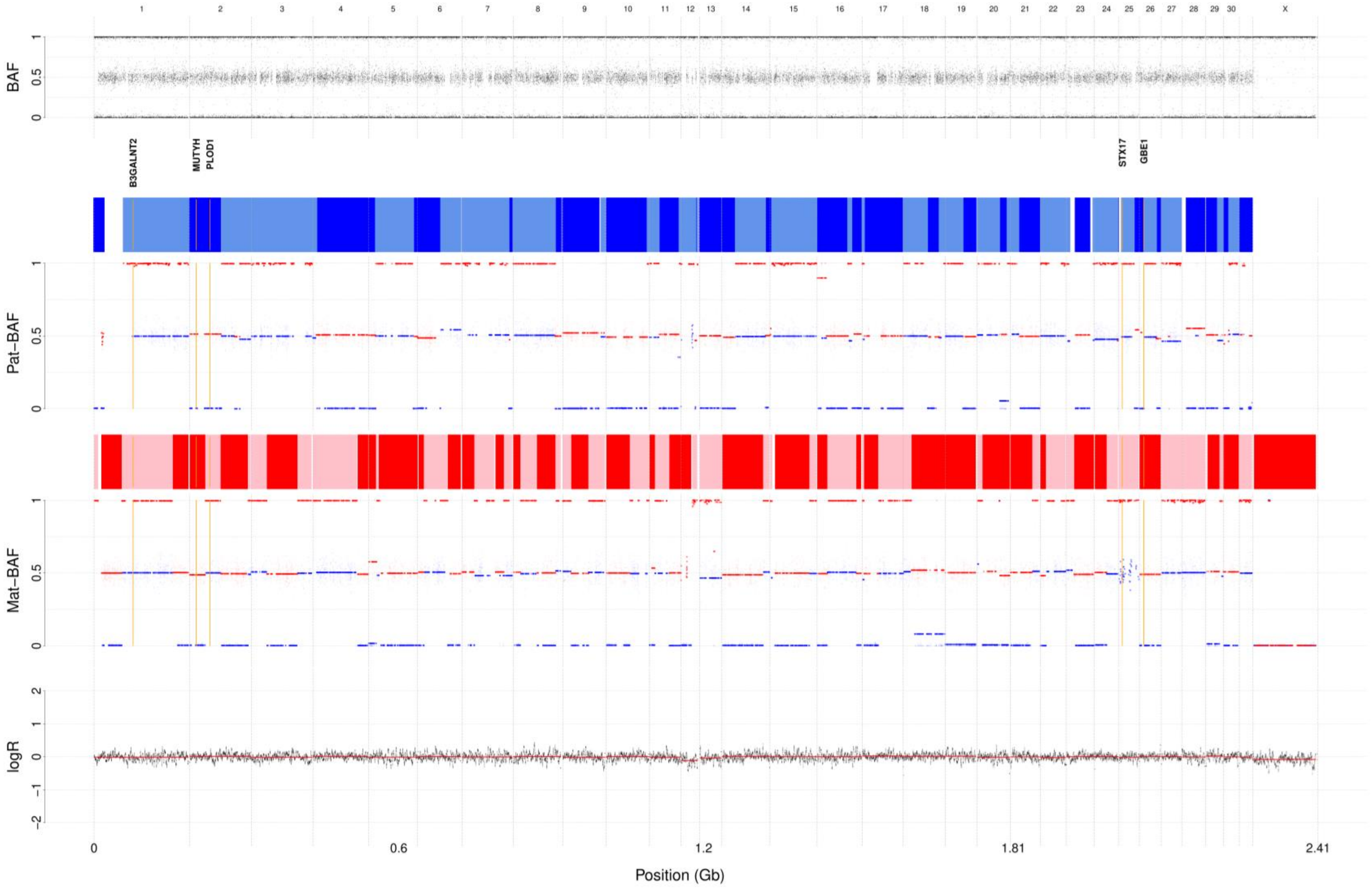

Mare02_Embryo04_Biopsy

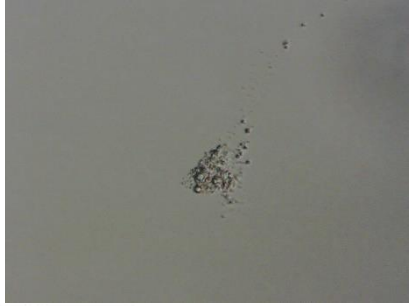

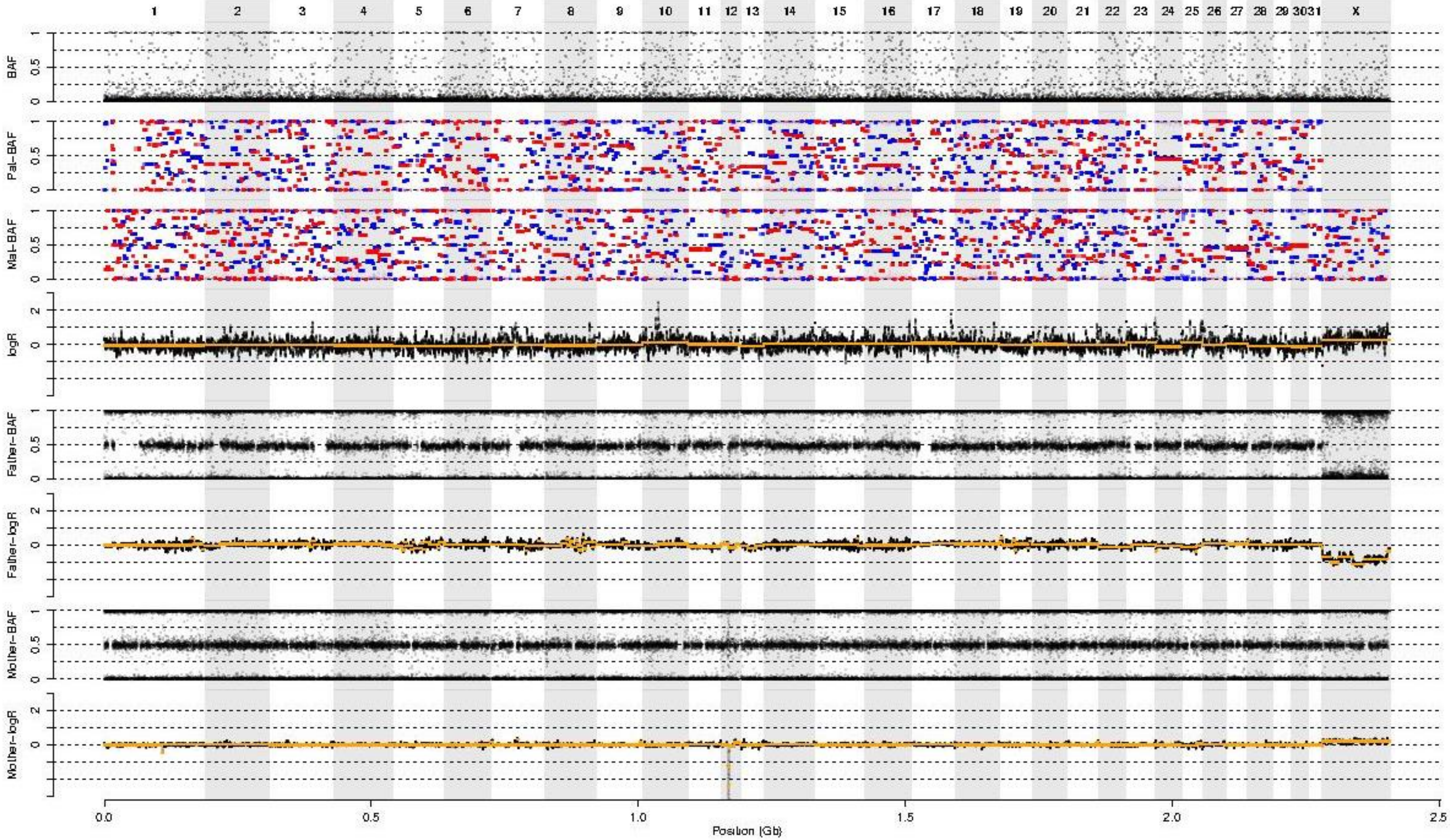

Mare02_Embryo04_Embryo

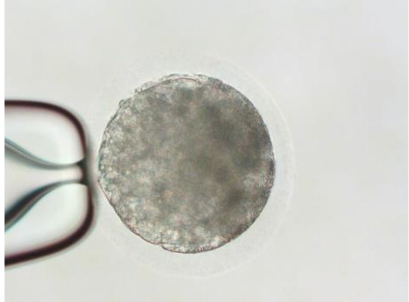

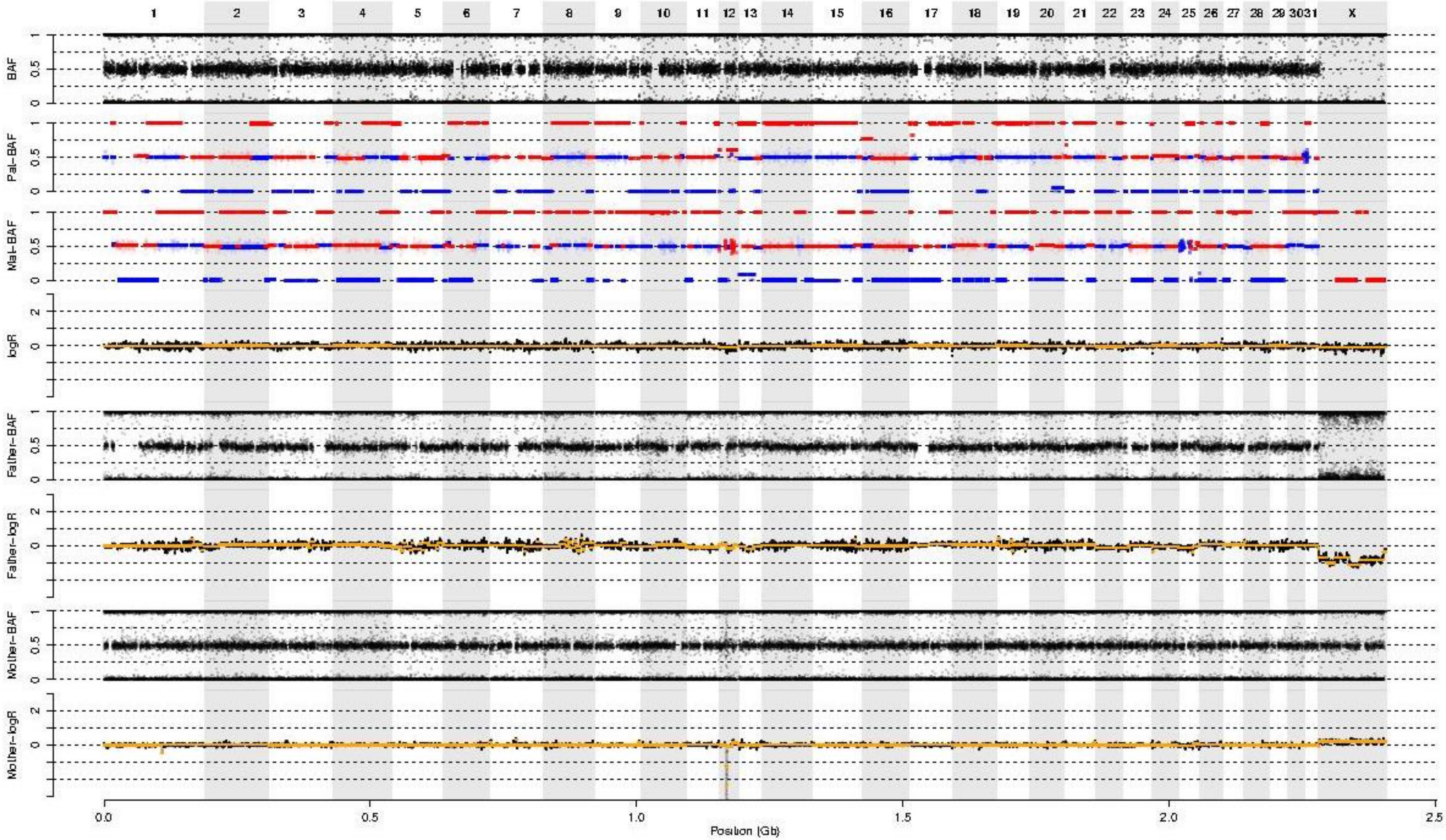

Mare02_Embryo05_Biopsy

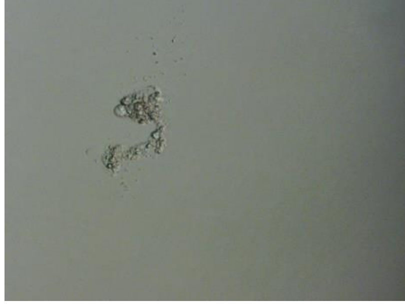

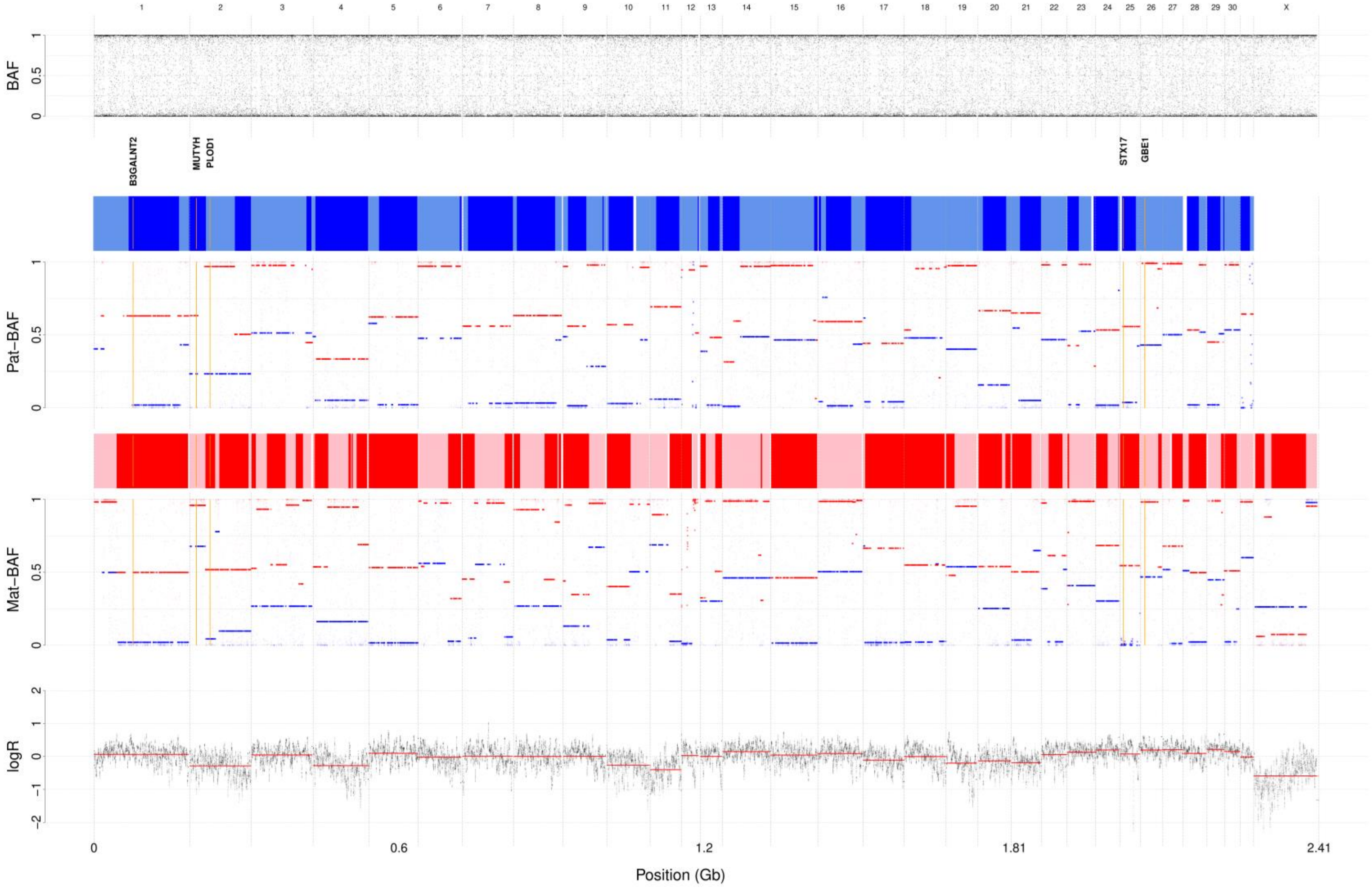

Mare02_Embryo05_Embryo

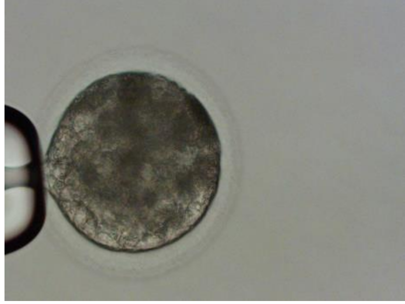

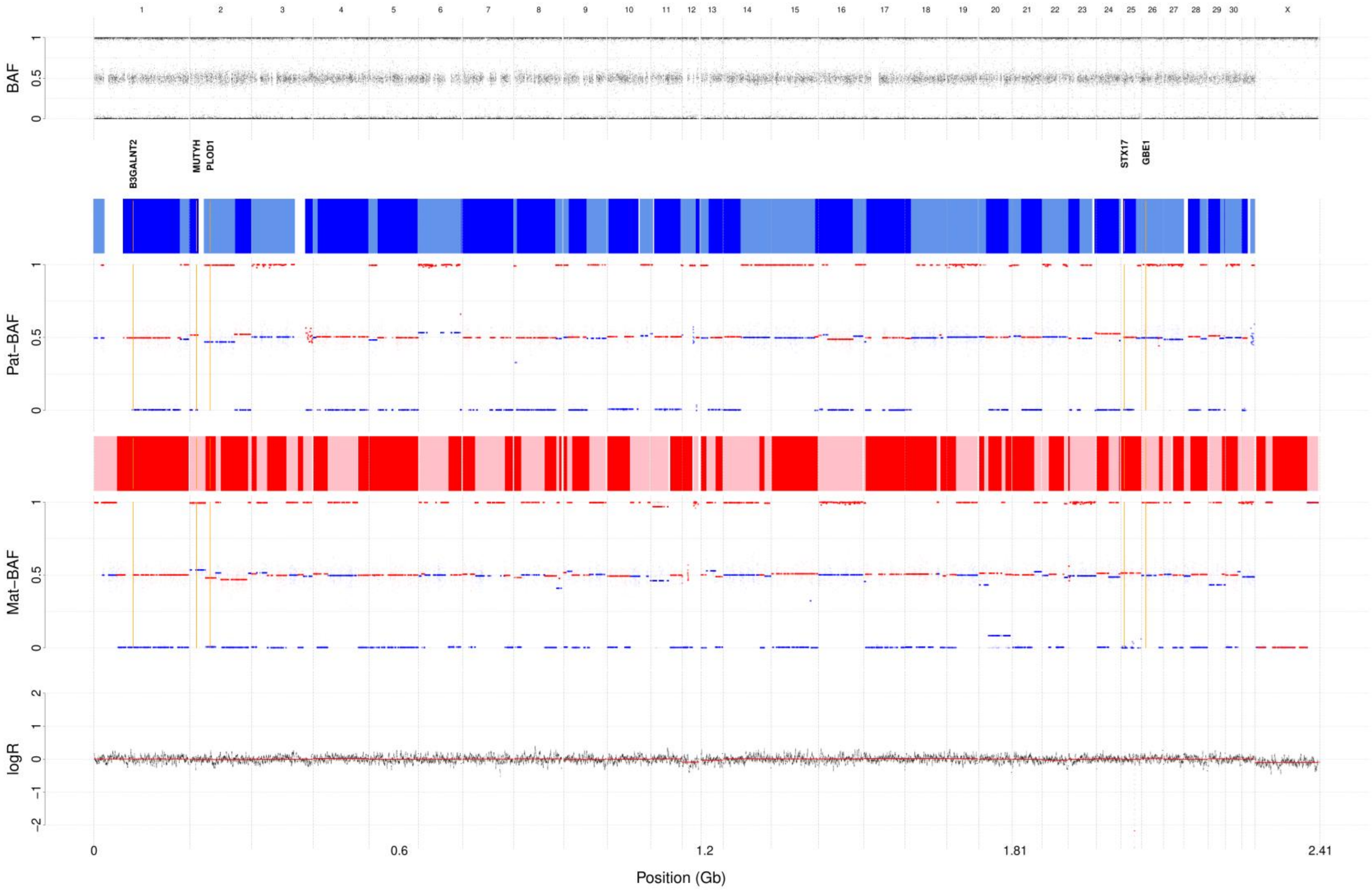

Mare02_Embryo06_Biopsy

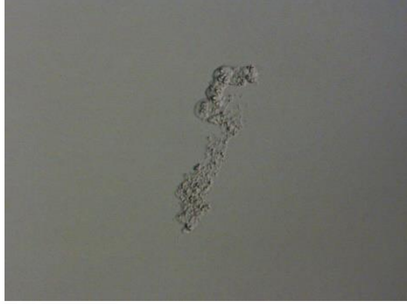

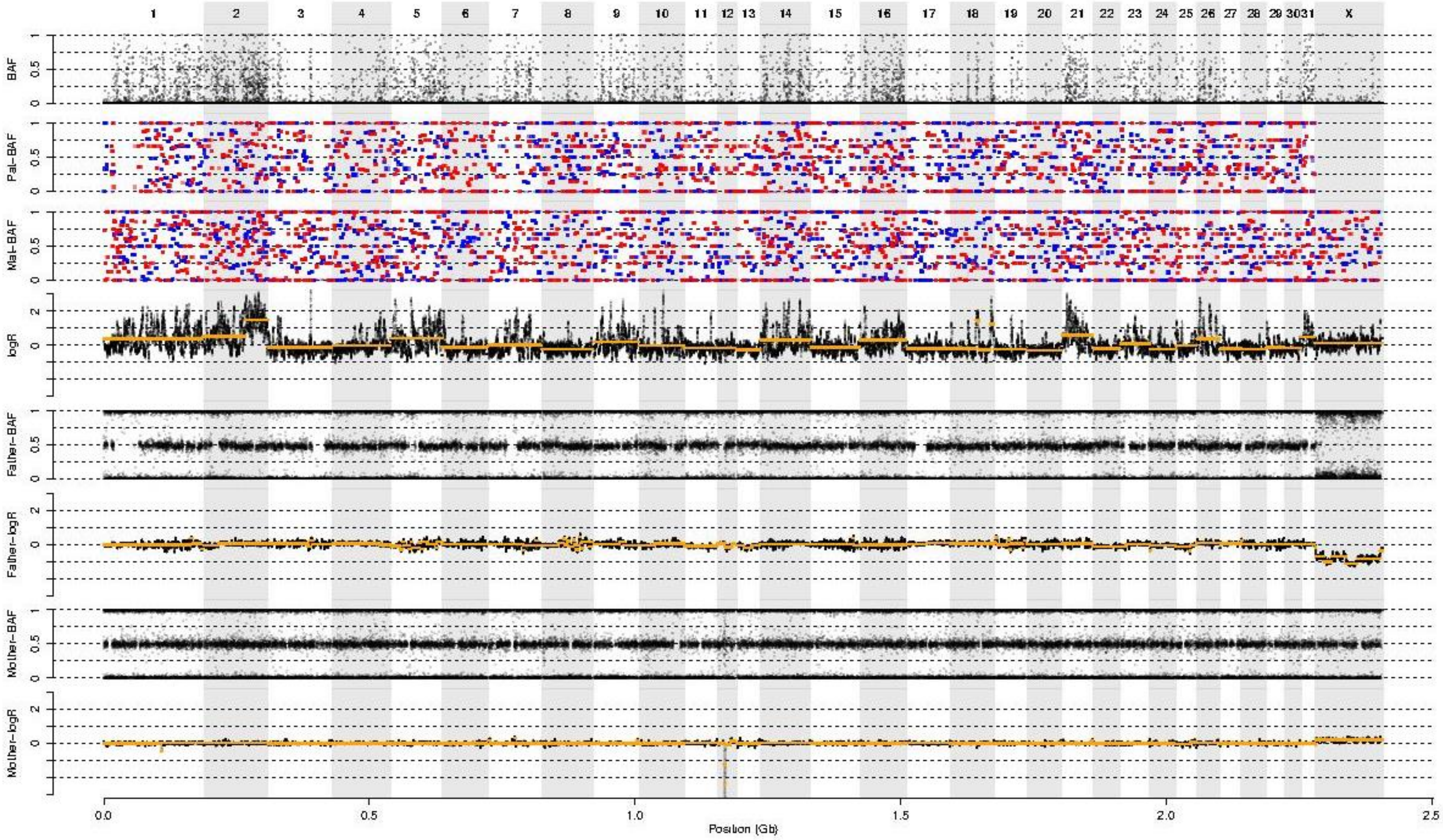

Mare02_Embryo06_Embryo

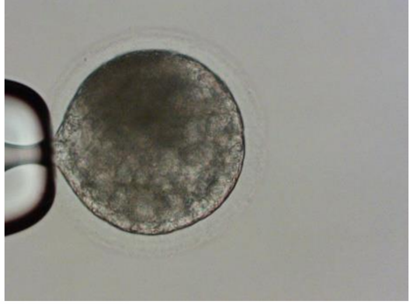

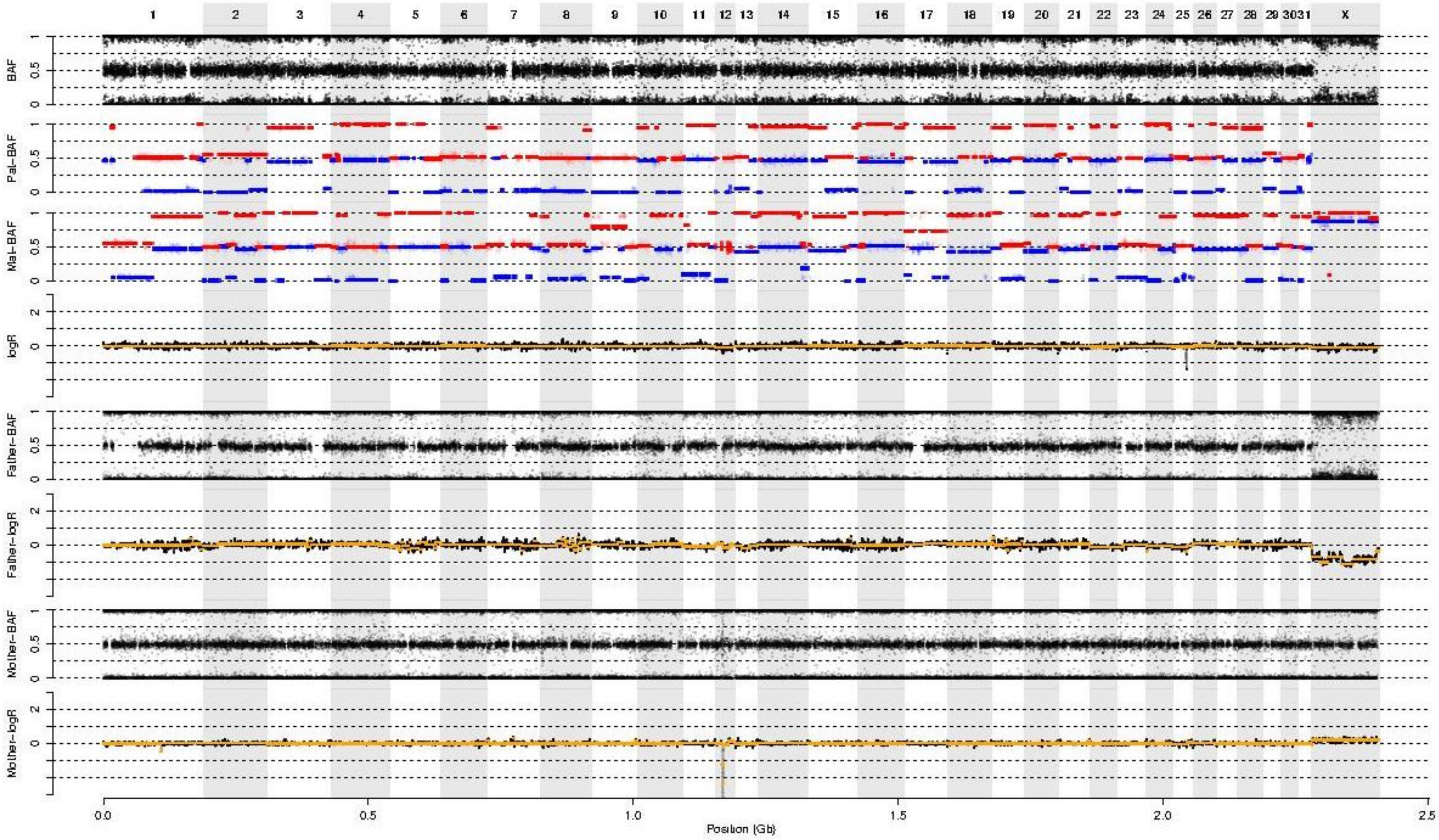

Mare02_Embryo07_Biopsy

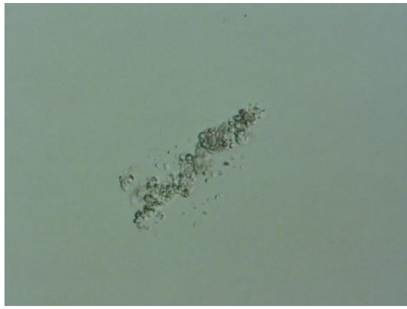

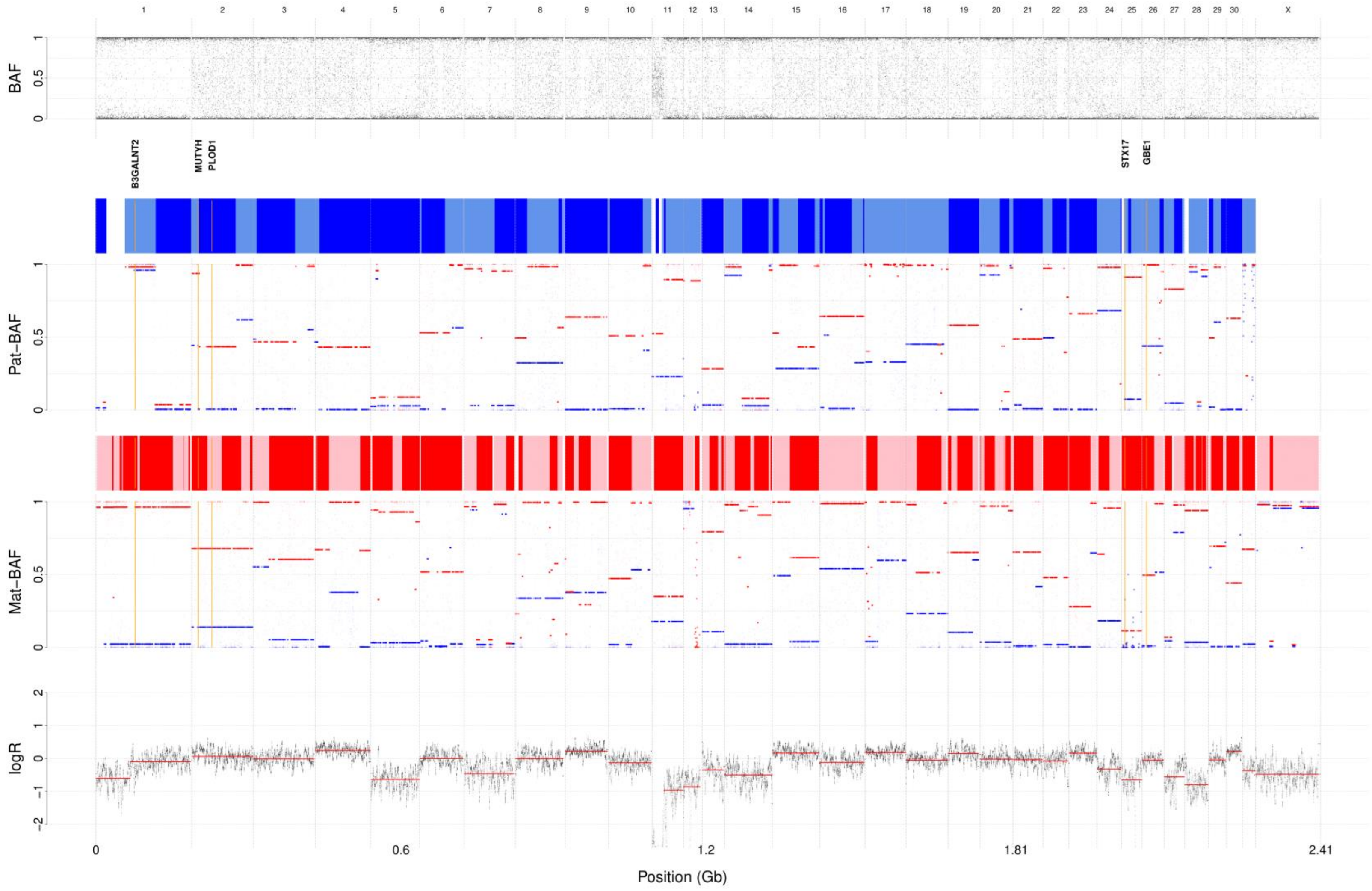

Mare02_Embryo07_Embryo

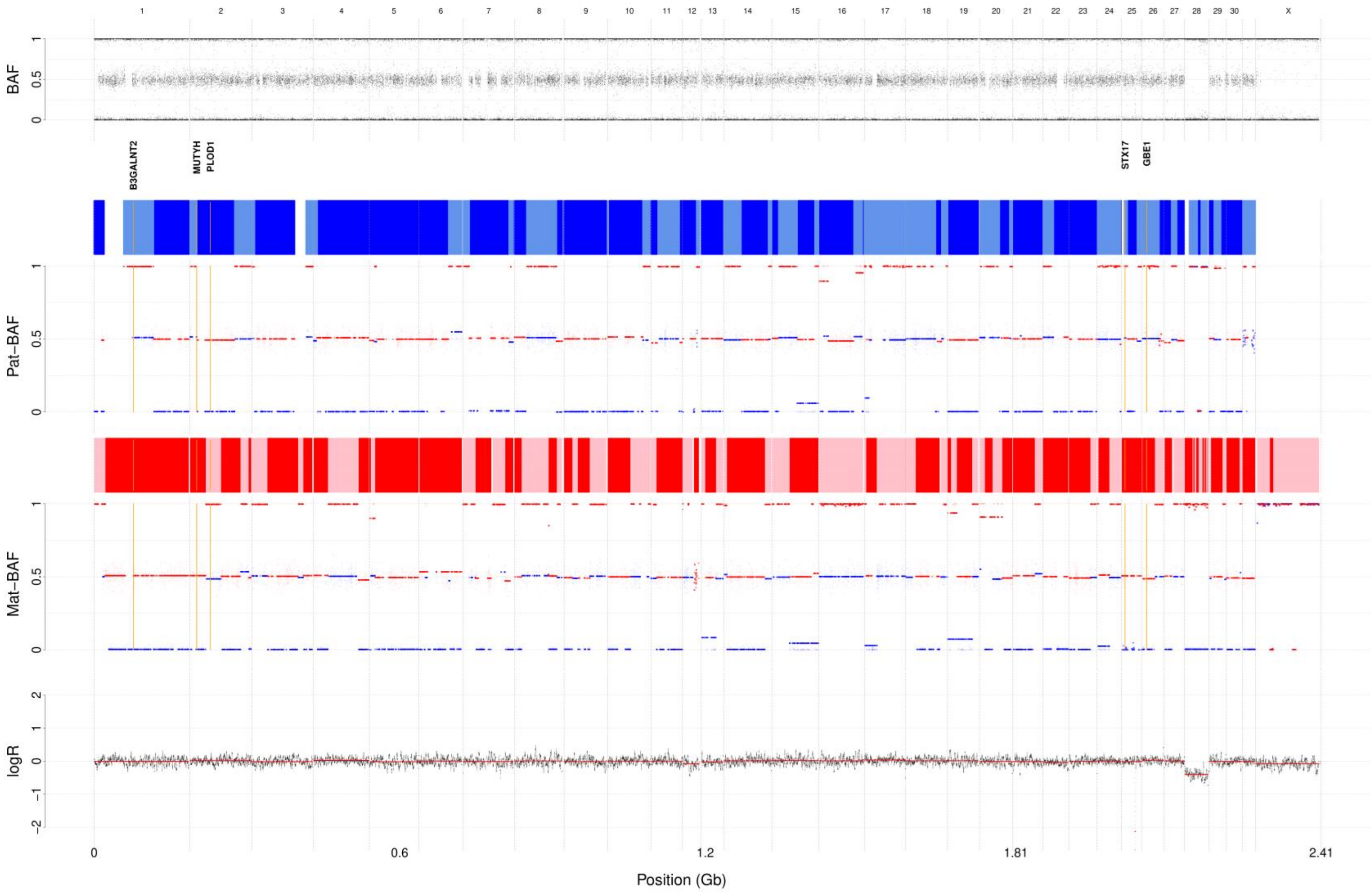

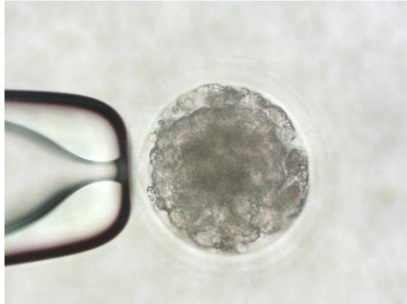

Mare03_Embryo01_Biopsy

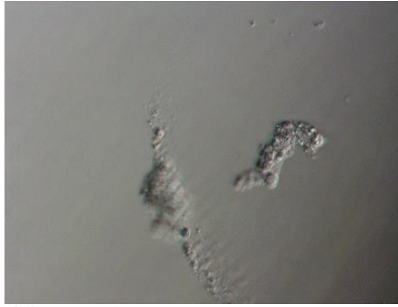

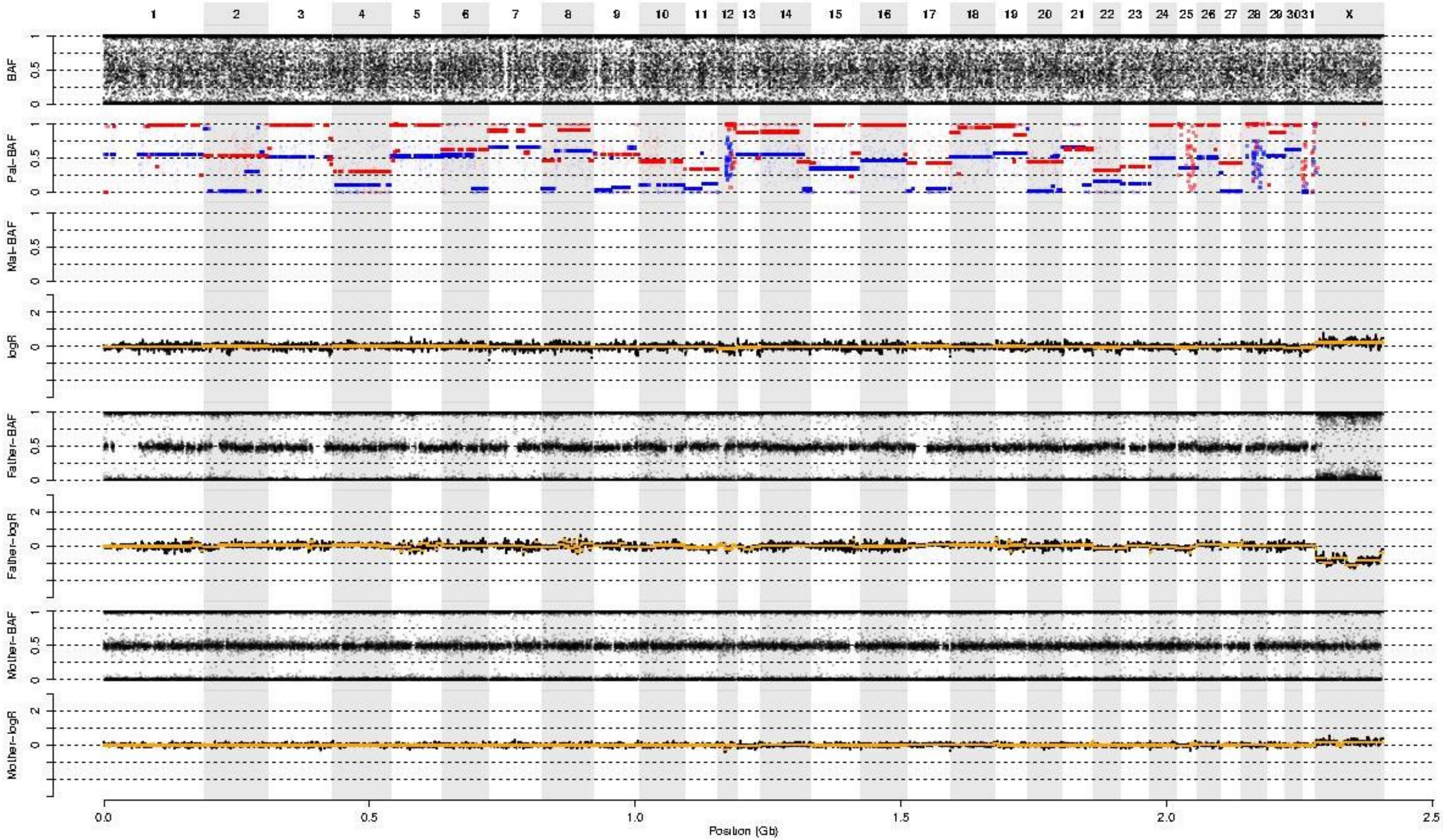

Mare03_Embryo01_Embryo

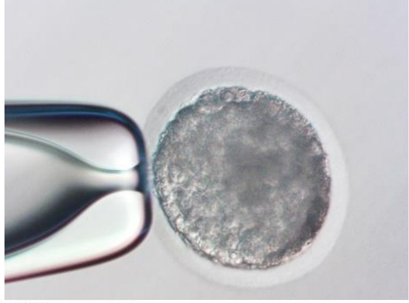

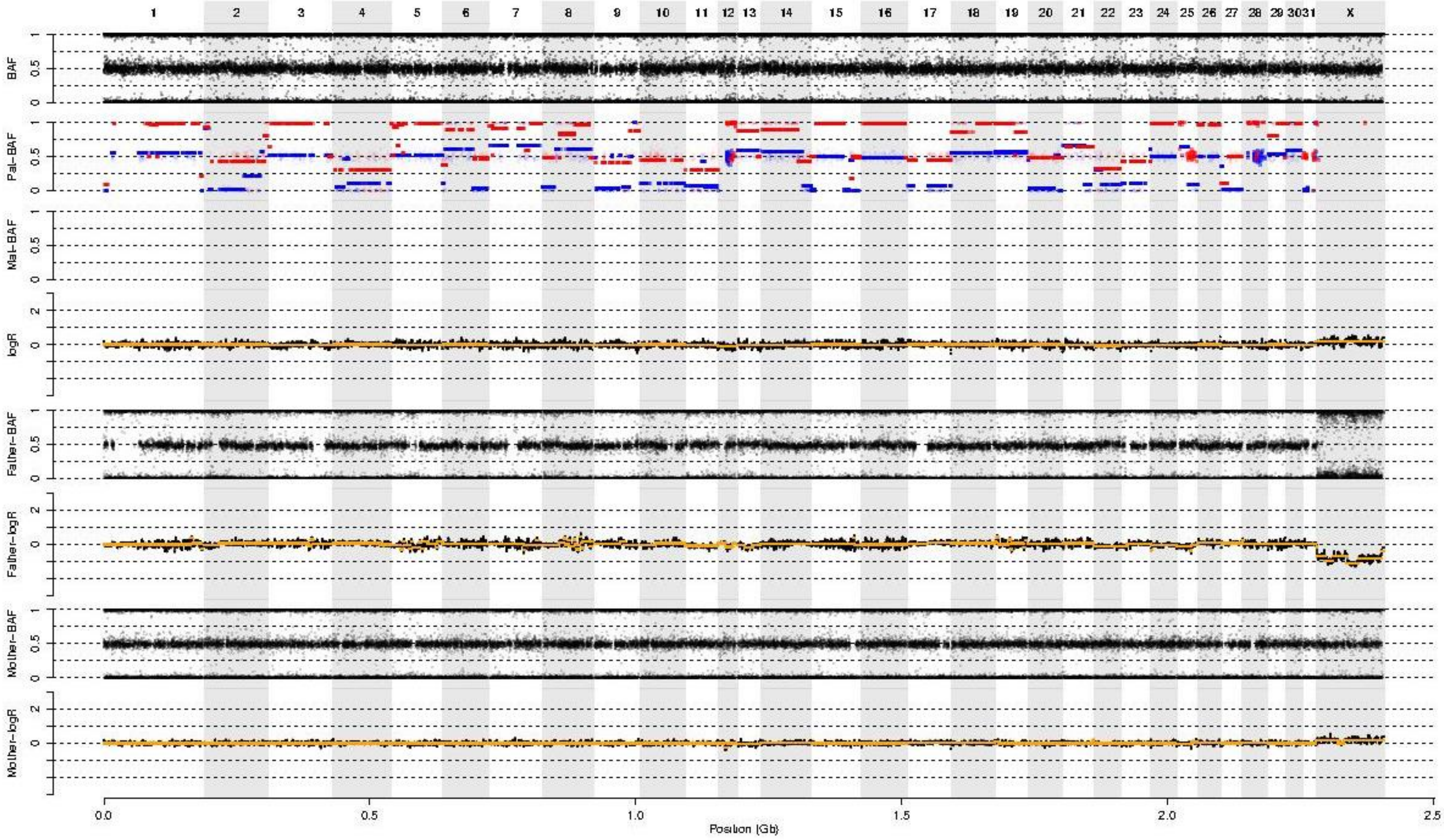

Mare04_Embryo01_Biopsy

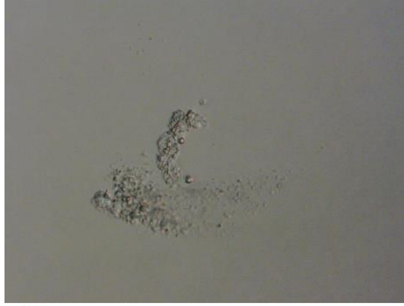

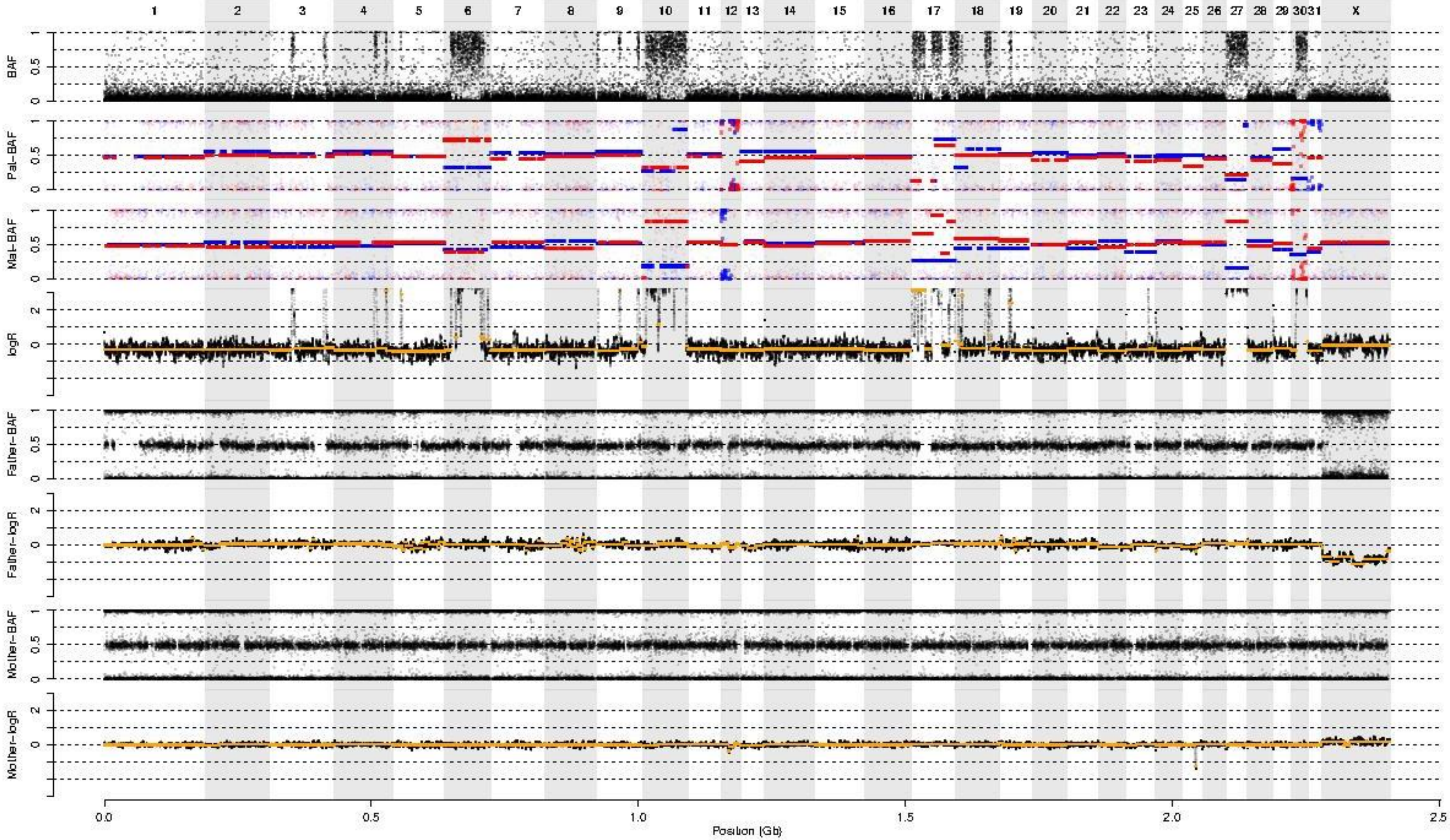

Mare04_Embryo01_Embryo

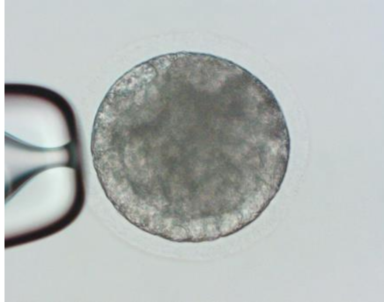

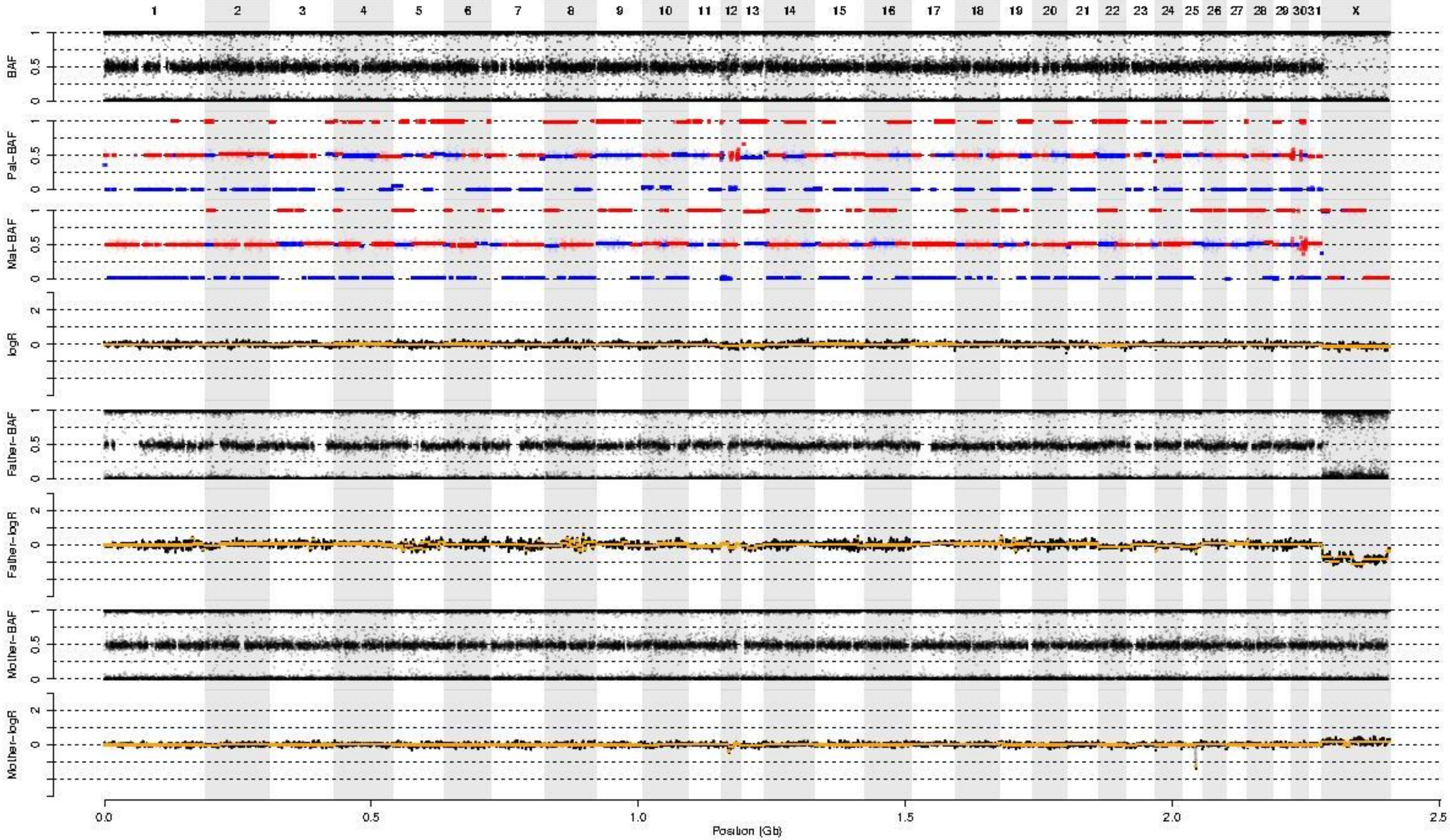

Mare04_Embryo02_Biopsy

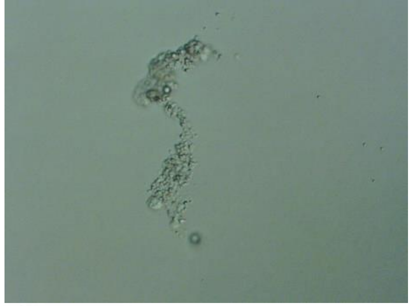

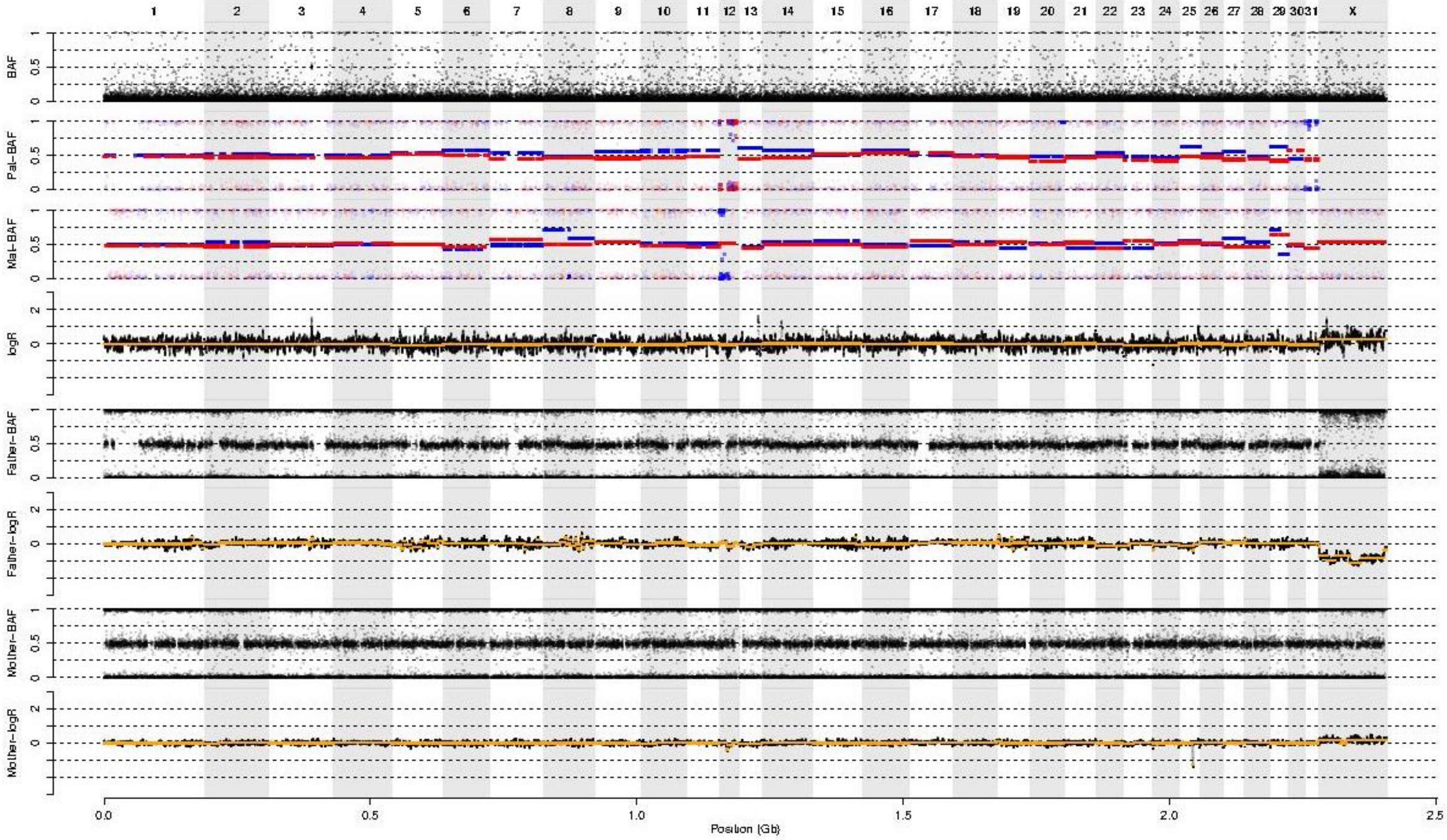

Mare04_Embryo02_Embryo

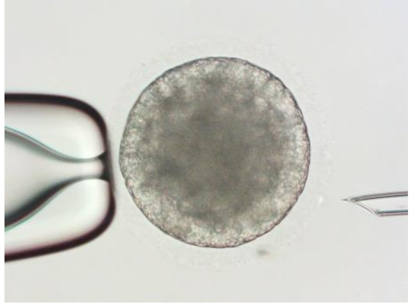

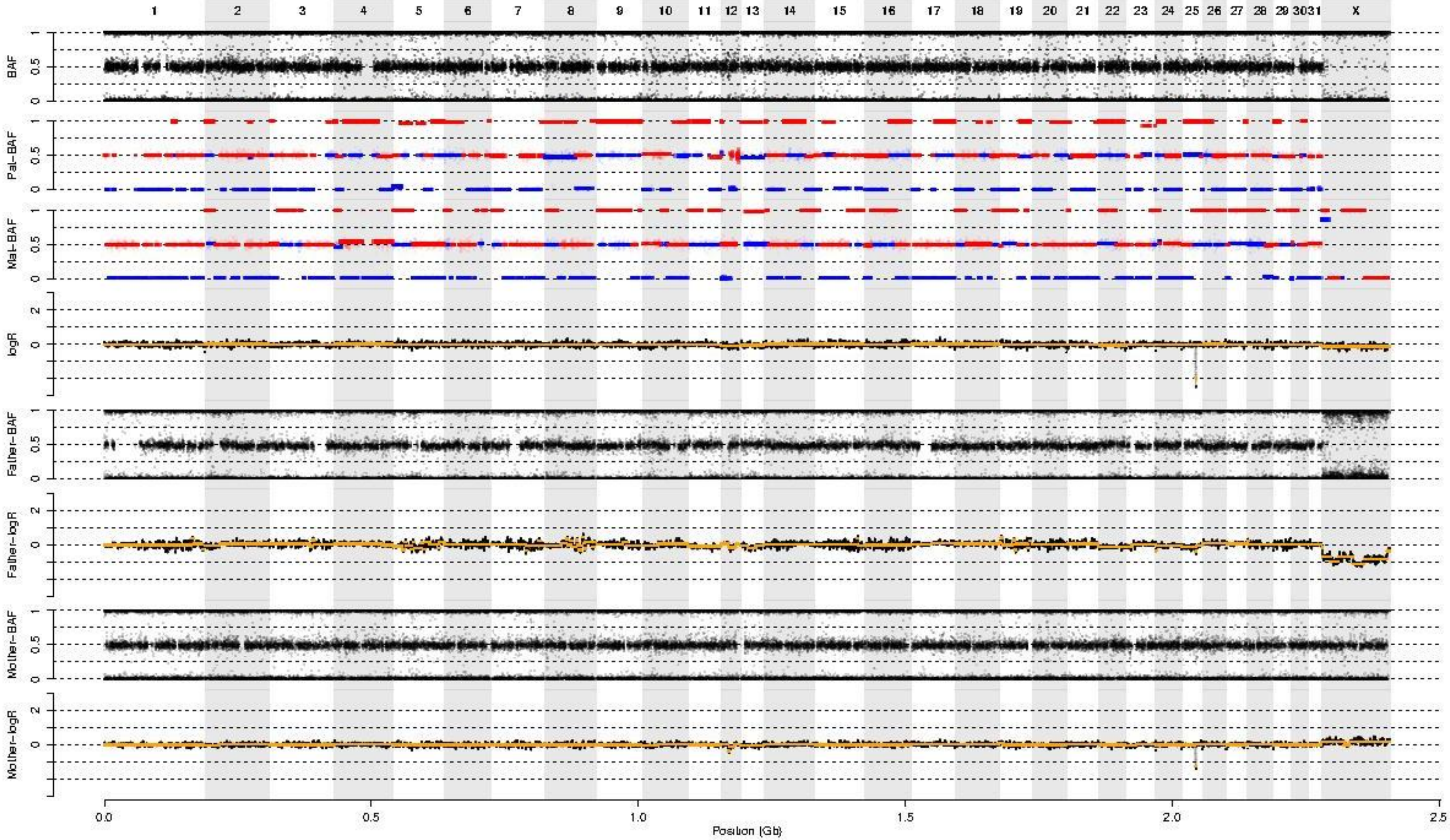

Figure 2. Haplarithm plots of chromosomes containing the five genes of interest of successfully analyzed trophectoderm biopsies and corresponding biopsied blastocyst combinations.

Mare01_Embryo01_Biopsy_Chromosome01

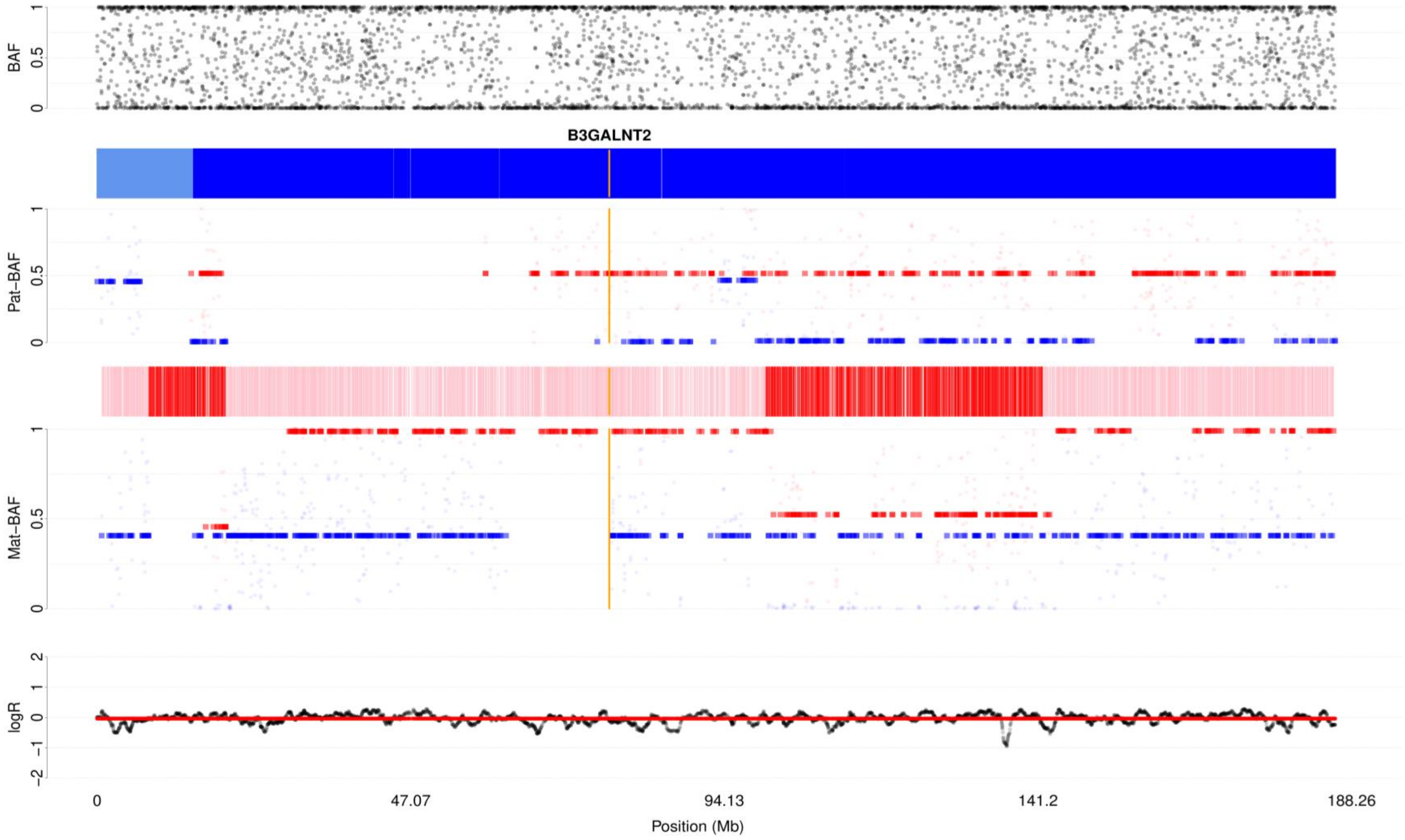

Mare01_Embryo01_Embryo_Chromosome01

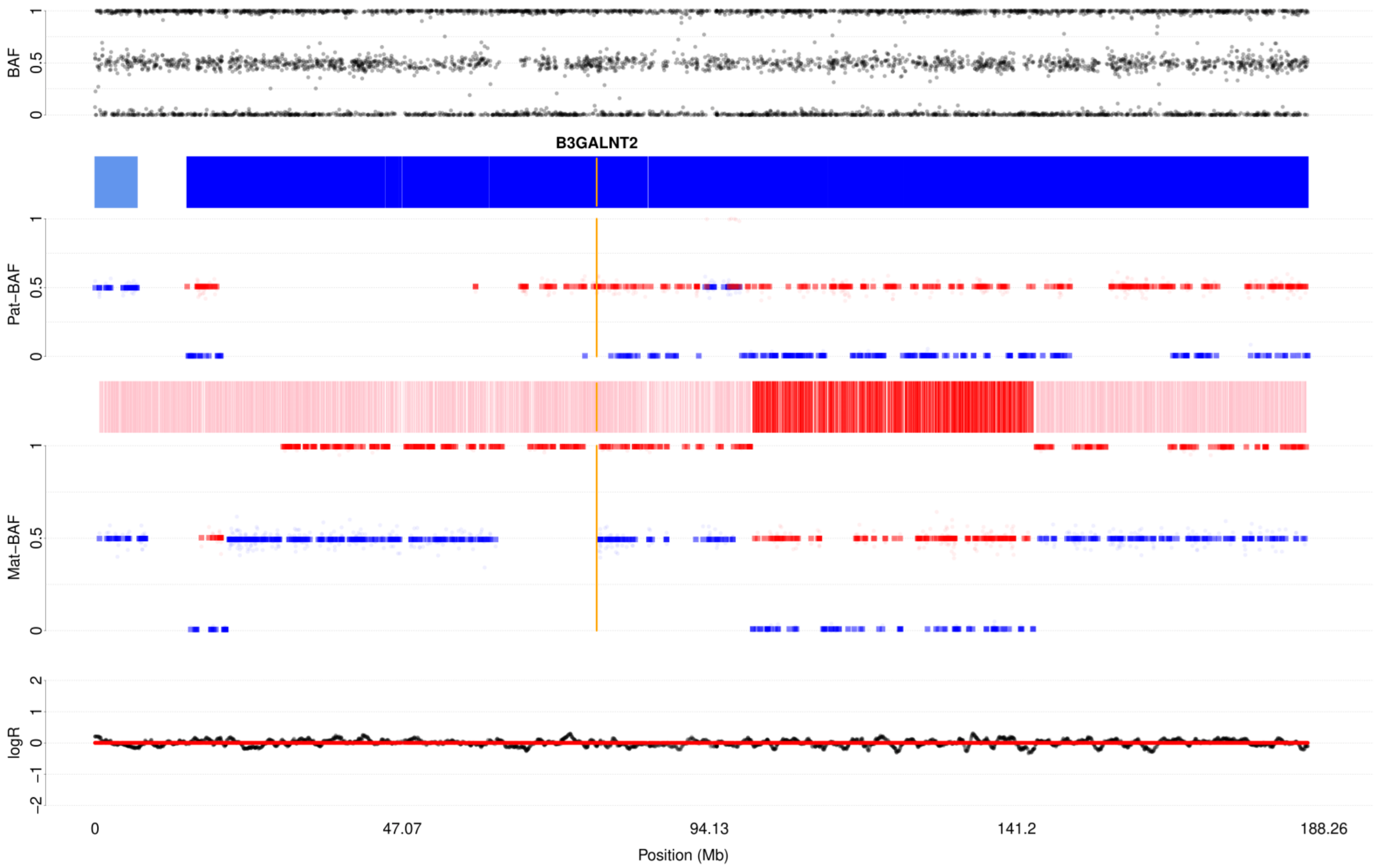

Mare01_Embryo01_Biopsy_Chromosome02

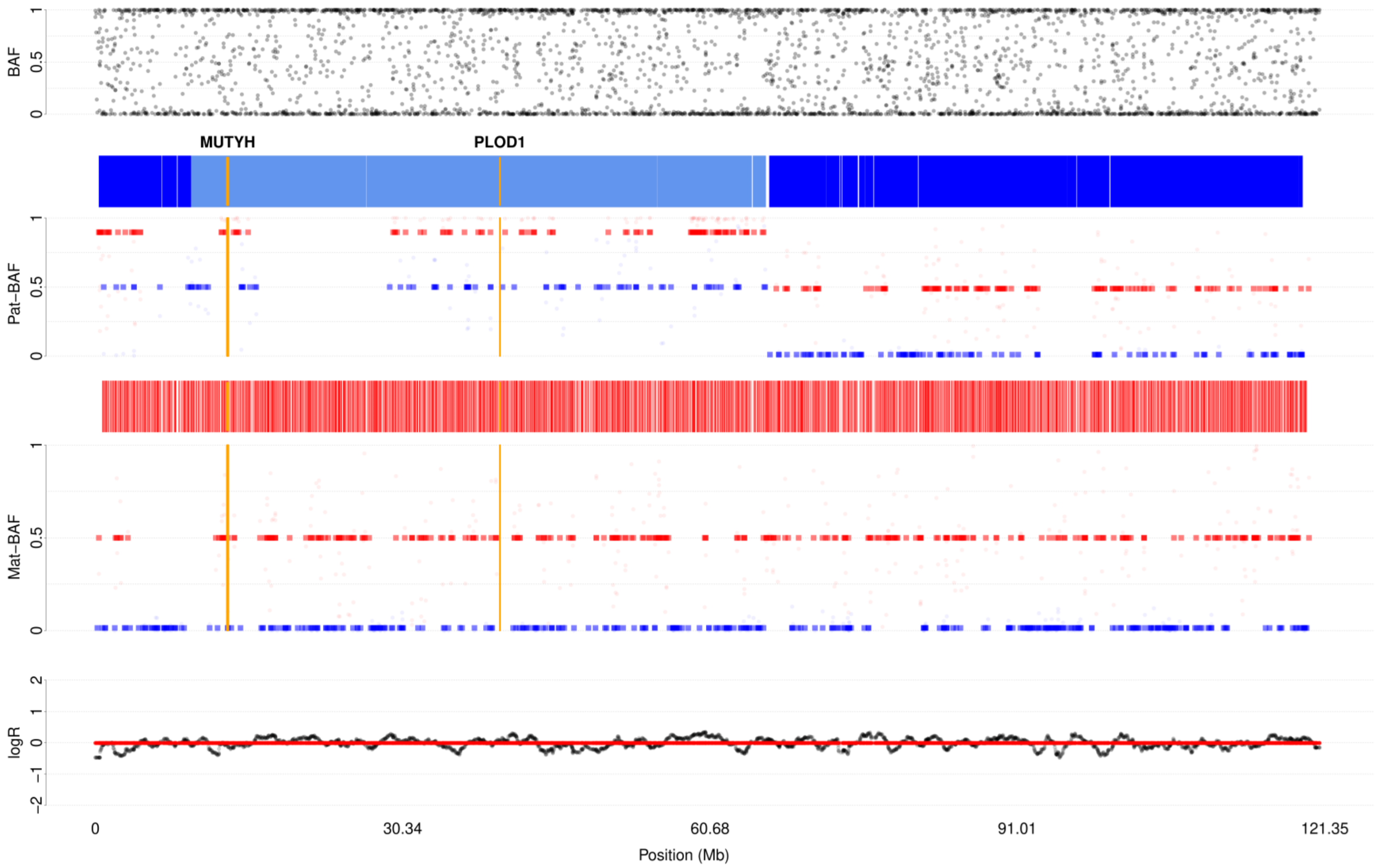

Mare01_Embryo01_Embryo_Chromosome02

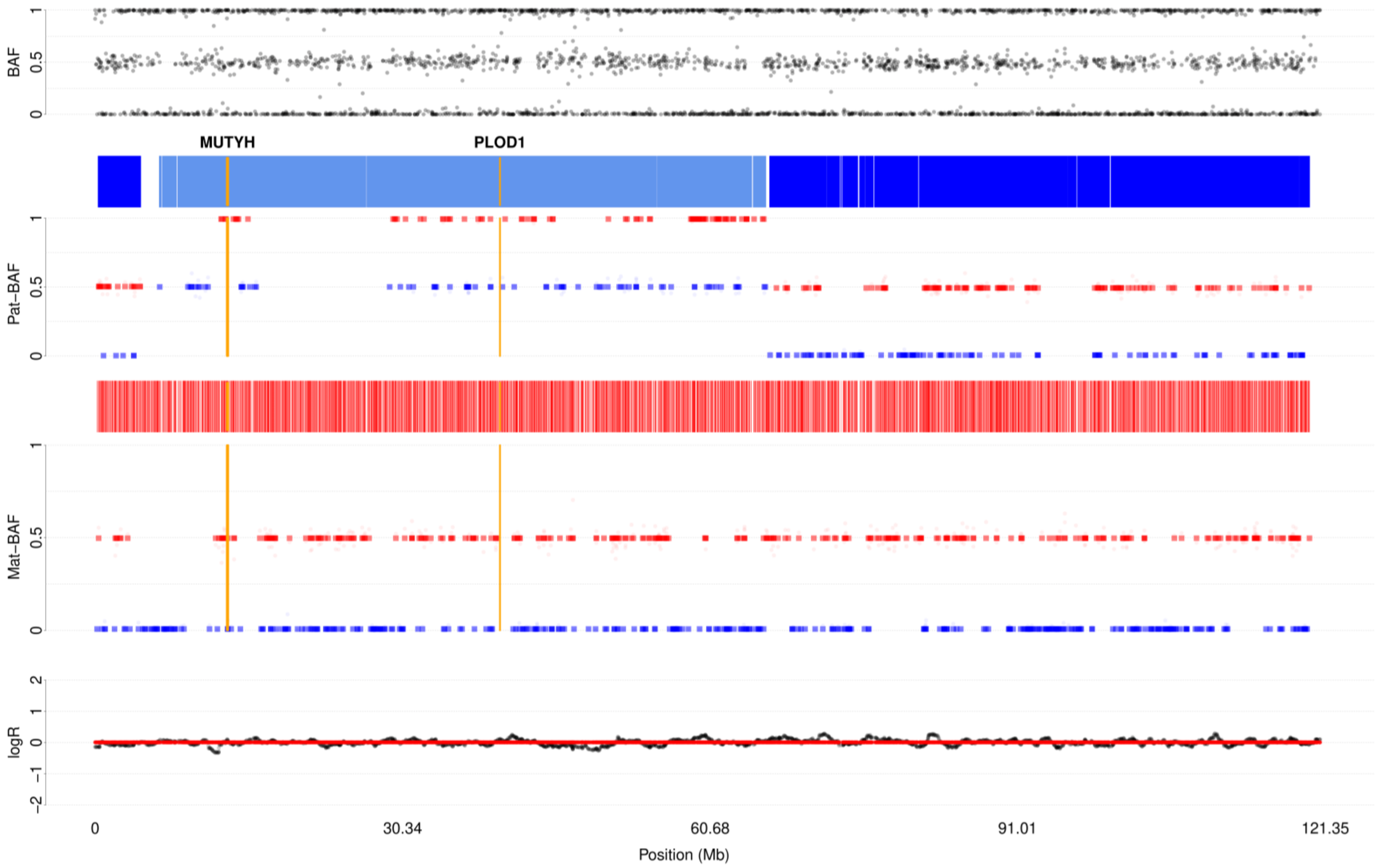

Mare01_Embryo01_Biopsy_Chromosome25

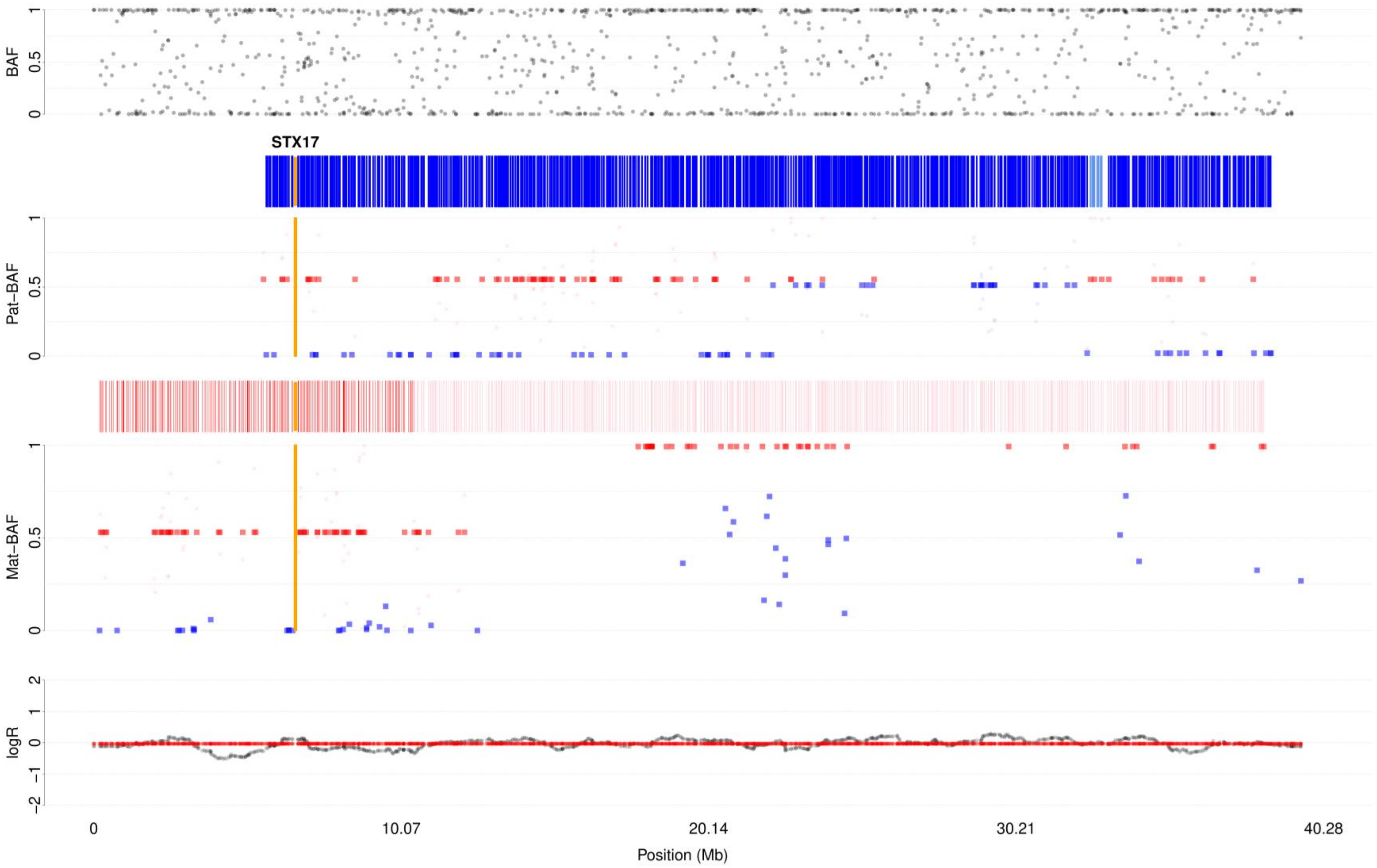

Mare01_Embryo01_Embryo_Chromosome25

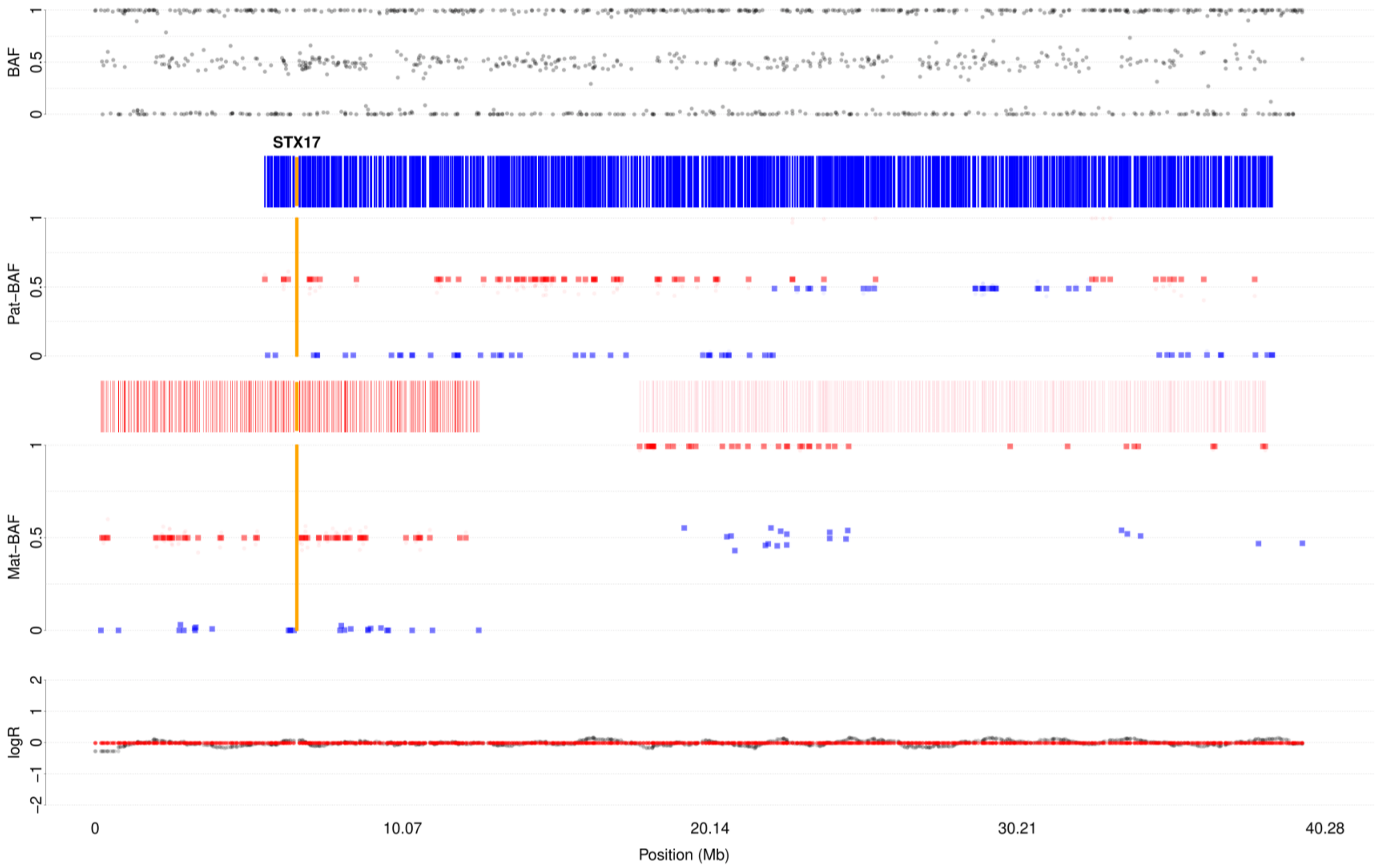

Mare01_Embryo01_Biopsy_Chromosome26

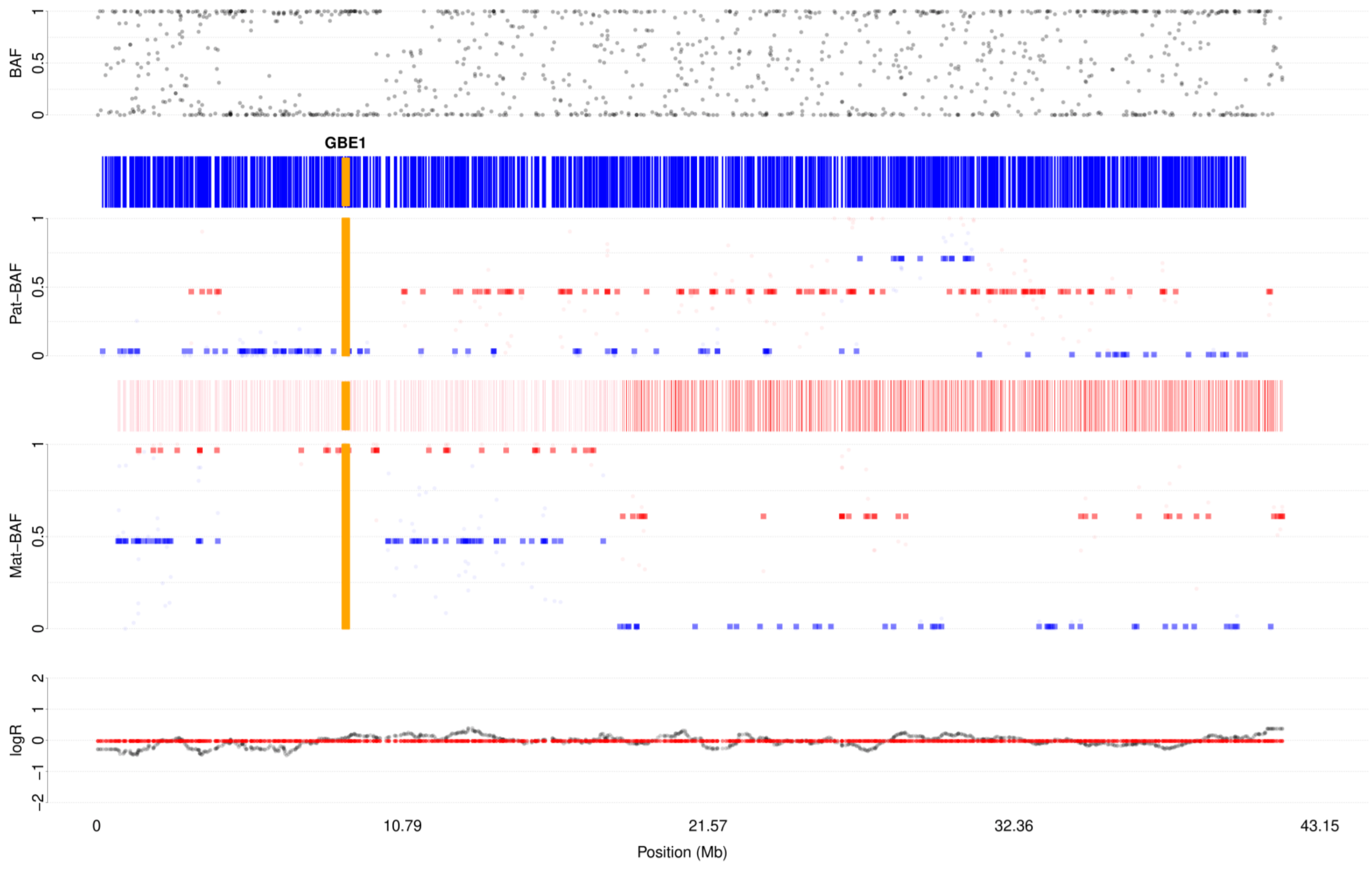

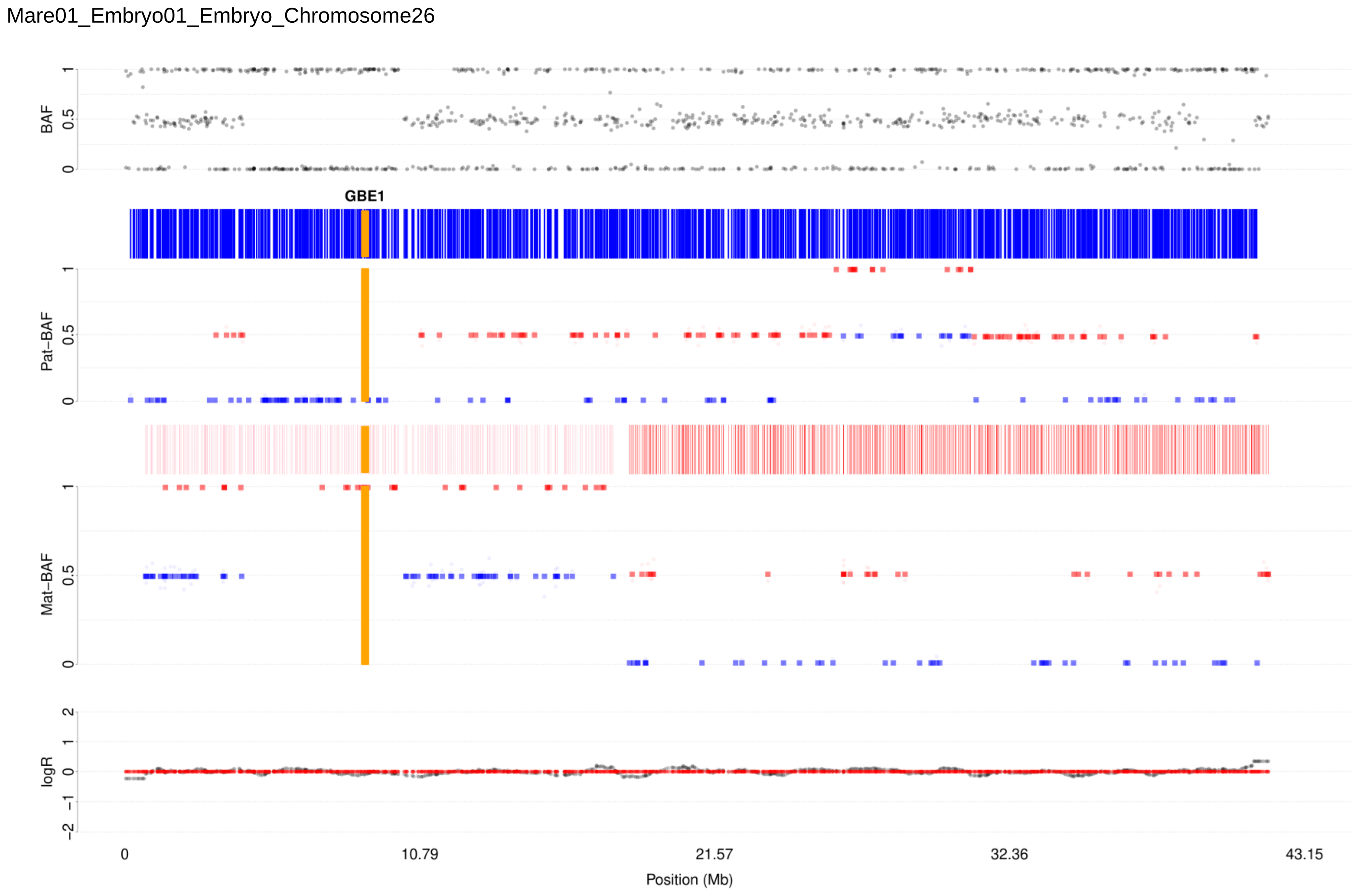
Mare01_Embryo01_Embryo_Chromosome26
BAF
0
0.5
1
GBE1
Pat−BAF
Mat−BAF
logR
−2
−1
2
0
10.79
21.57
32.36
43.15
Position (Mb)

Mare01_Embryo02_Biopsy_Chromosome01

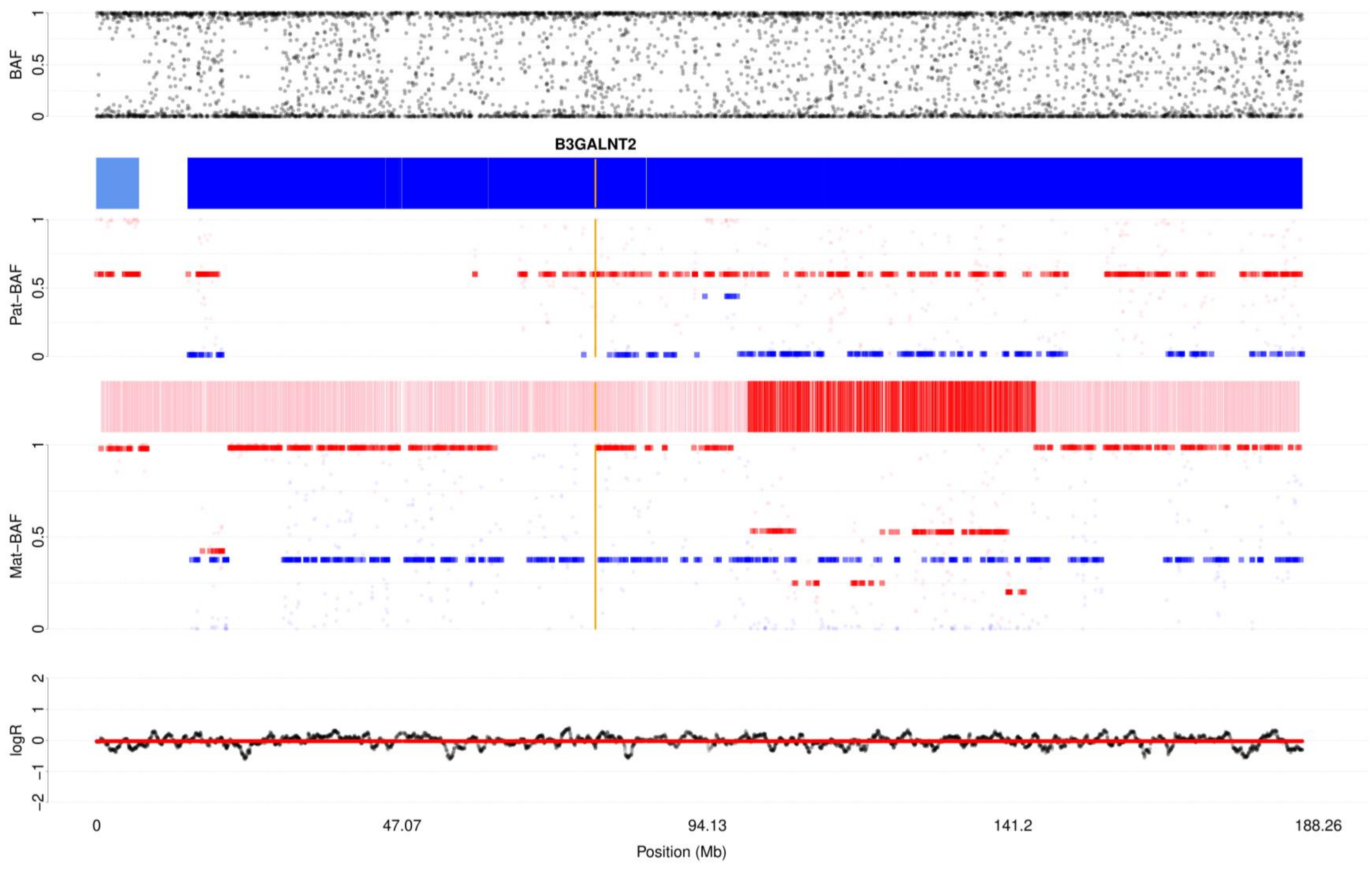

Mare01_Embryo02_Embryo_Chromosome01

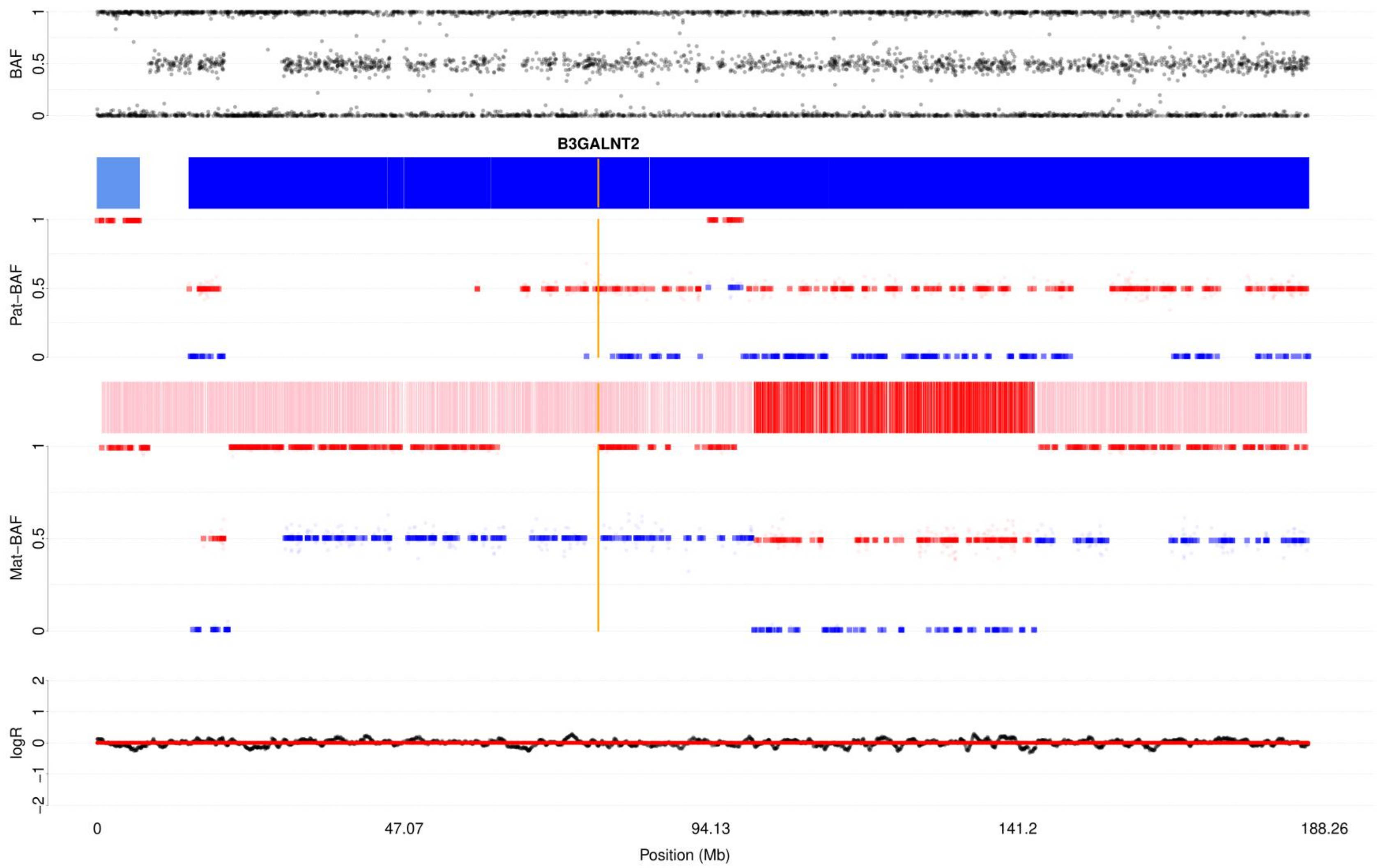

Mare01_Embryo02_Biopsy_Chromosome02

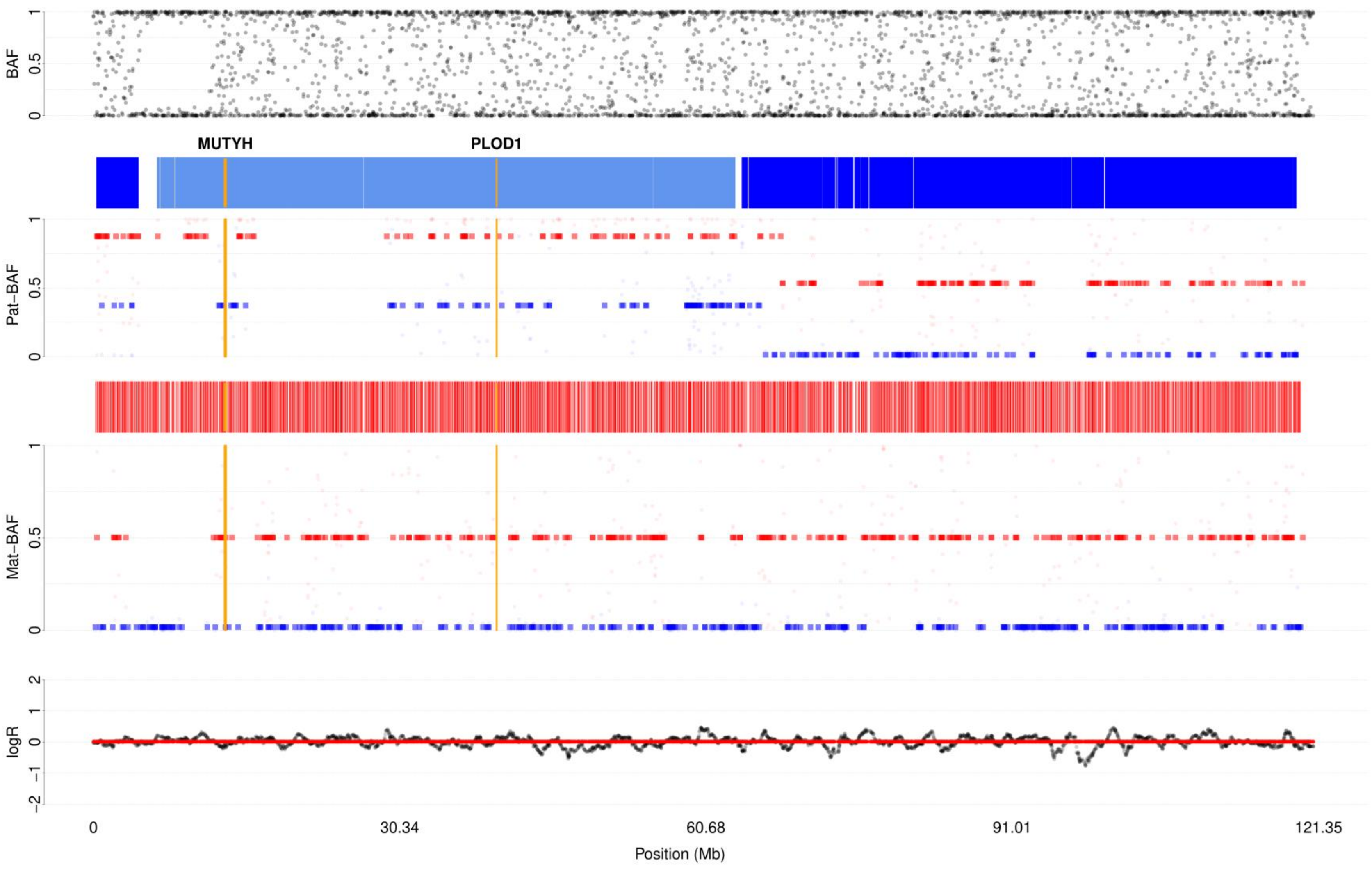

Mare01_Embryo02_Embryo_Chromosome02

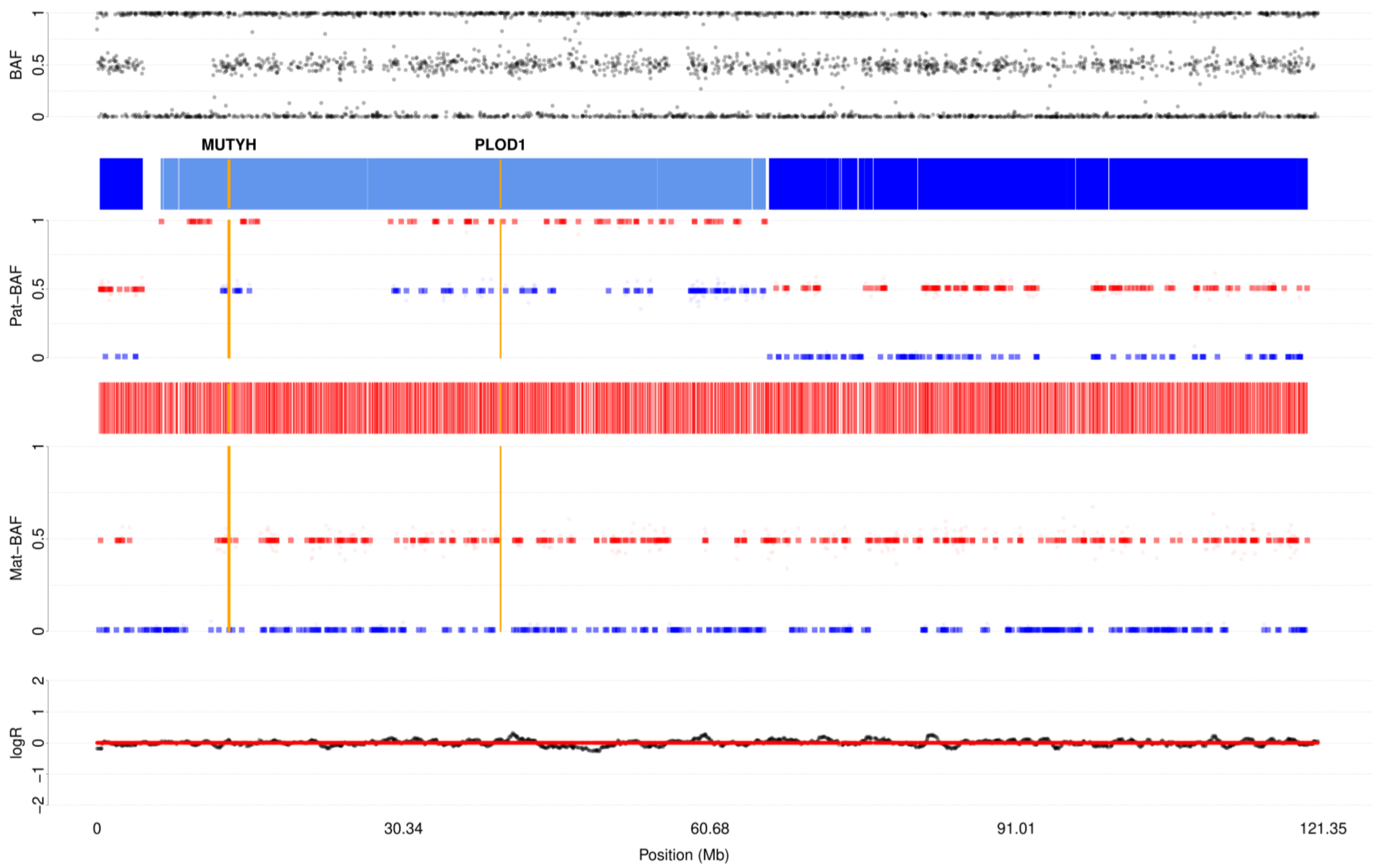

Mare01_Embryo02_Biopsy_Chromosome25

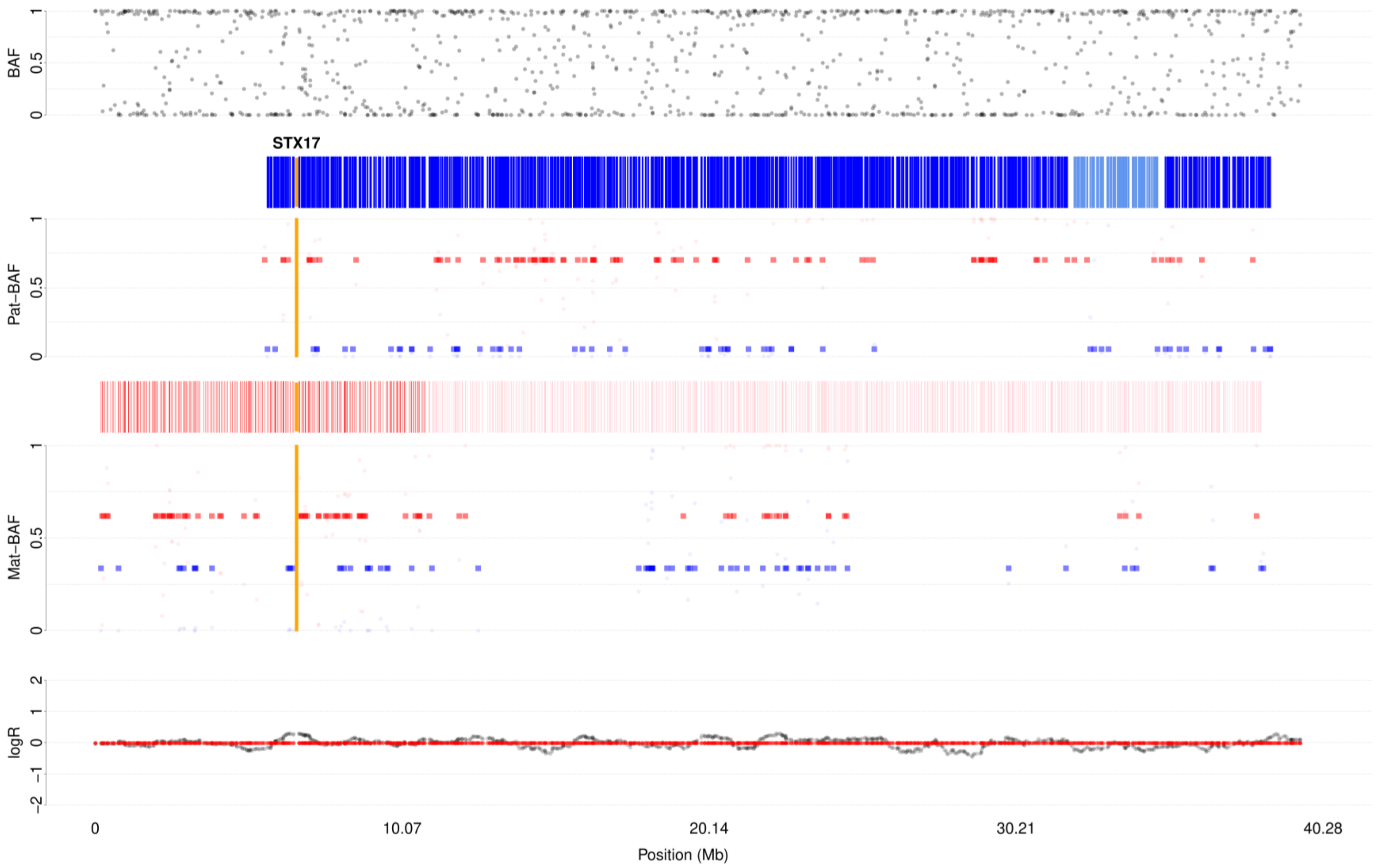

Mare01_Embryo02_Embryo_Chromosome25

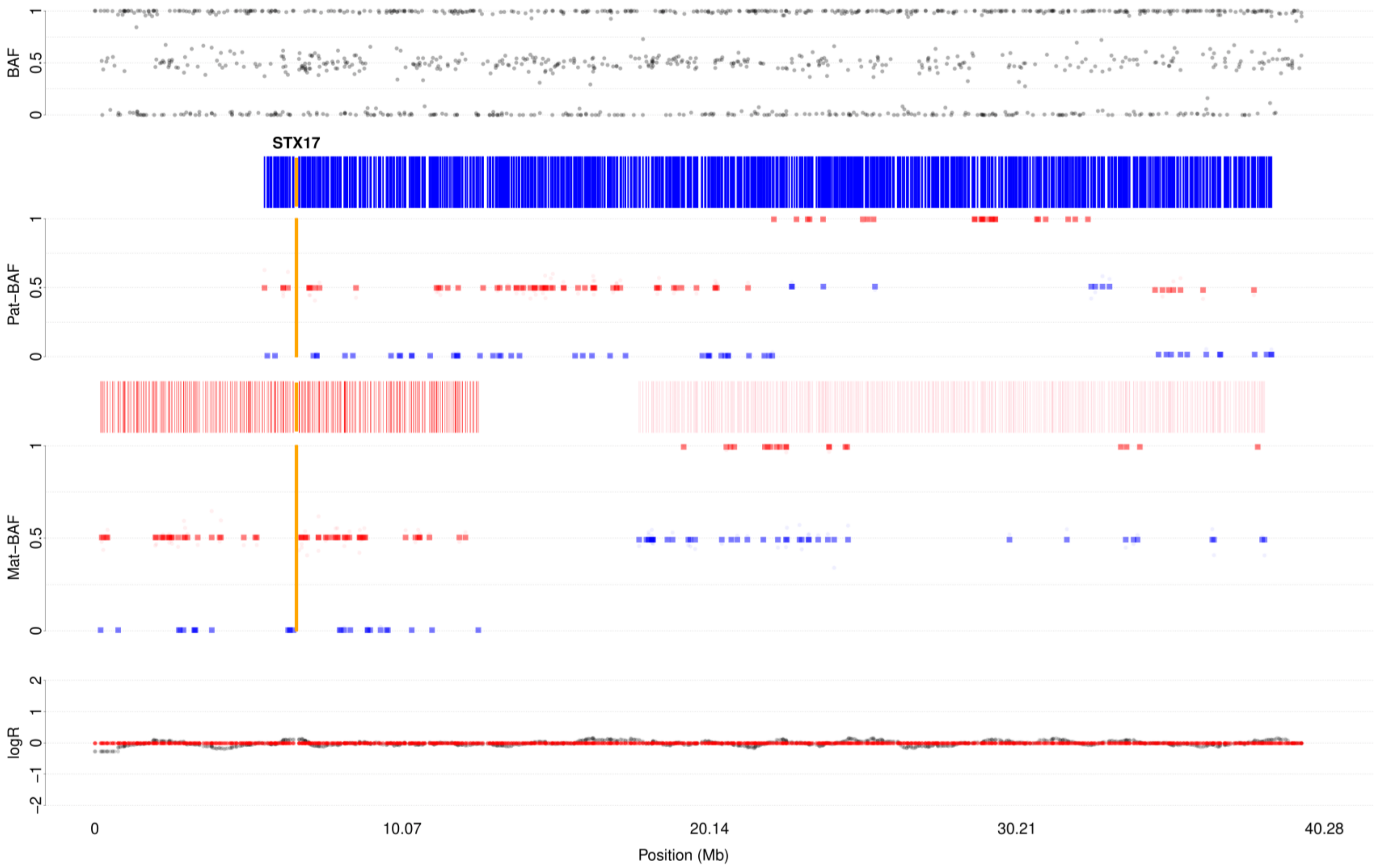

Mare01_Embryo02_Biopsy_Chromosome26

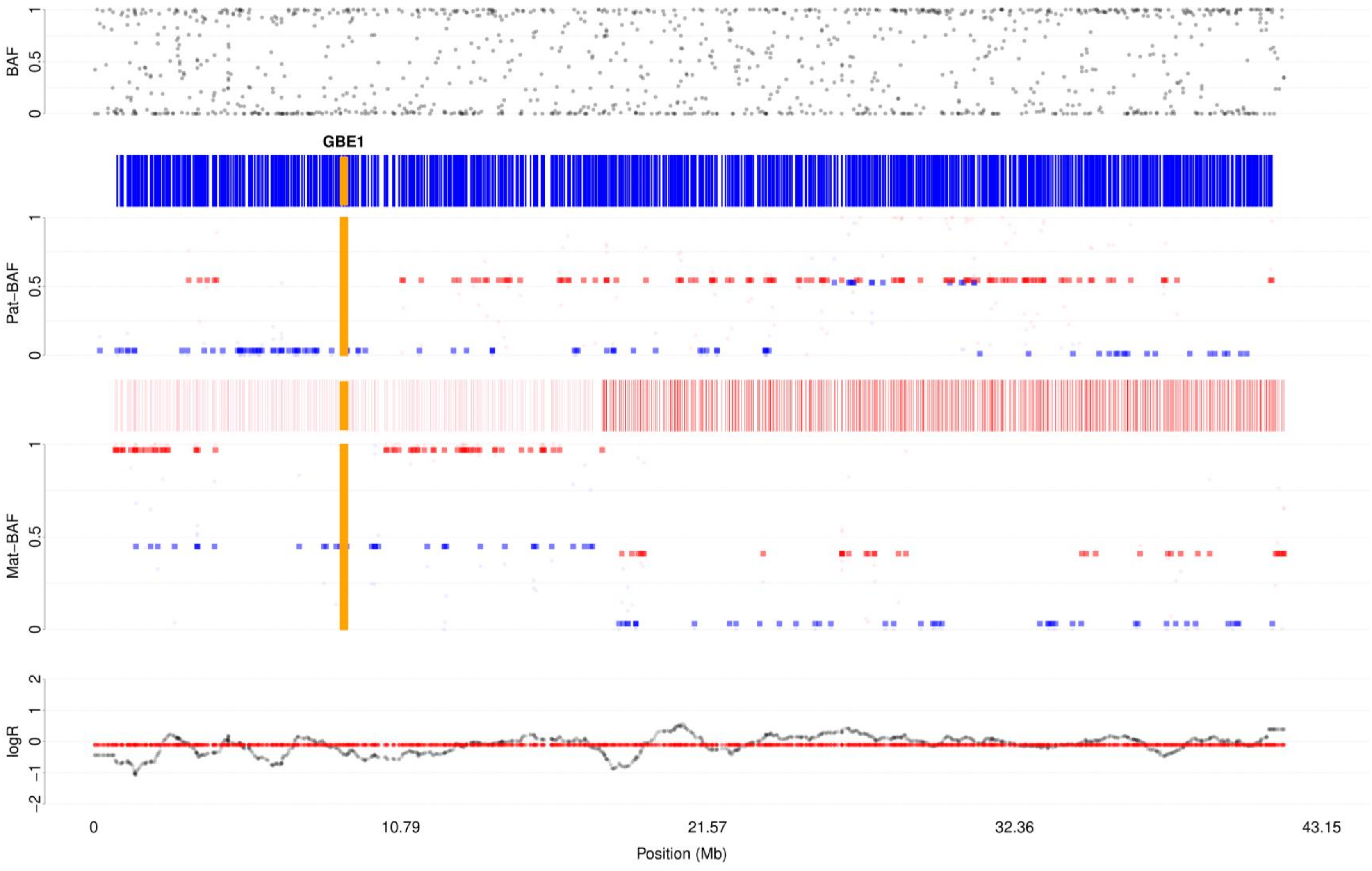

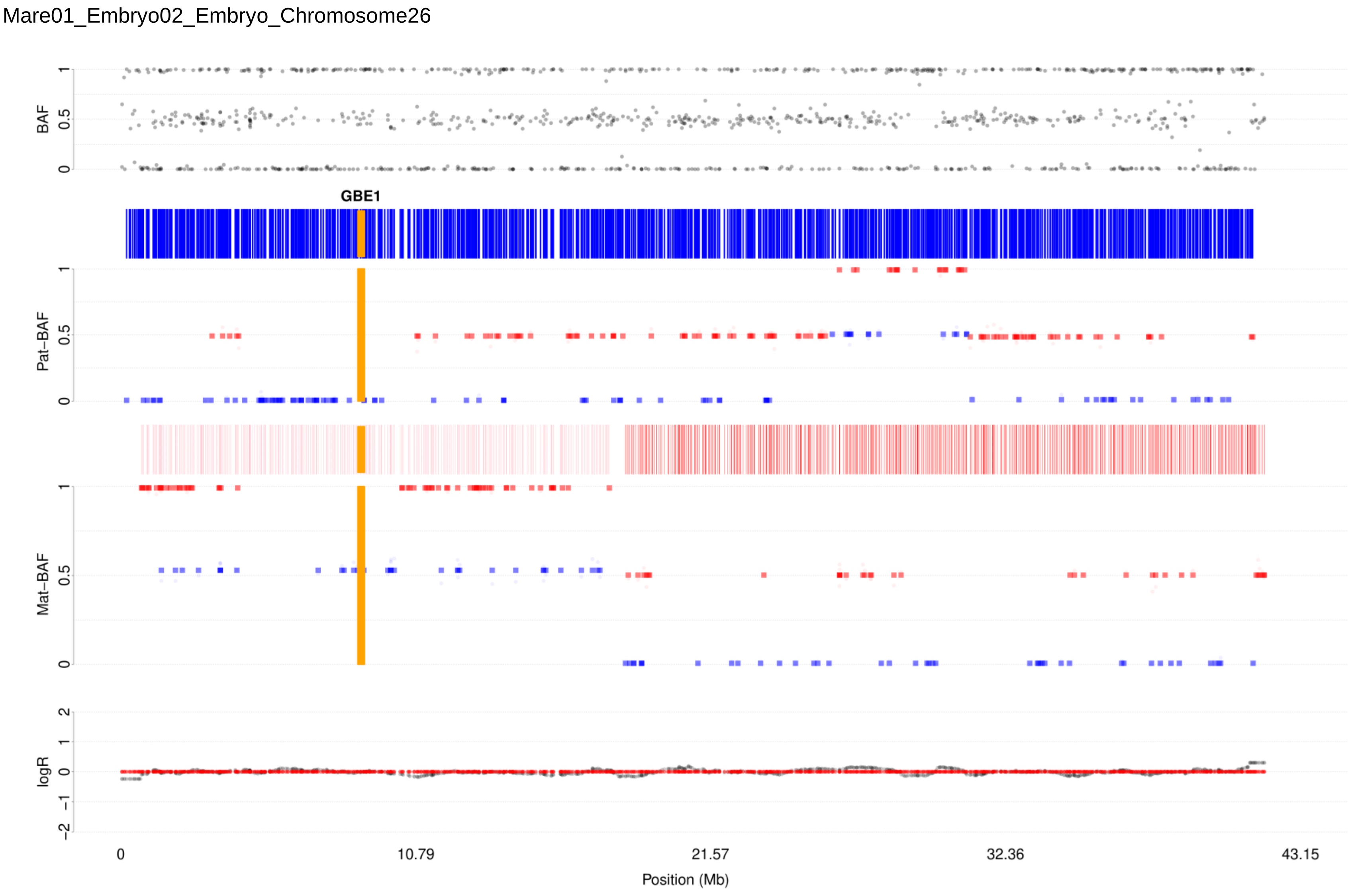
Mare01_Embryo02_Embryo_Chromosome26
BAF
Pat–BAF
Mat–BAF
logR
GBE1
0
0.5
1
−2
−1
2
10.79
21.57
32.36
43.15
Position (Mb)

Mare01_Embryo03_Biopsy_Chromosome01

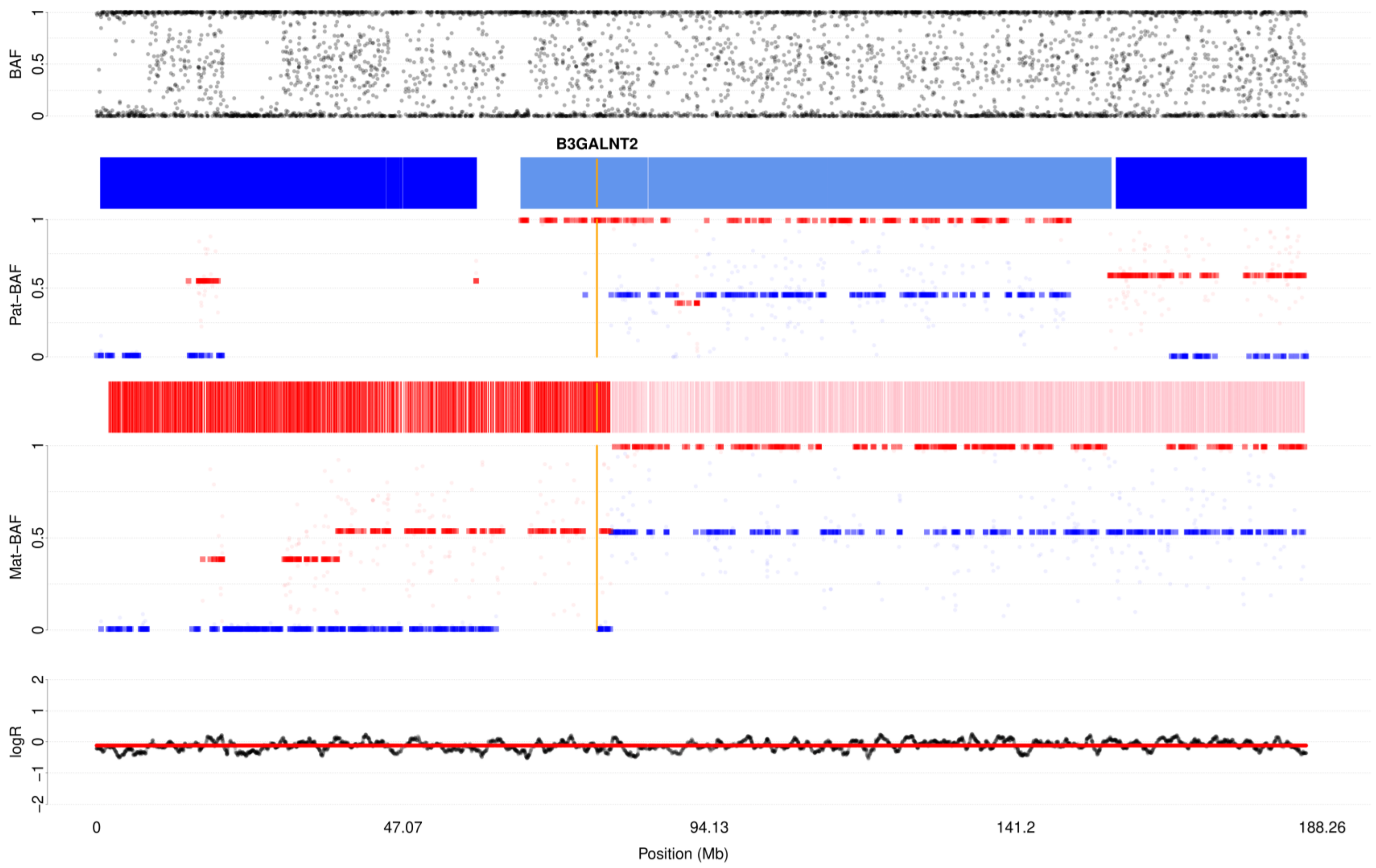

Mare01_Embryo03_Embryo_Chromosome01

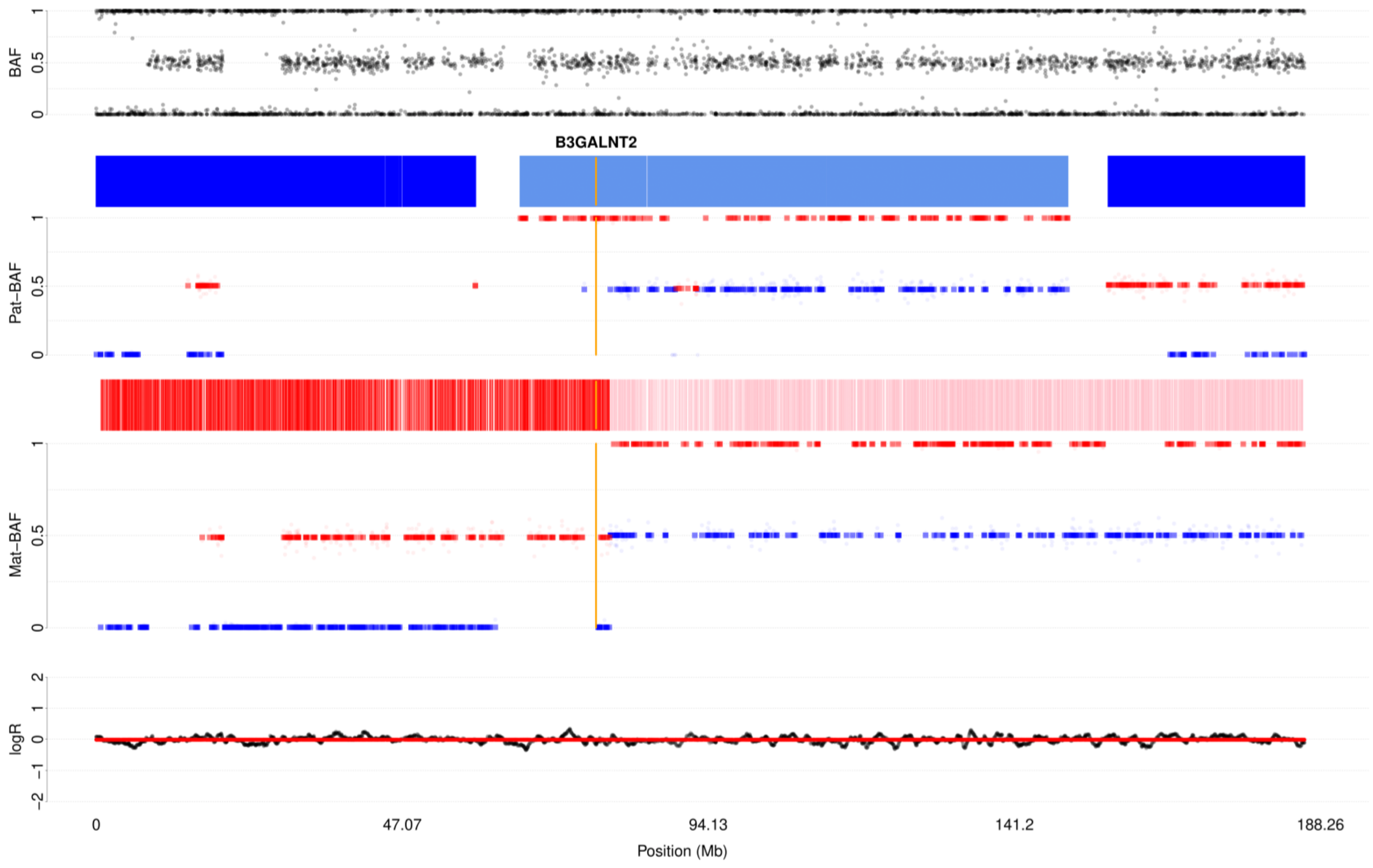

Mare01_Embryo03_Biopsy_Chromosome02

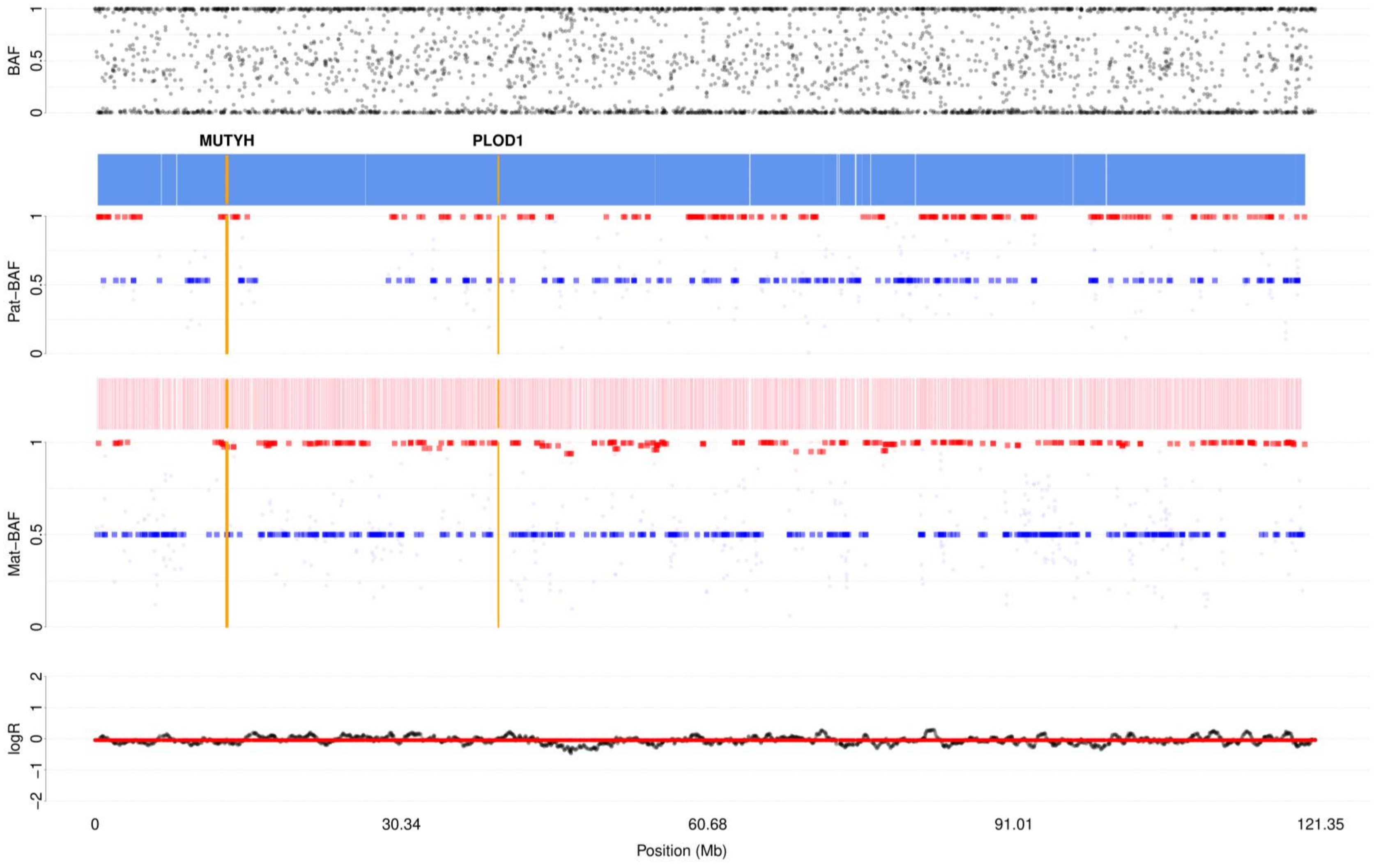

Mare01_Embryo03_Embryo_Chromosome02

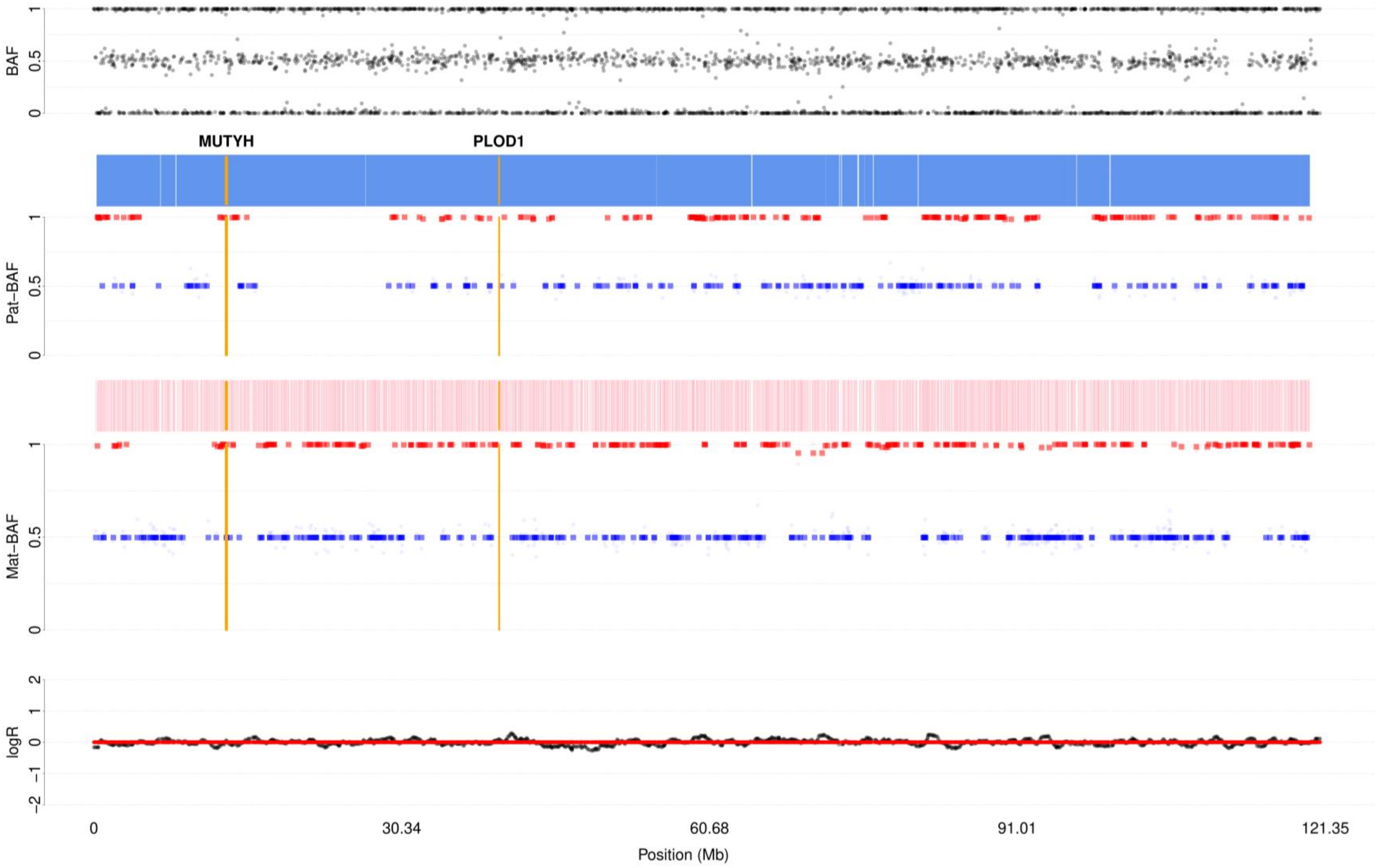

Mare01_Embryo03_Biopsy_Chromosome25

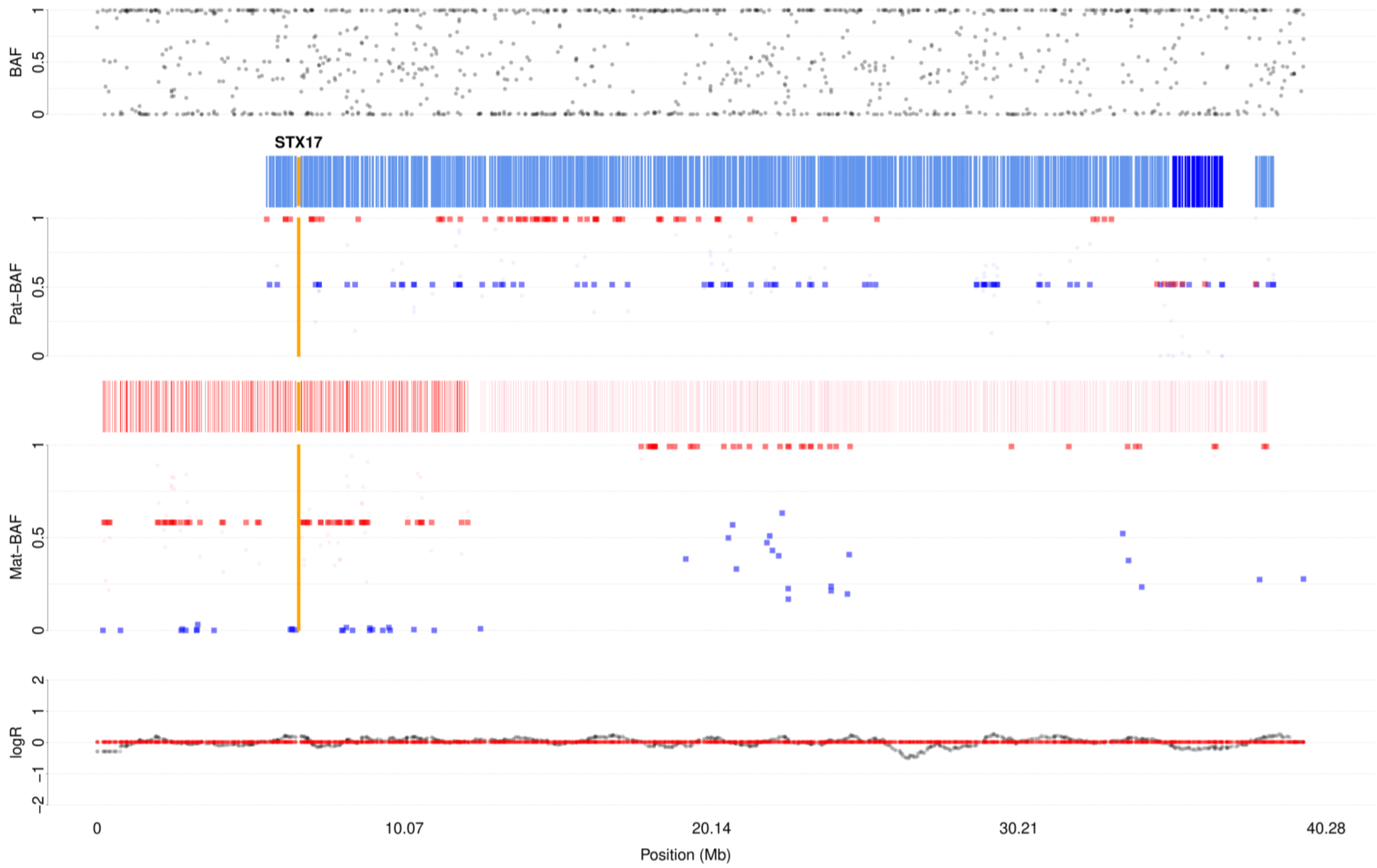

Mare01_Embryo03_Embryo_Chromosome25

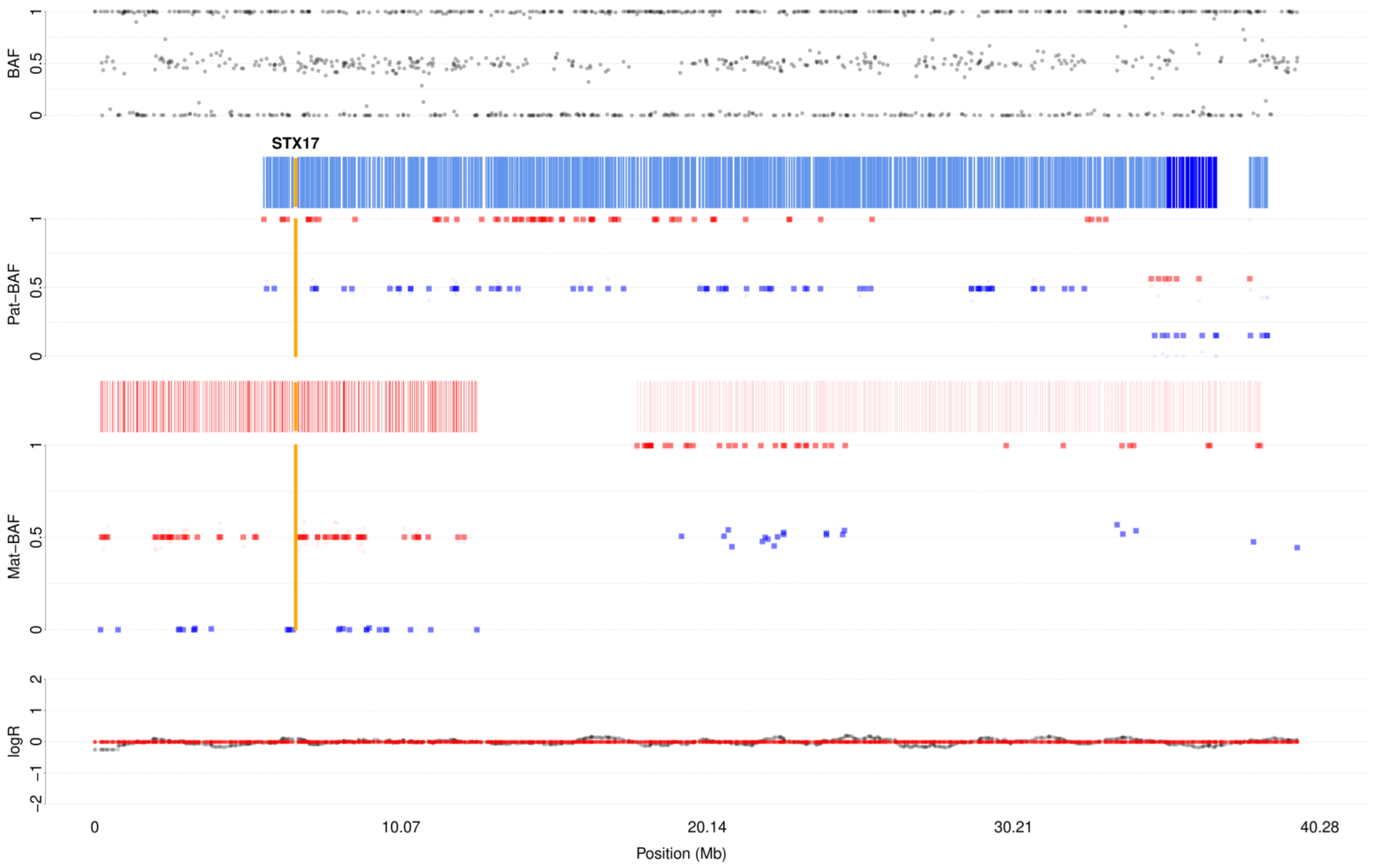

Mare01_Embryo03_Biopsy_Chromosome26

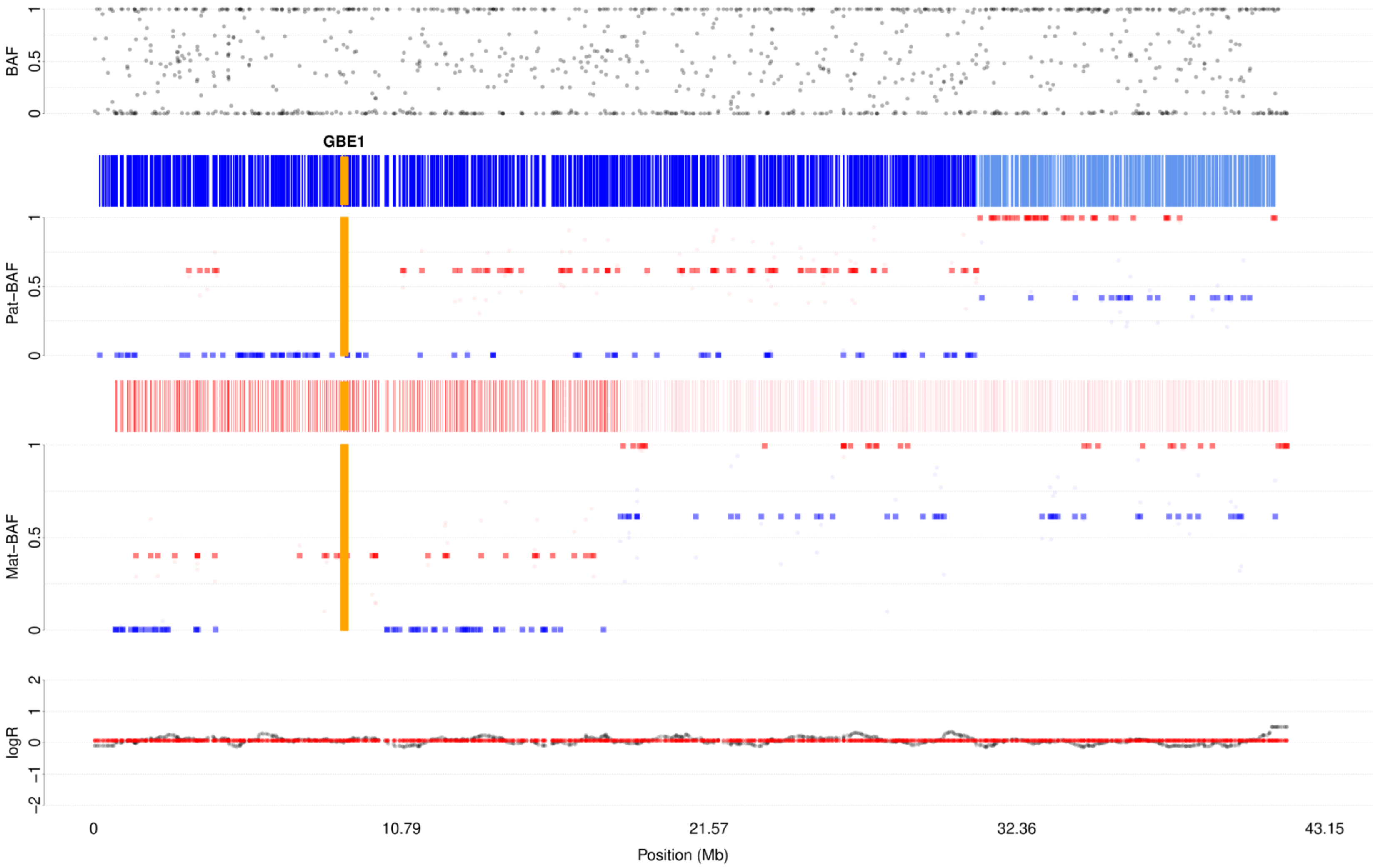

Mare01_Embryo03_Embryo_Chromosome26

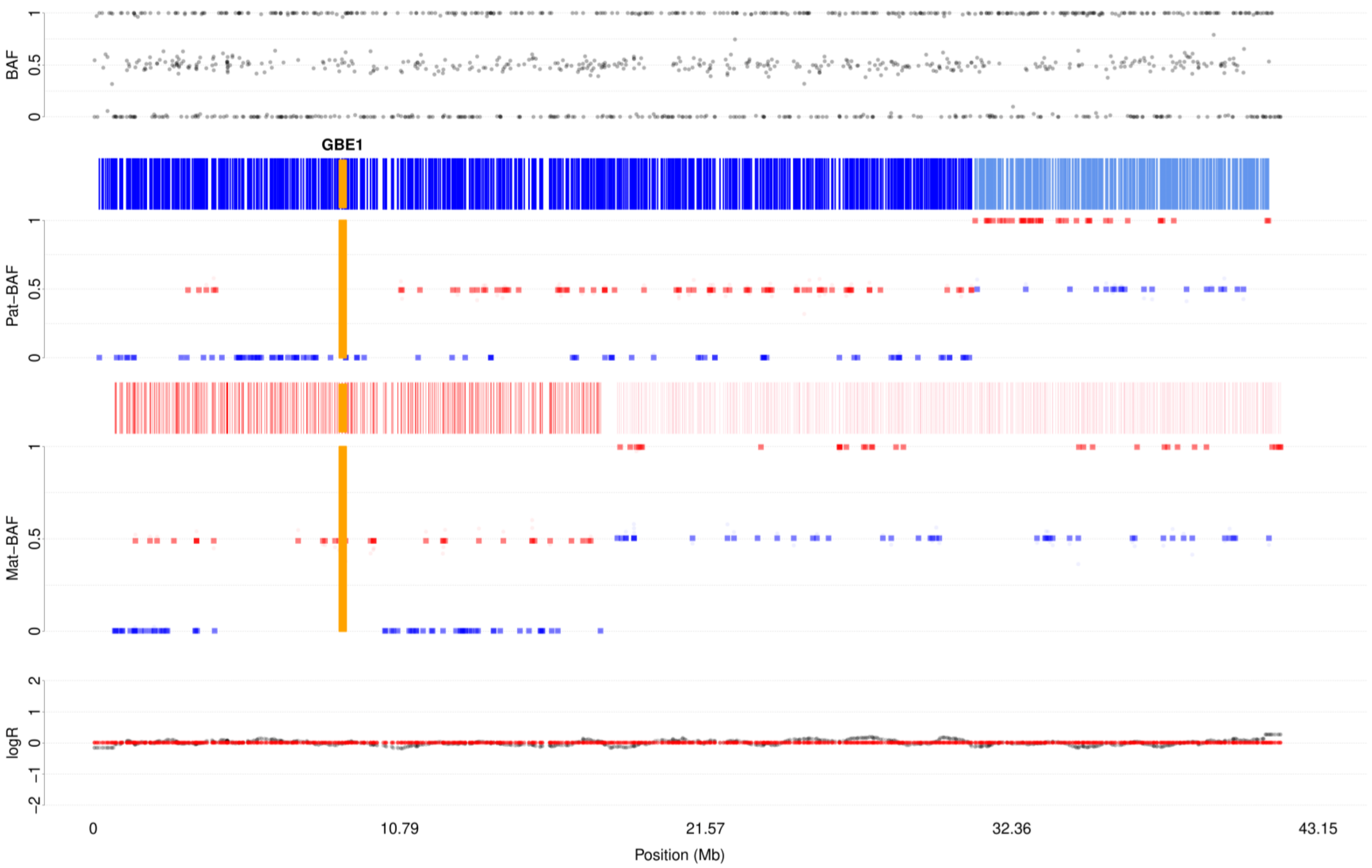

Mare01_Embryo04_Biopsy_Chromosome01

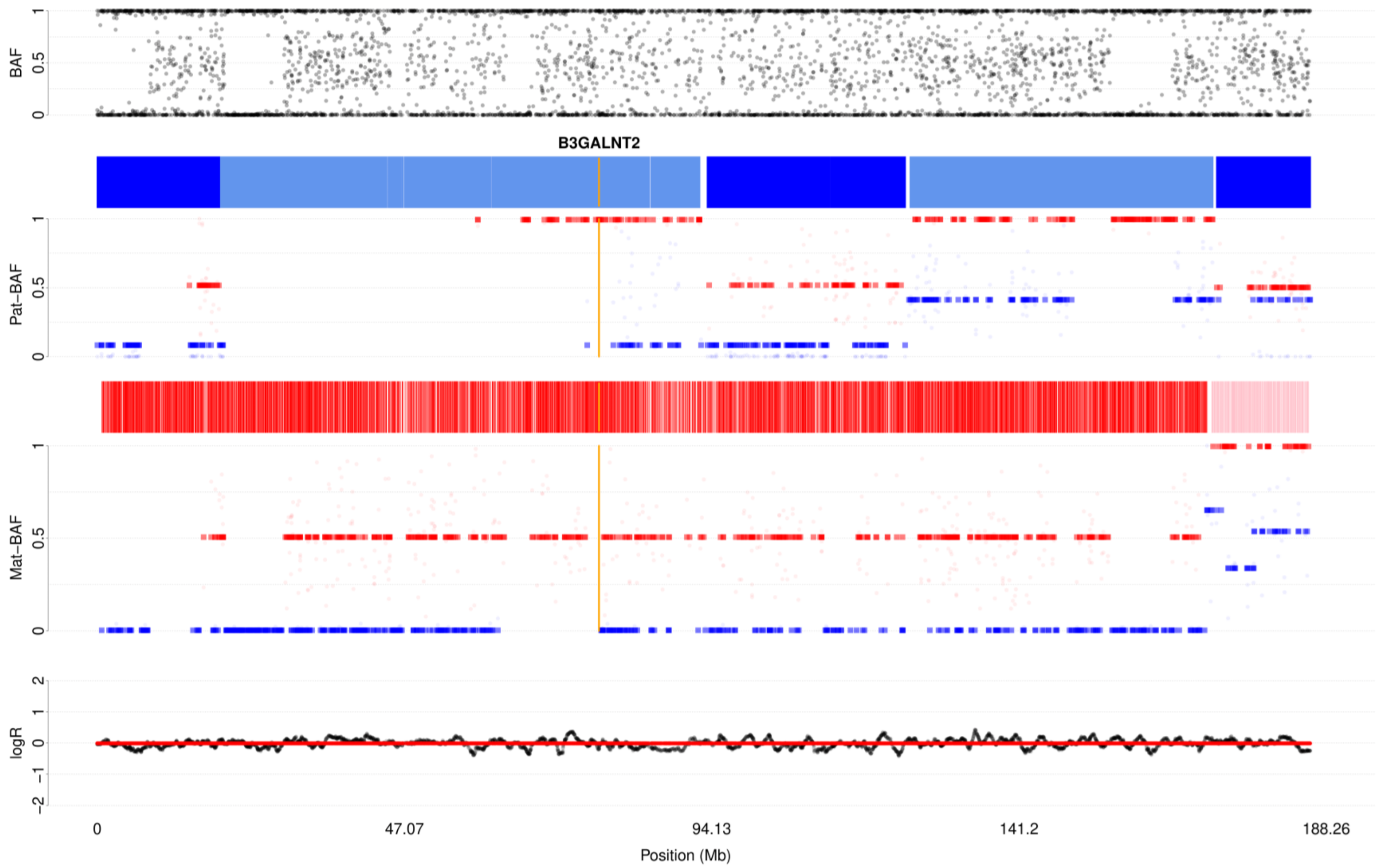

Mare01_Embryo04_Embryo_Chromosome01

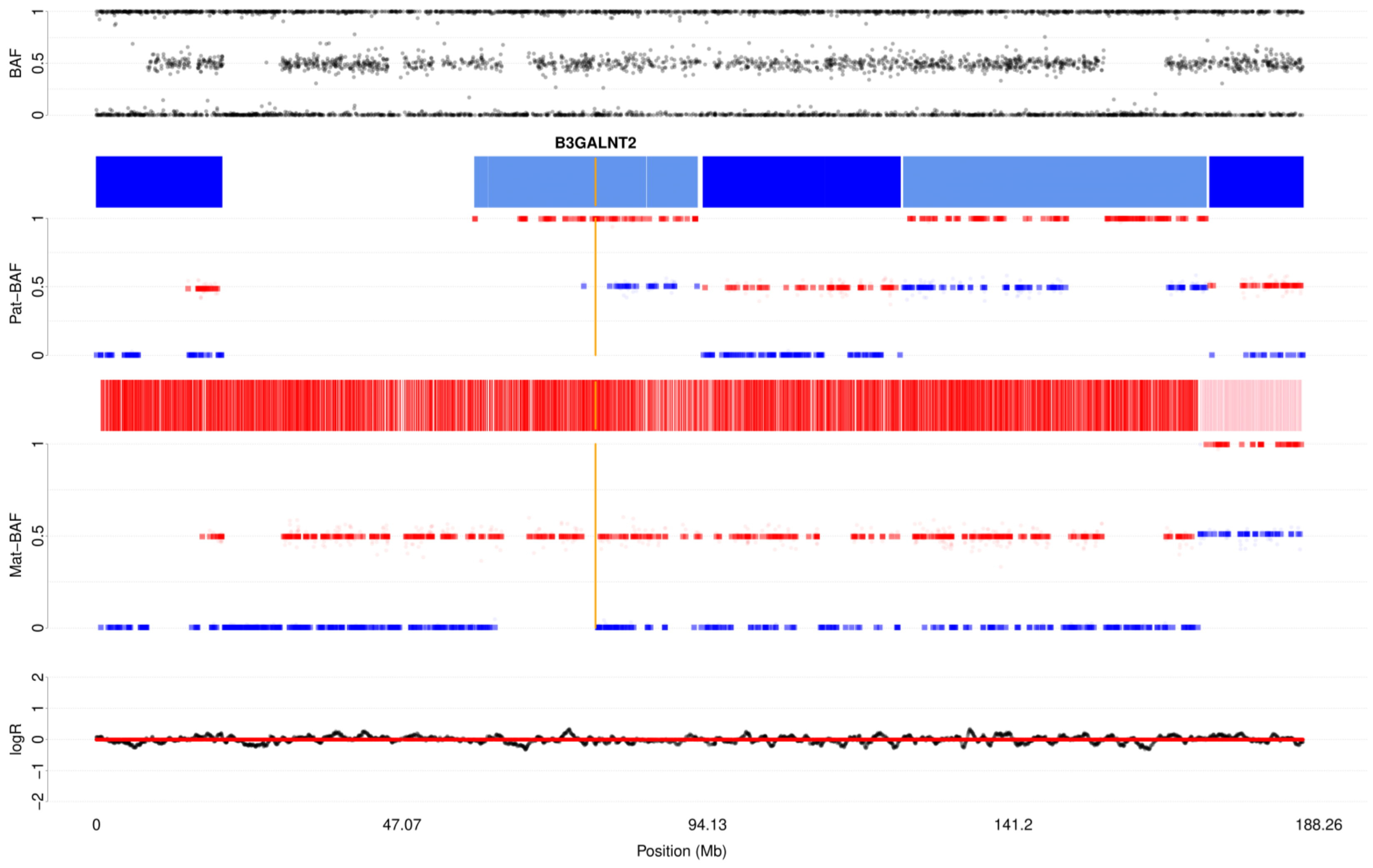

Mare01_Embryo04_Biopsy_Chromosome02

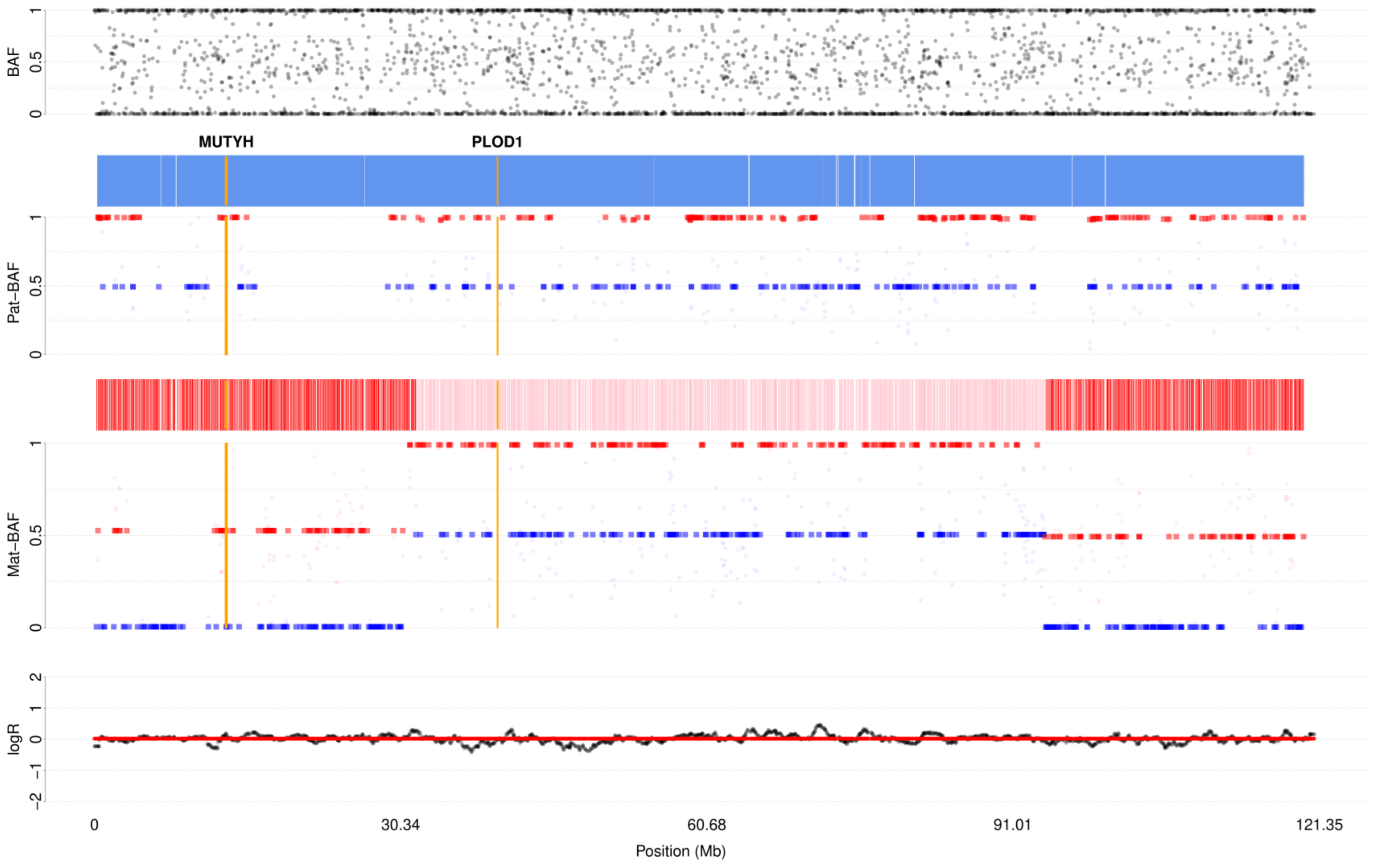

Mare01_Embryo04_Embryo_Chromosome02

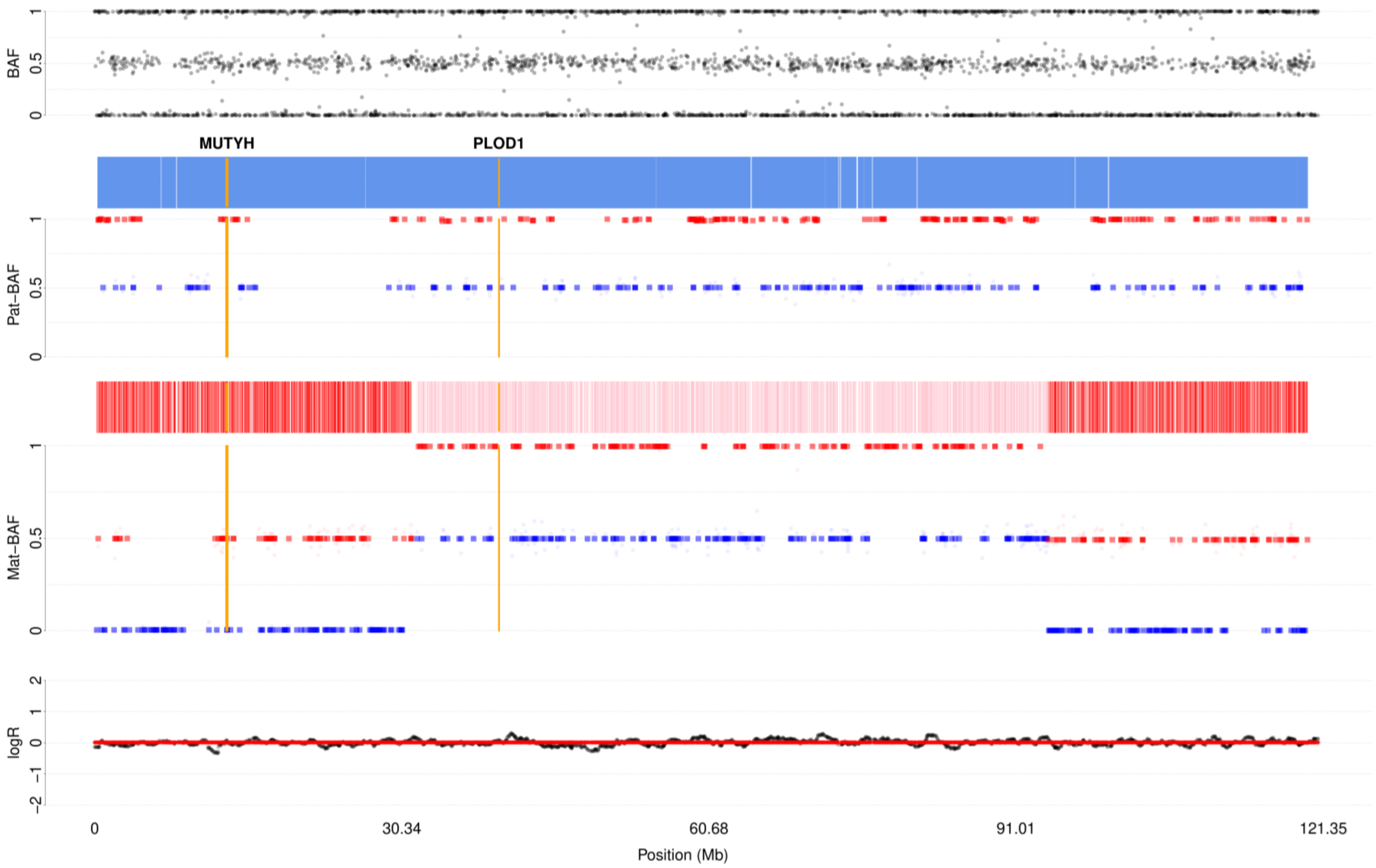

Mare01_Embryo04_Biopsy_Chromosome25

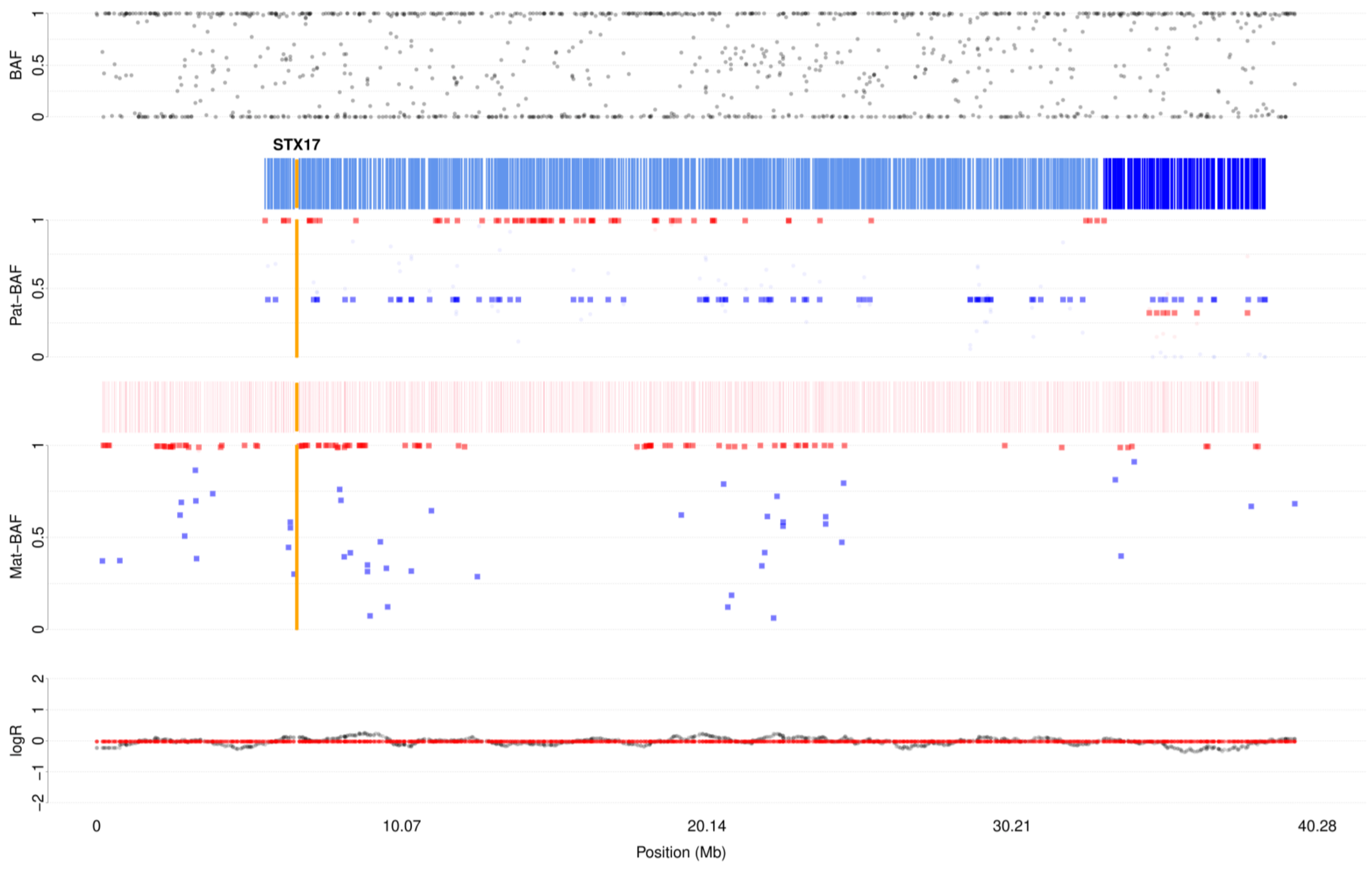

Mare01_Embryo04_Embryo_Chromosome25

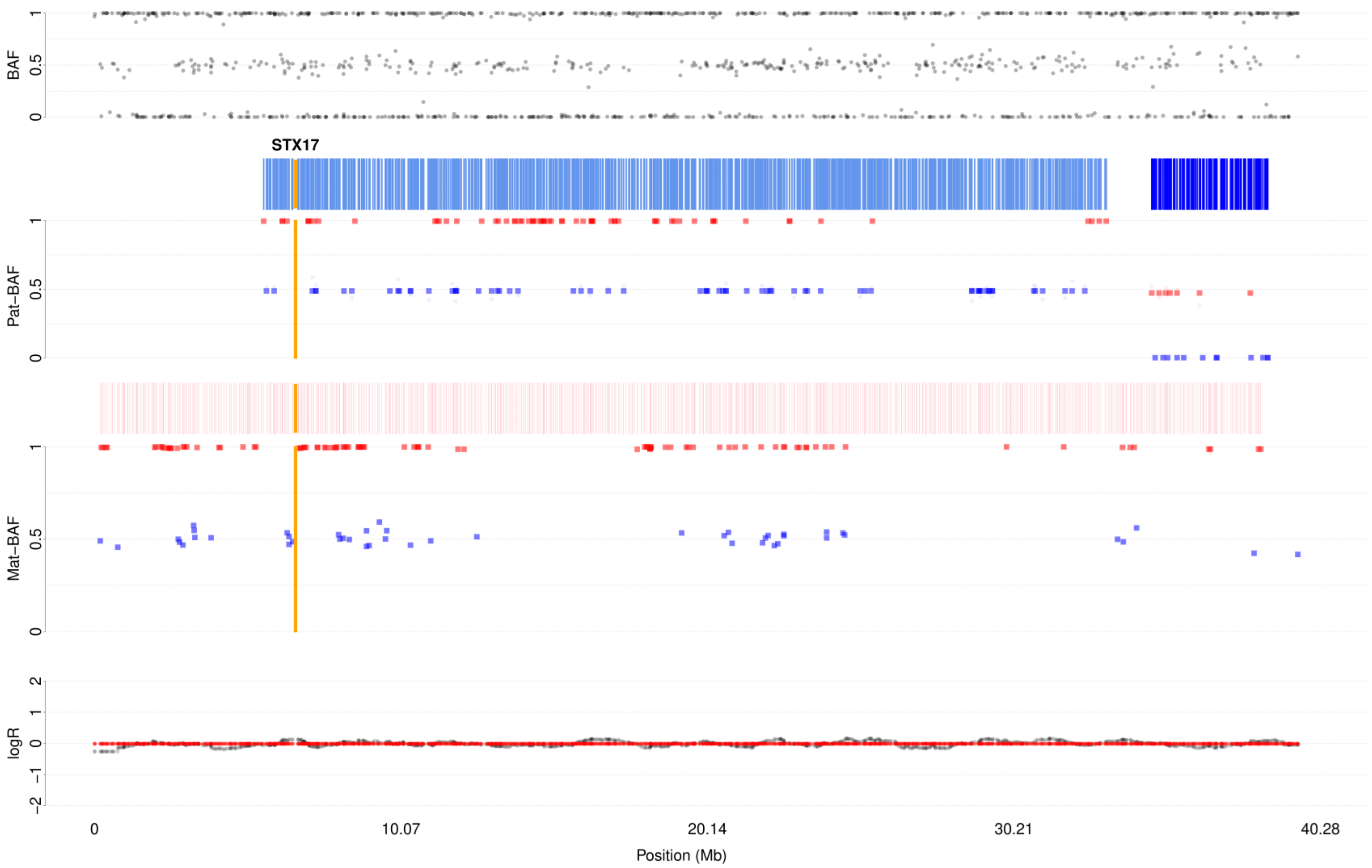

Mare01_Embryo04_Biopsy_Chromosome26

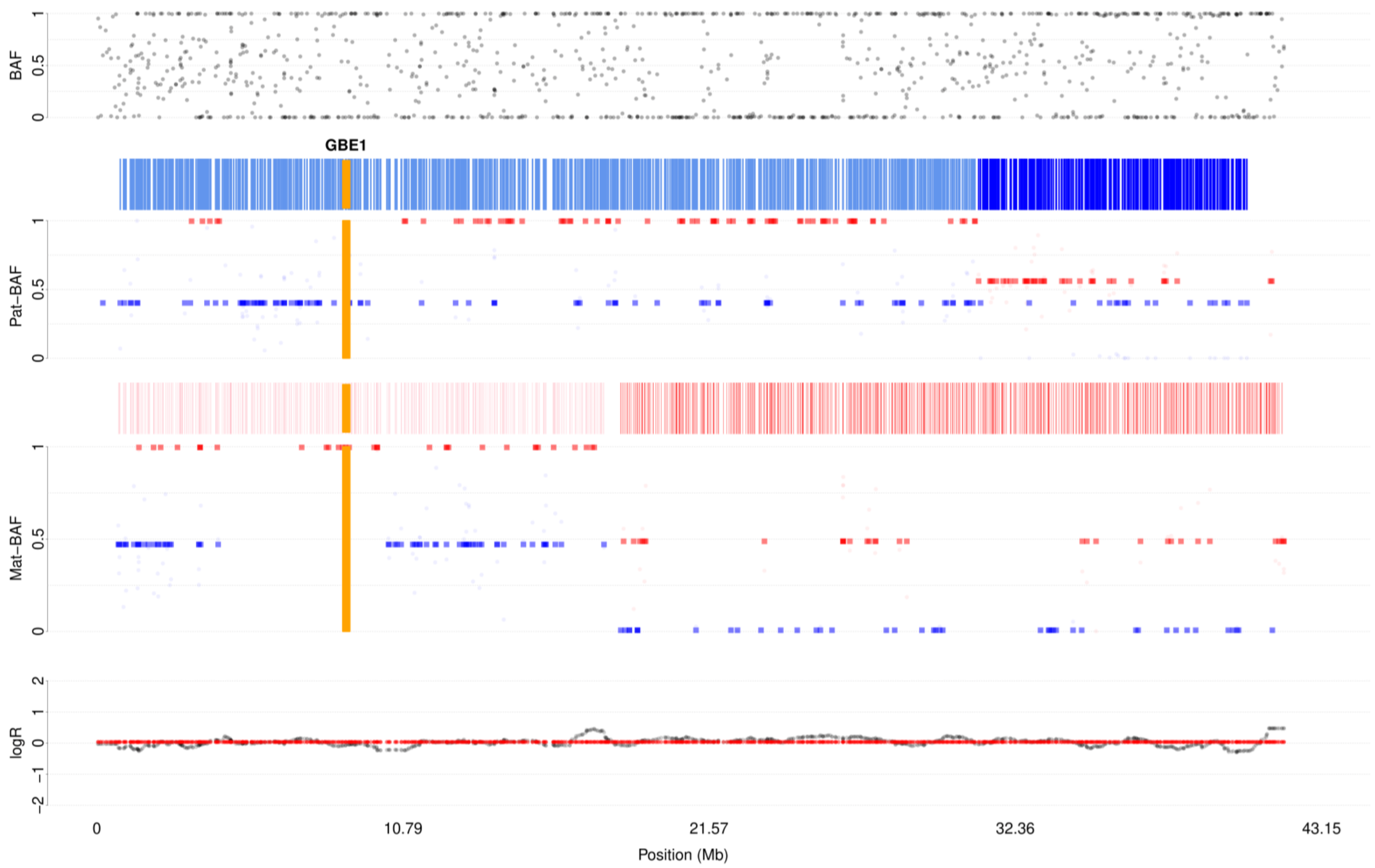

Mare01_Embryo04_Embryo_Chromosome26

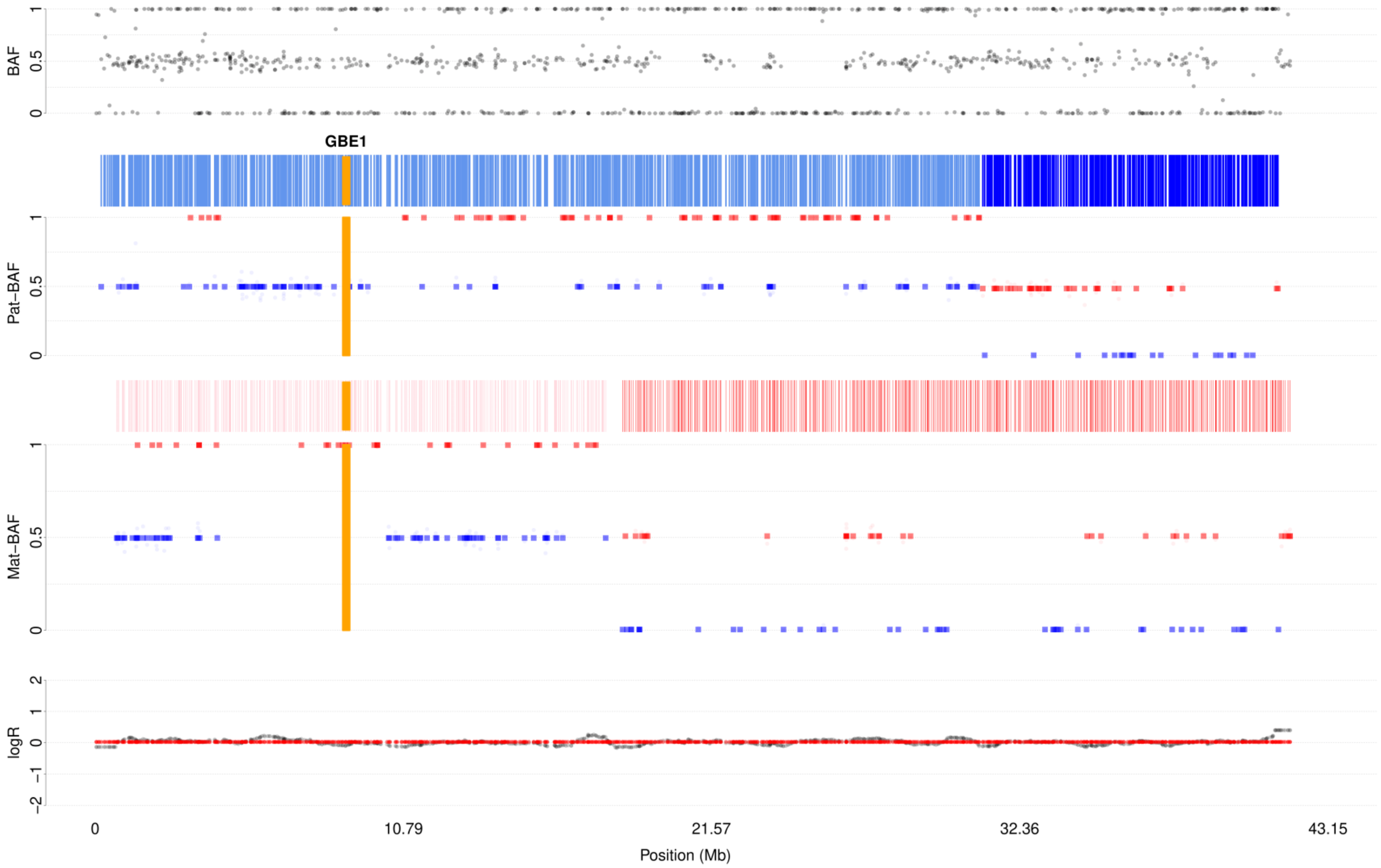

Mare02_Embryo02_Biopsy_Chromosome01

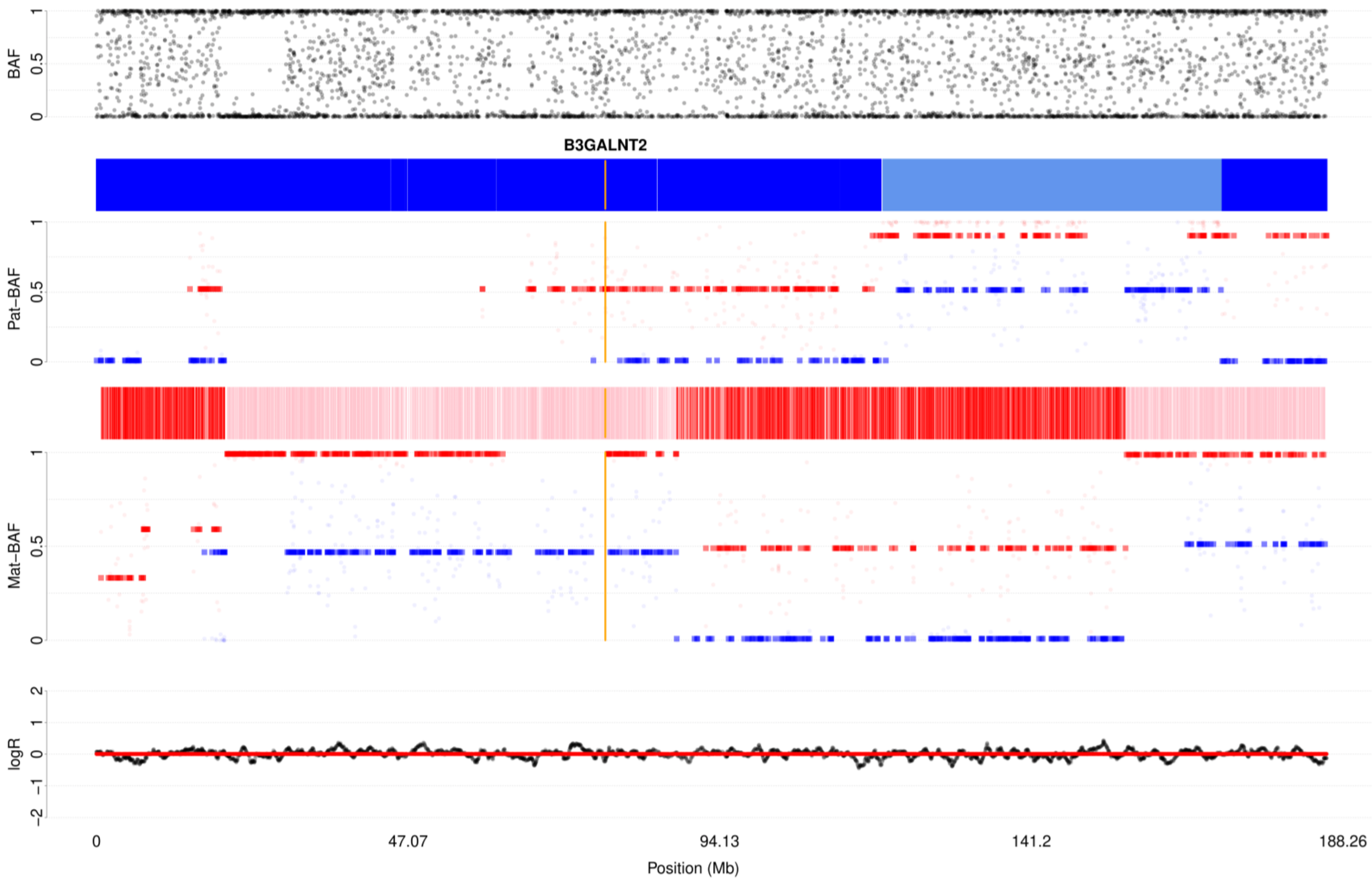

Mare02_Embryo02_Embryo_Chromosome01

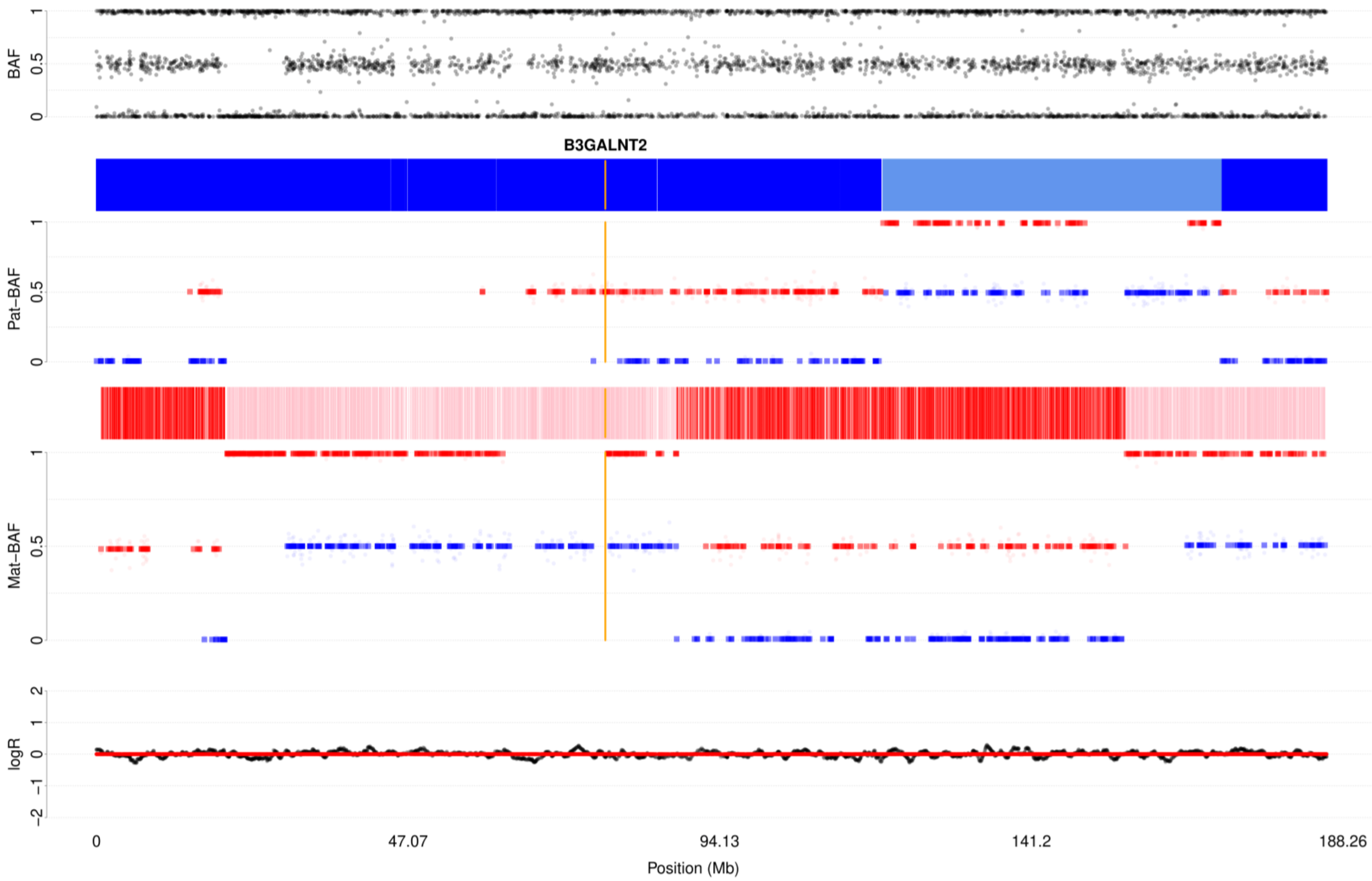

Mare02_Embryo02_Biopsy_Chromosome02

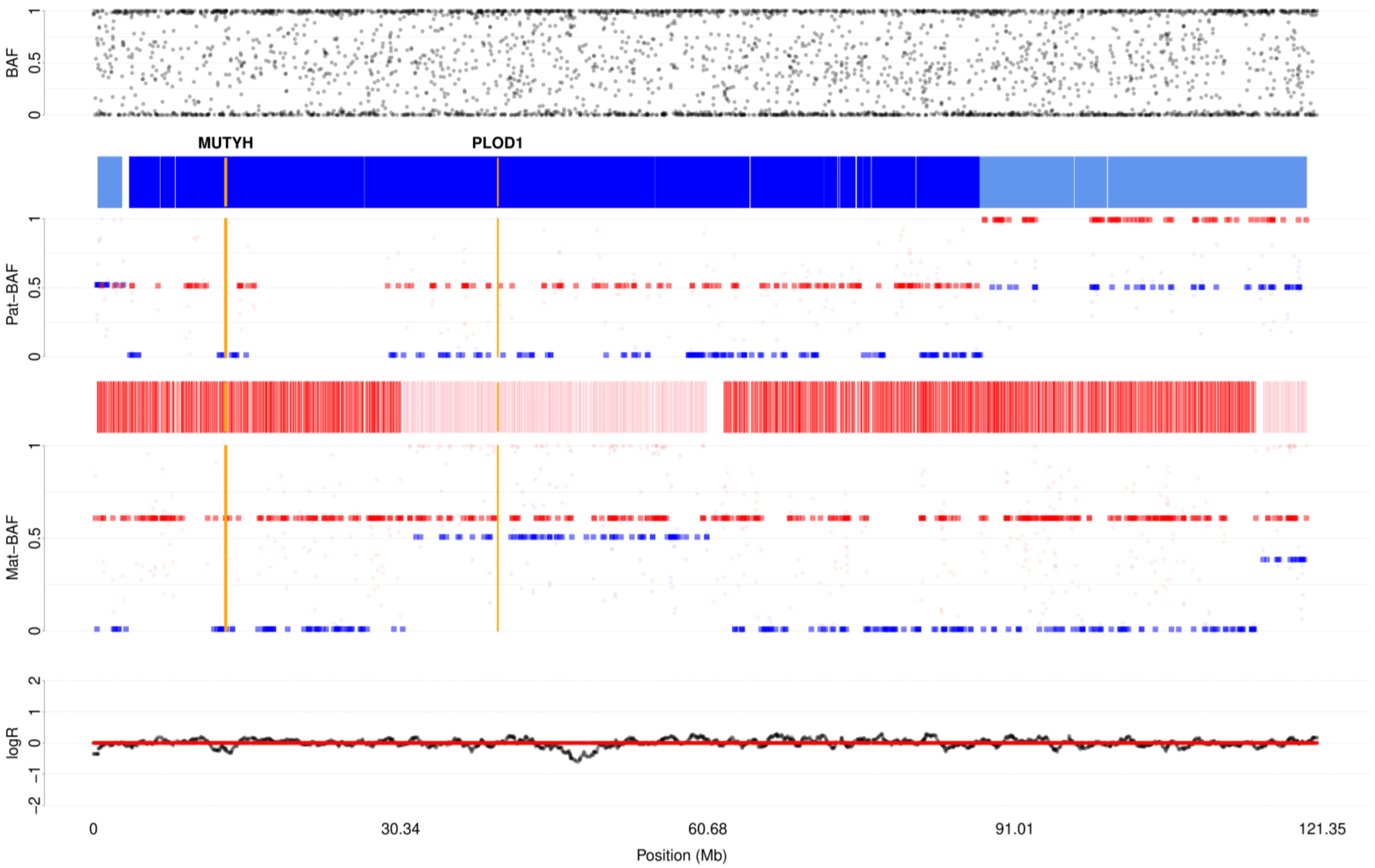

Mare02_Embryo02_Embryo_Chromosome02

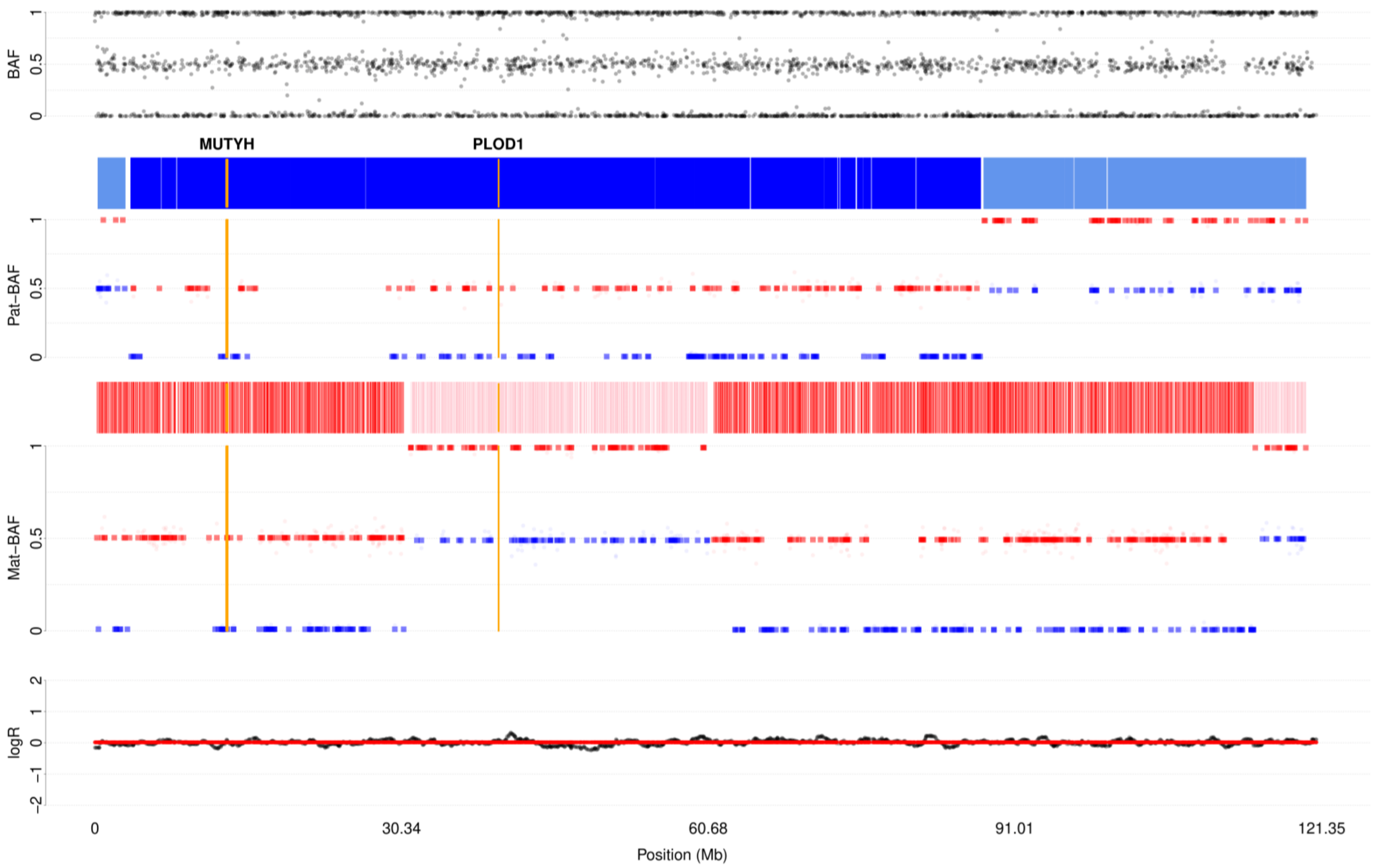

Mare02_Embryo02_Biopsy_Chromosome25

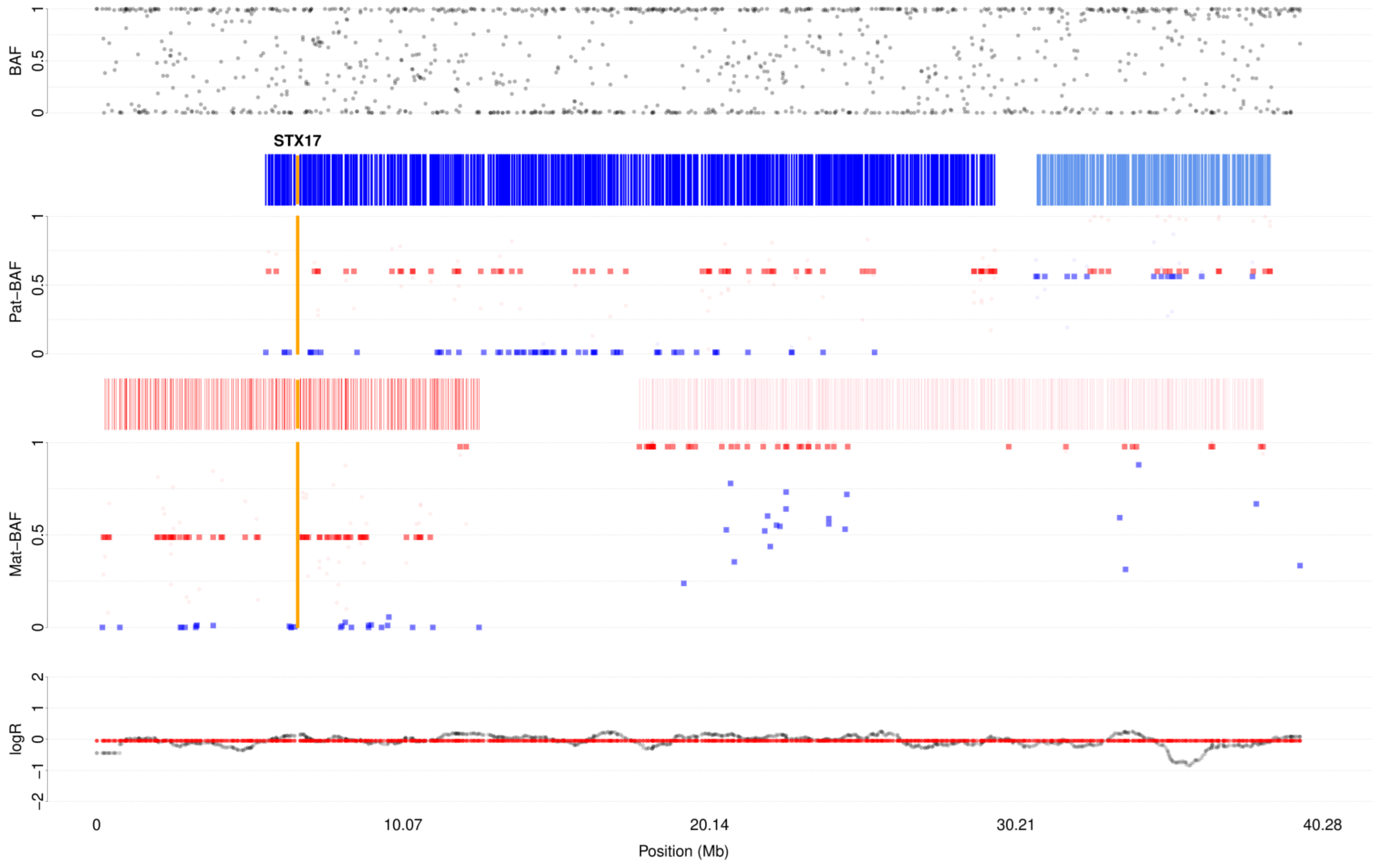

Mare02_Embryo02_Embryo_Chromosome25

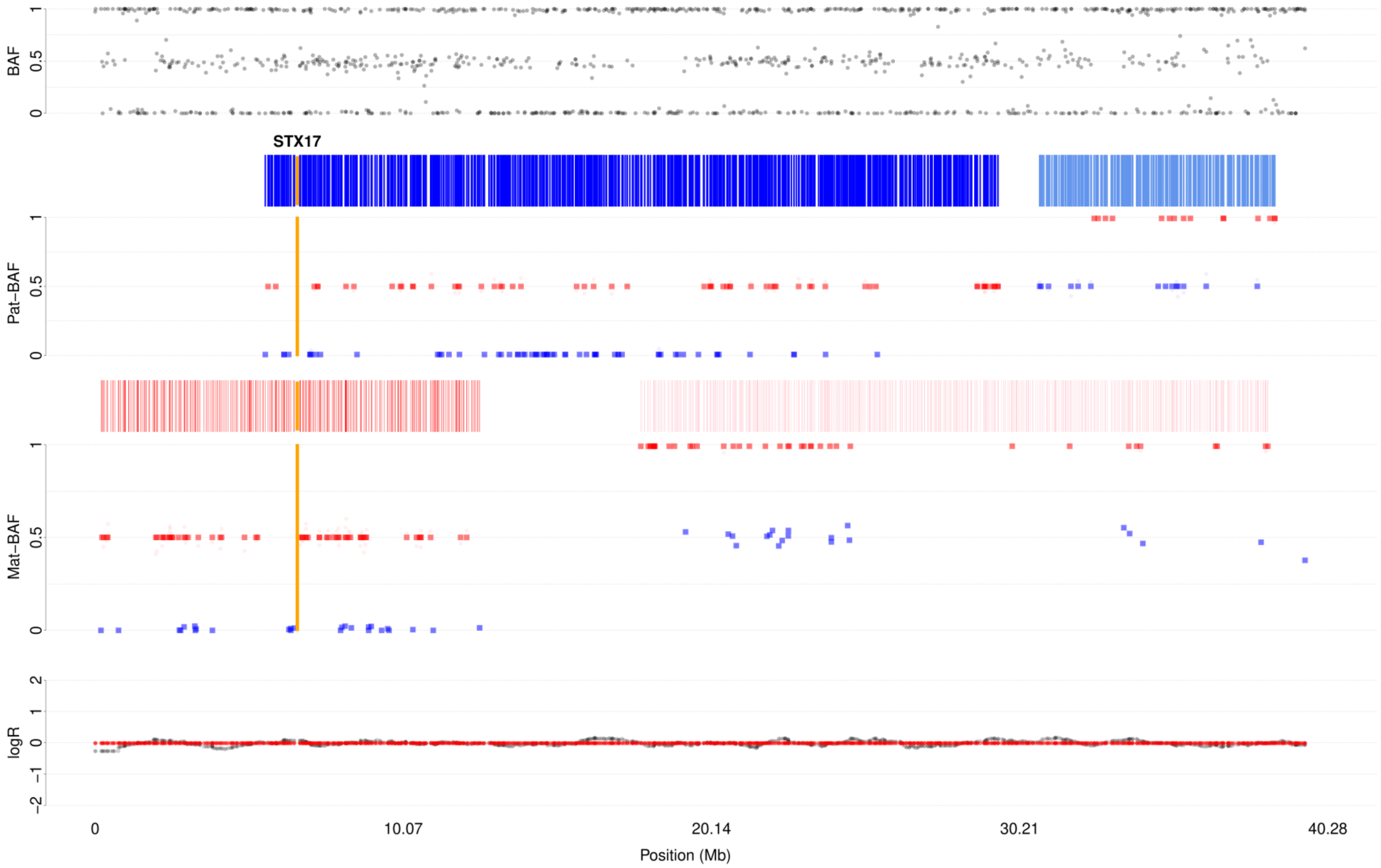

Mare02_Embryo02_Biopsy_Chromosome26

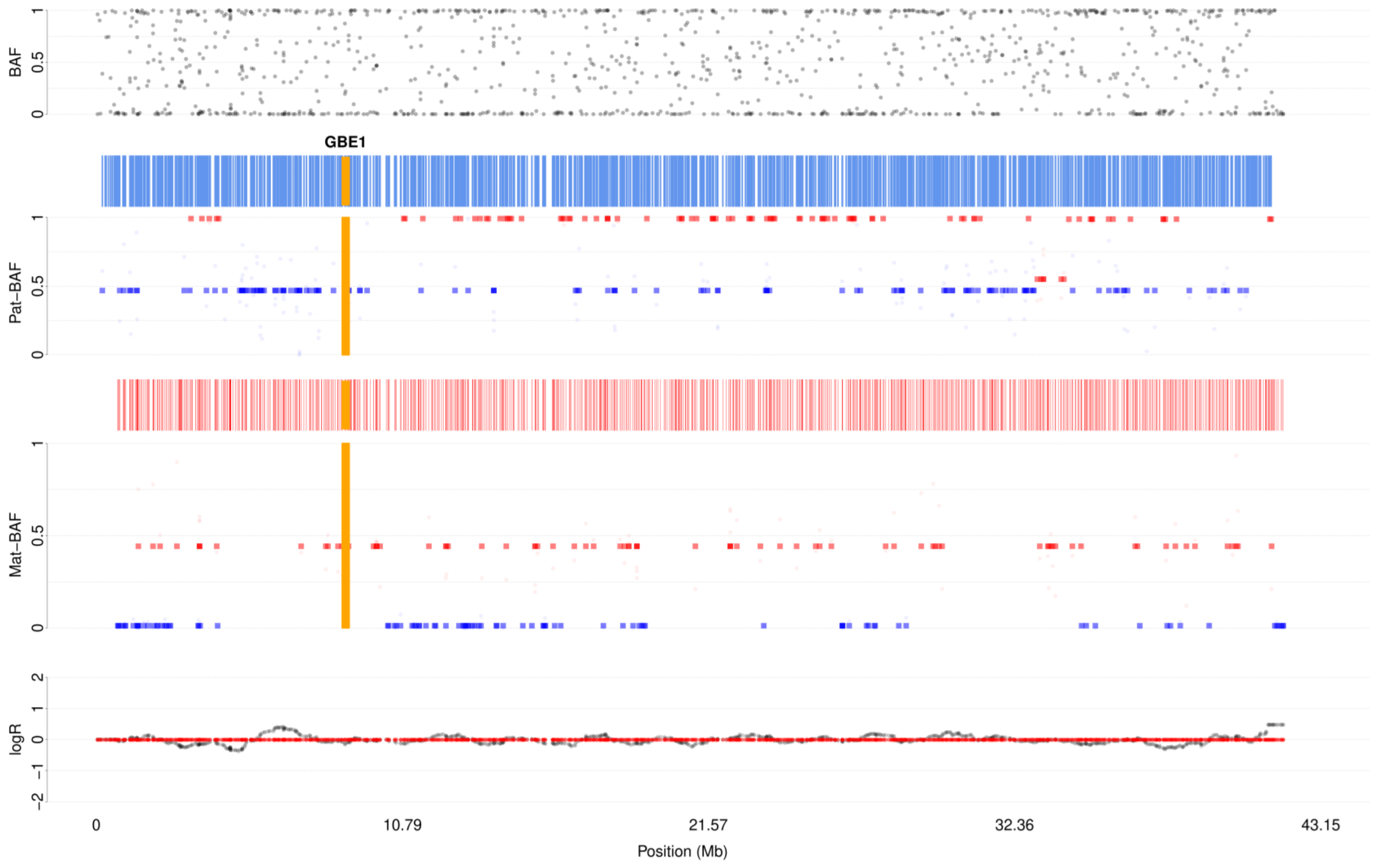

Mare02_Embryo02_Embryo_Chromosome26

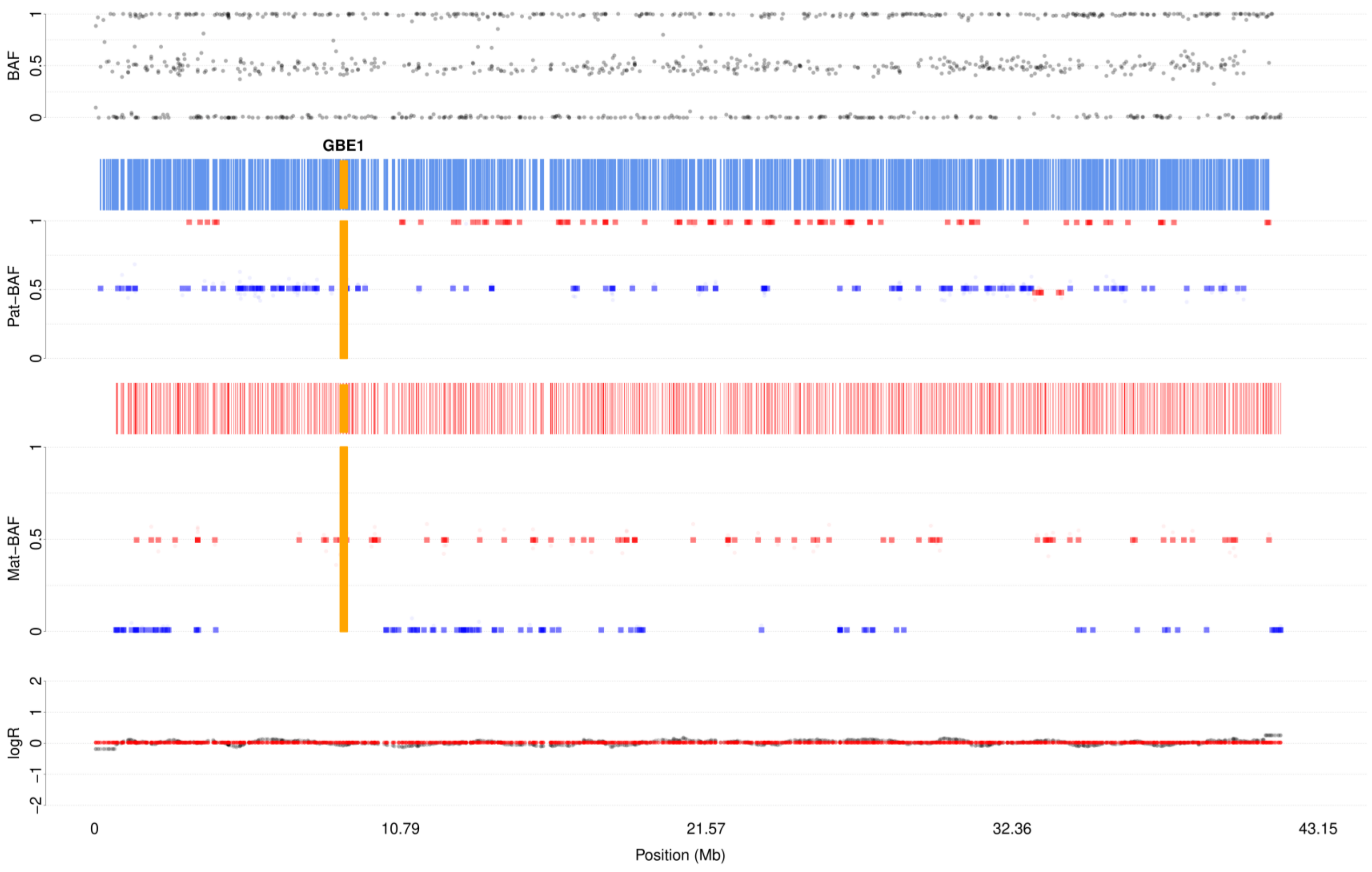

Mare02_Embryo03_Biopsy_Chromosome01

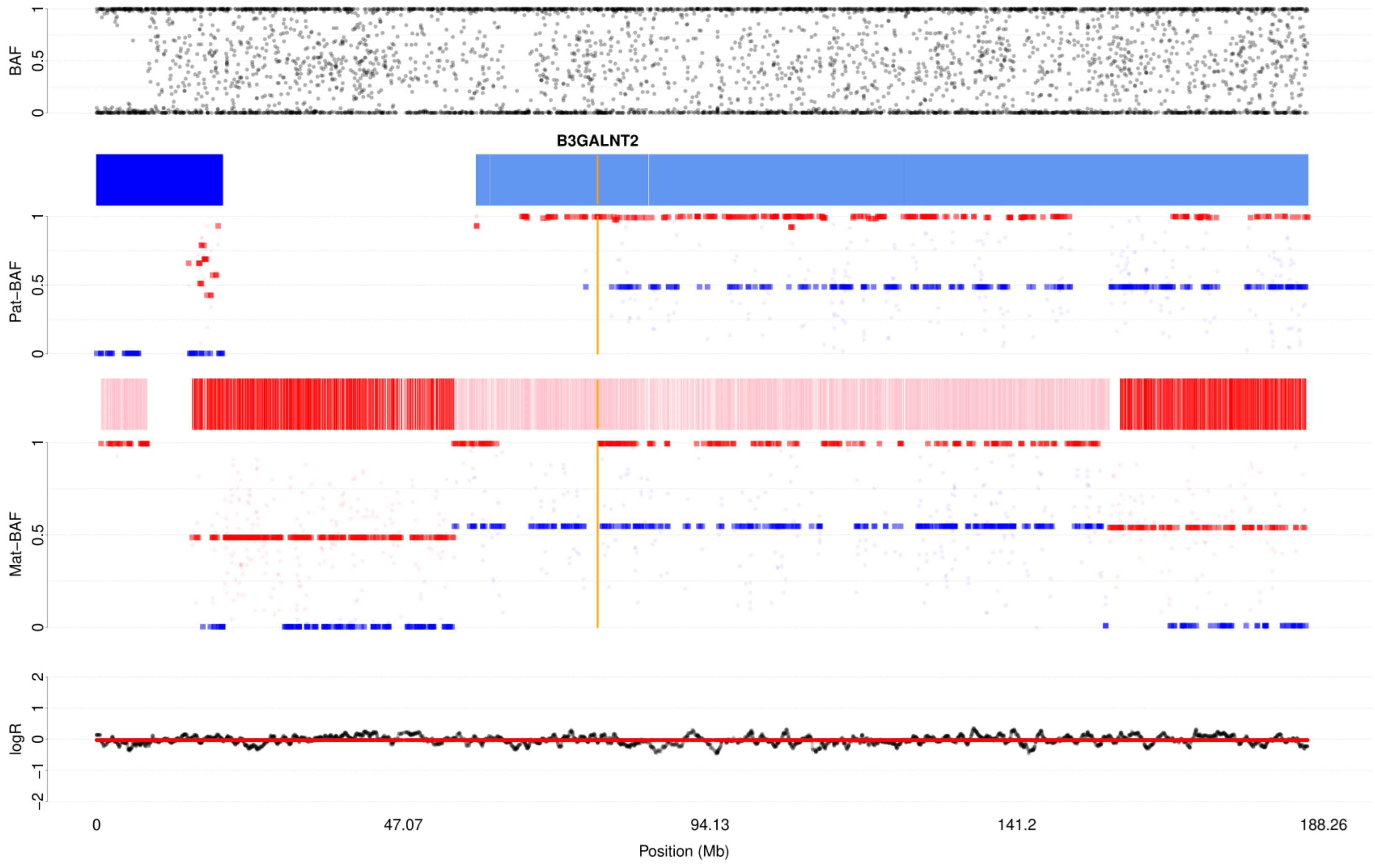

Mare02_Embryo03_Embryo_Chromosome01

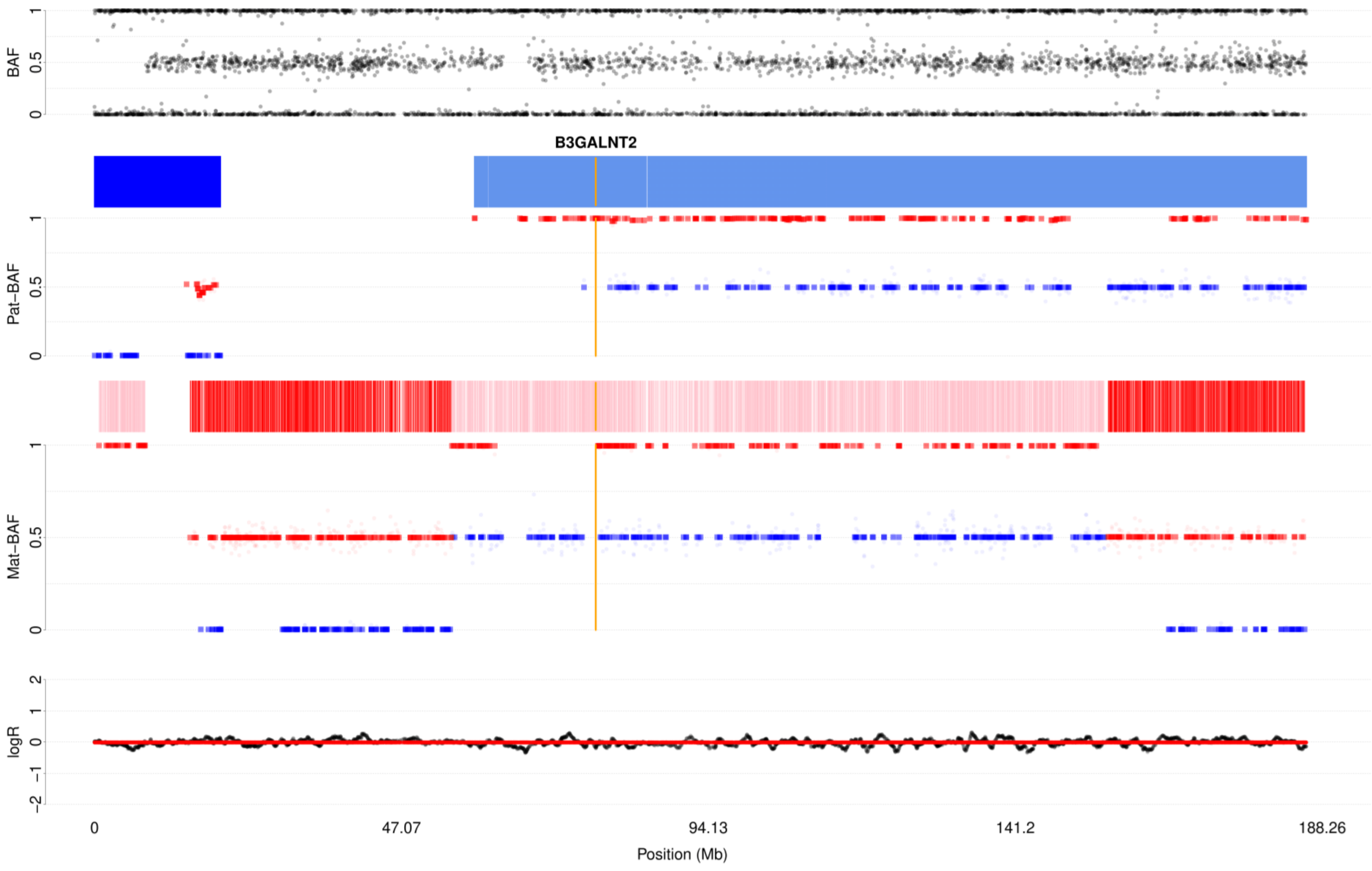

Mare02_Embryo03_Biopsy_Chromosome02

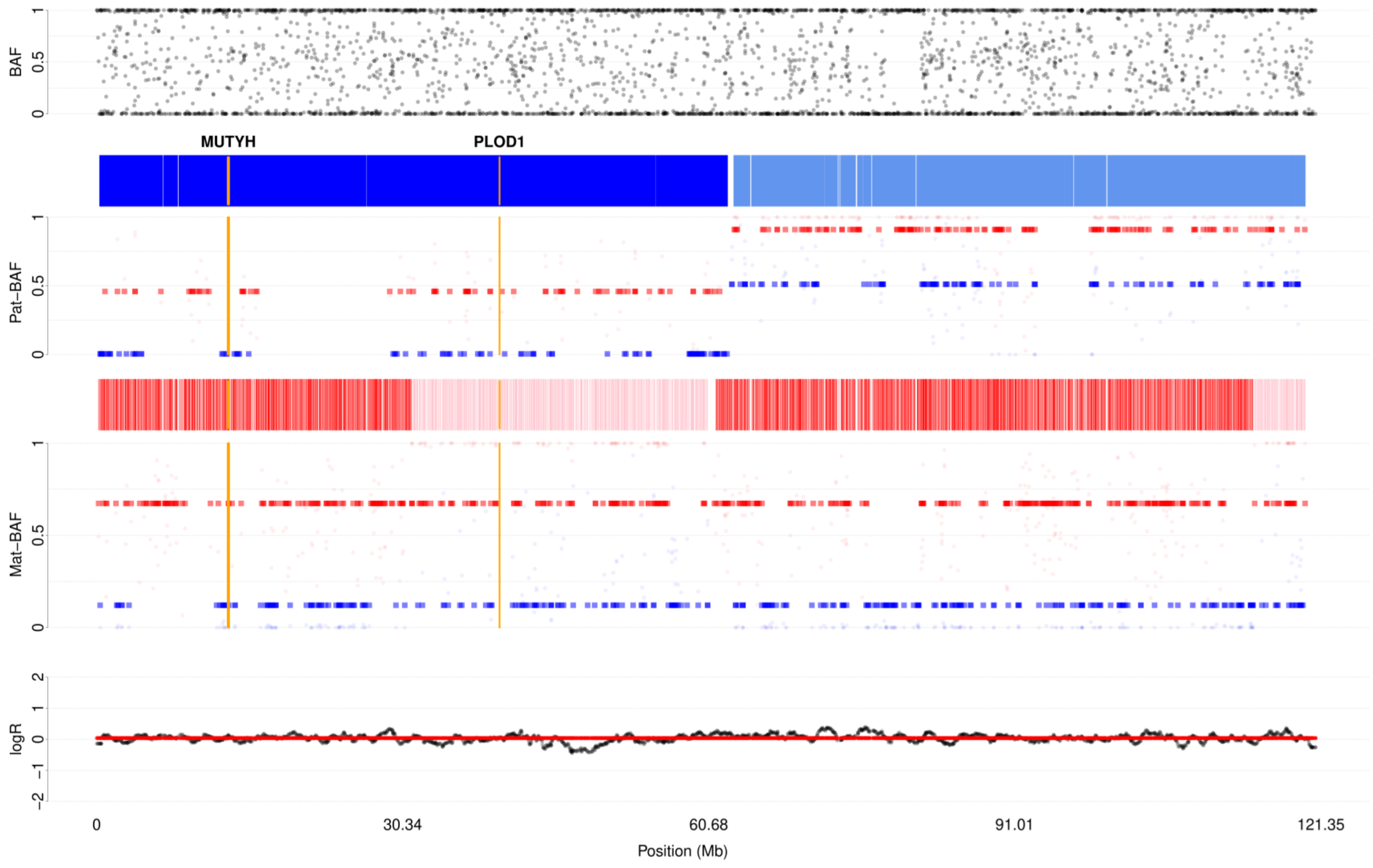

Mare02_Embryo03_Embryo_Chromosome02

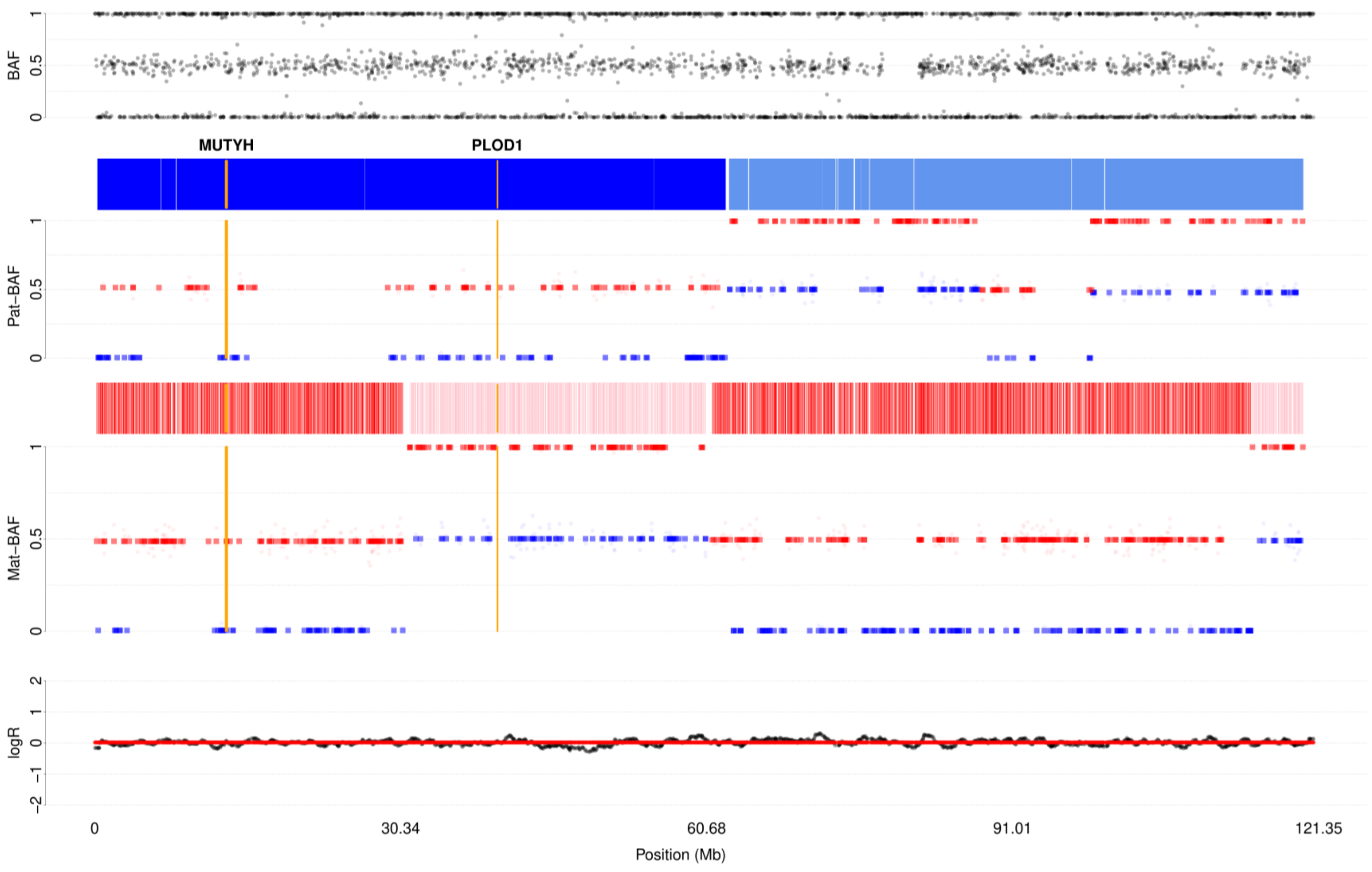

Mare02_Embryo03_Biopsy_Chromosome25

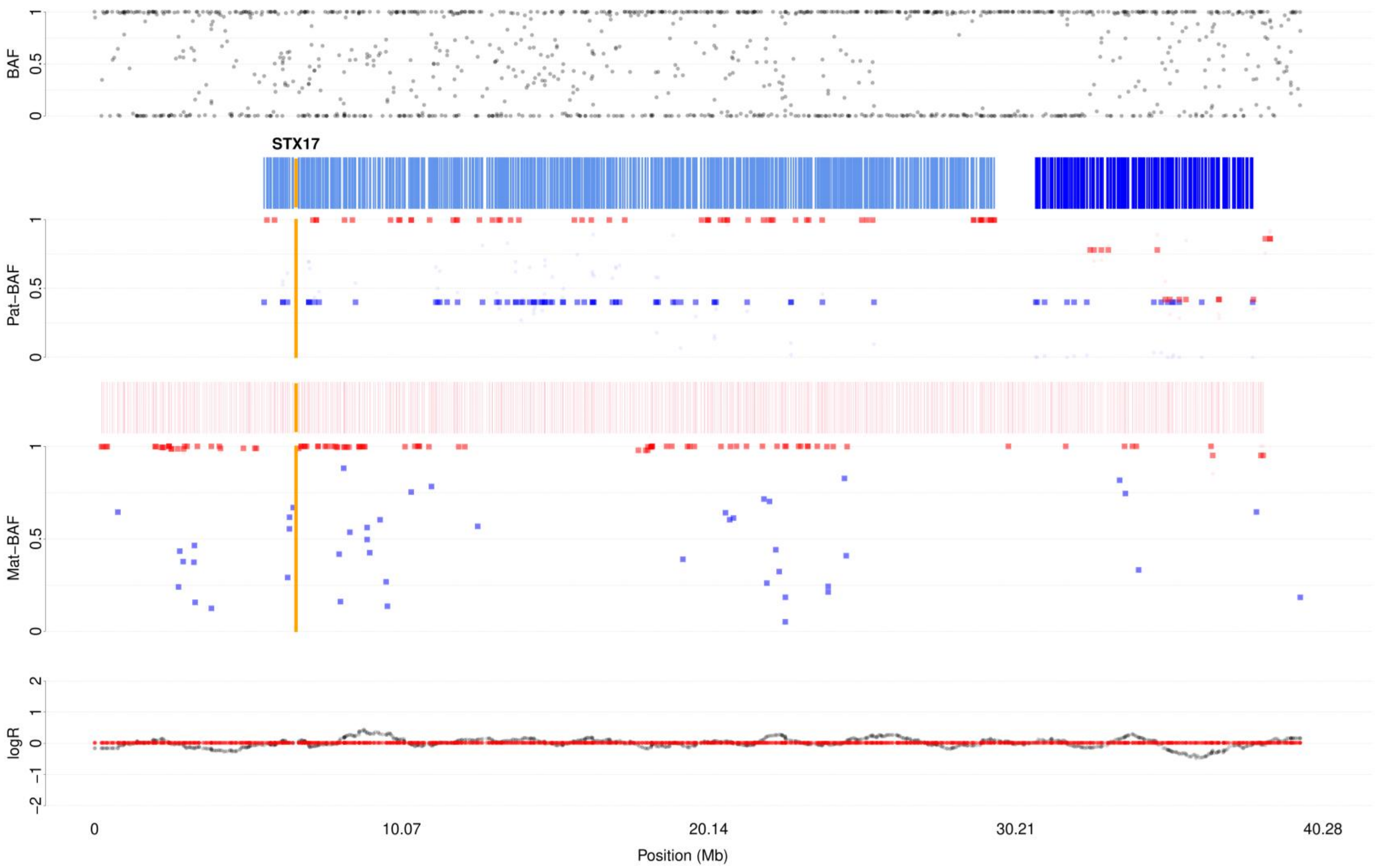

Mare02_Embryo03_Embryo_Chromosome25

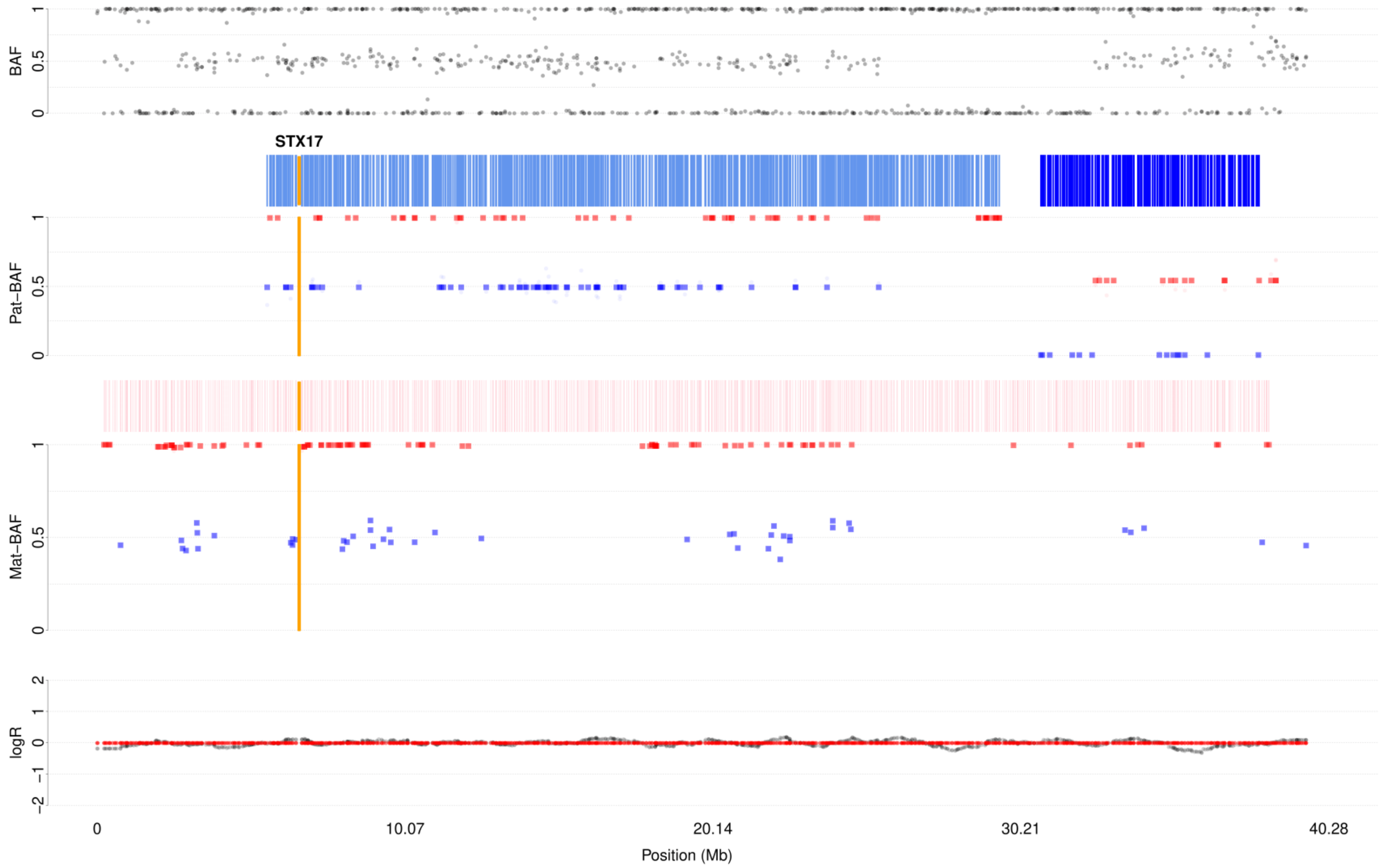

Mare02_Embryo03_Biopsy_Chromosome26

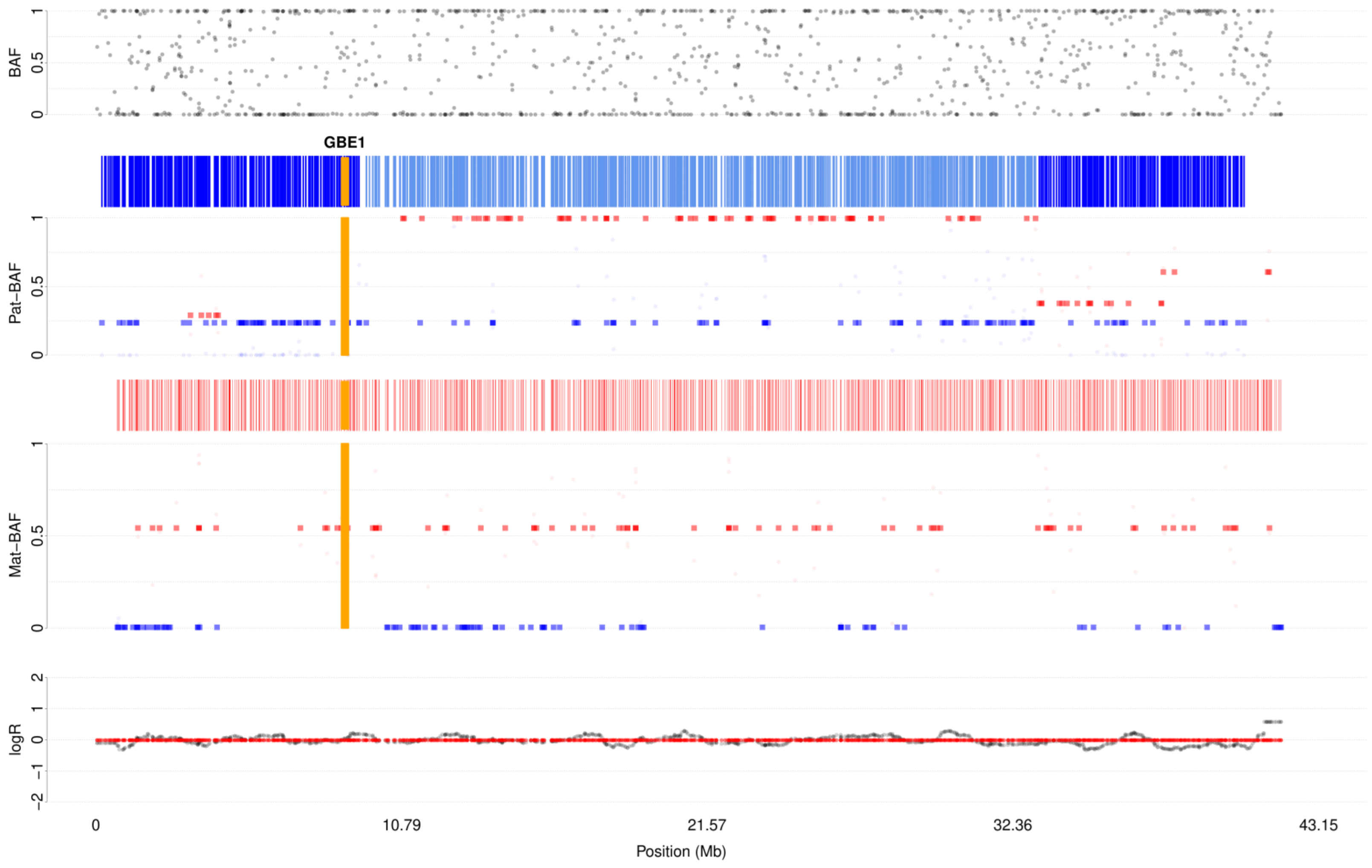

Mare02_Embryo03_Embryo_Chromosome26

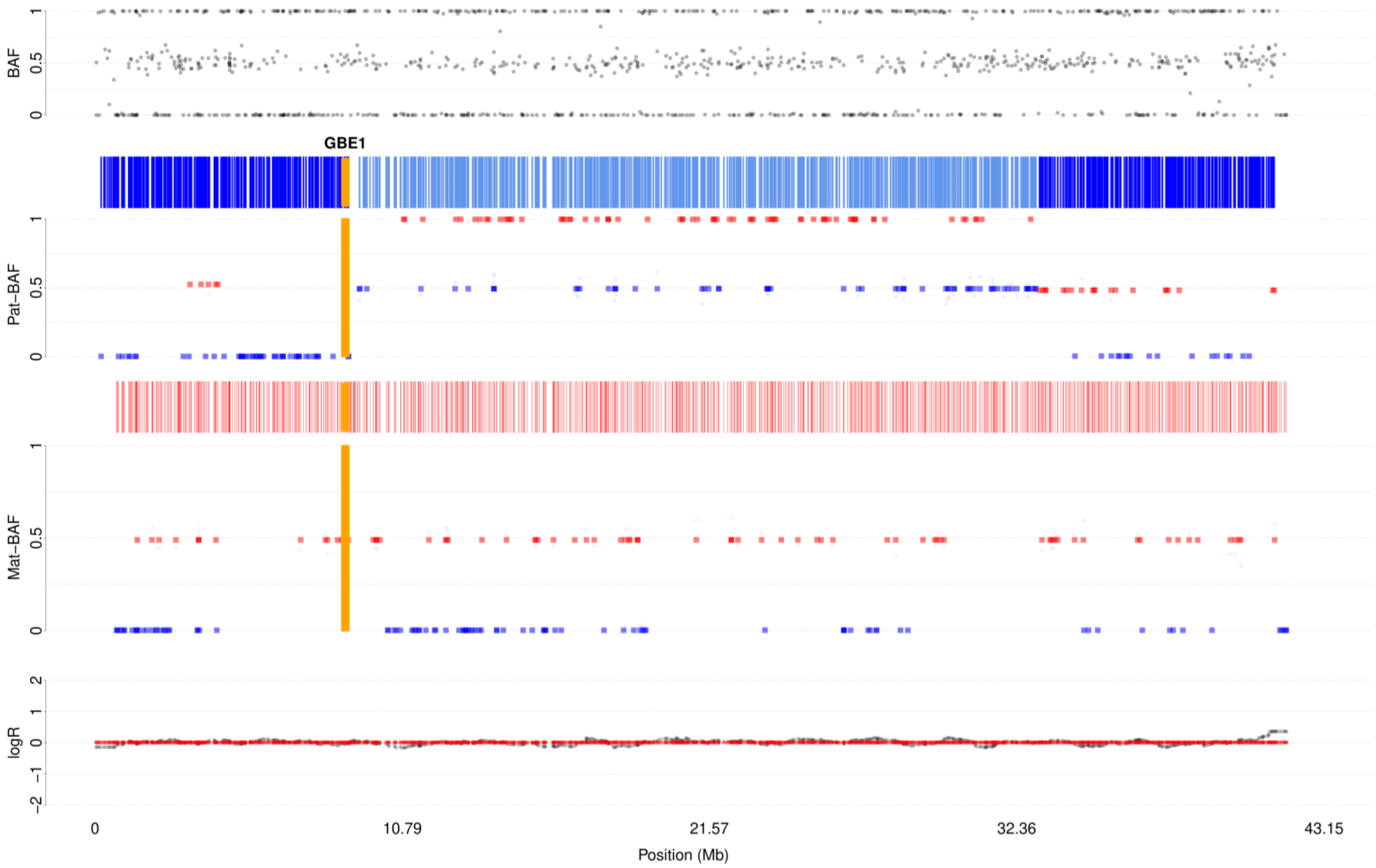

Mare02_Embryo05_Biopsy_Chromosome01

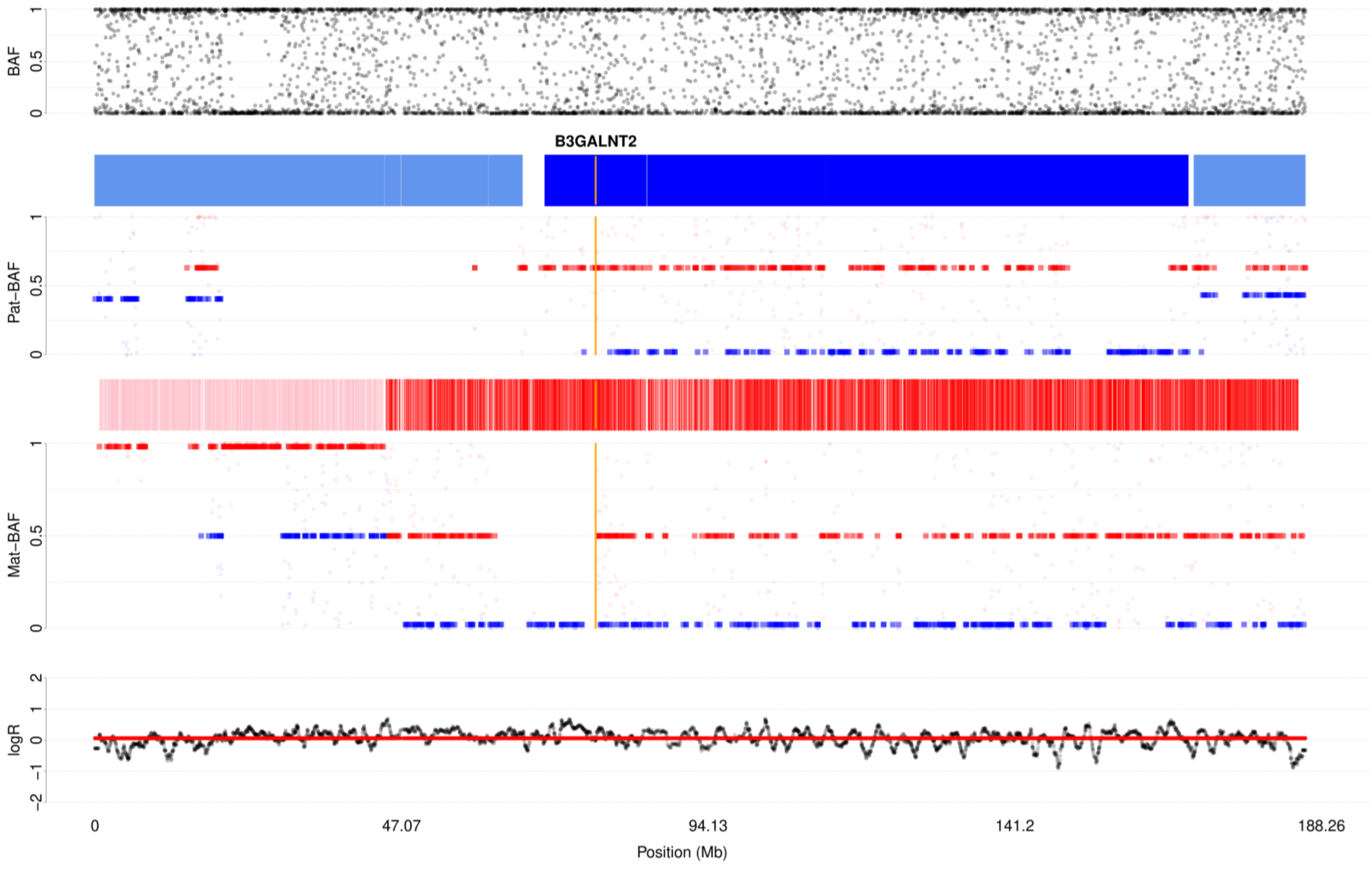

Mare02_Embryo05_Embryo_Chromosome01

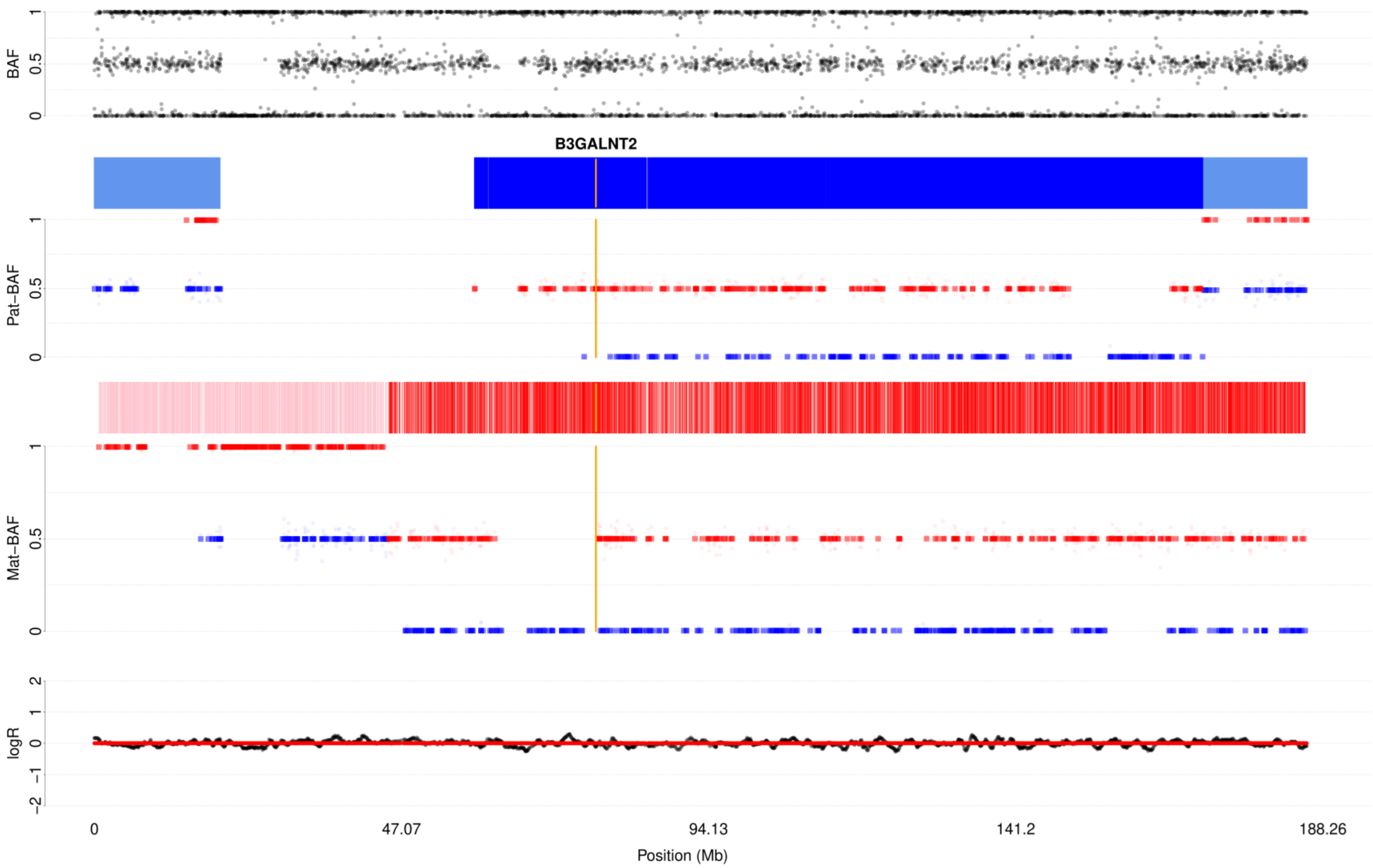

Mare02_Embryo05_Biopsy_Chromosome02

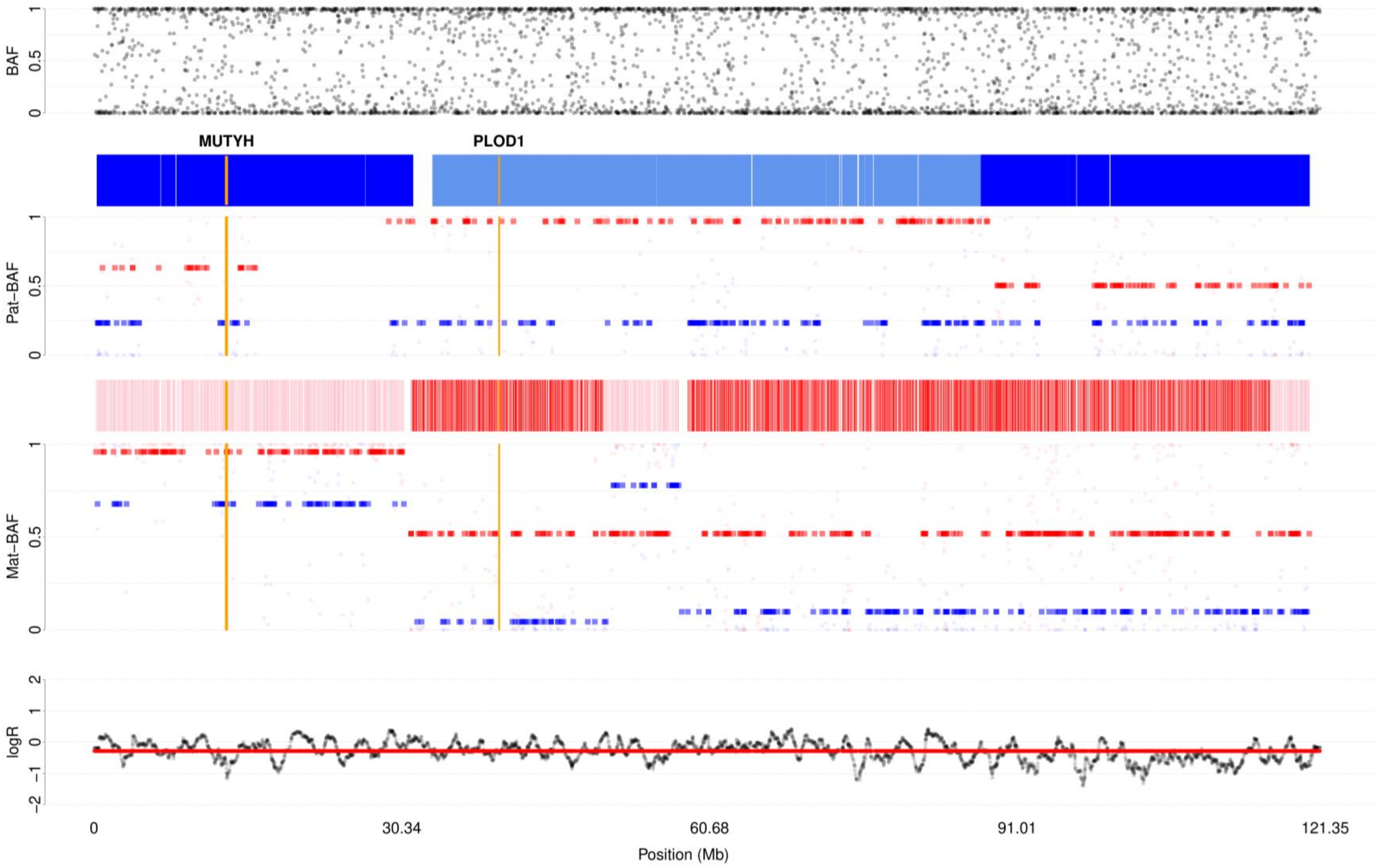

Mare02_Embryo05_Embryo_Chromosome02

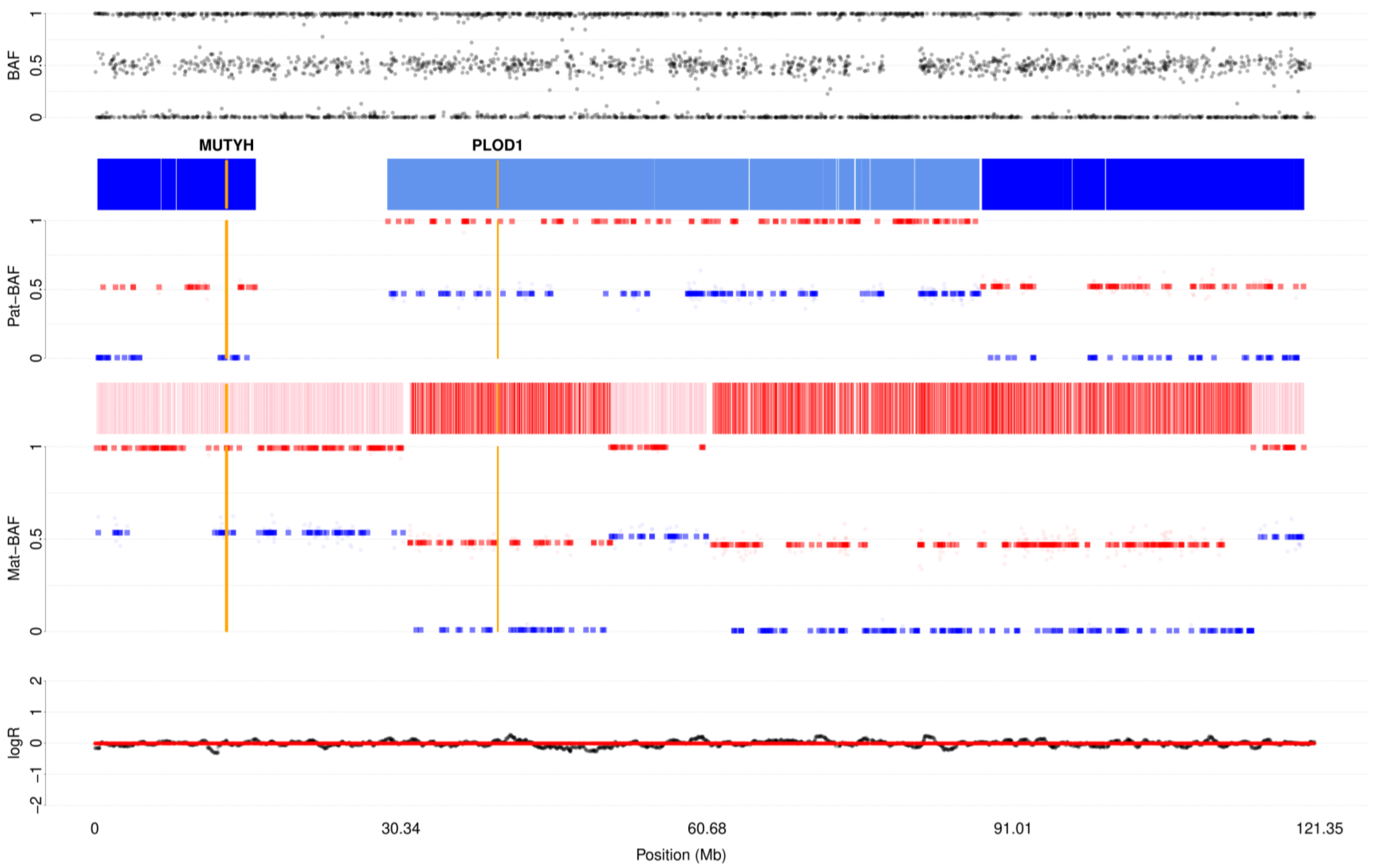

Mare02_Embryo05_Biopsy_Chromosome25

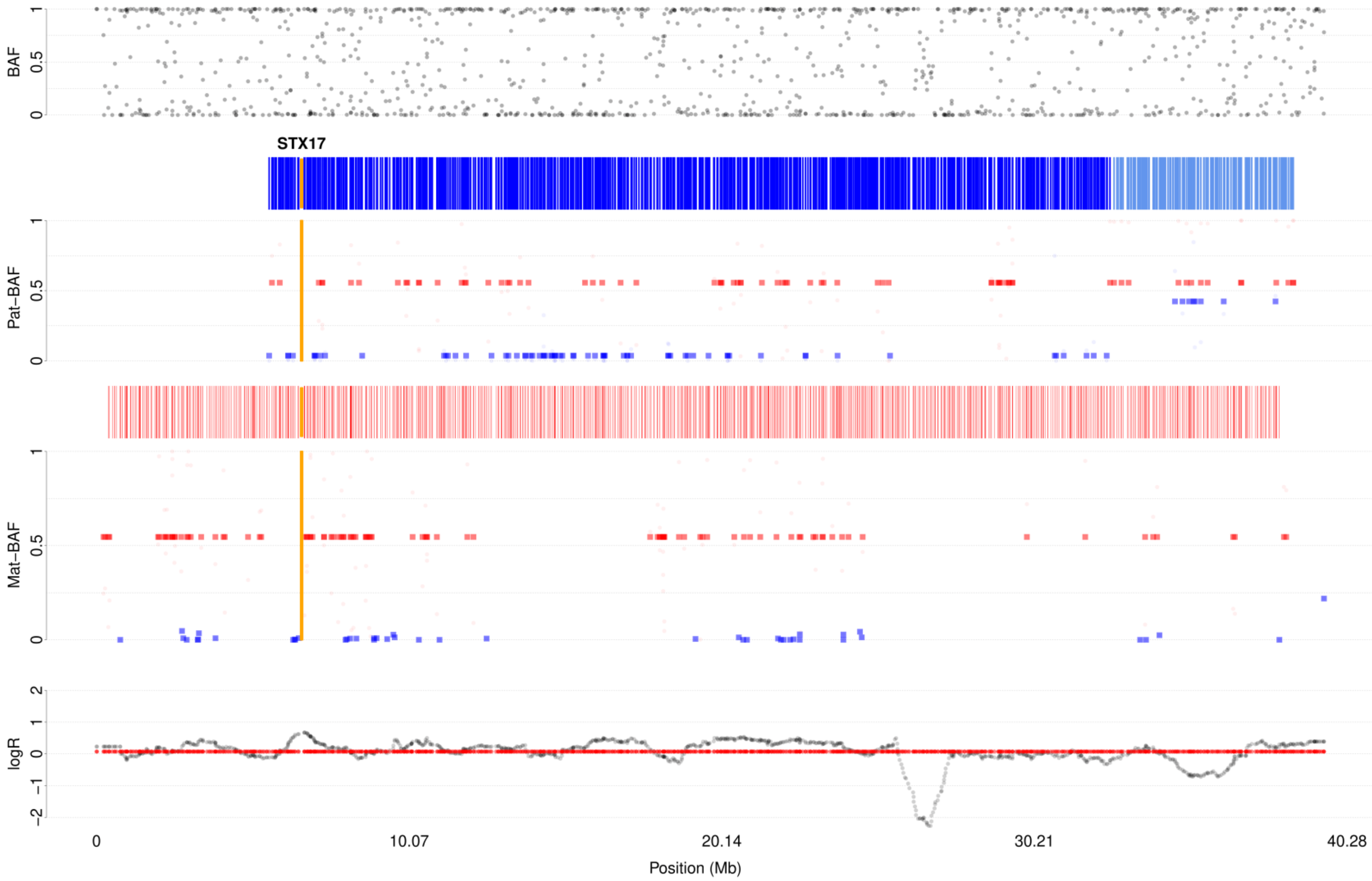

Mare02_Embryo05_Embryo_Chromosome25

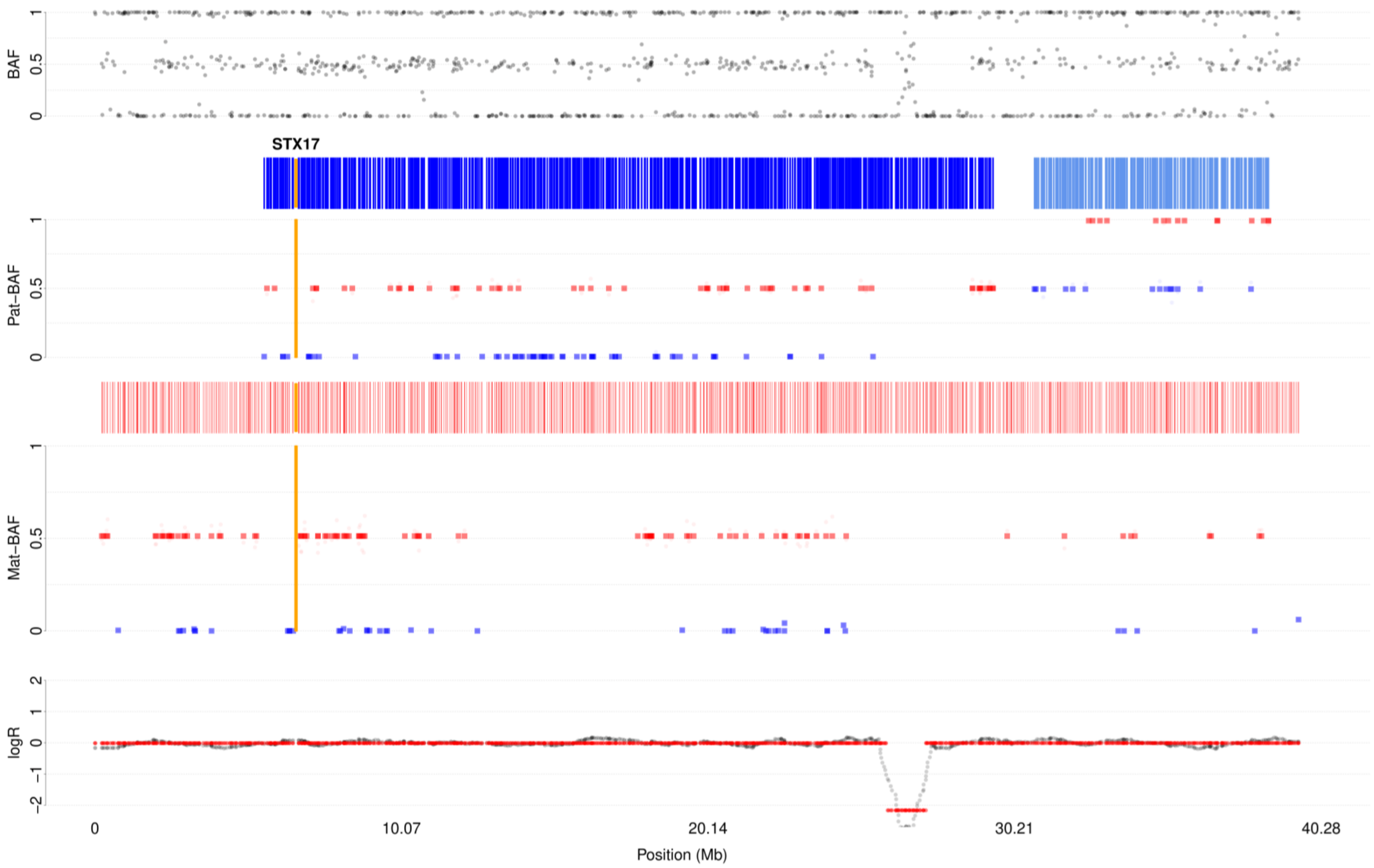

Mare02_Embryo05_Biopsy_Chromosome26

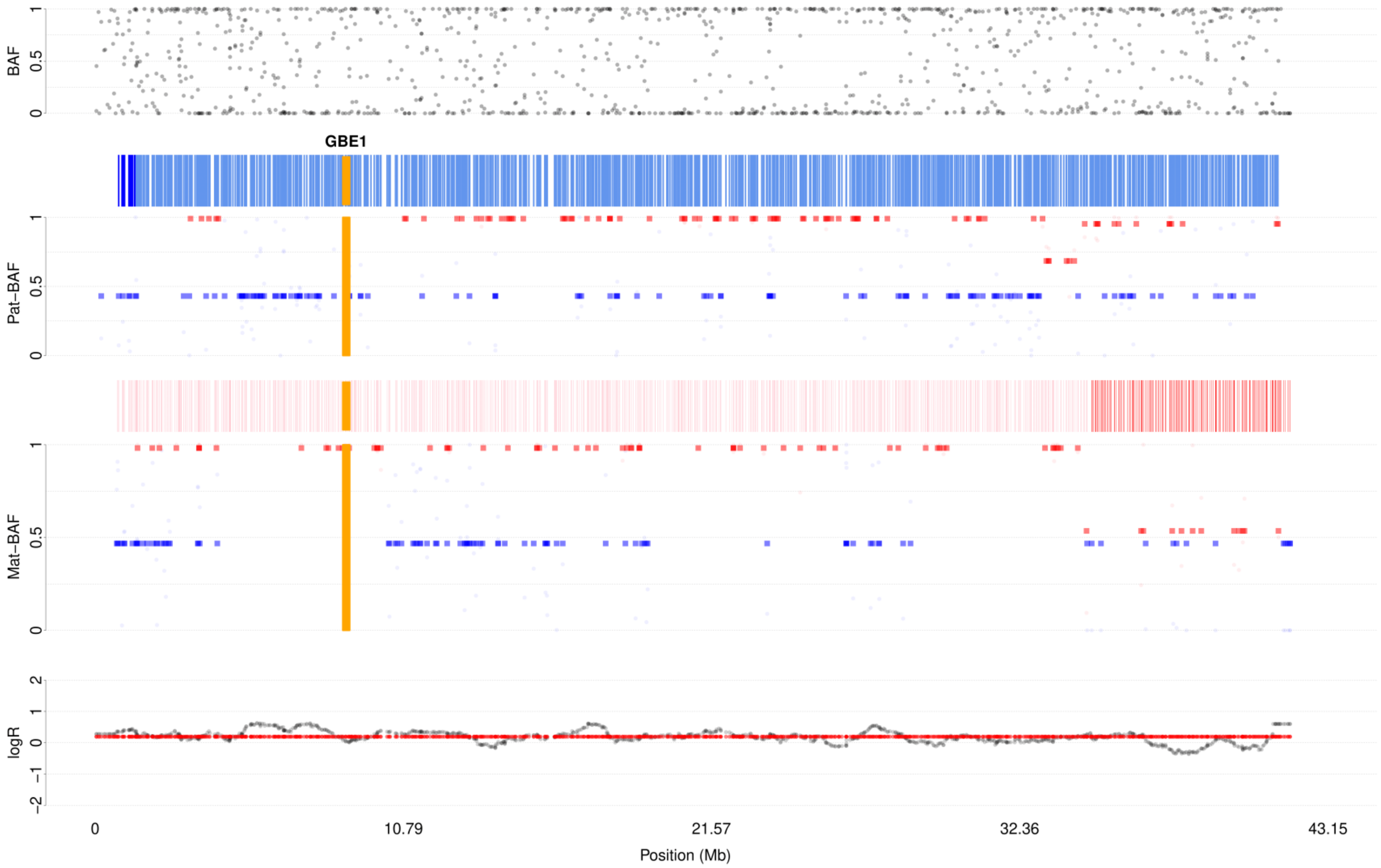

Mare02_Embryo05_Embryo_Chromosome26

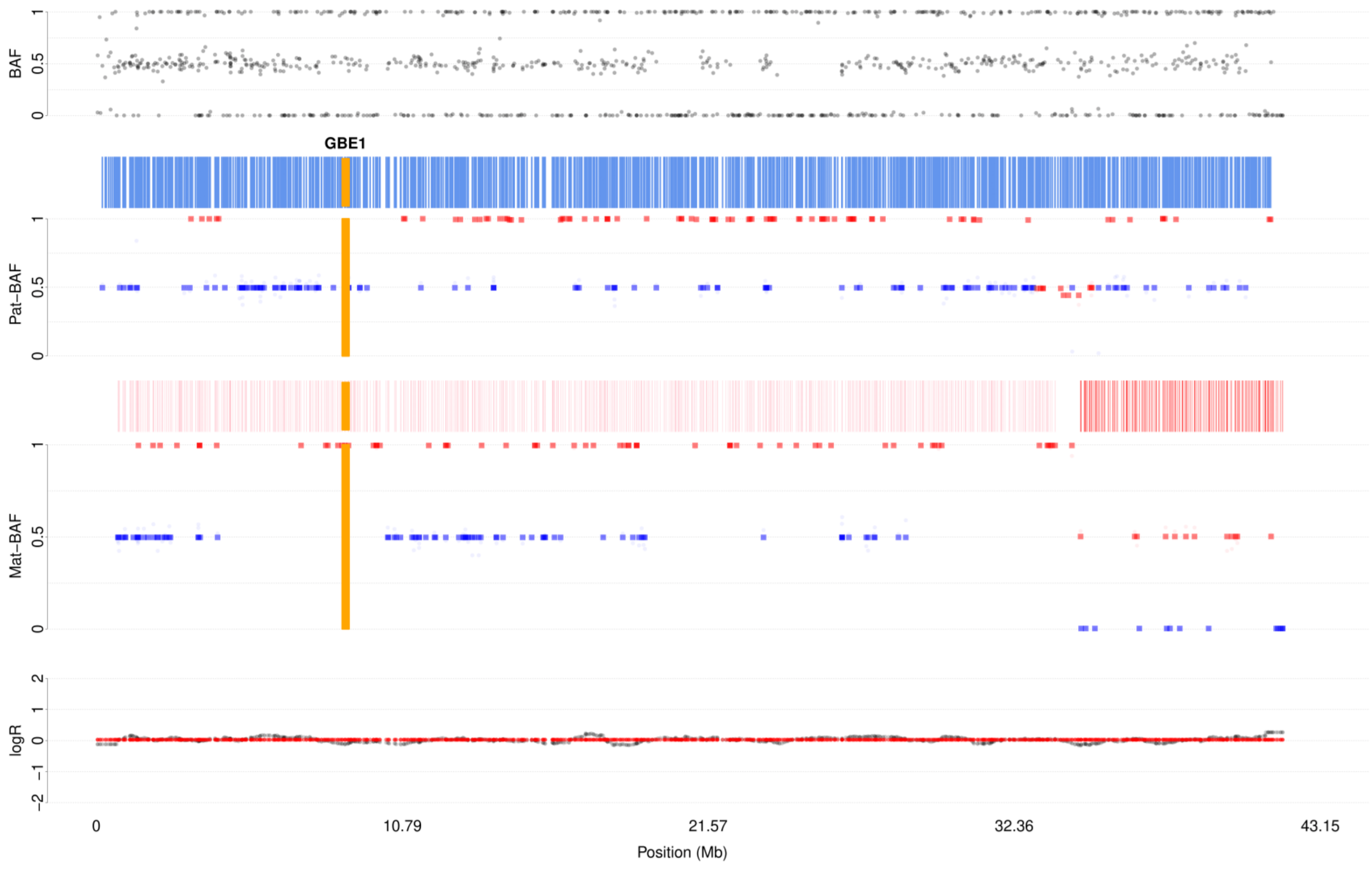

Mare02_Embryo07_Biopsy_Chromosome01

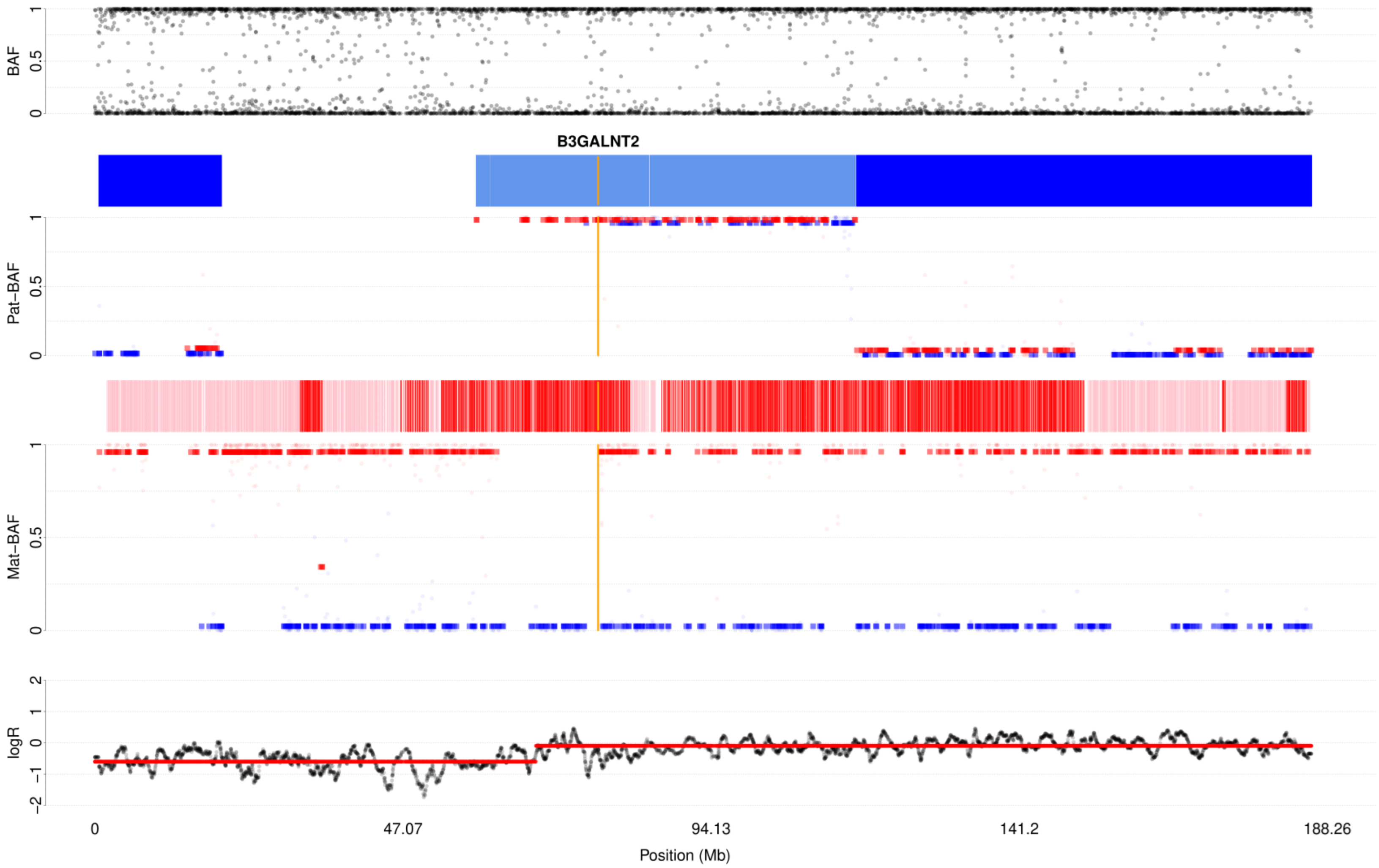

Mare02_Embryo07_Embryo_Chromosome01

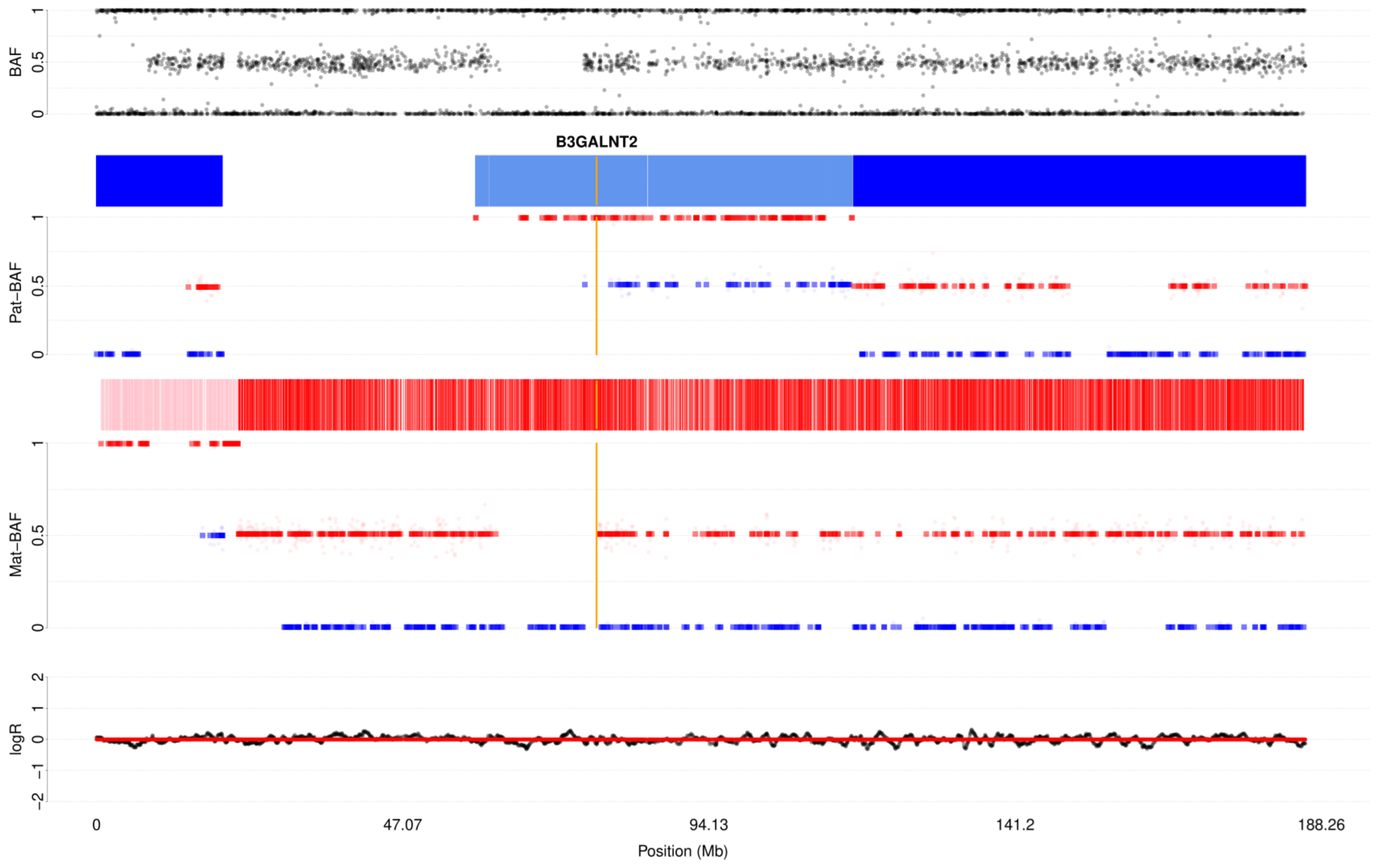

Mare02_Embryo07_Biopsy_Chromosome02

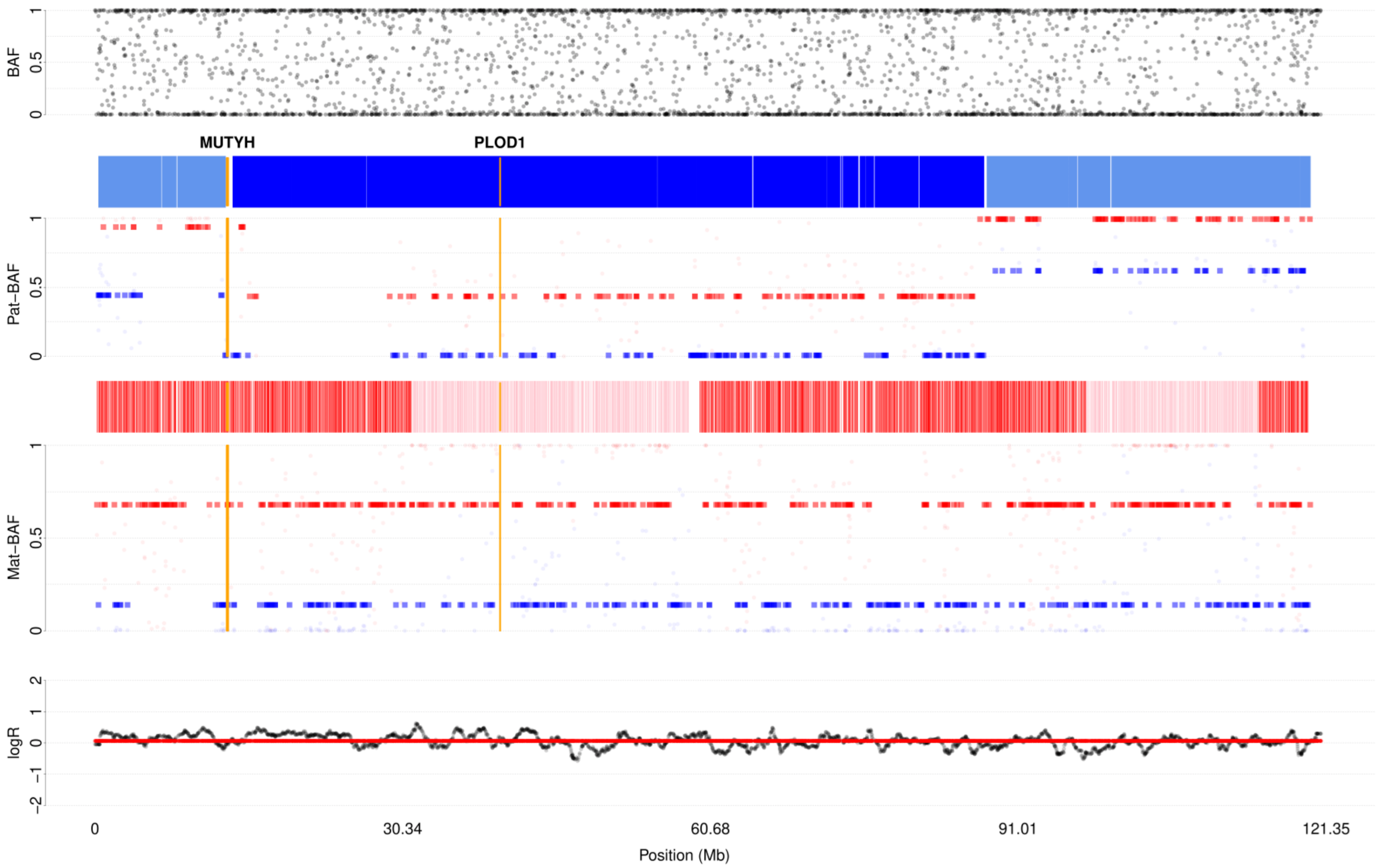

Mare02_Embryo07_Embryo_Chromosome02

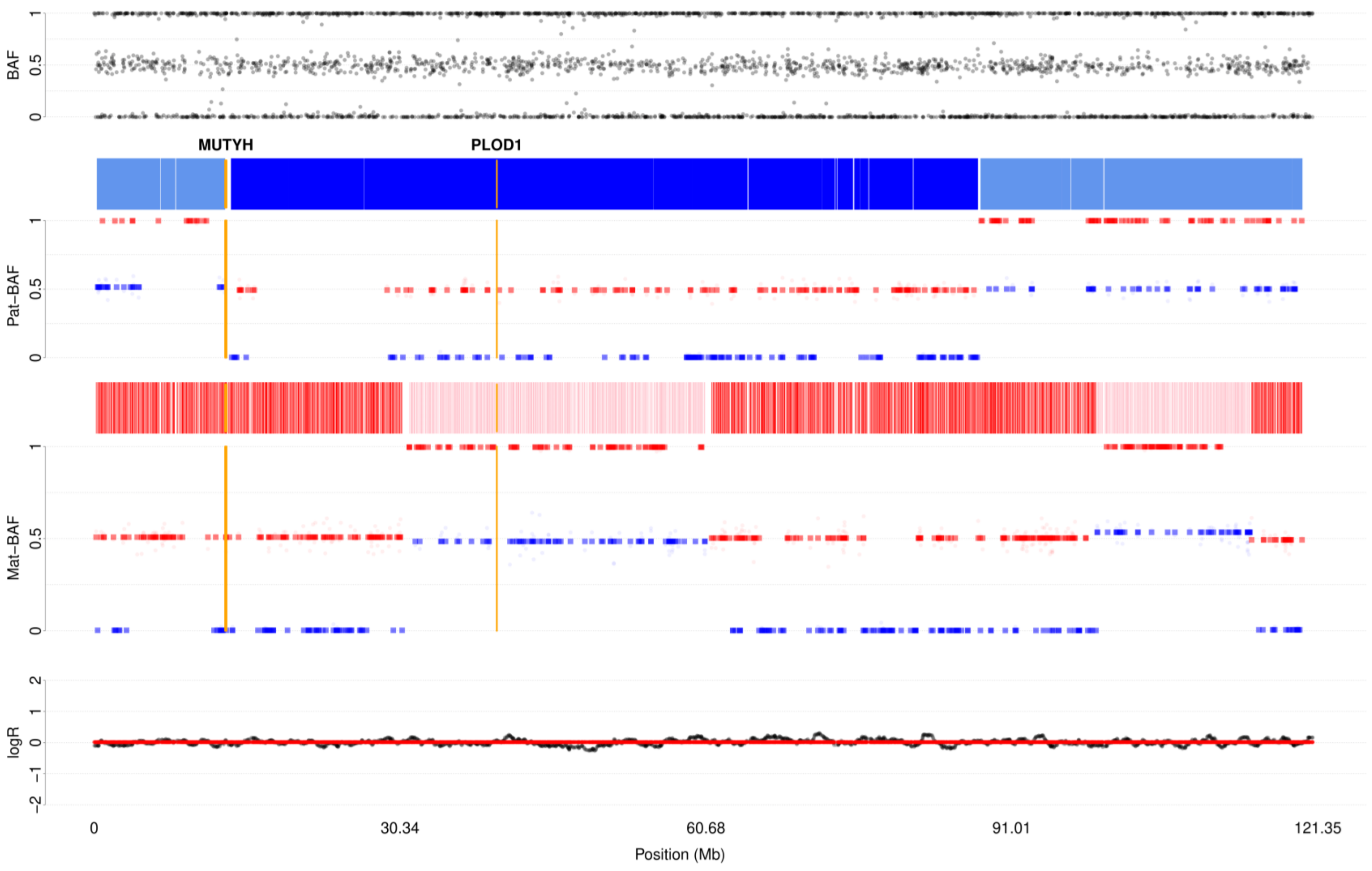

Mare02_Embryo07_Biopsy_Chromosome25

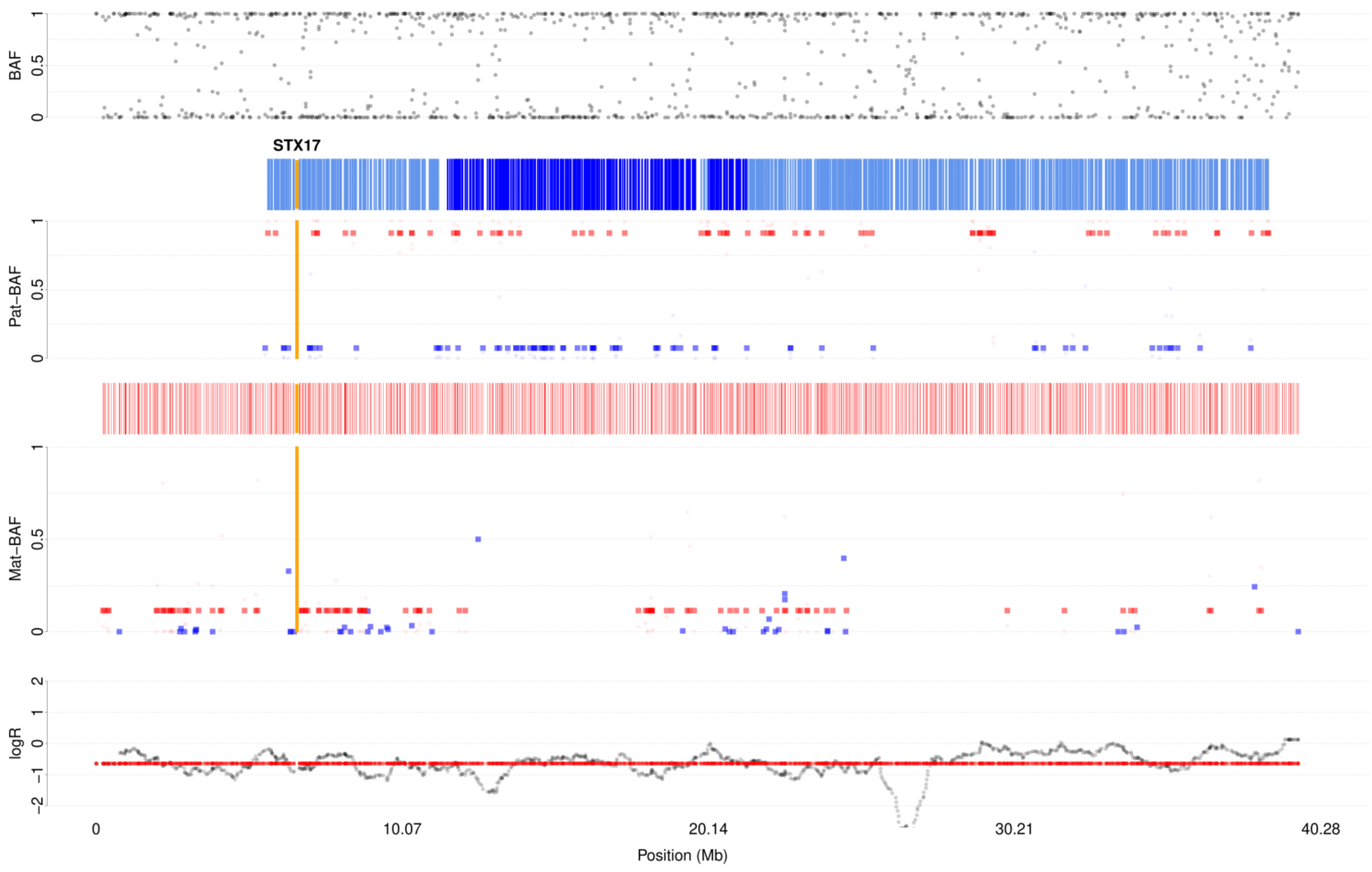

Mare02_Embryo07_Embryo_Chromosome25

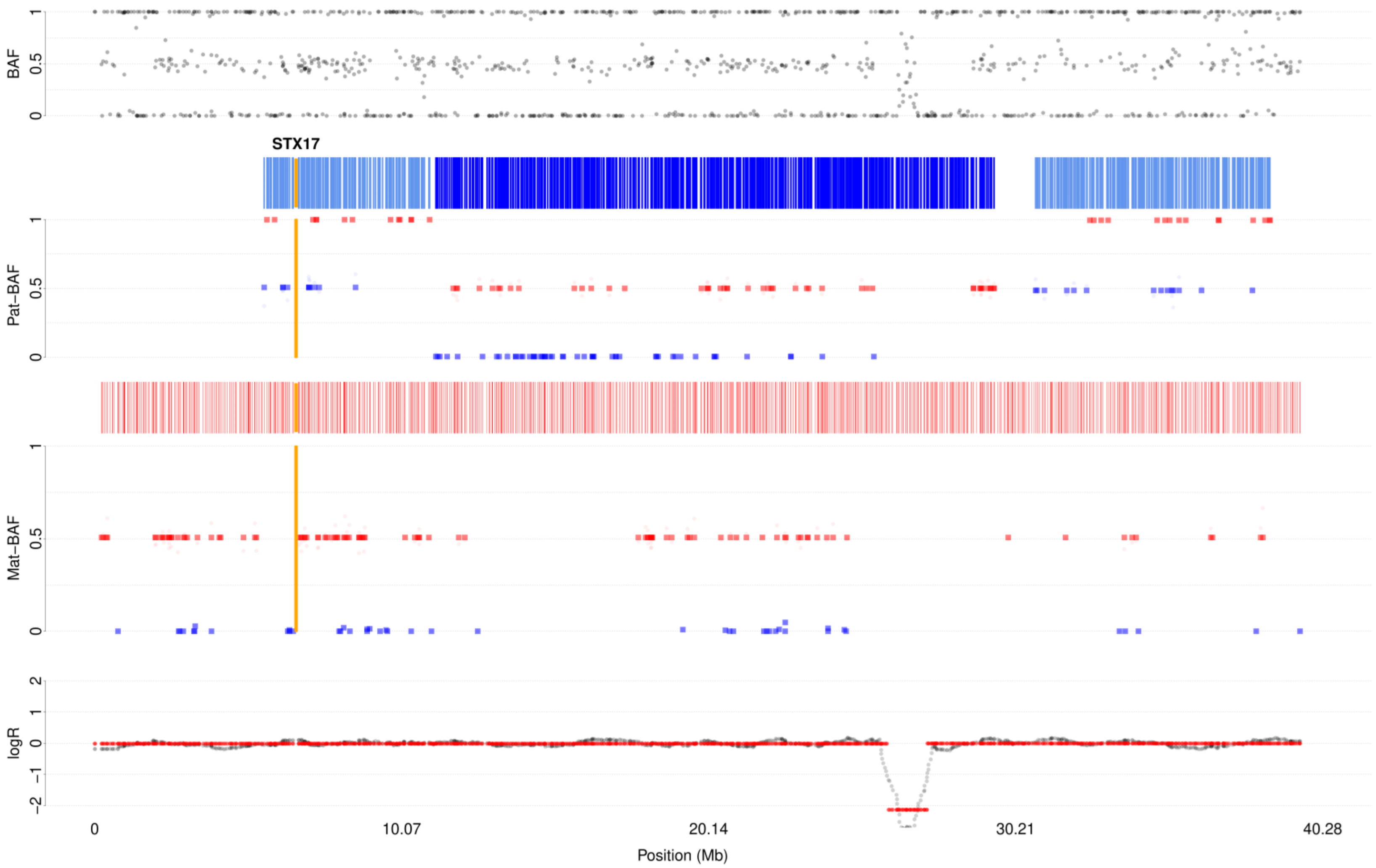

Mare02_Embryo07_Biopsy_Chromosome26

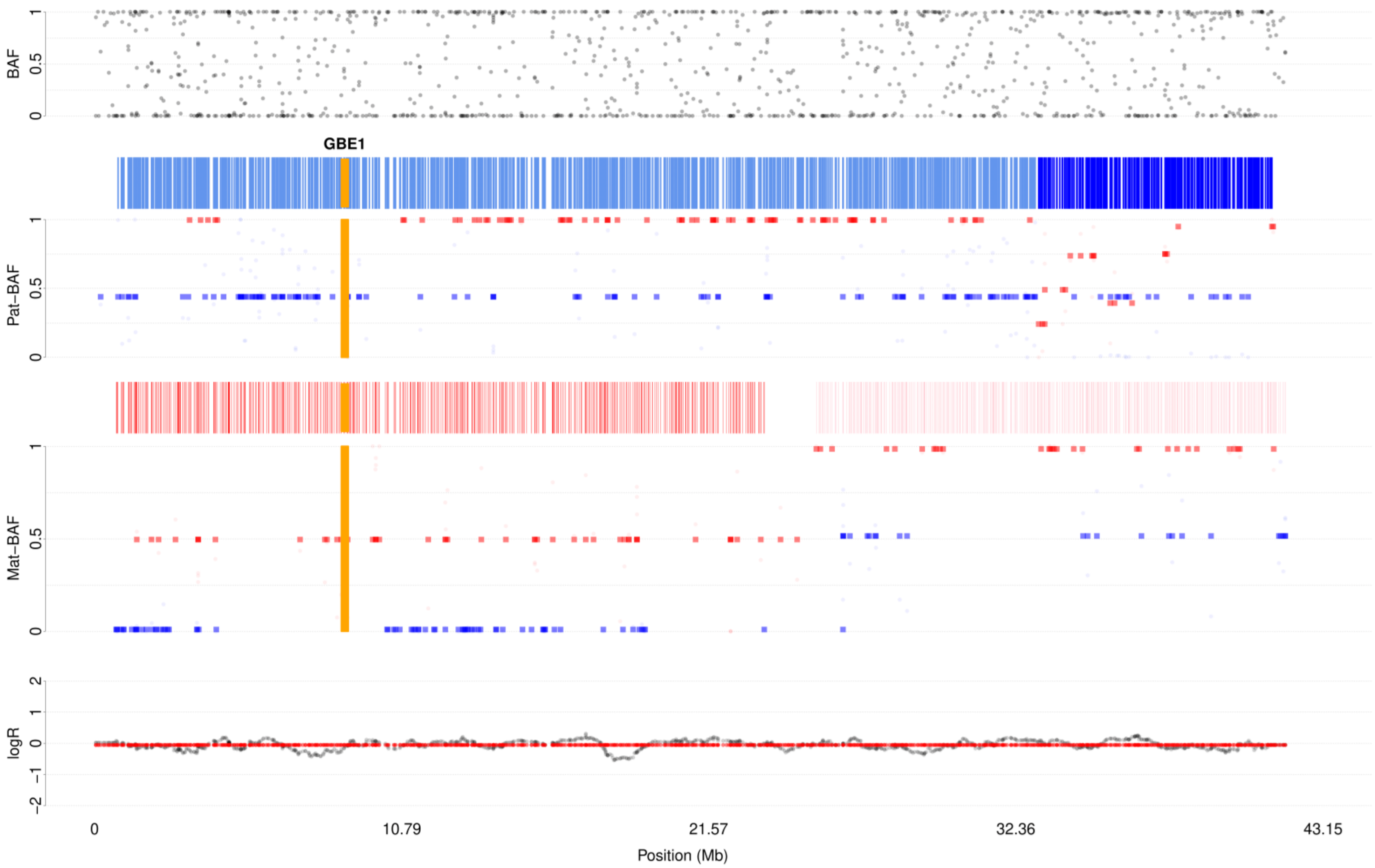

Mare02_Embryo07_Embryo_Chromosome26

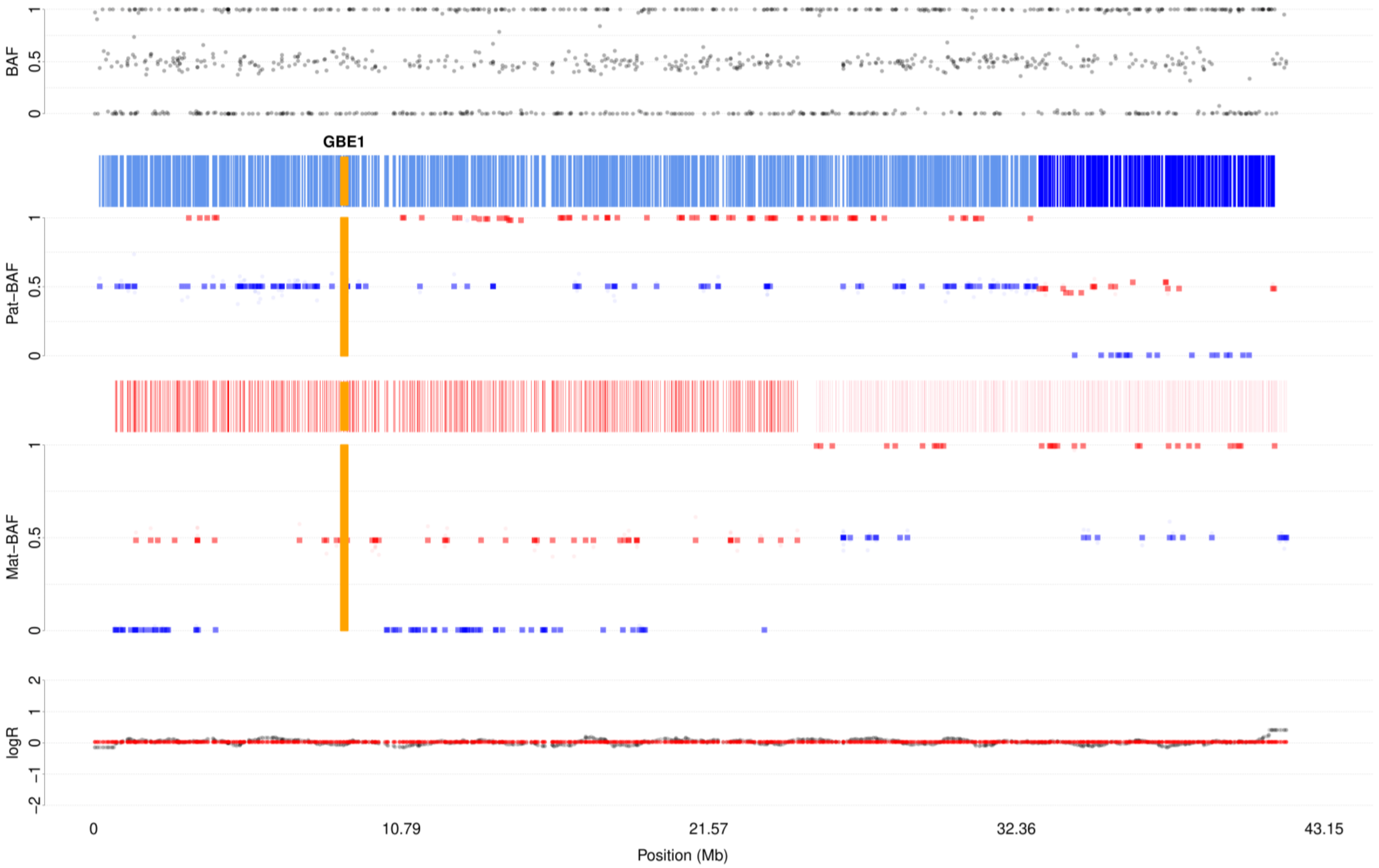

Mare03_Embryo01_Biopsy_Chromosome01

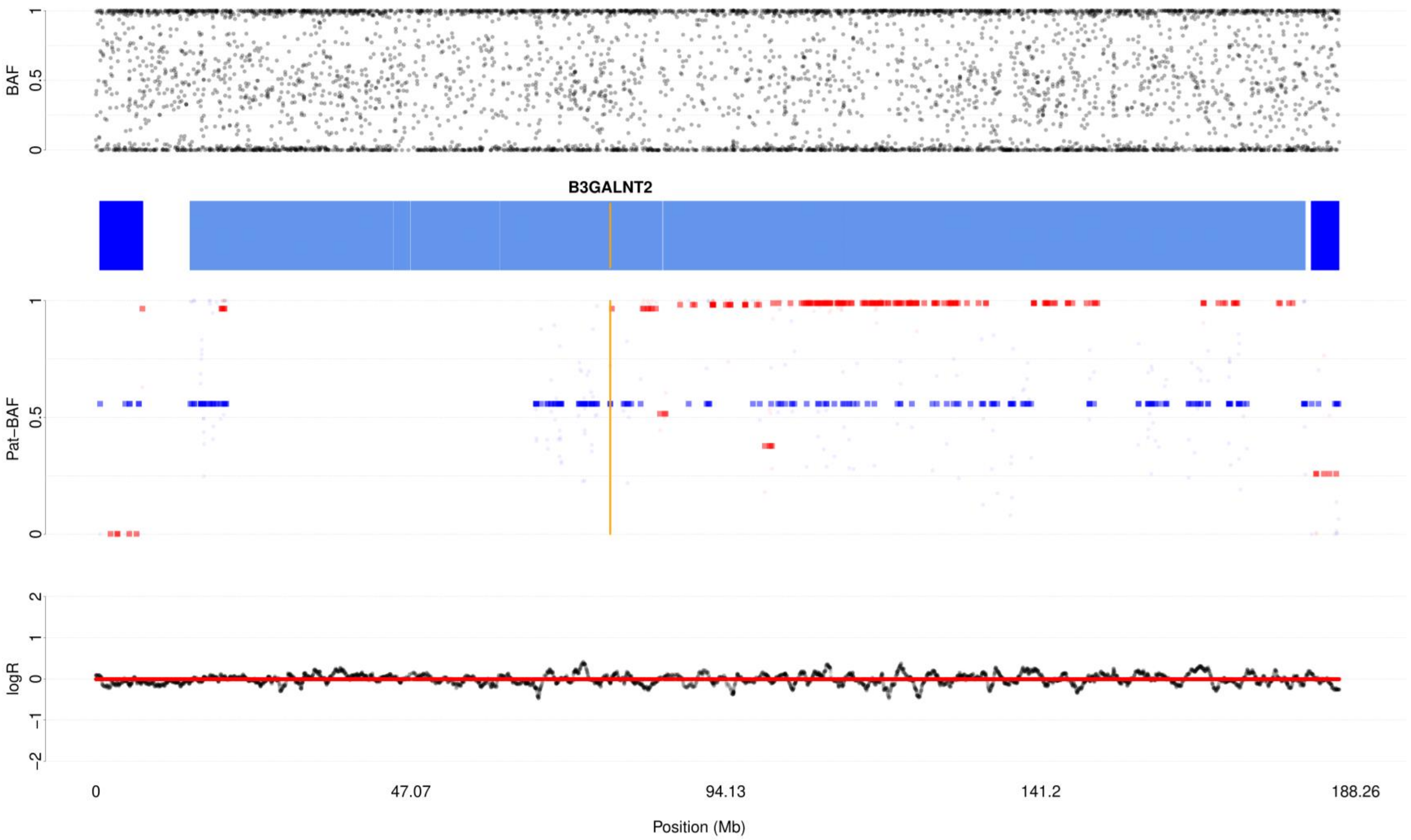

Mare03_Embryo01_Embryo_Chromosome01

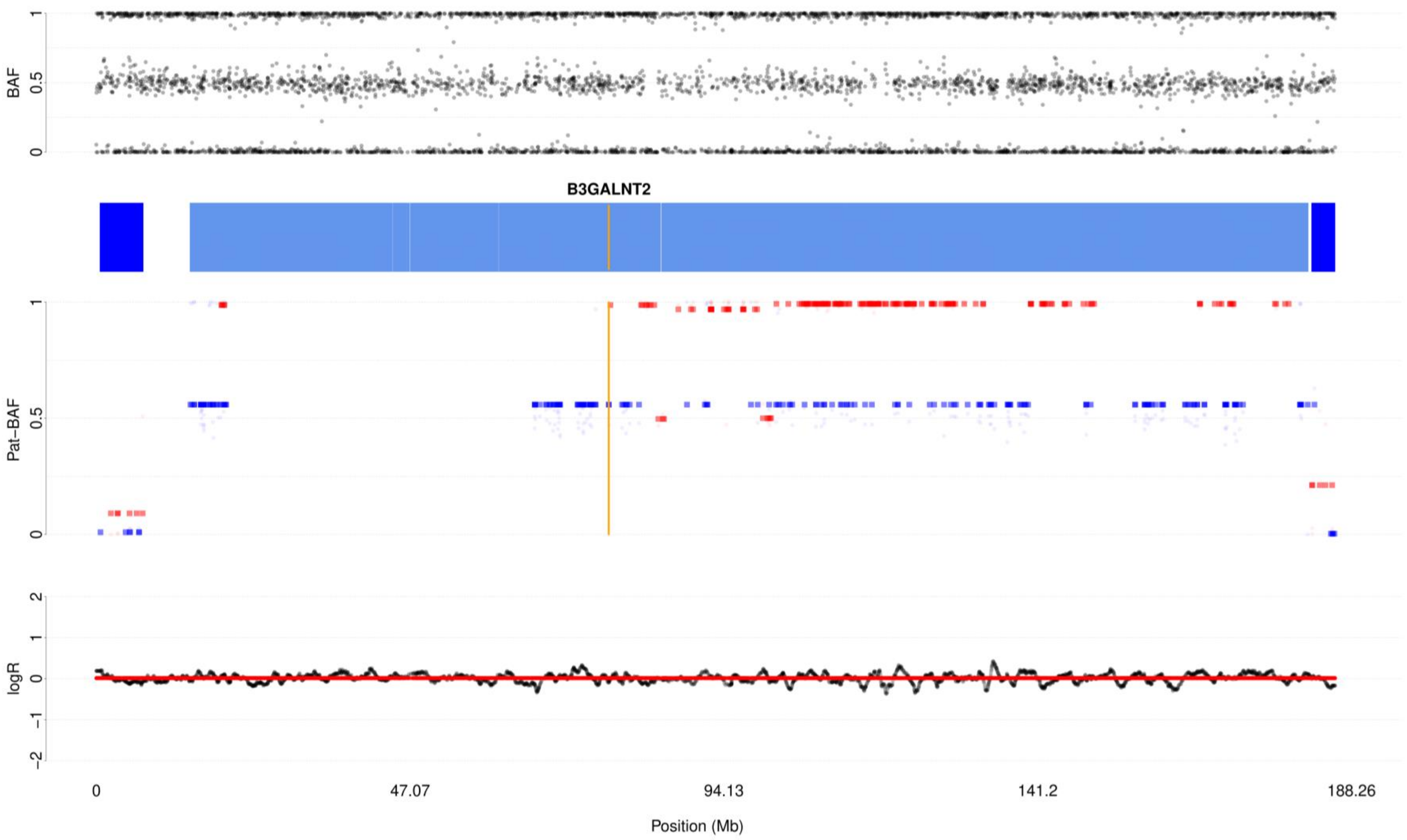

Mare03_Embryo01_Biopsy_Chromosome02

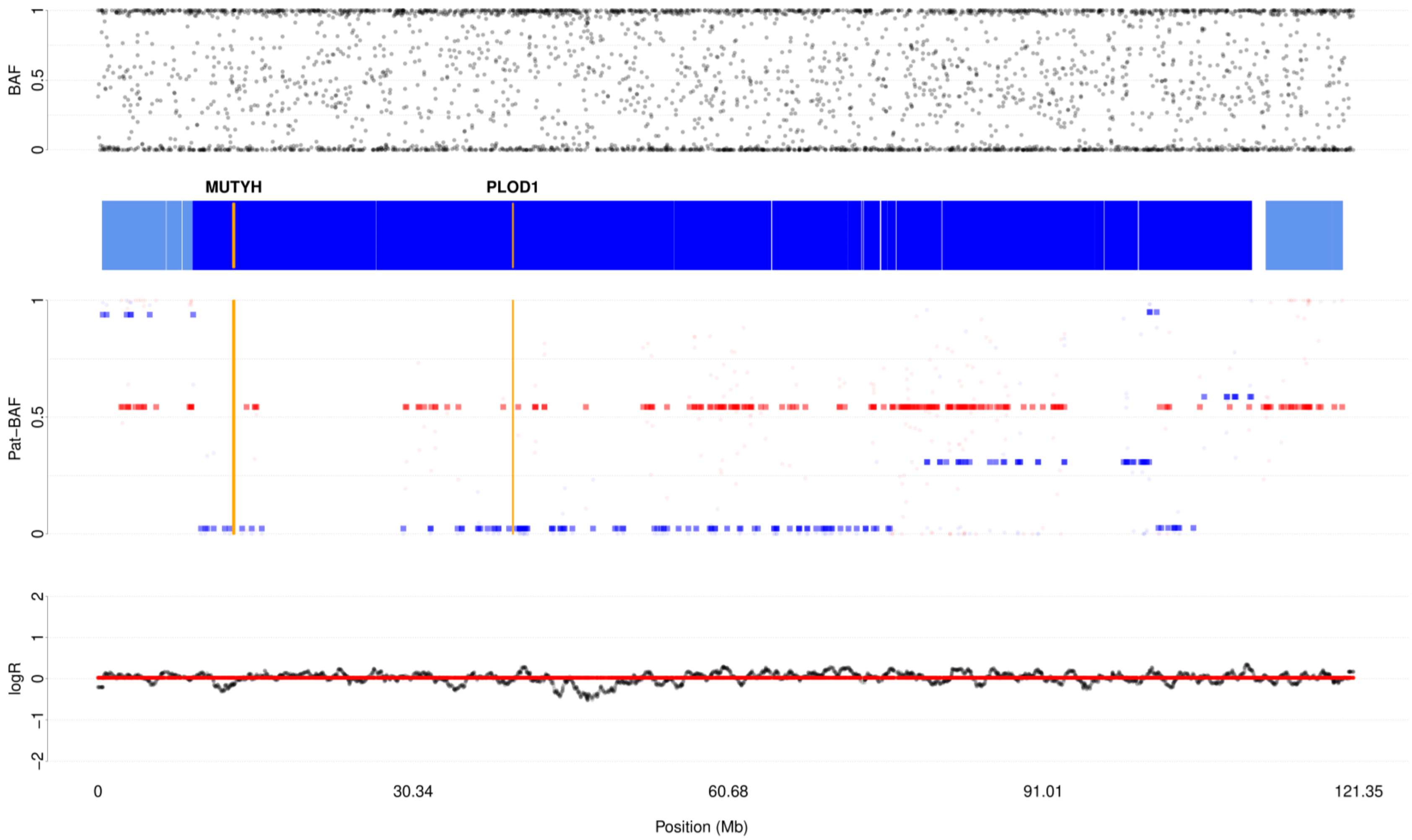

Mare03_Embryo01_Embryo_Chromosome02

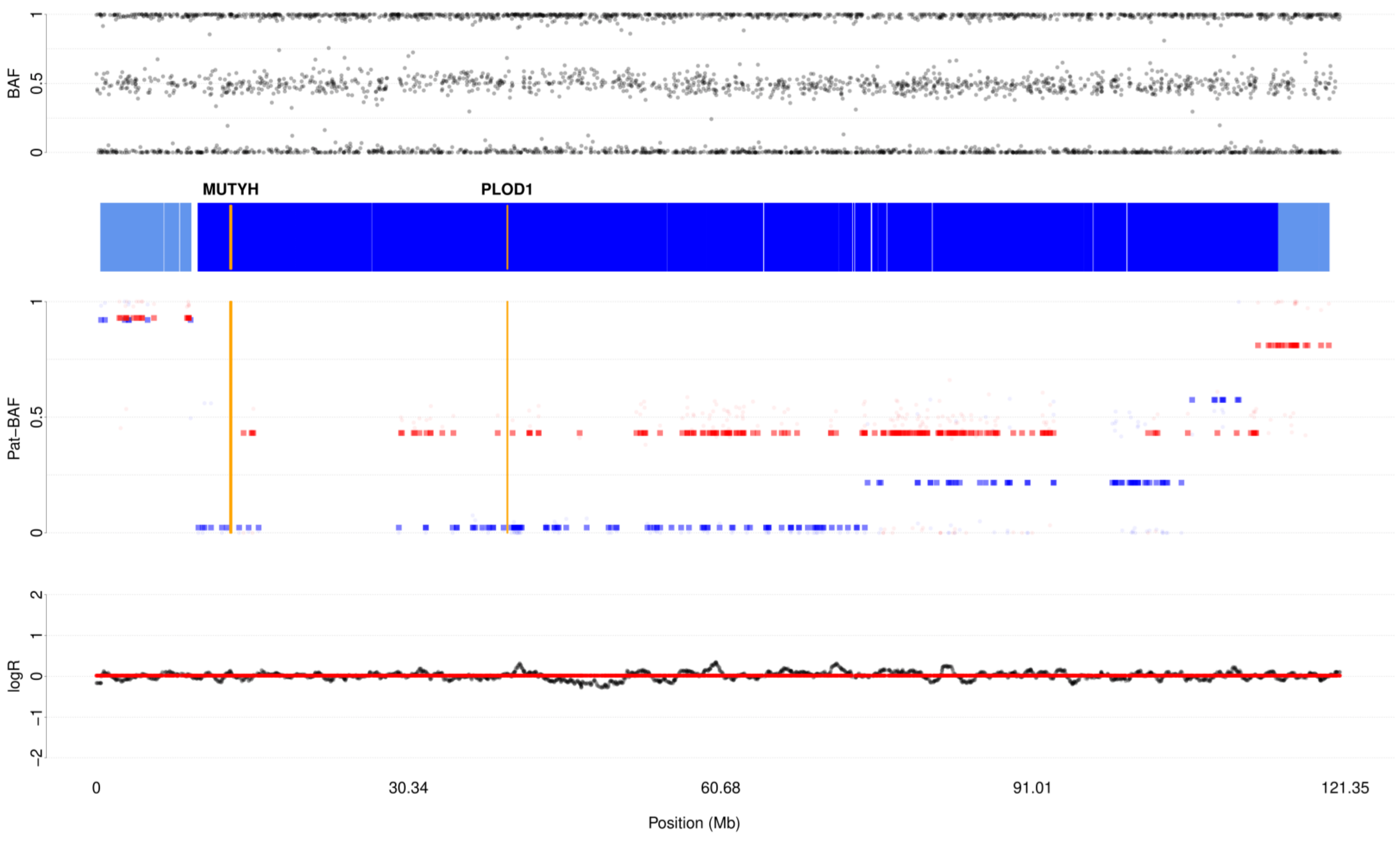

Mare03_Embryo01_Biopsy_Chromosome25

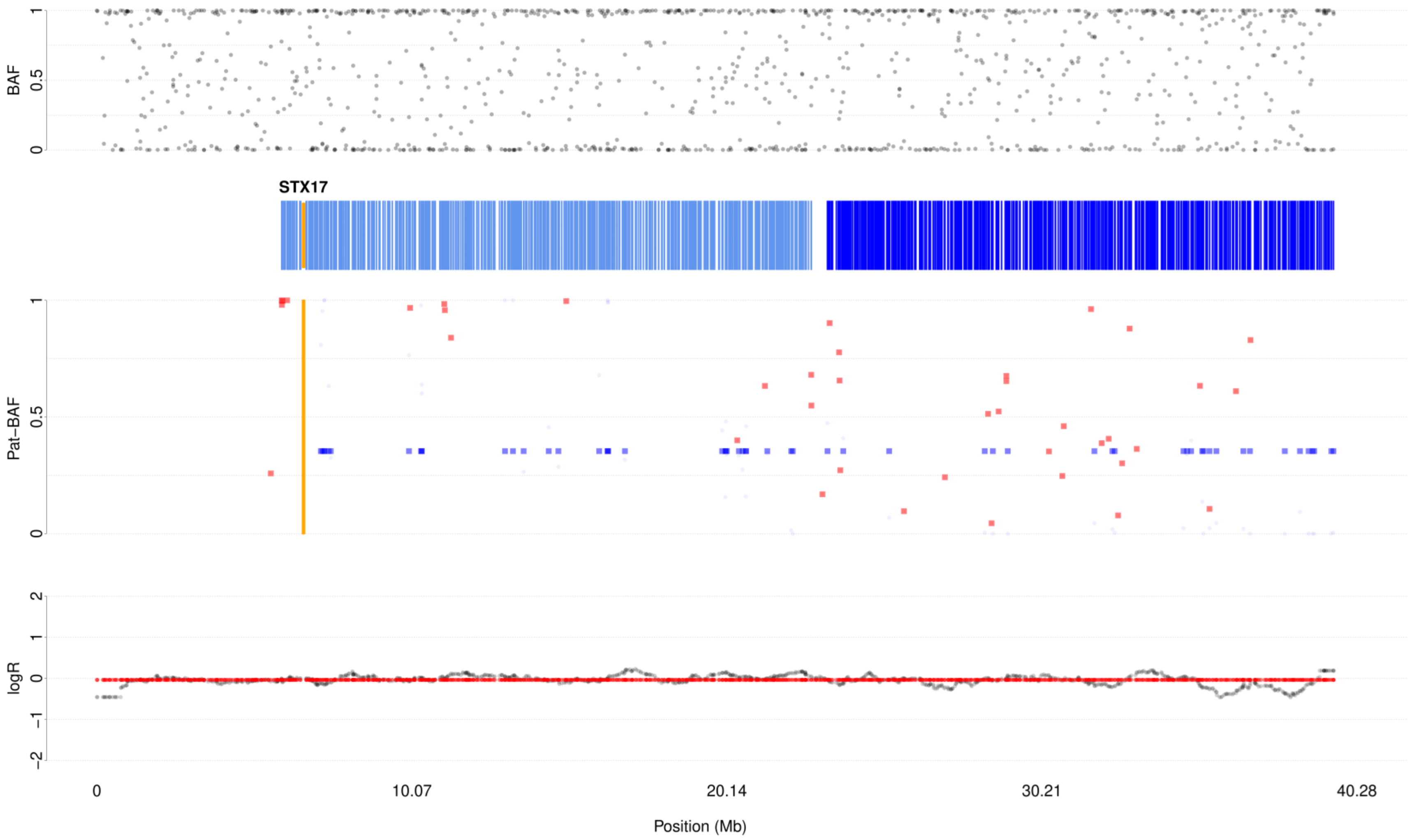

Mare03_Embryo01_Embryo_Chromosome25

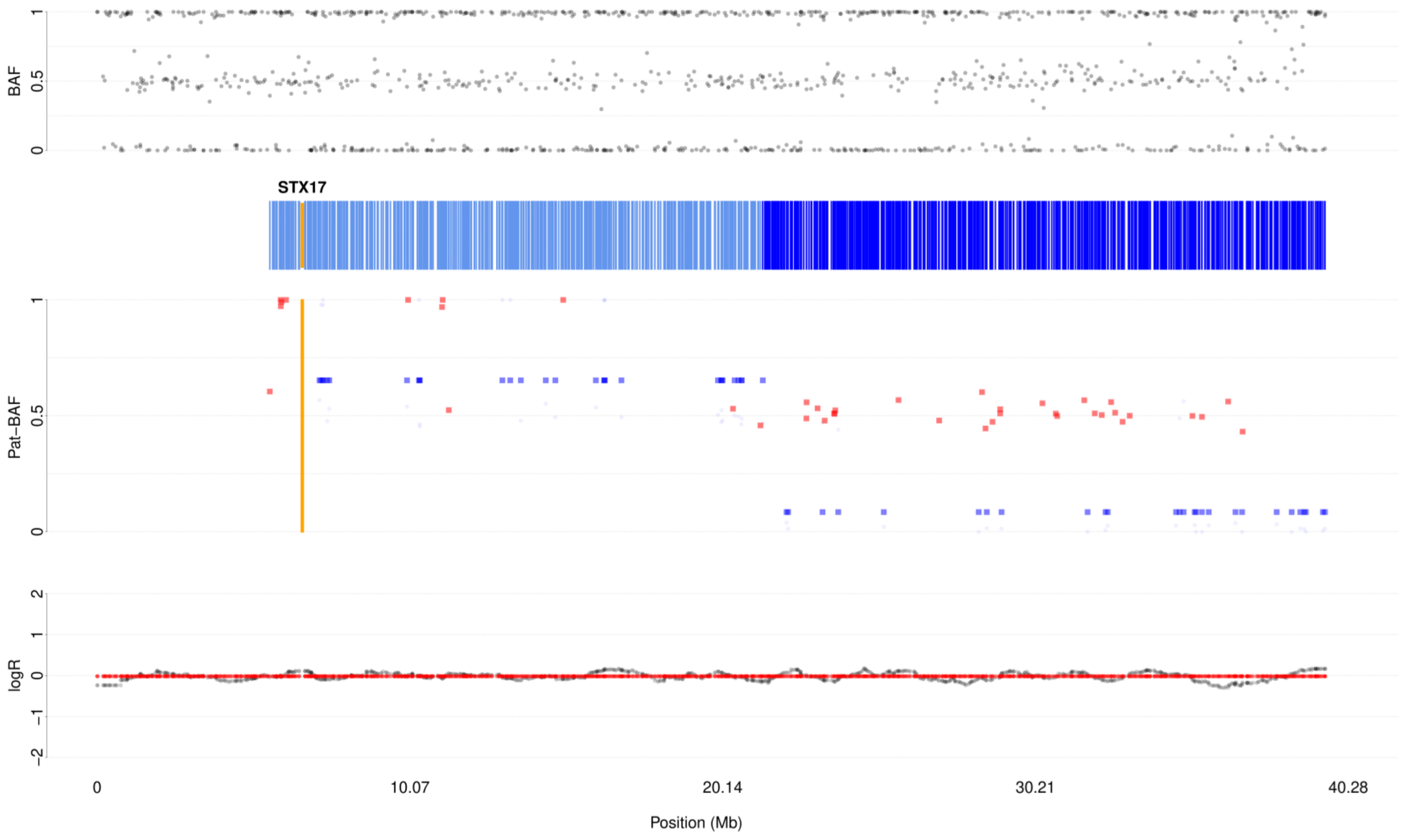

Mare03_Embryo01_Biopsy_Chromosome26

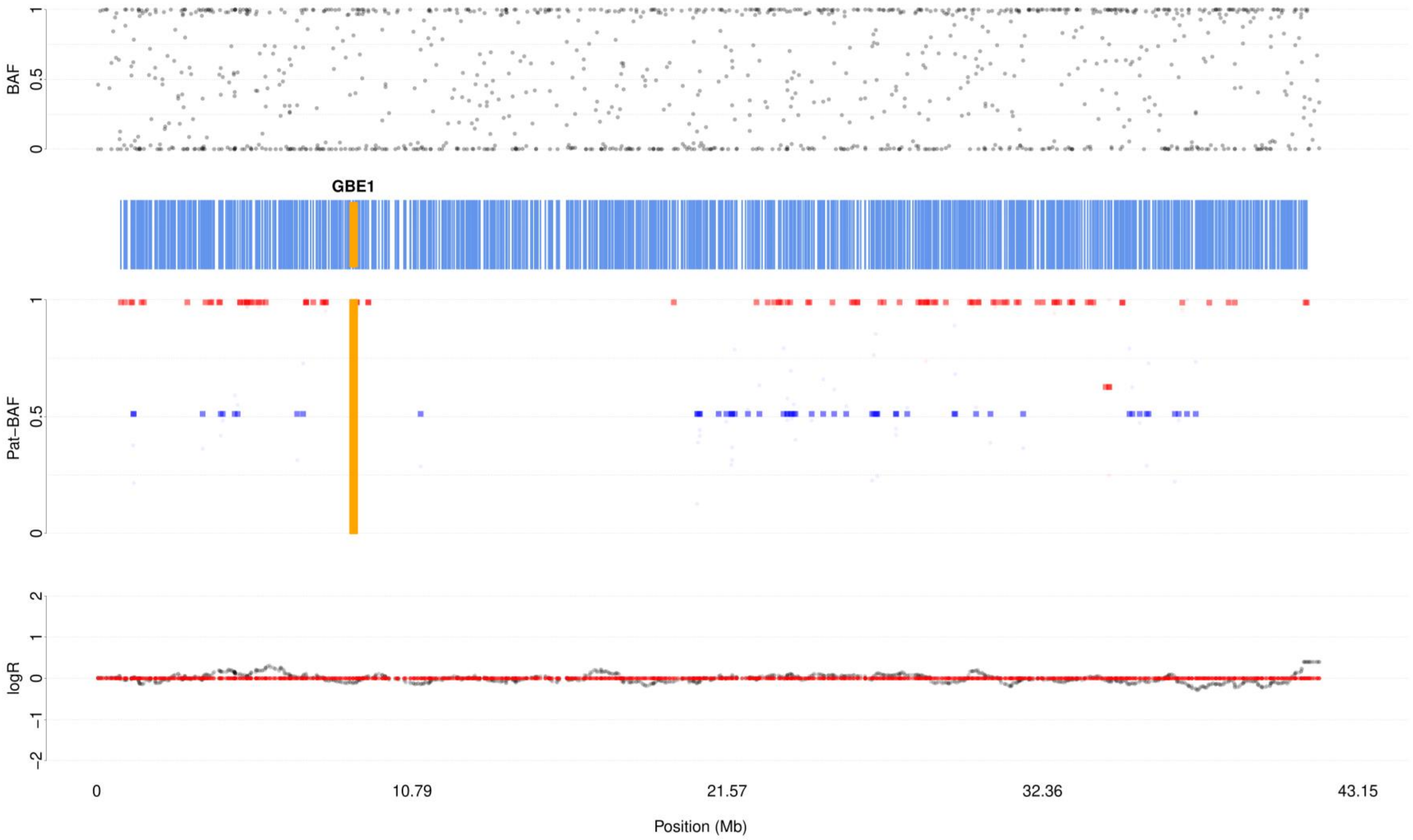

Mare03_Embryo01_Embryo_Chromosome26

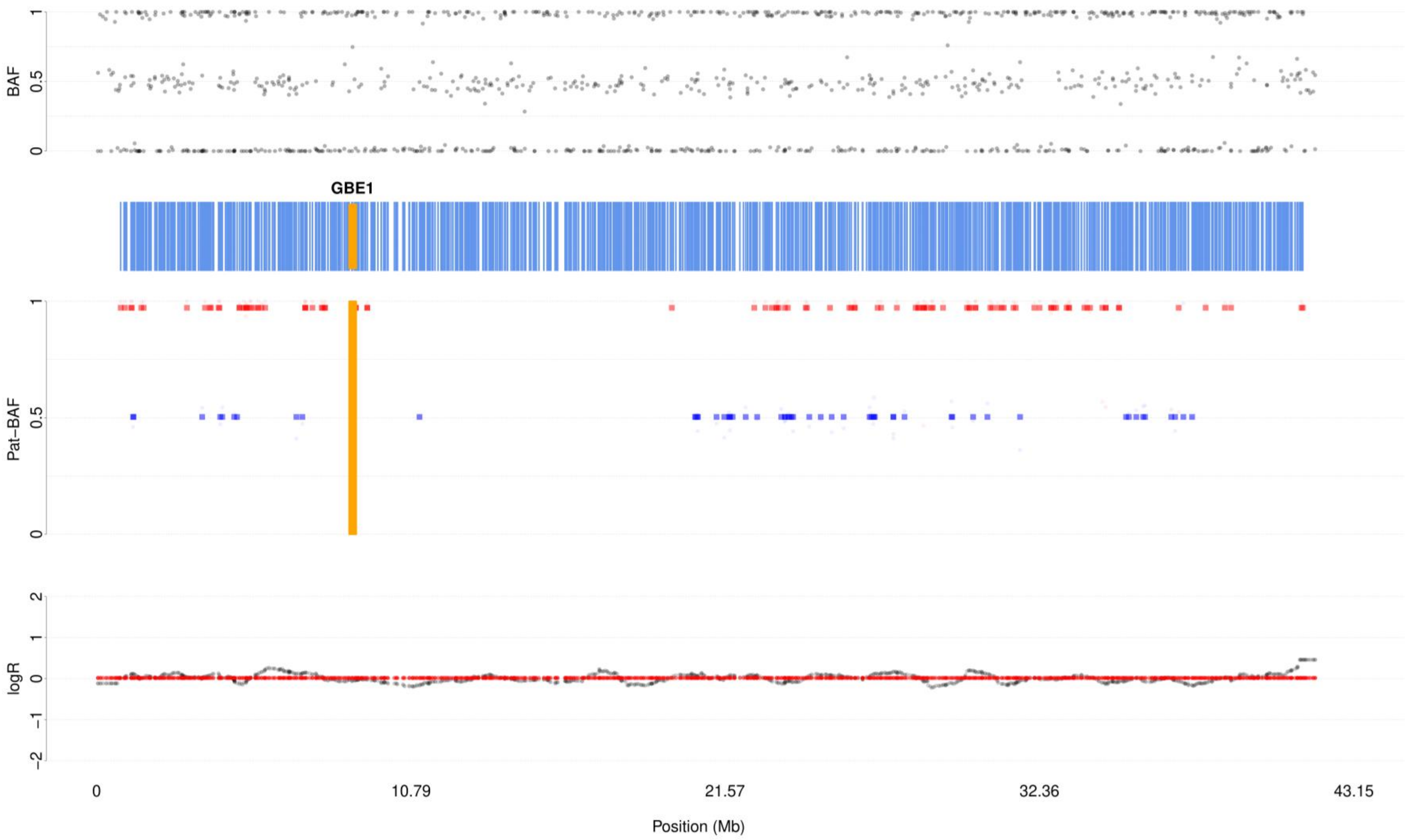

Supplement: Supplementary file 1 — Supplementary Information 1. [file 41598_2023_48103_MOESM1_ESM.pdf]
