# Supplementary material for: Genome-wide equine preimplantation genetic testing enabled by simultaneous haplotyping and copy number detection
Source: Sci Rep. 2024 Jan 23;14:2003. doi: 10.1038/s41598-023-48103-7 (PMC10805710; doi:10.1038/s41598-023-48103-7)

**ADDITIONAL FILE 2:** DATA OVERVIEW OF THE BLASTOMERE SAMPLES FROM ARRESTED CLEAVAGE-STAGE EMBRYOS

Table 1. Characteristics of the blastomere samples, concentration after WGA and coverage.

Figure 1. Pictures of the arrested cleavage-stage embryos and the blastomere samples and haplarithm plots of the blastomere samples.

**Table 1.** Characteristics of the blastomere samples, concentration after WGA and coverage.

| Sample | Time of collection (day post ICSI) | Concentration after WGA (ng/µL) | Coverage of analyzed SNP genotypes (%) | Sex |
|---|---|---|---|---|
| Mare01_ Embryo01_Cell1 | 11 | 432 | 45.14* | ♀ |
| Mare01_ Embryo01_Cell2 | 11 | 348 | 39.49* | ♀ |
| Mare01_ Embryo01_Cell3 | 11 | 364 | 45.54* | ♀ |
| Mare01_ Embryo01_Cell4 | 11 | Lost during collection | | ♀ |
| Mare01_ Embryo01_Cell5 | 11 | 372 | 35.80* | ♀ |
| Mare01_ Embryo02_Cell1 | 11 | 378 | 43.62* | ♀ |
| Mare01_ Embryo02_Cell2 | 11 | 412 | 46.92* | ♀ |
| Mare01_ Embryo02_Cell3 | 11 | Lost during collection | | ♀ |
| Mare01_ Embryo02_Cell4 | 11 | 316 | 61.29 | ♀ |
| Mare01_ Embryo02_Cell5 | 11 | 174 | 50.97 | ♀ |
| Mare01_ Embryo02_Cell6 | 11 | 222 | 60.75 | ♀ |
| Mare01_ Embryo02_Cell7 | 11 | 392 | 42.20* | ♀ |
| Mare01_ Embryo02_Cell8 | 11 | 406 | 49.39* | ♀ |
| Mare02_ Embryo01_Cell1 | 11 | 390 | 50.30* | ♀ |
| Mare02_ Embryo01_Cell2 | 11 | 374 | 47.50* | ♀ |
| Mare02_ Embryo01_Cell3 | 11 | 364 | 48.69* | ♀ |
| Mare02_ Embryo02_Cell1 | 11 | 338 | 45.73* | ♂ |
| Mare02_ Embryo02_Cell2 | 11 | 282 | 44.60* | ♂ |
| Mare02_ Embryo02_Cell3 | 11 | 416 | 44.48* | ♂ |
| Mare02_ Embryo02_Cell4 | 11 | 388 | 47.56* | ♂ |
| Mare02_ Embryo03_Cell1 | 11 | 426 | 47.31* | ♀ |
| Mare02_ Embryo03_Cell2 | 11 | 308 | 45.53* | ♀ |
| Mare02_ Embryo03_Cell3 | 11 | 364 | 47.57* | ♀ |
| Mare02_ Embryo03_Cell4 | 11 | 90.4 | 62.20 | ♀ |
| Mare02_ Embryo03_Cell5 | 11 | 280 | 61.91 | ♀ |
| Mare02_ Embryo03_Cell6 | 11 | 76.2 | 54.72 | ♀ |
| Mare04_ Embryo01_Cell1 | 12 | 226 | 58.97 | ♀ |
| Mare04_ Embryo01_Cell2 | 12 | 260 | 41.32* | ♀ |
| **Average coverage (%)** | | | 48.83 | |
| **Average coverage successful (*) samples (%)** | | | 45.19 | |

*Samples successfully analyzed by haplarithmisis. ICSI: intracytoplasmic sperm injection; SNP: single nucleotide polymorphism; WGA: whole genome amplification.

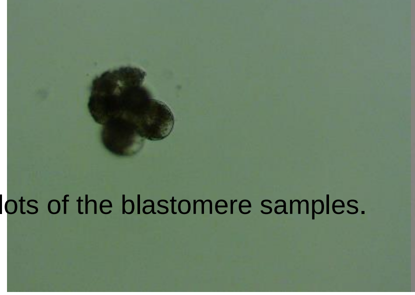

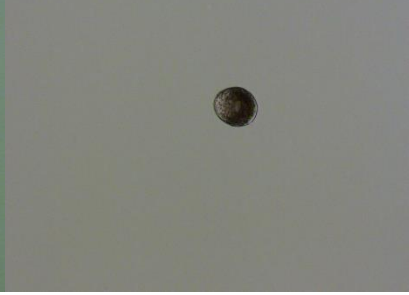

**Figure 1.** Pictures of the arrested cleavage-stage embryos and the blastomere samples and haplarithm plots of the blastomere samples.

Mare01_ Embryo01_Cell1

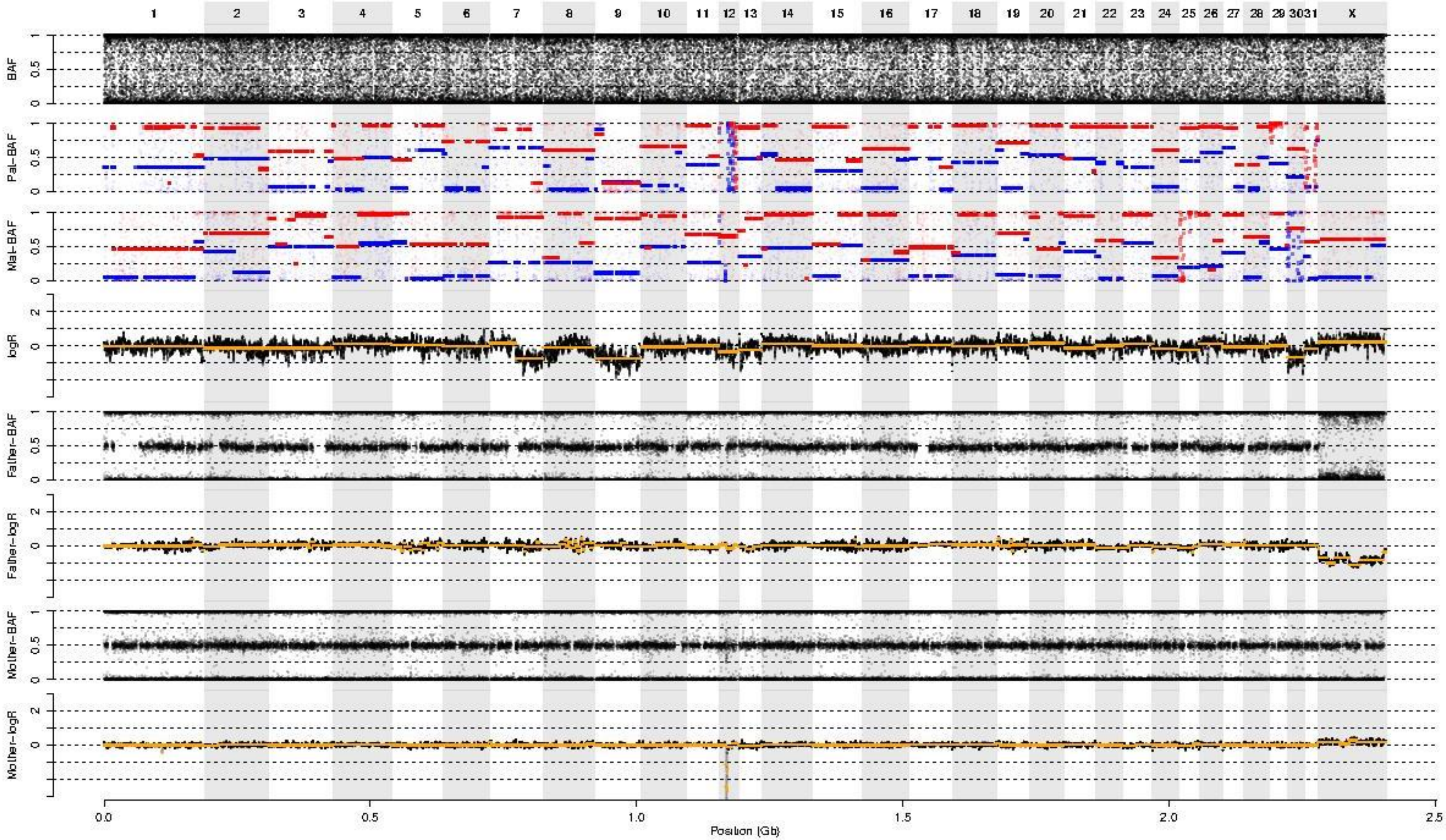

Mare01_ Embryo01_Cell2

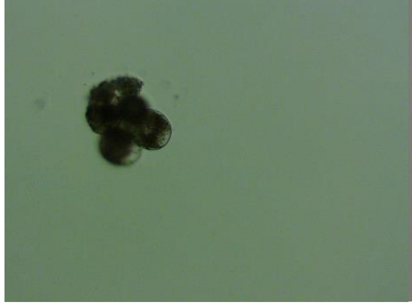

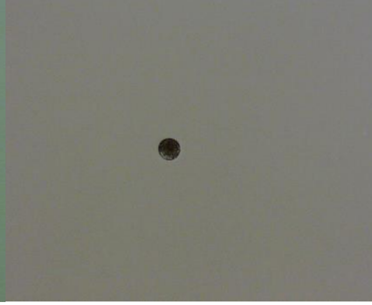

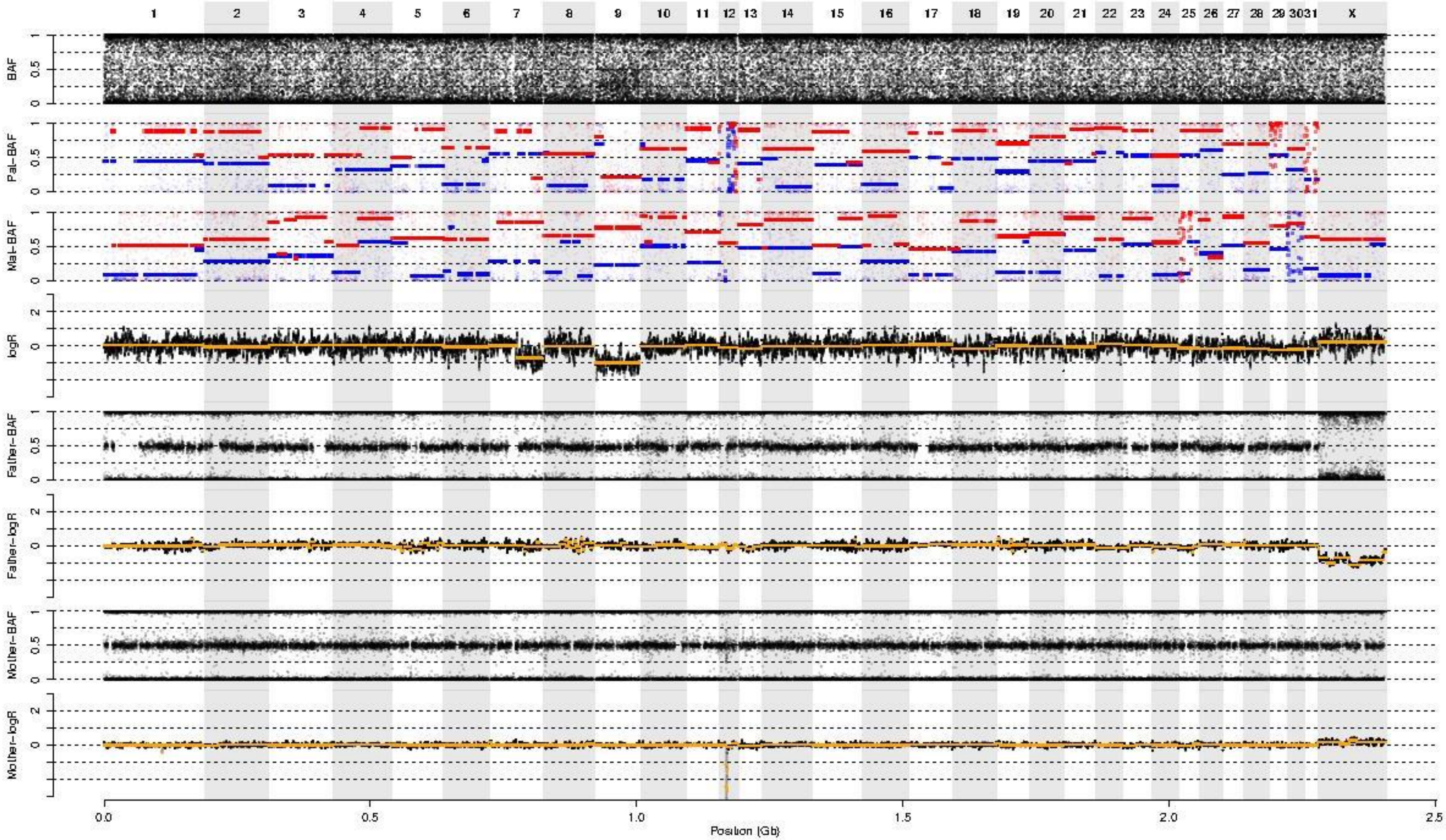

Mare01_ Embryo01_Cell3

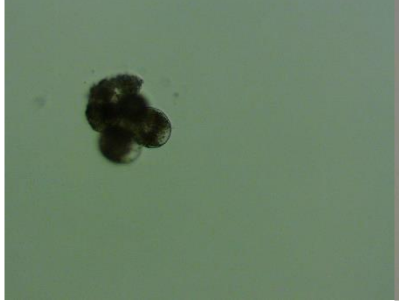

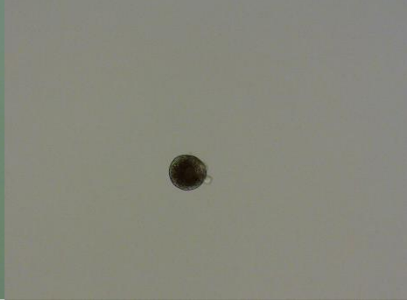

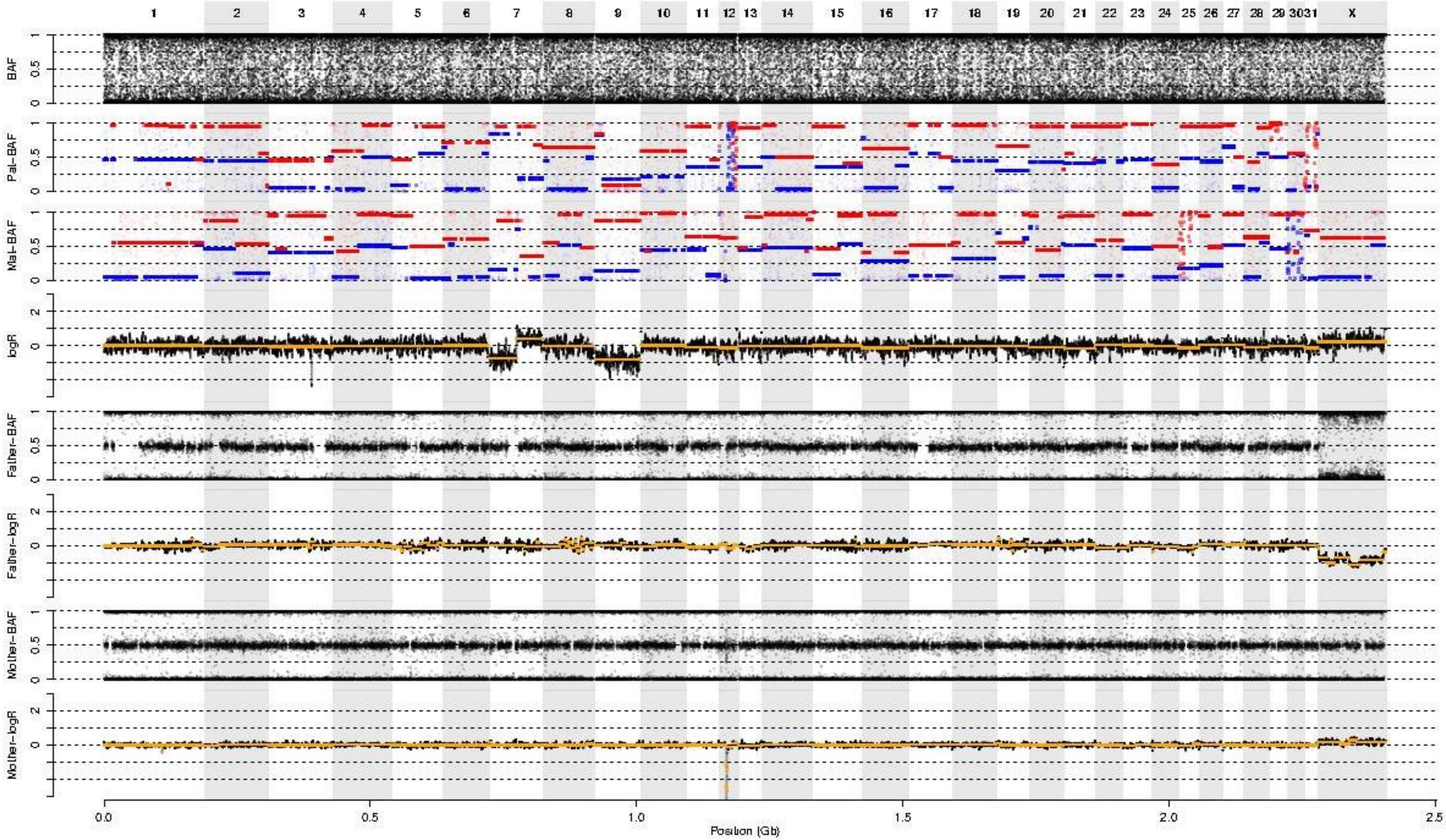

Mare01_ Embryo01_Cell4

Lost during collection.

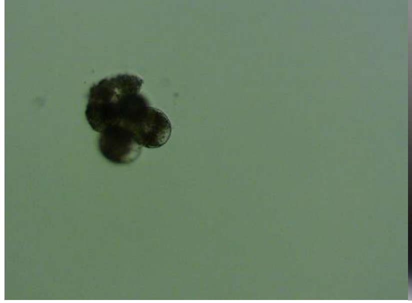

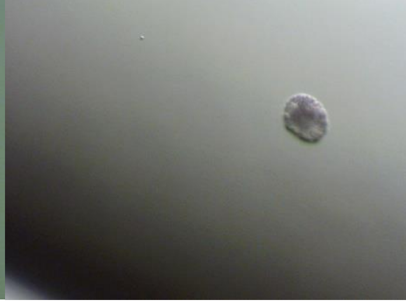

Mare01_ Embryo01_Cell5

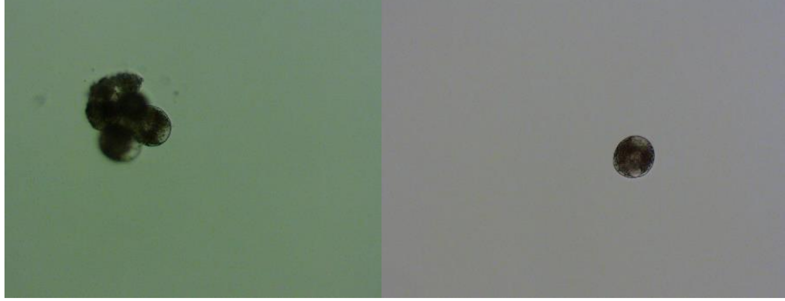

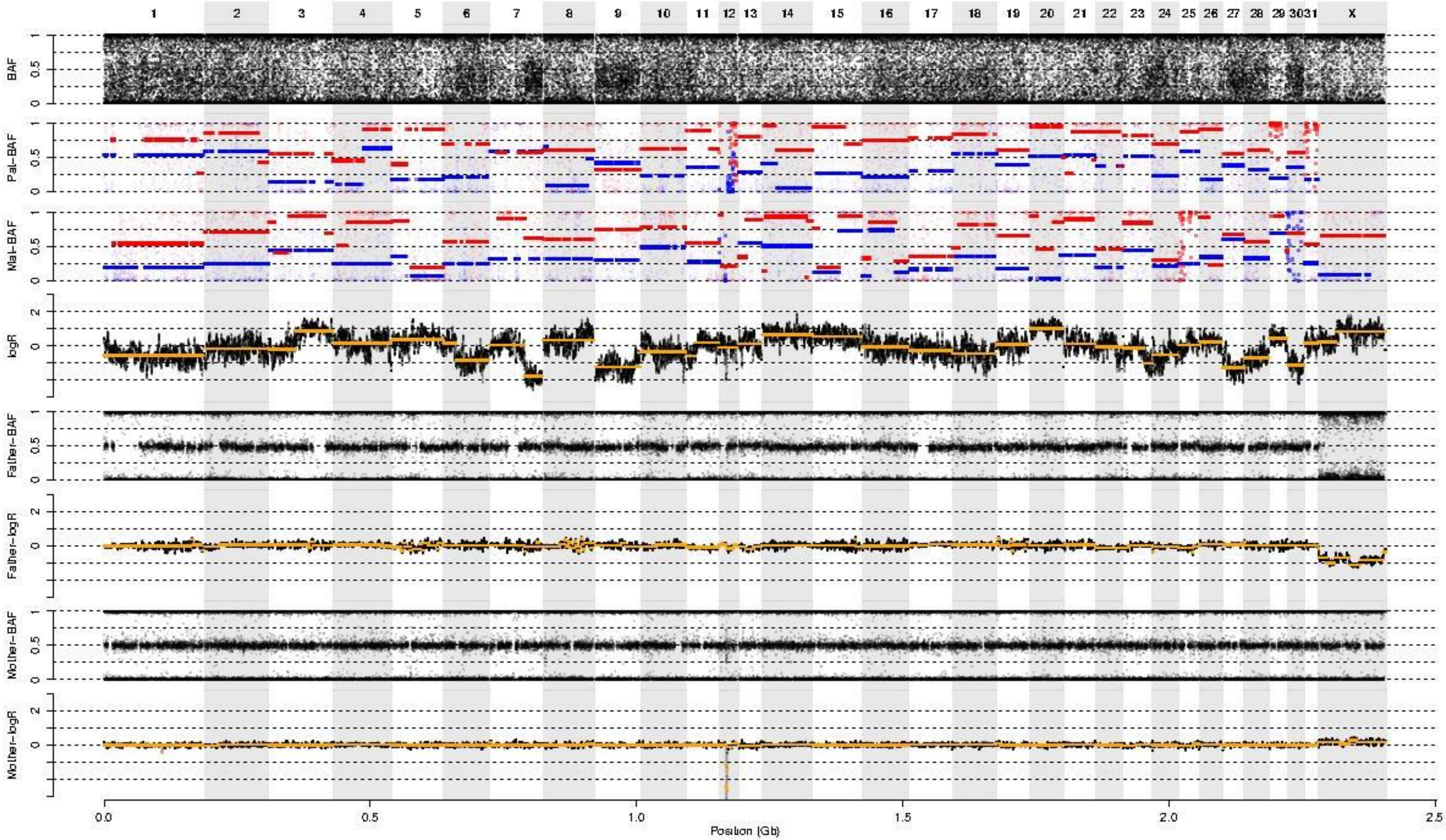

Mare01_ Embryo02_Cell1

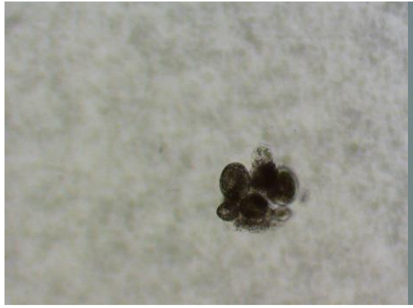

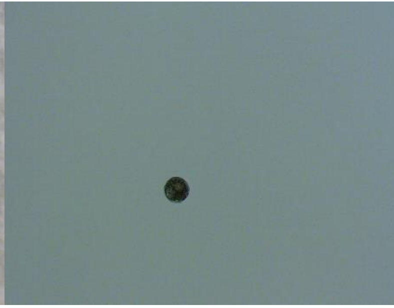

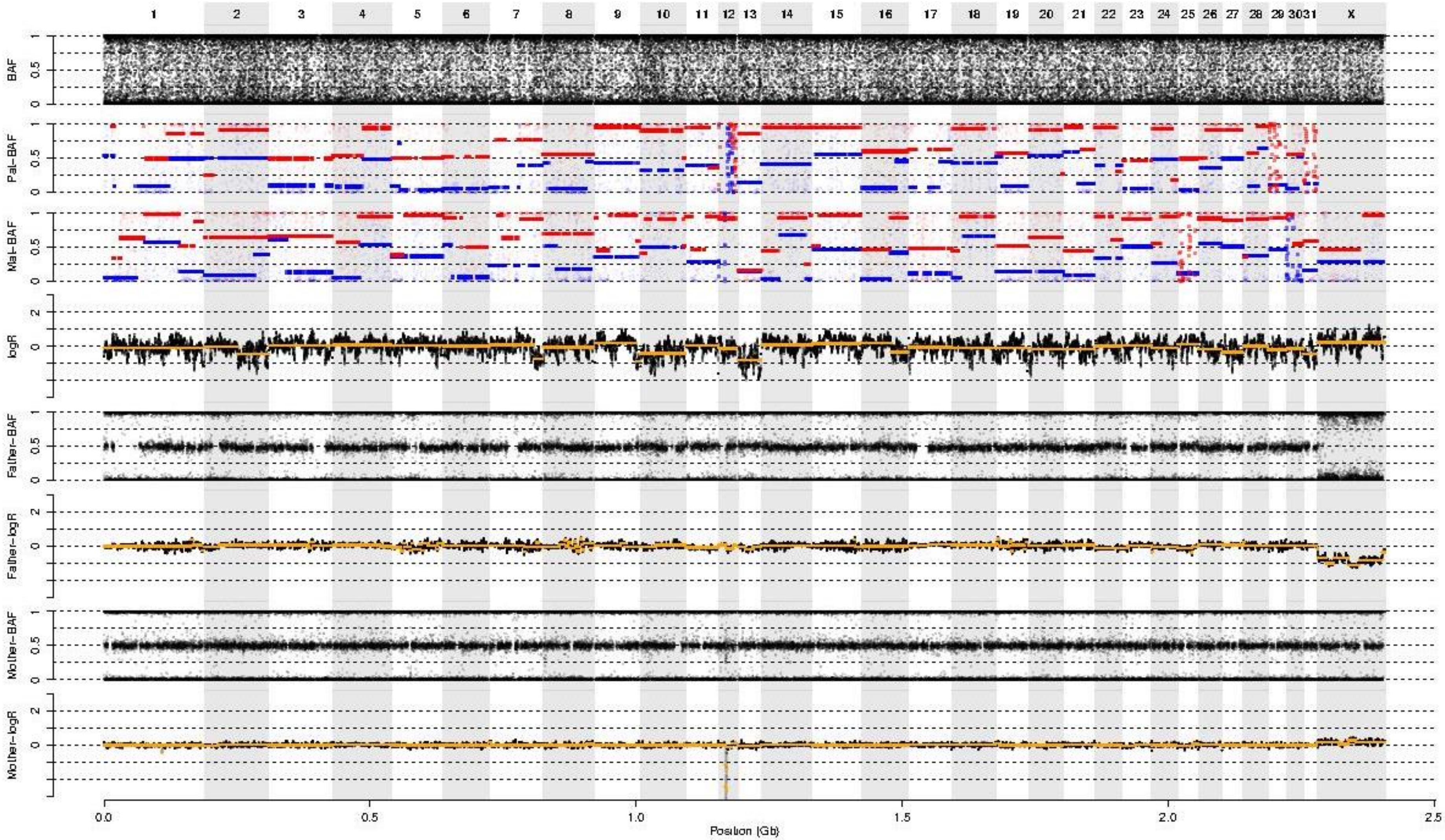

Mare01_ Embryo02_Cell2

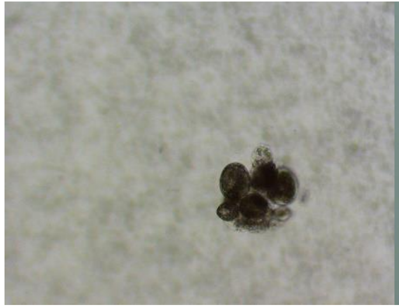

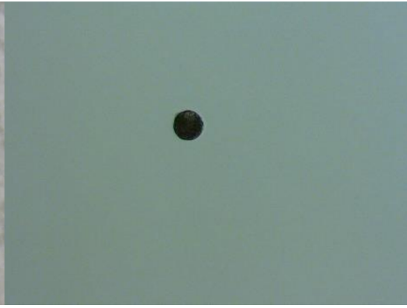

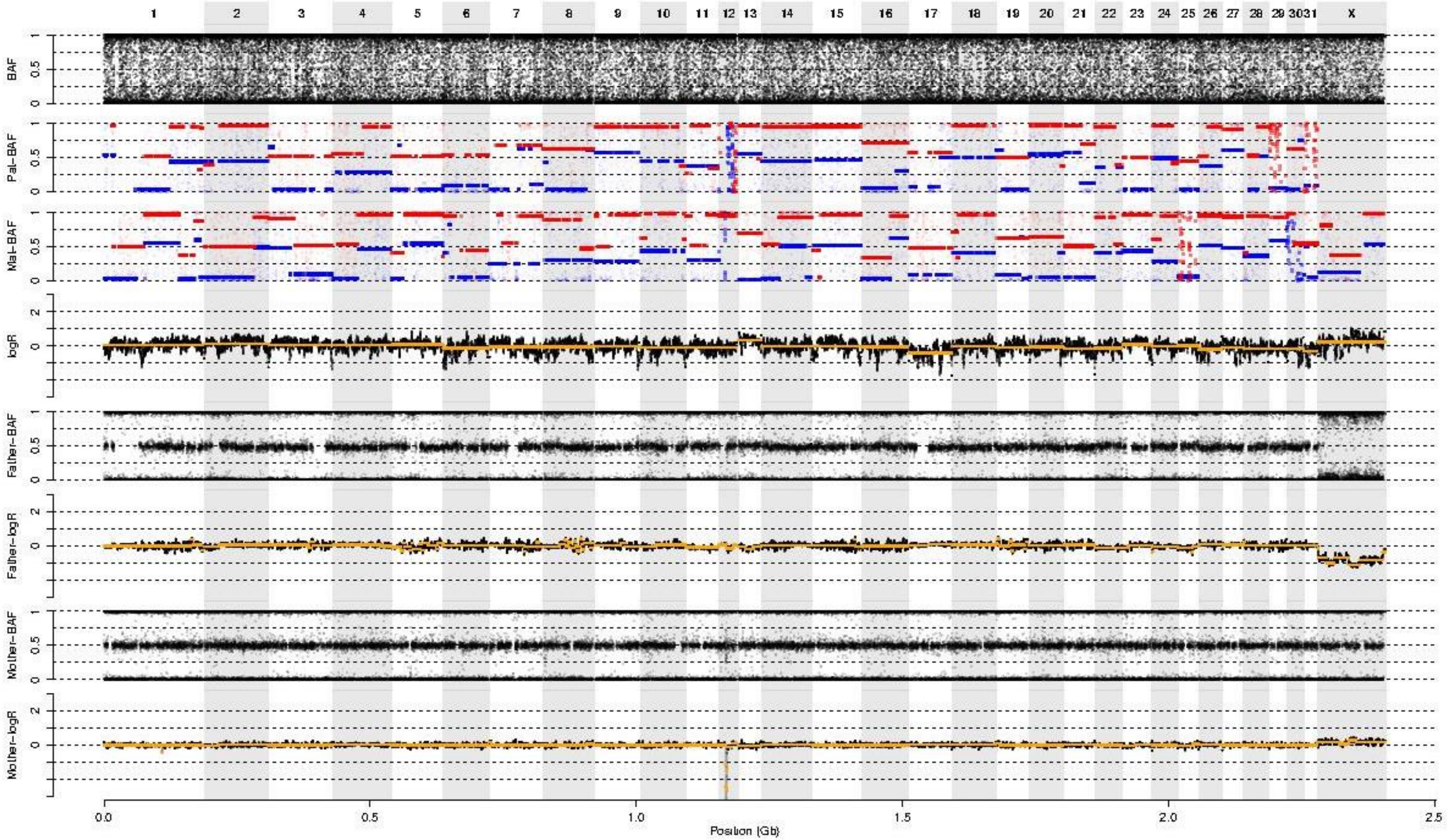

Mare01_ Embryo02_Cell3

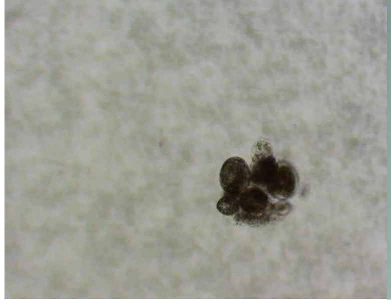

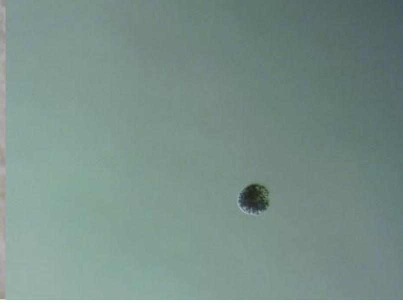

Lost during collection.

Mare01_ Embryo02_Cell4

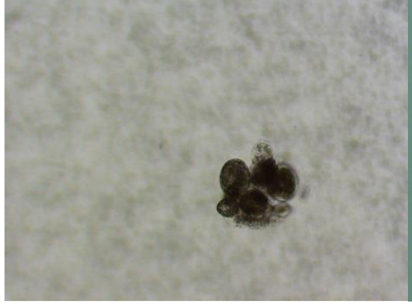

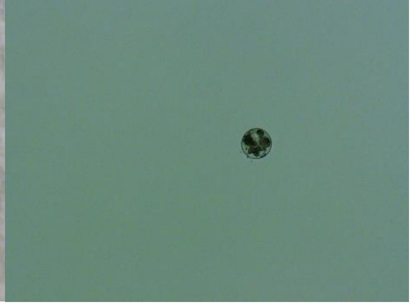

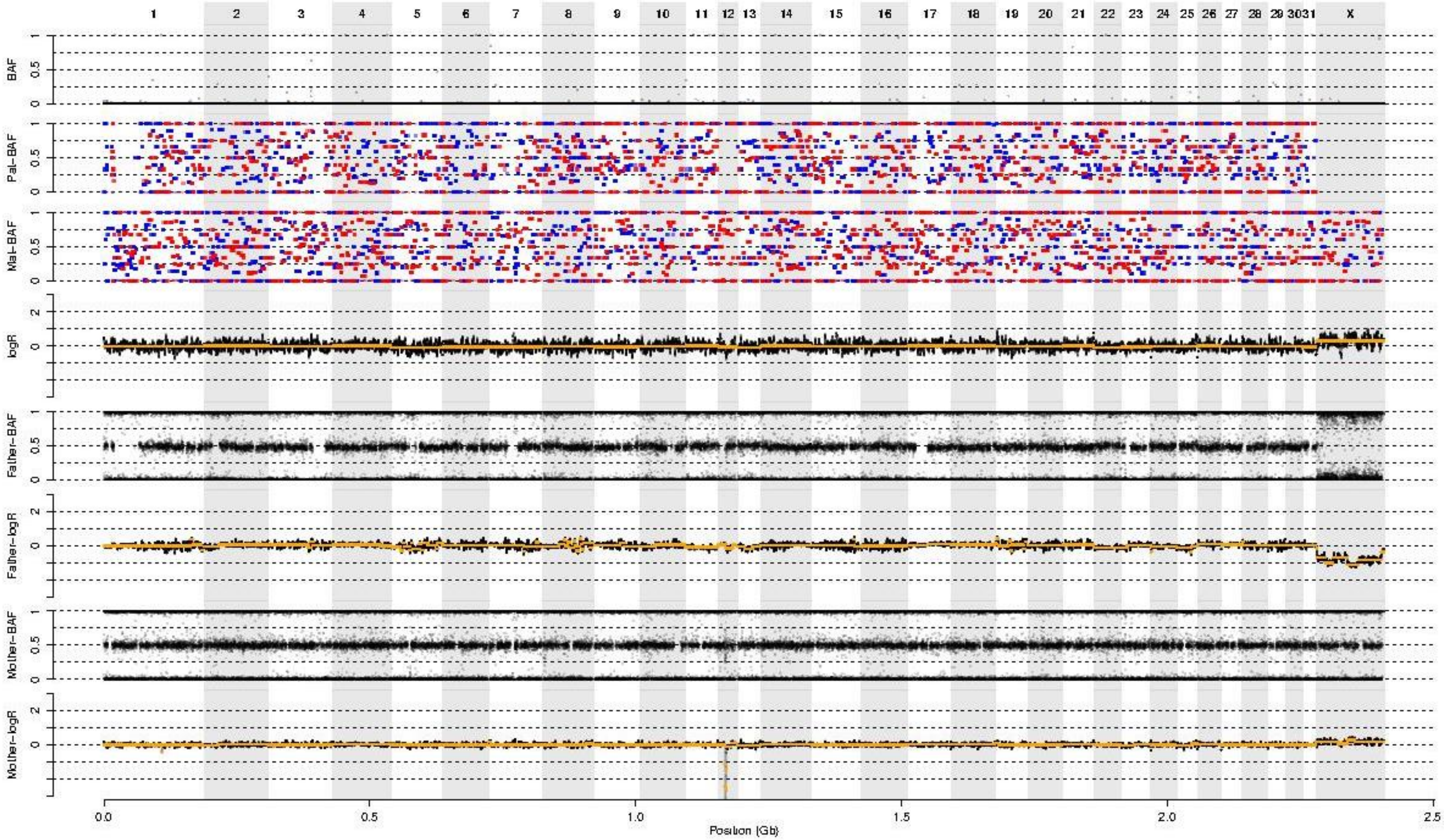

Mare01_ Embryo02_Cell5

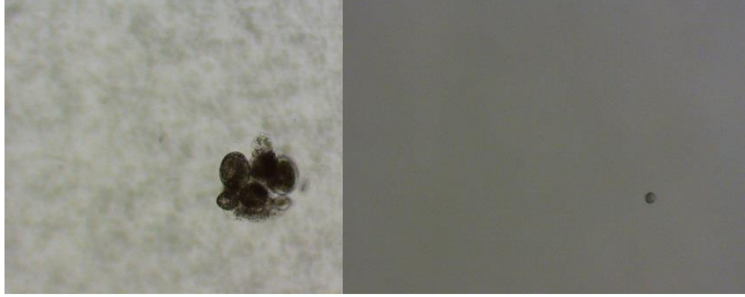

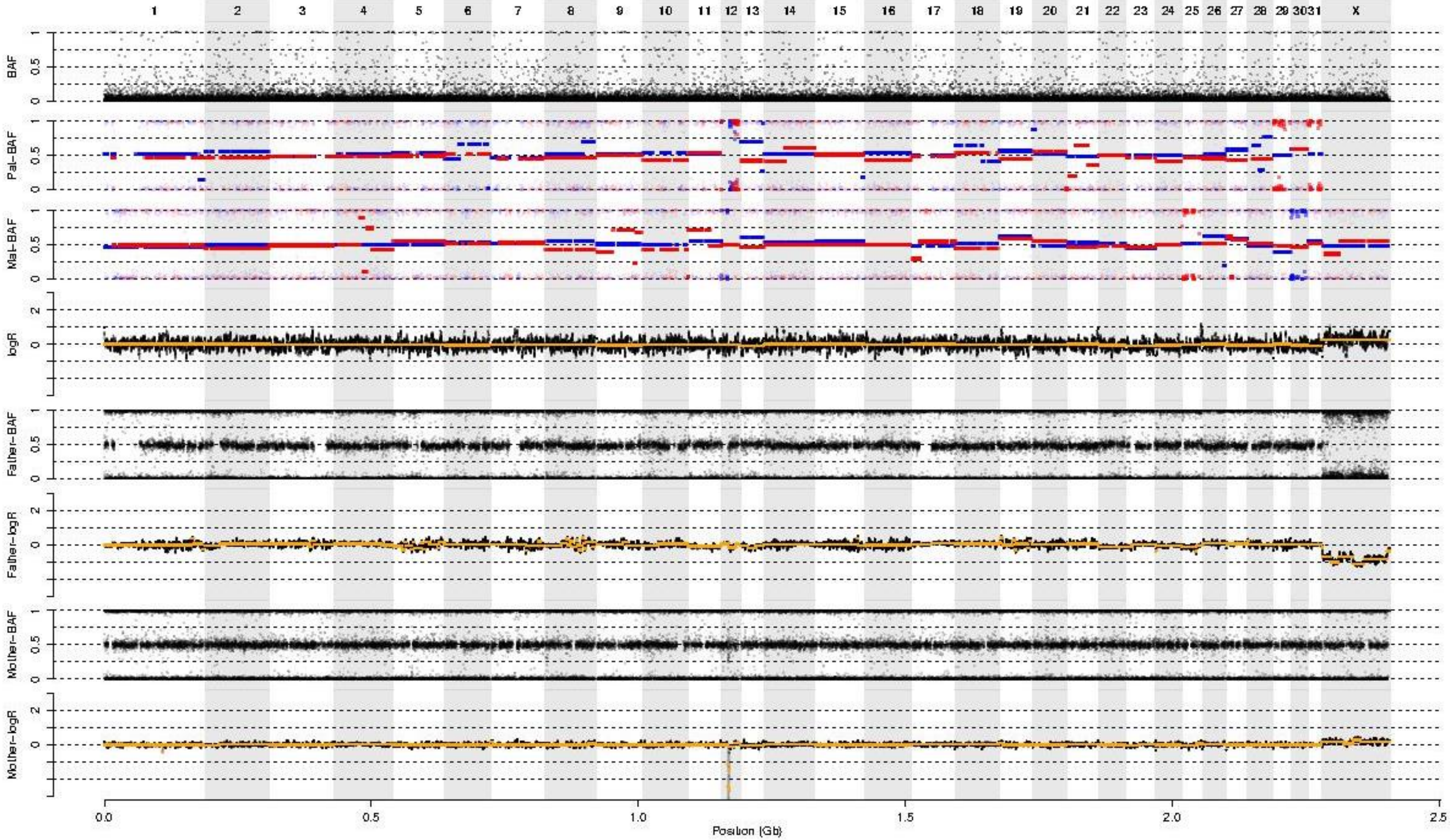

Mare01_ Embryo02_Cell6

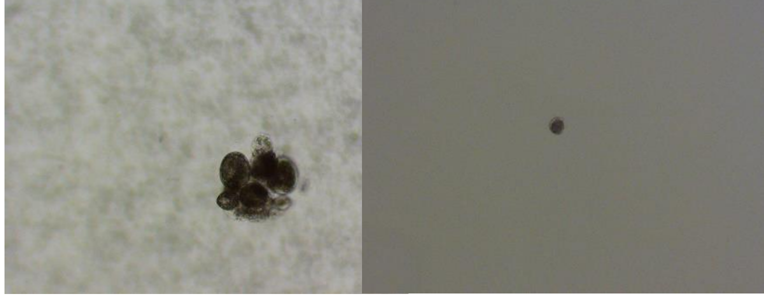

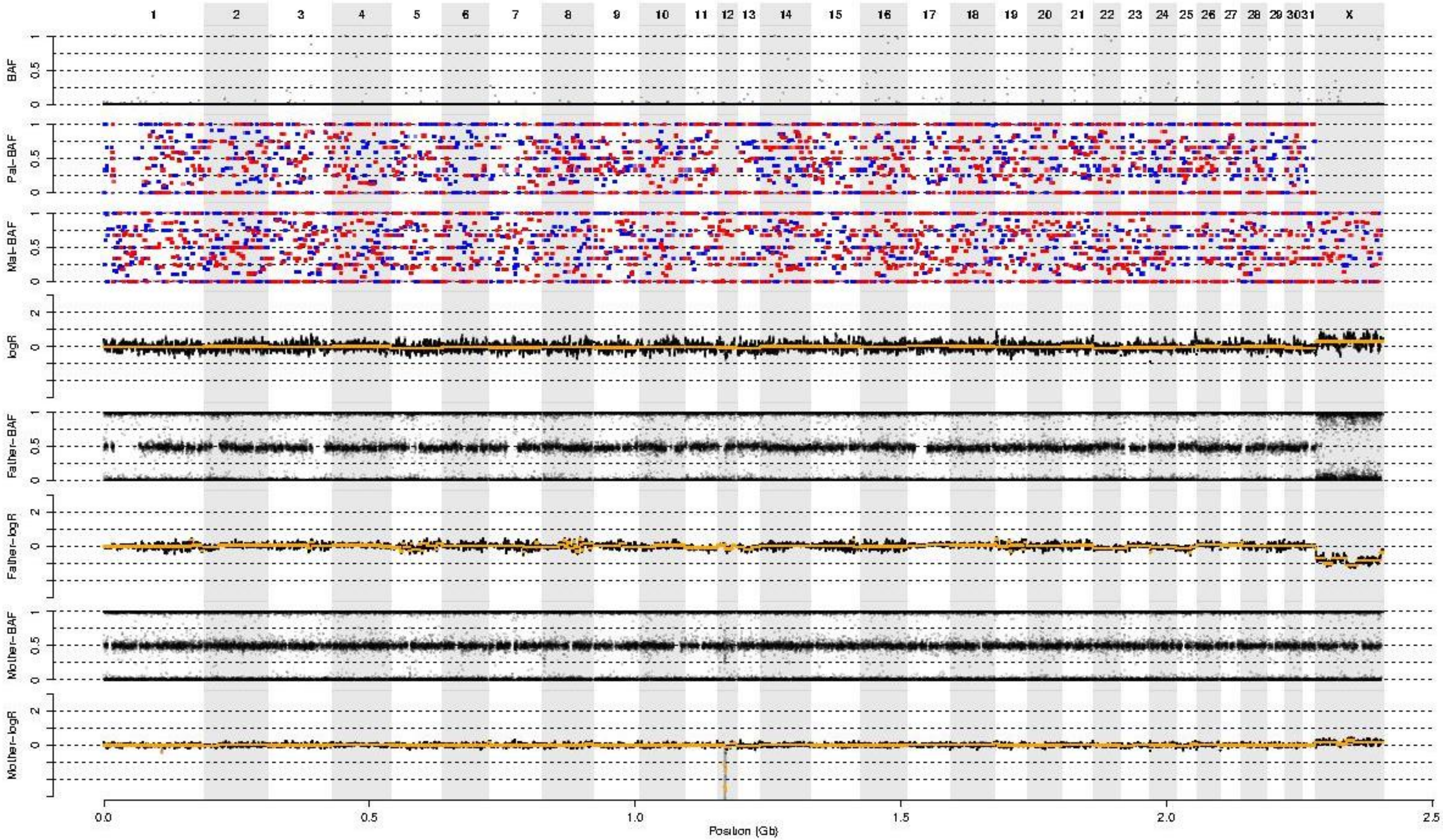

Mare01_ Embryo02_Cell7

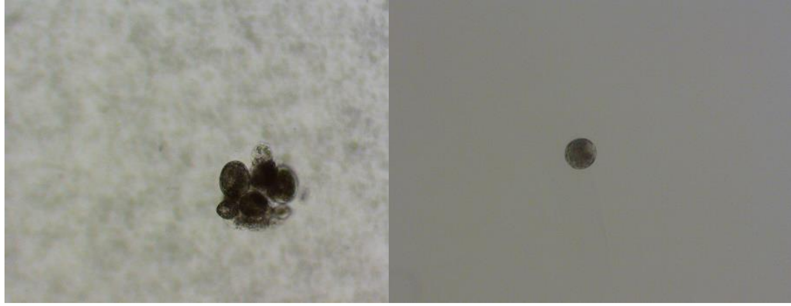

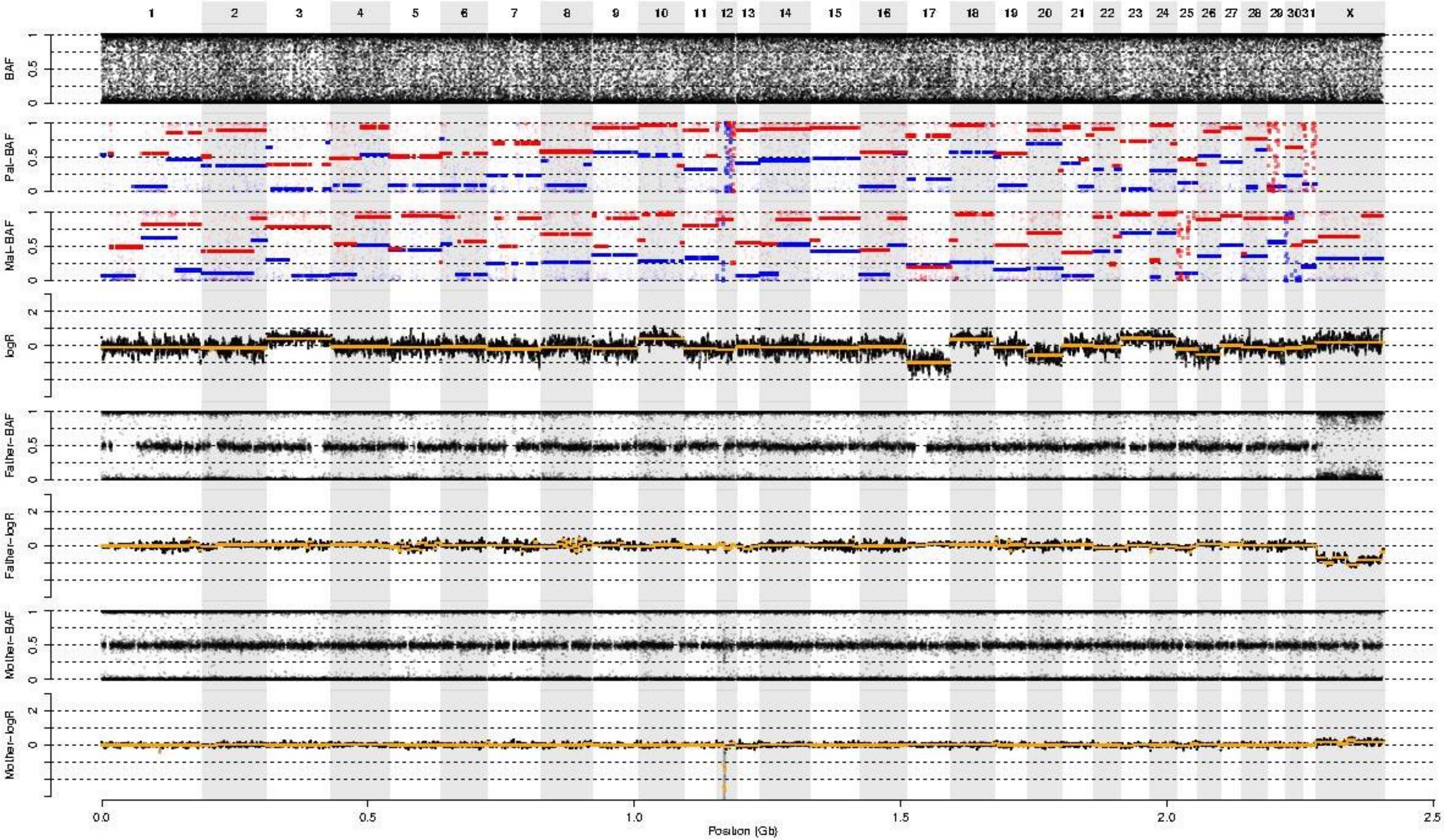

Mare01_ Embryo02_Cell8

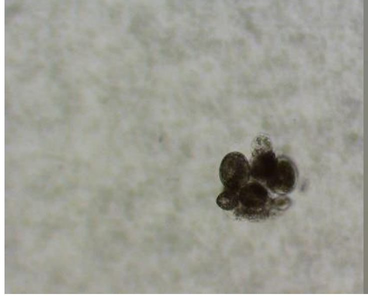

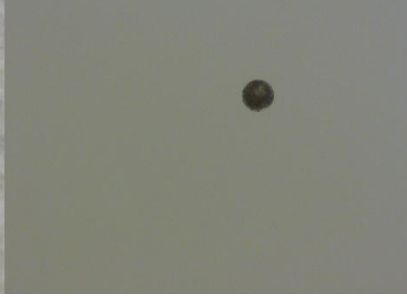

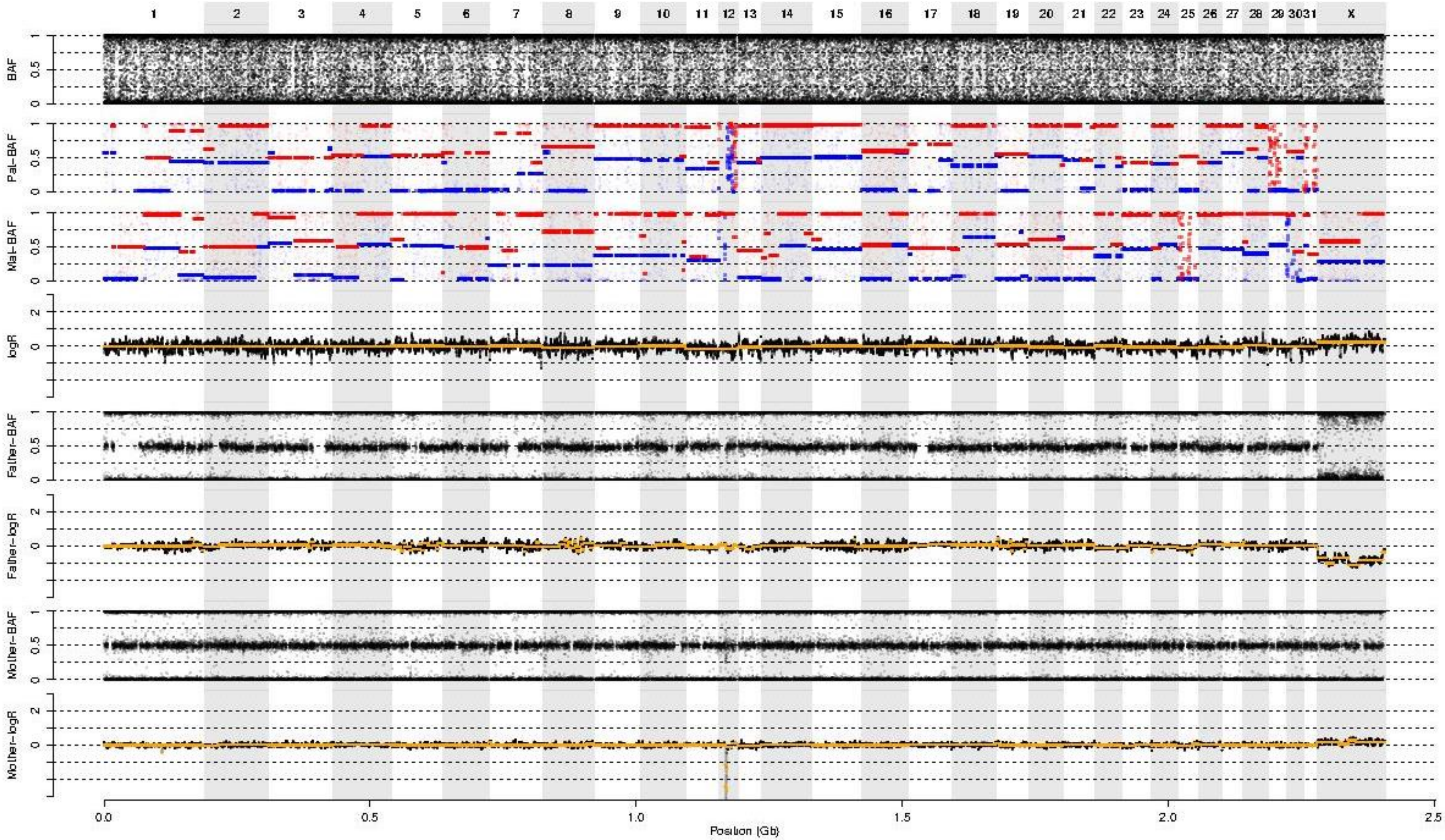

Mare02_ Embryo01_Cell1

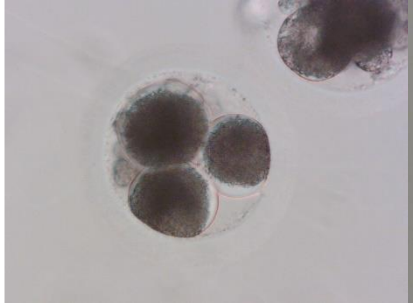

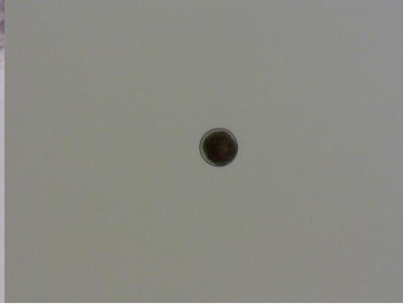

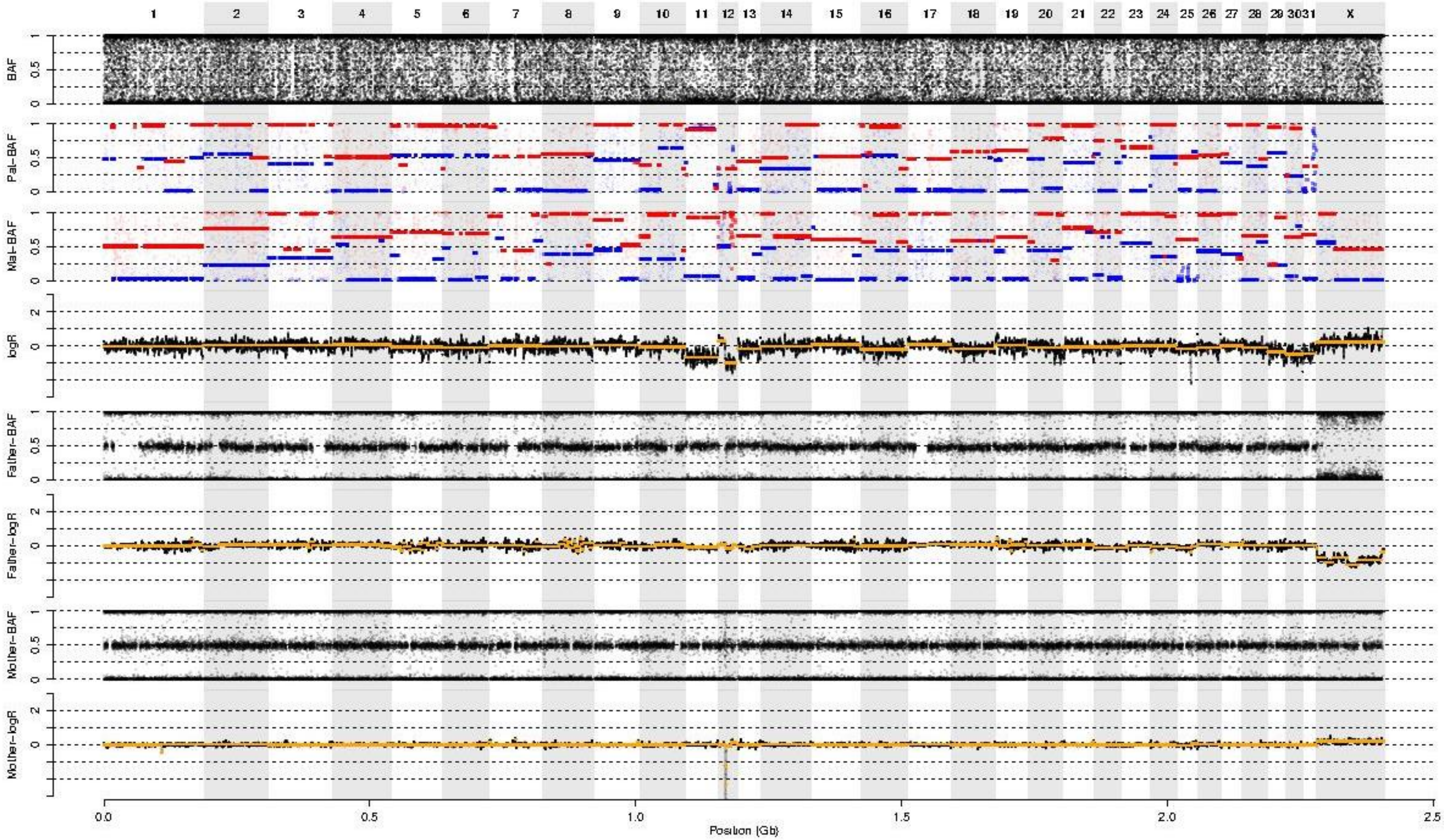

Mare02_ Embryo01_Cell2

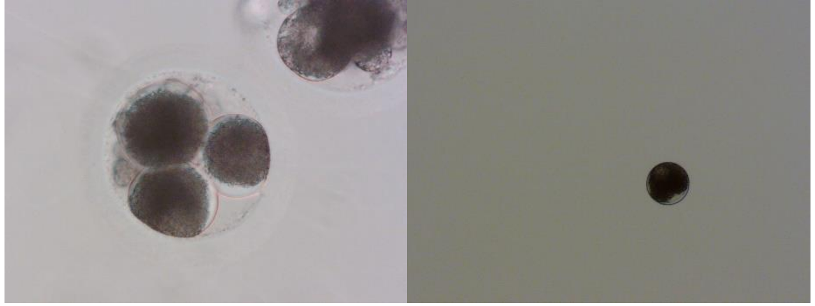

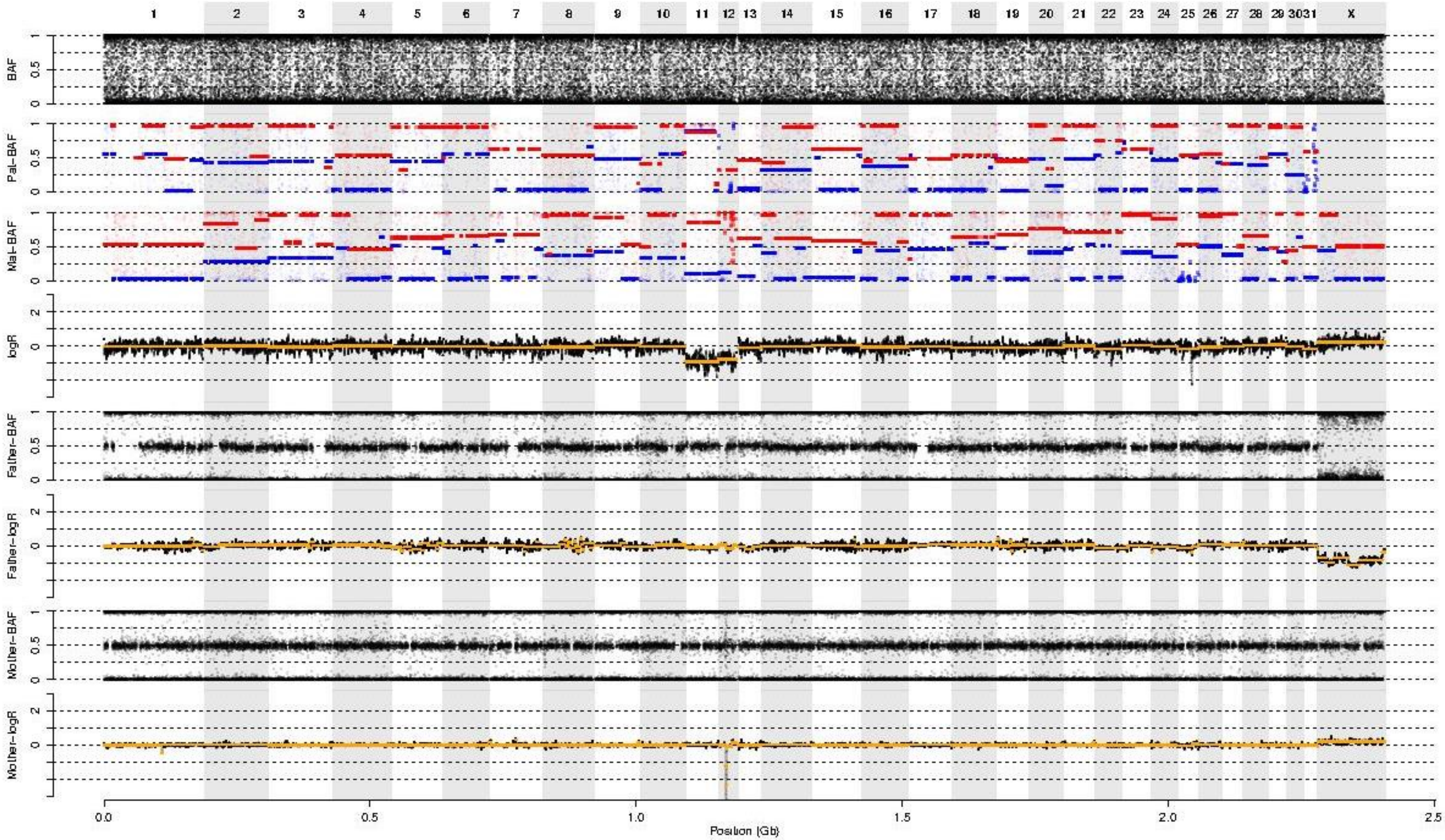

Mare02_ Embryo01_Cell3

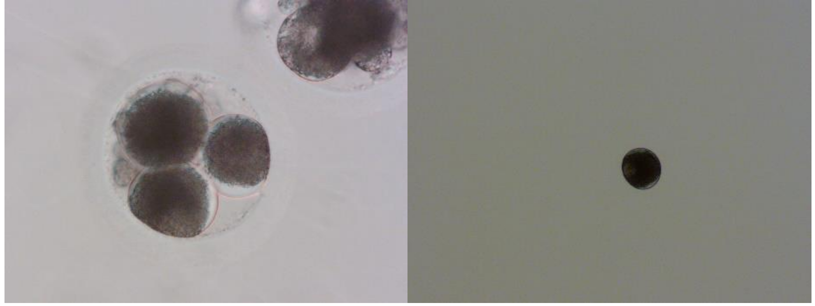

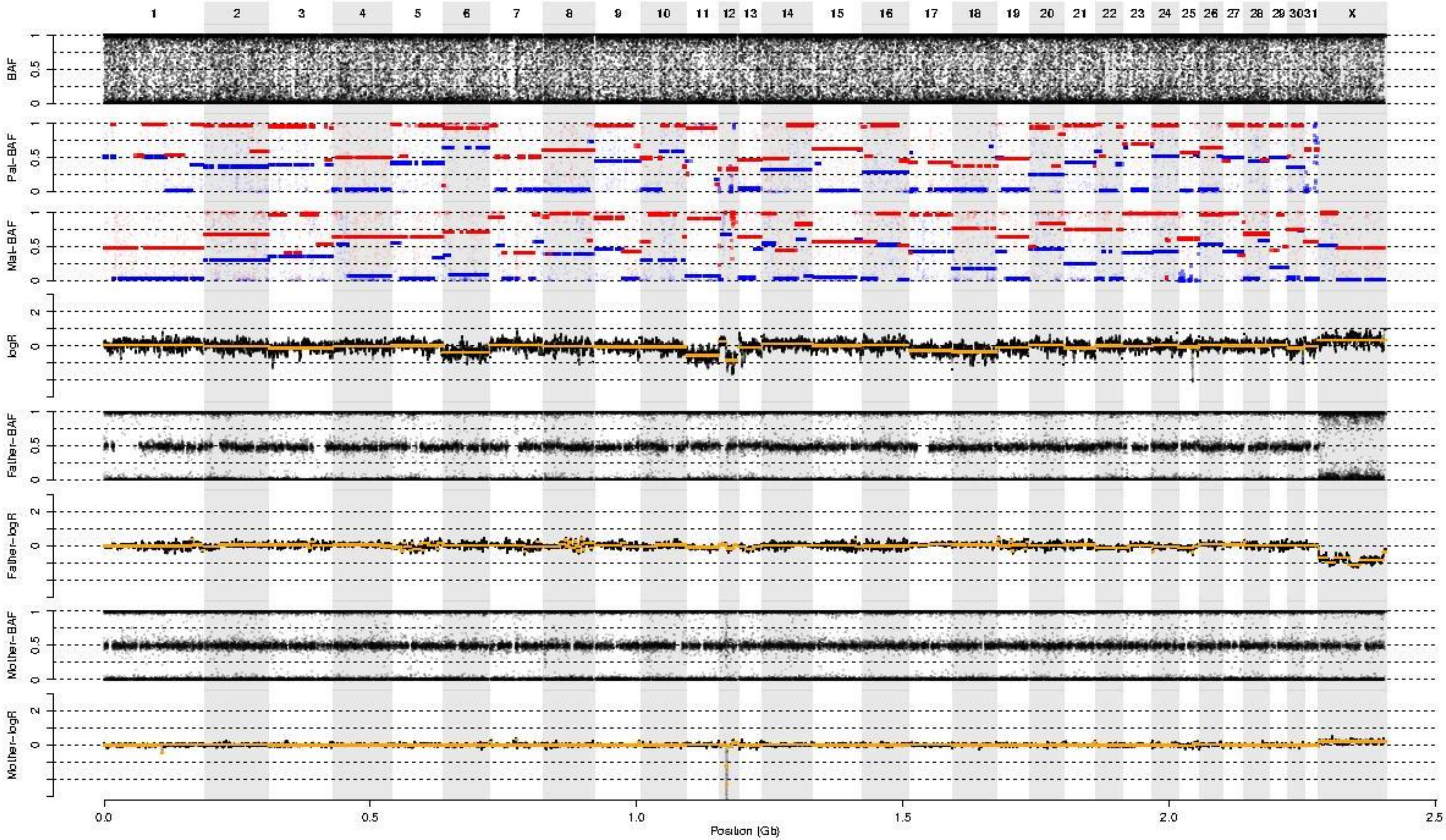

Mare02_ Embryo02_Cell1

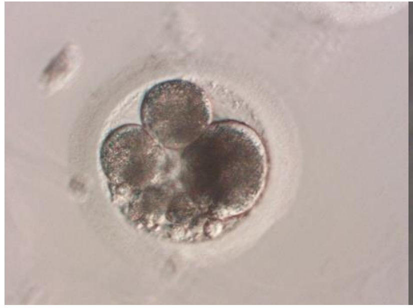

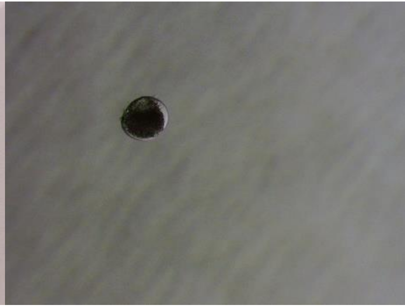

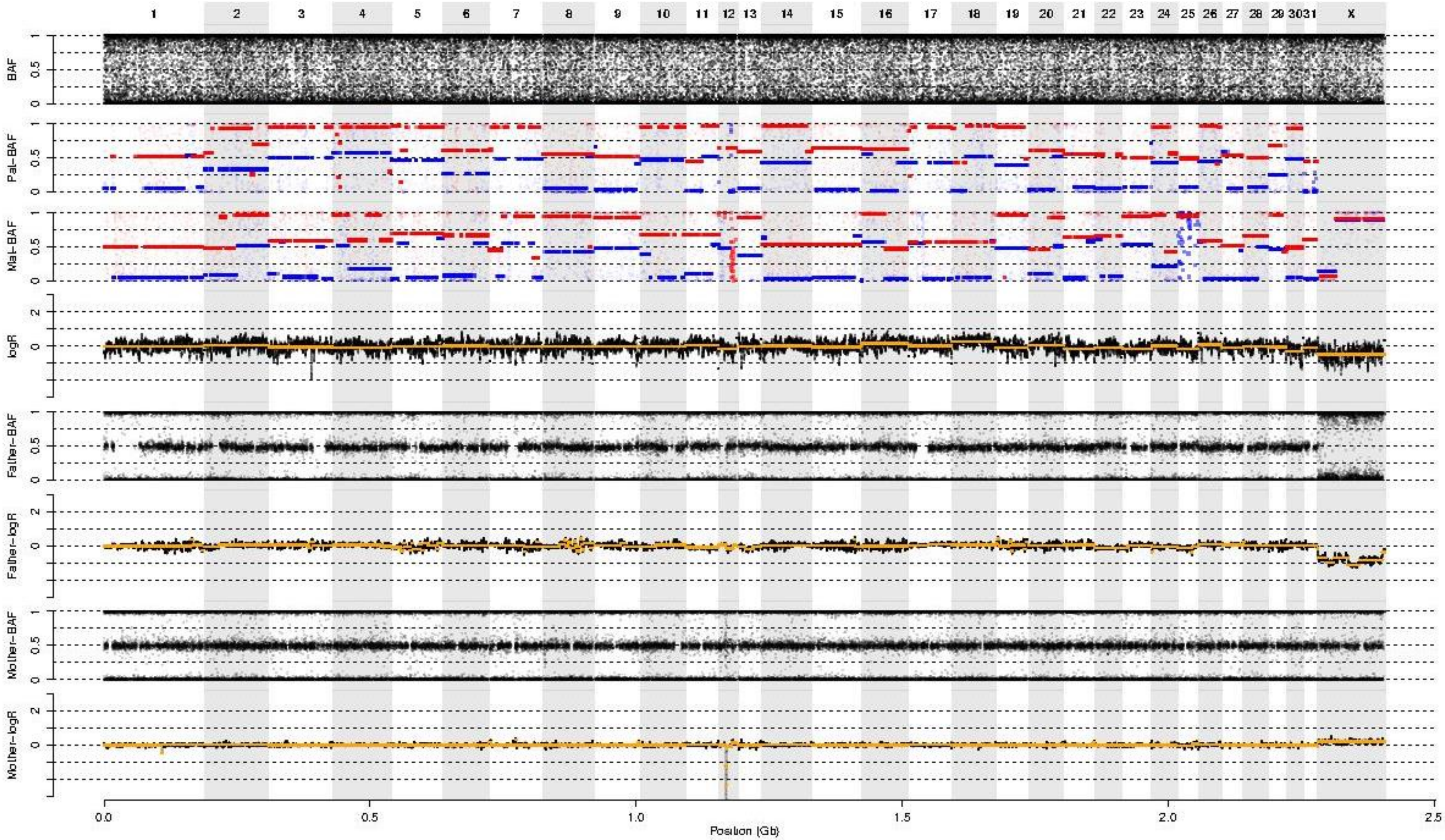

Mare02_ Embryo02_Cell2

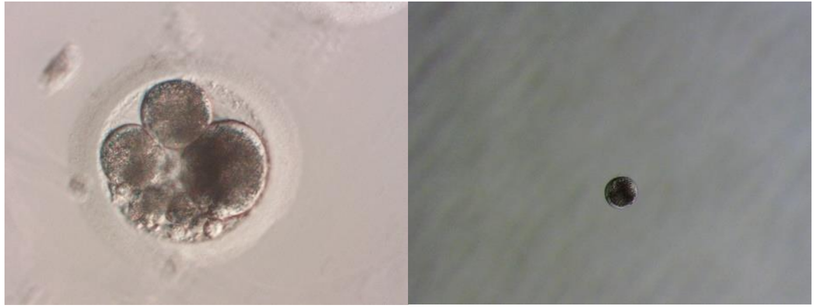

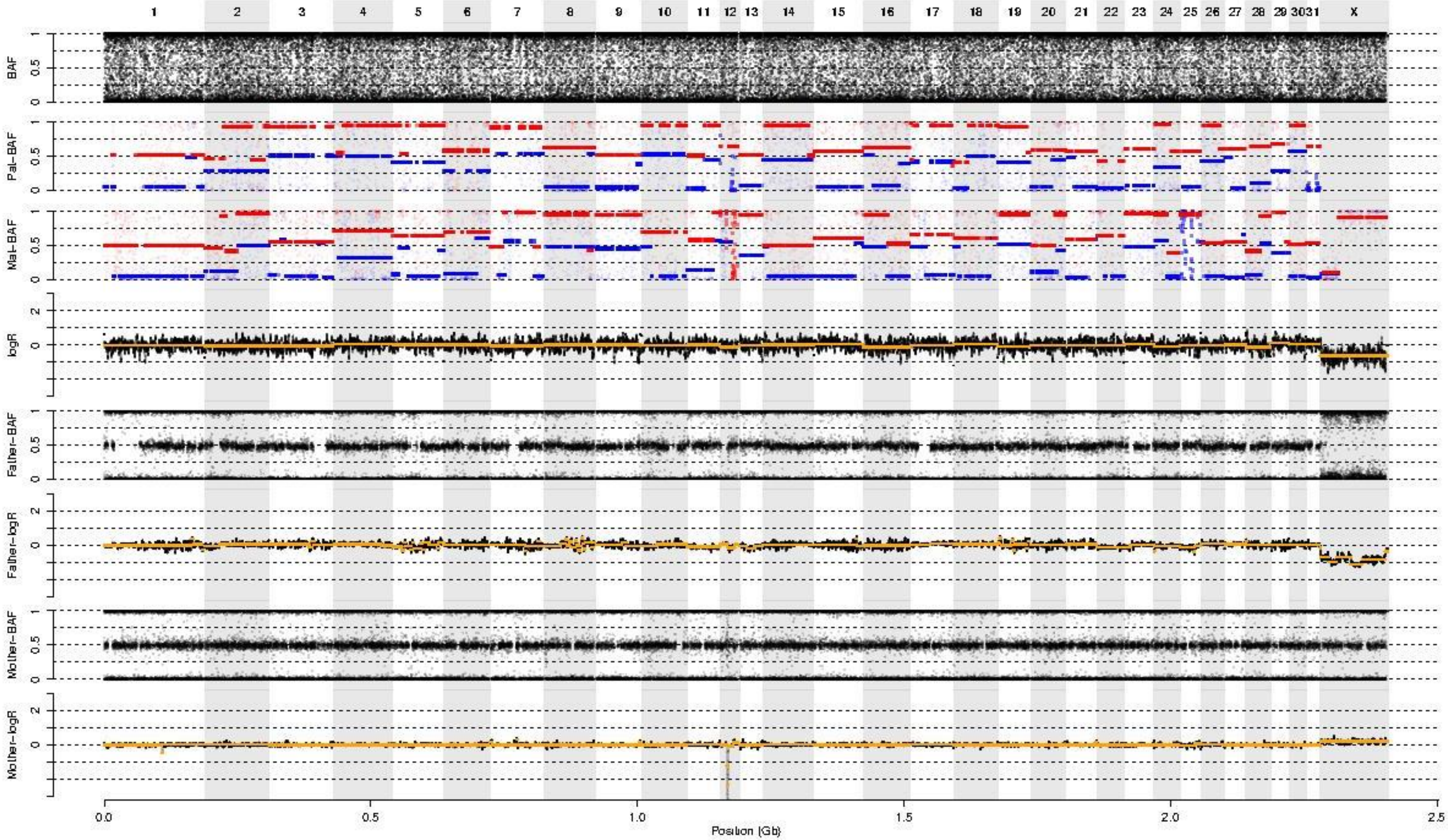

Mare02_ Embryo02_Cell3

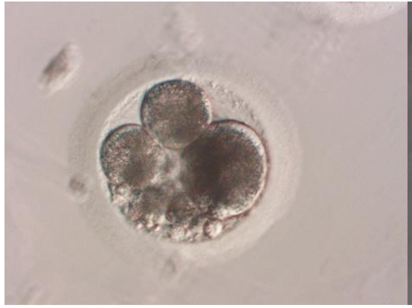

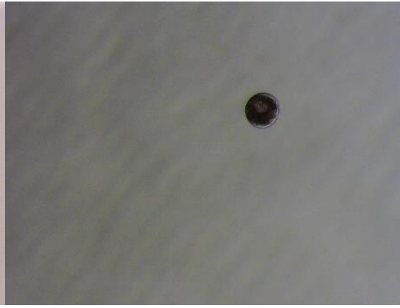

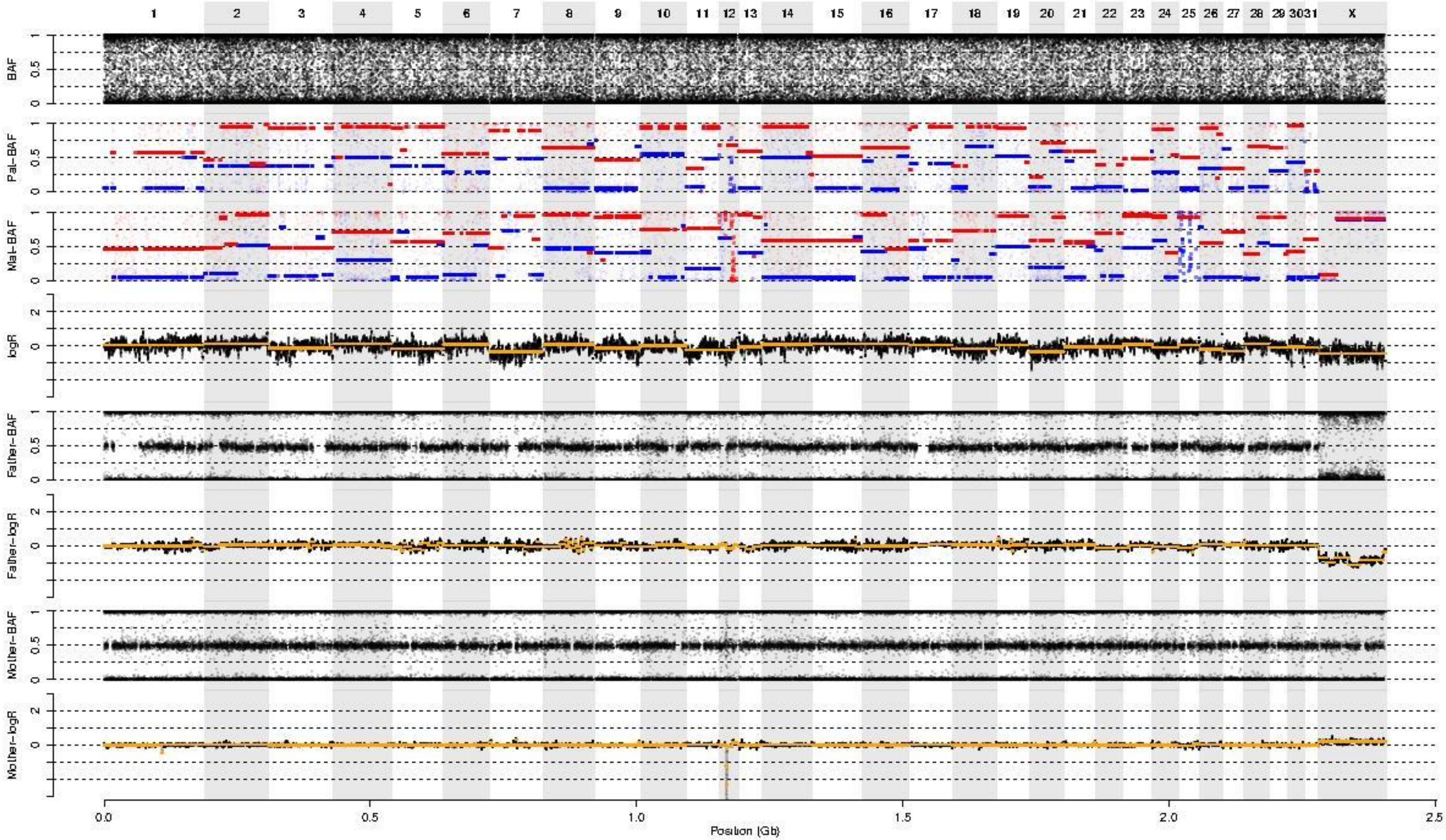

Mare02_ Embryo02_Cell4

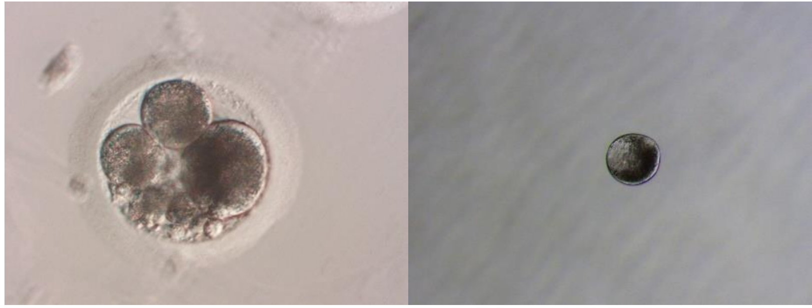

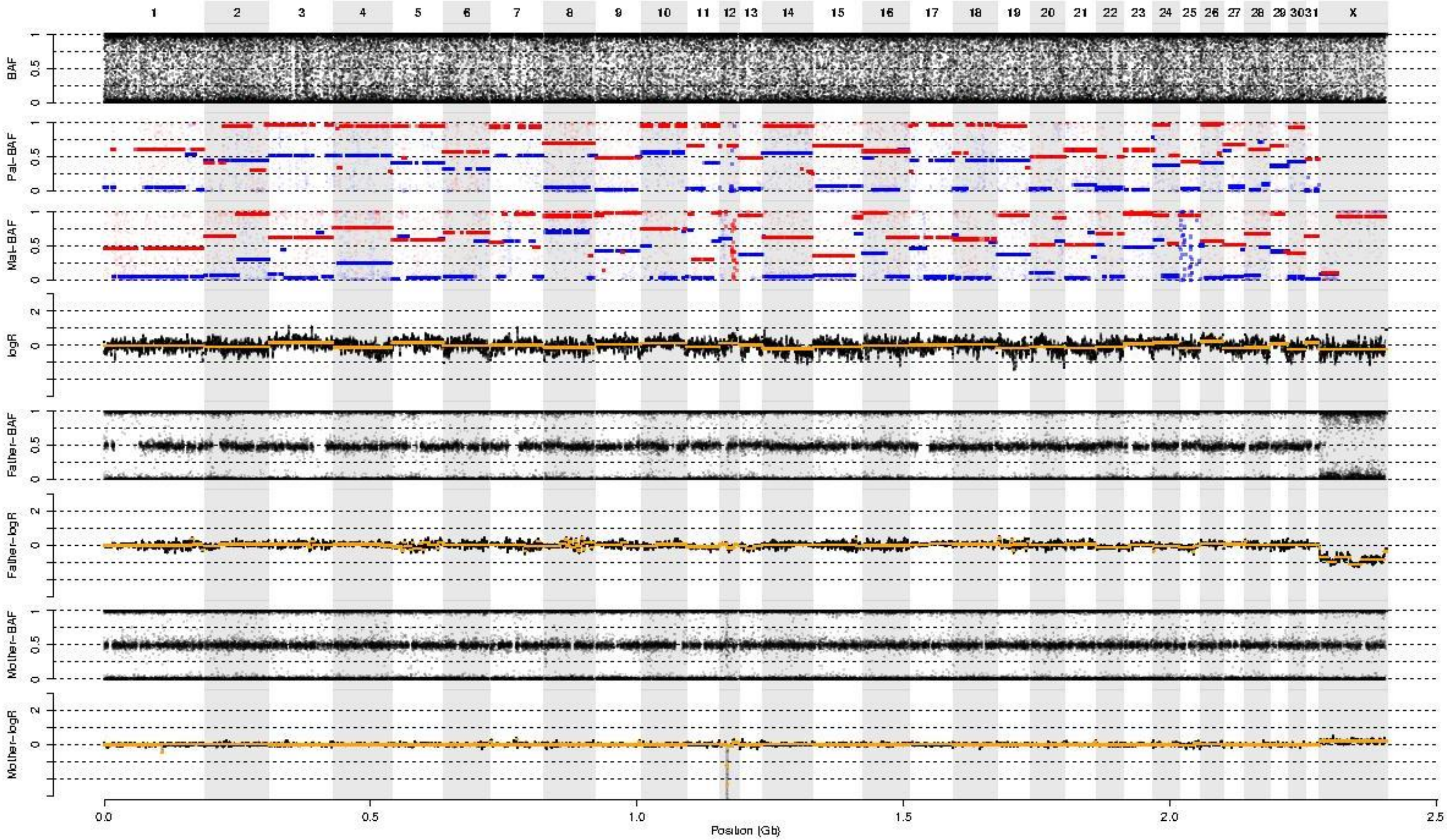

Mare02_ Embryo03_Cell1

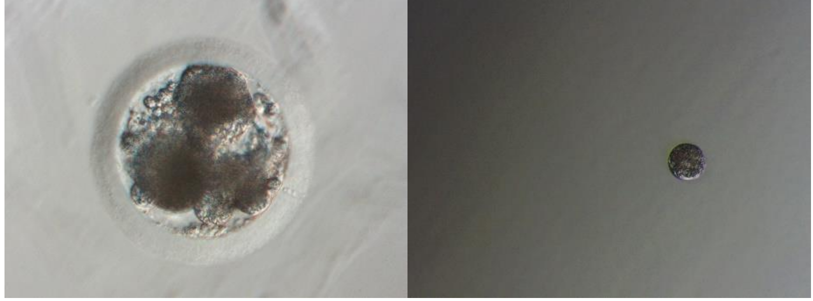

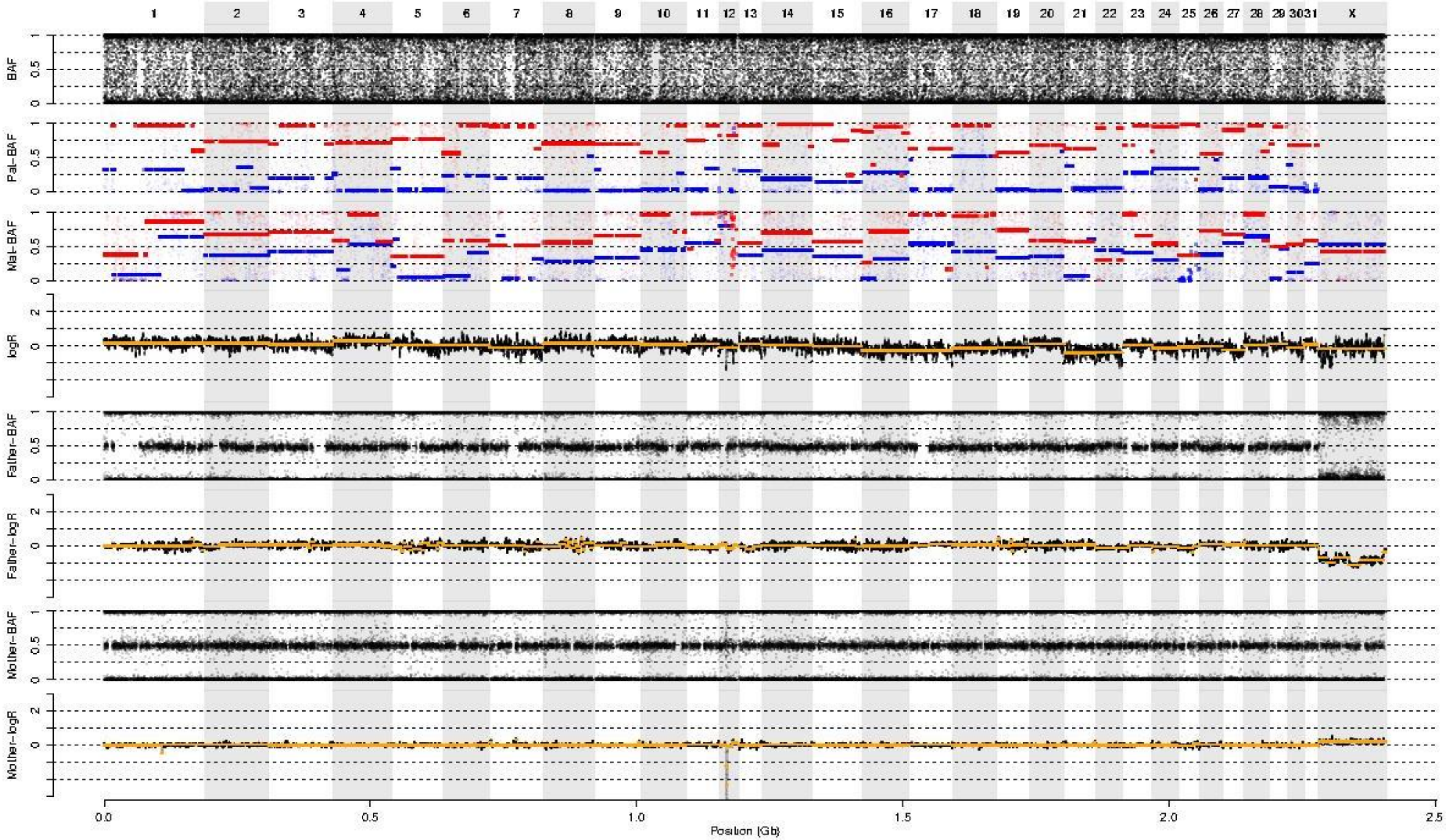

Mare02_ Embryo03_Cell2

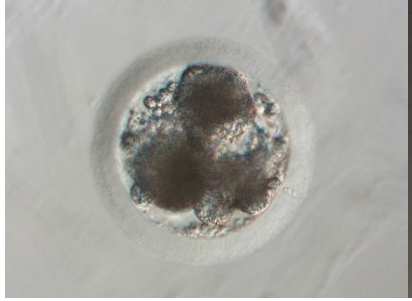

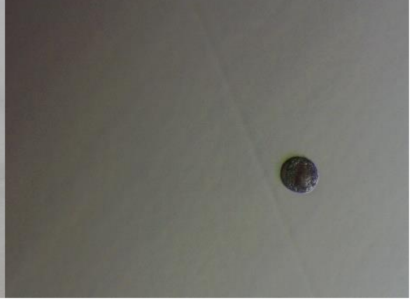

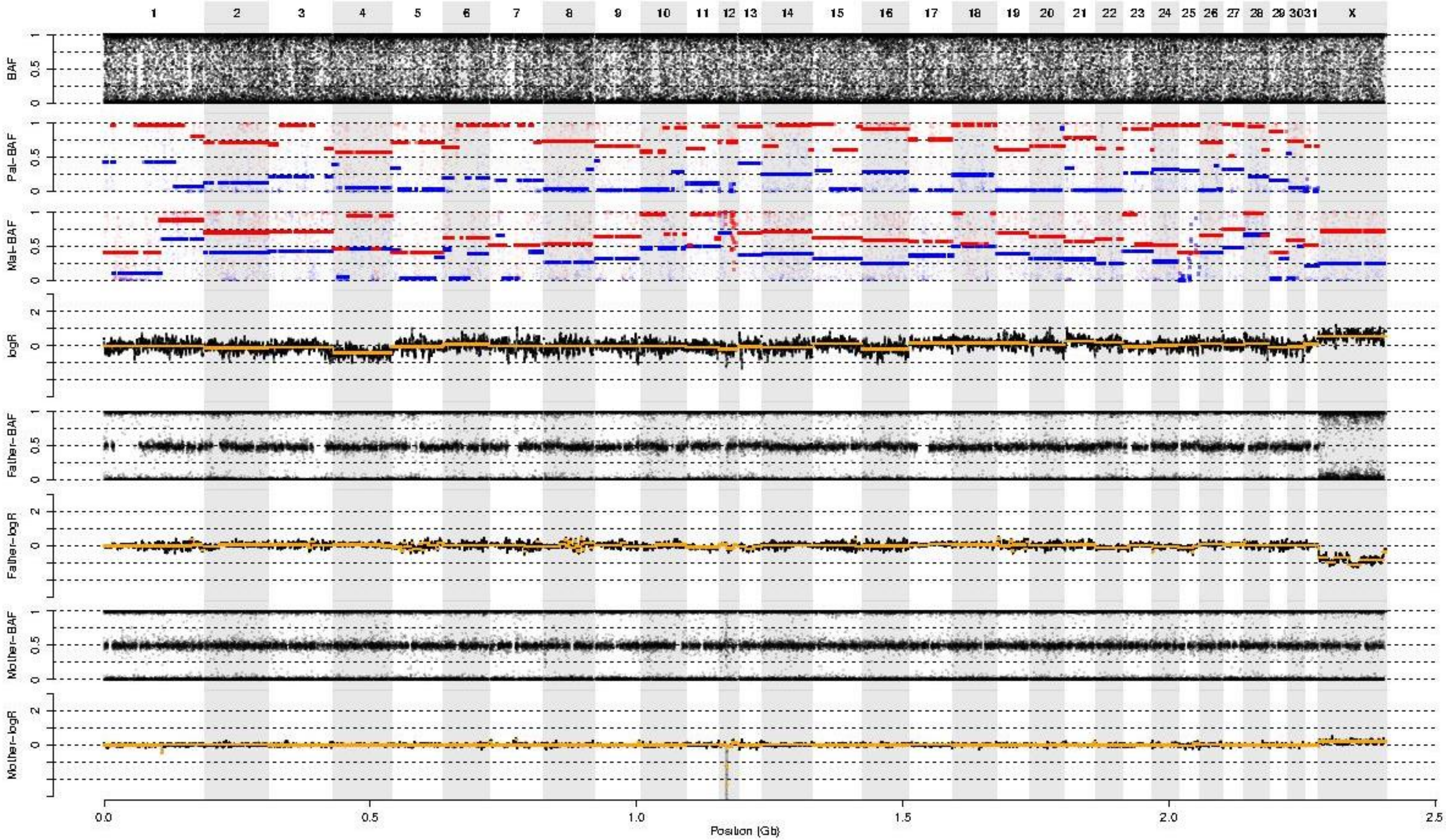

Mare02_ Embryo03_Cell3

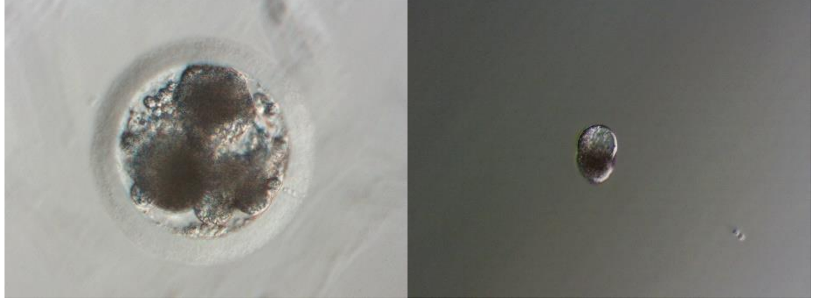

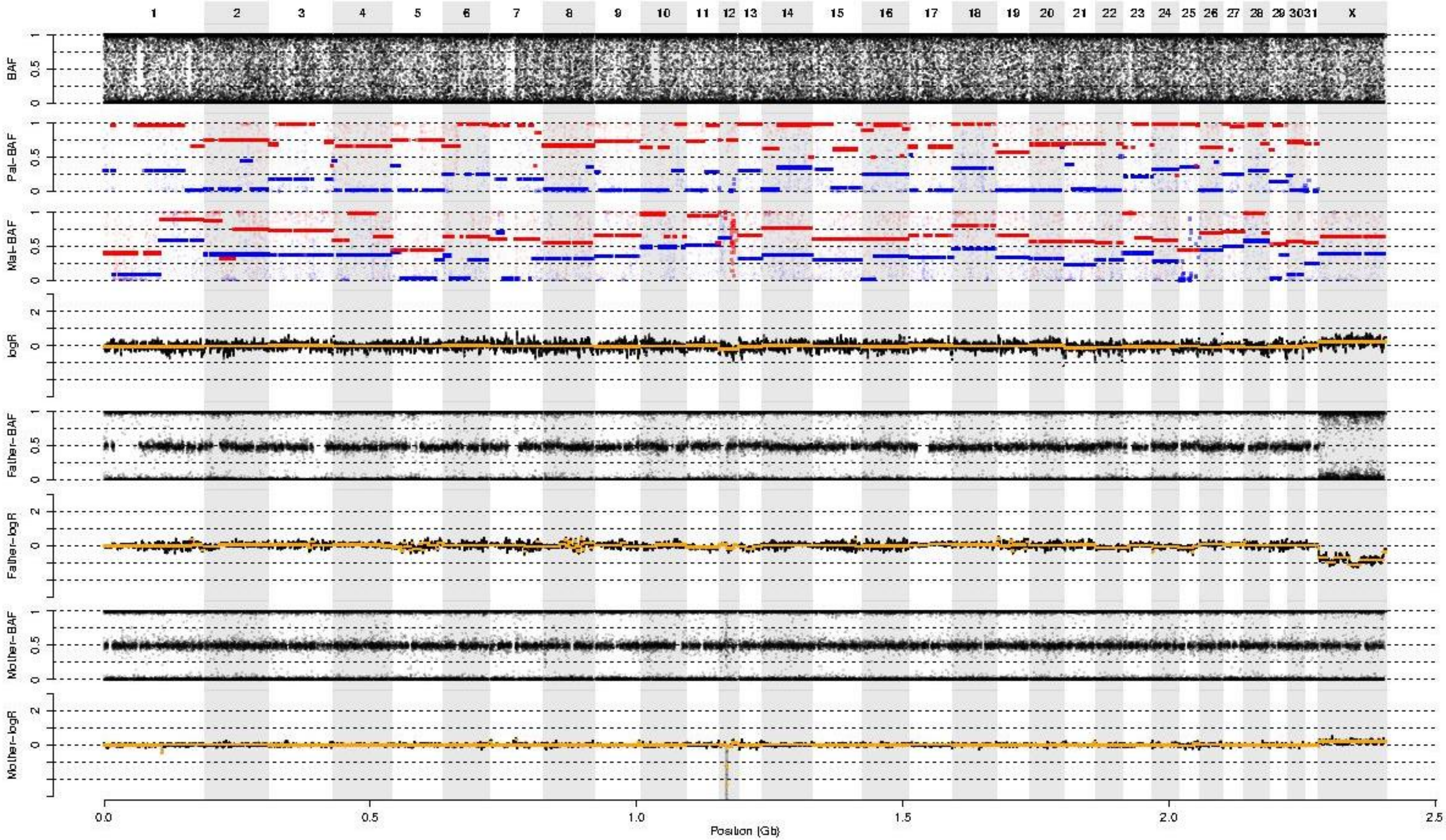

Mare02_ Embryo03_Cell4

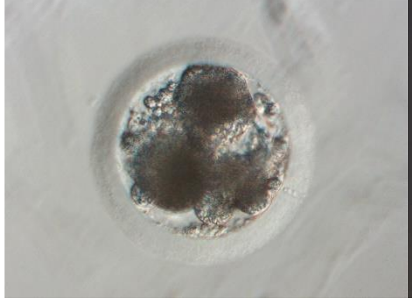

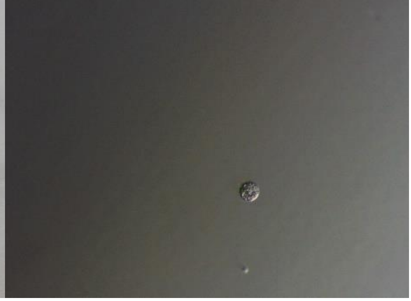

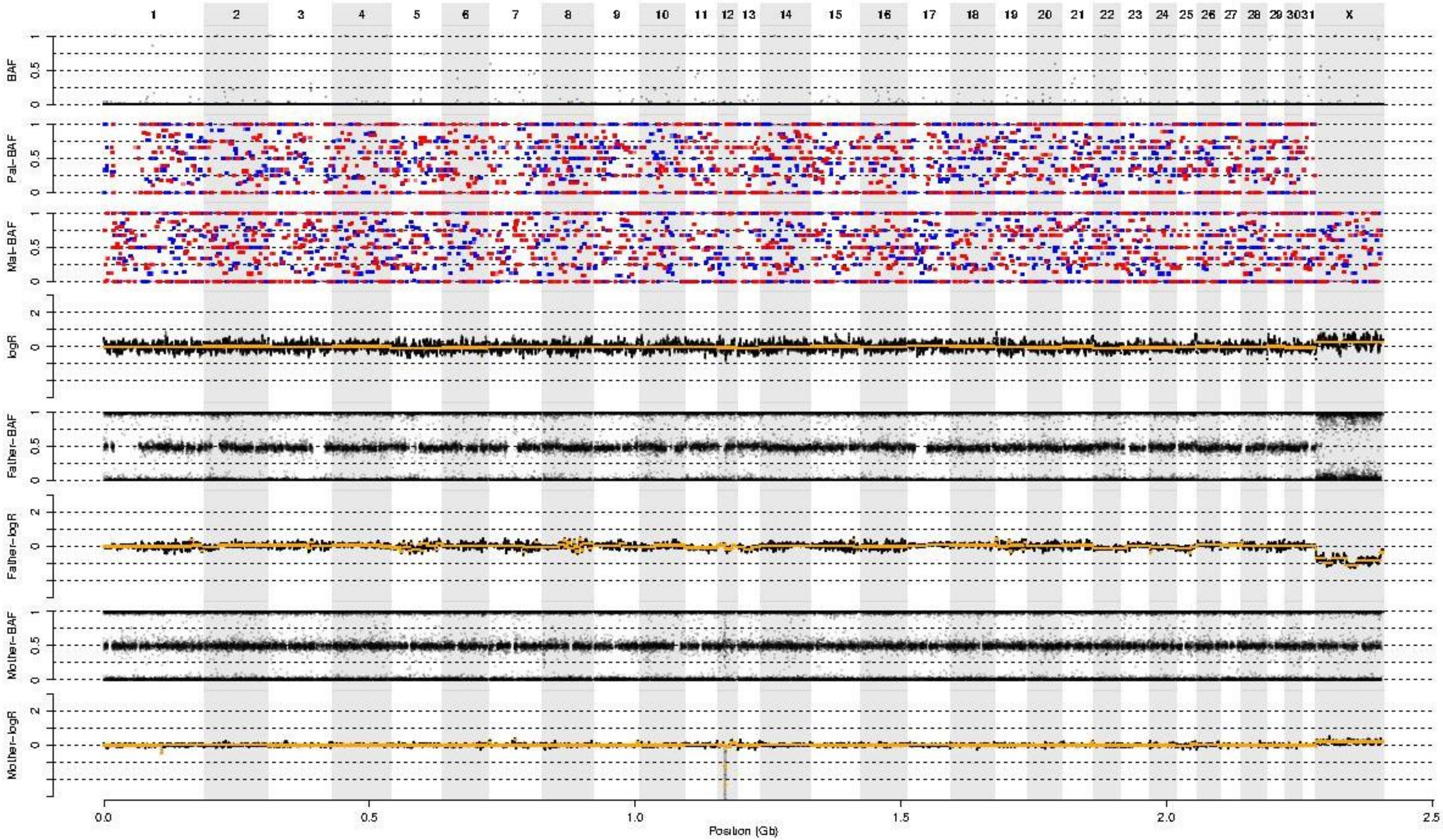

Mare02_ Embryo03_Cell5

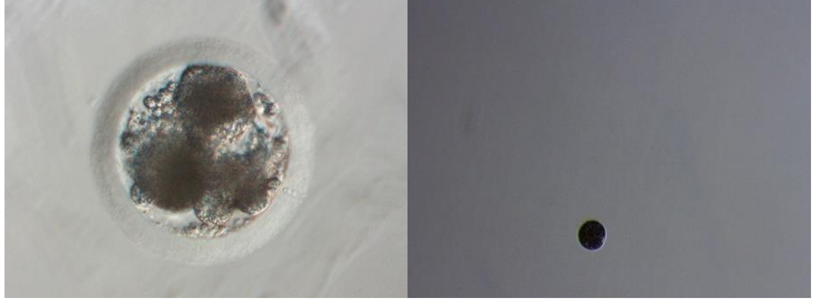

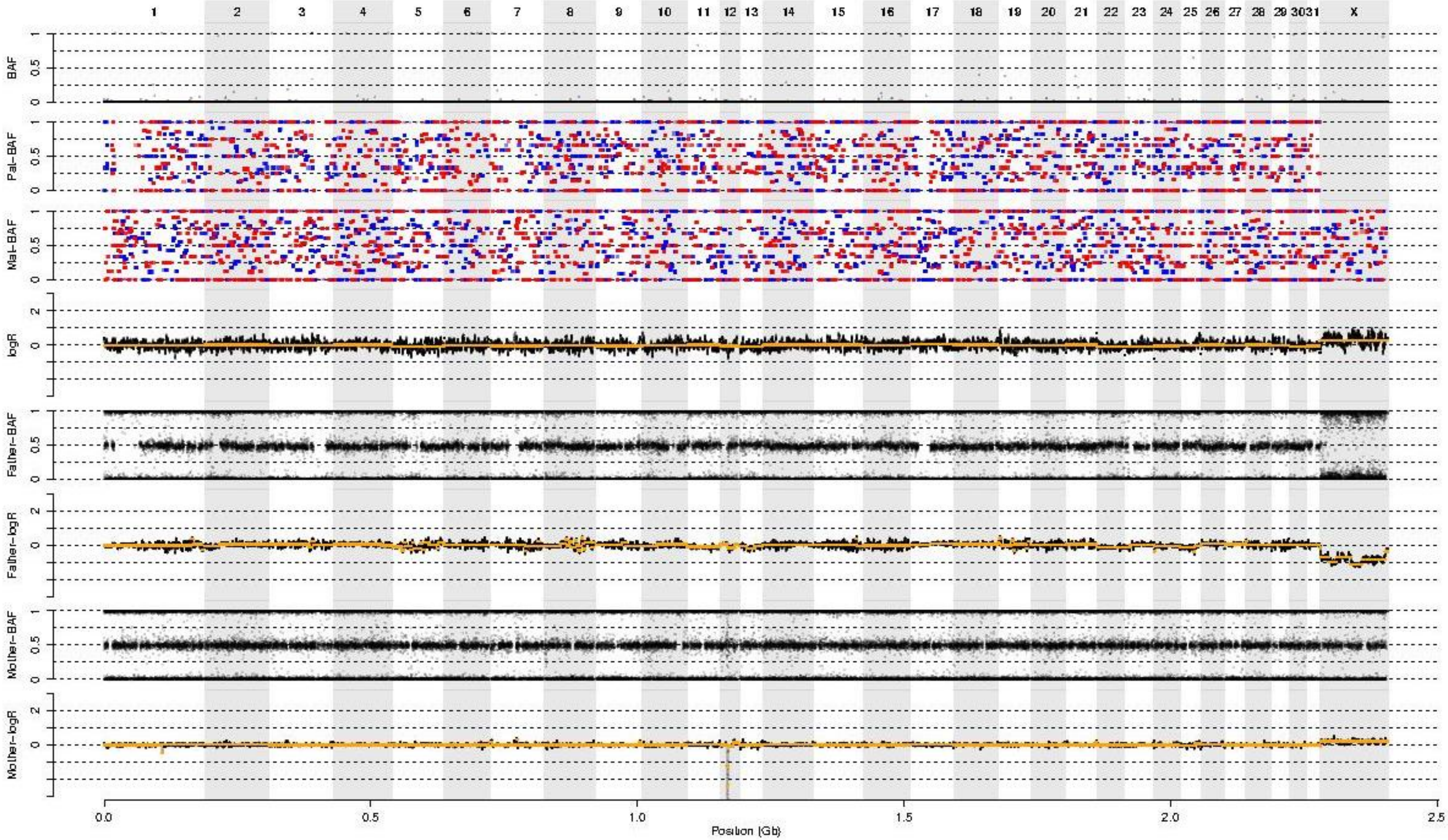

Mare02_ Embryo03_Cell6

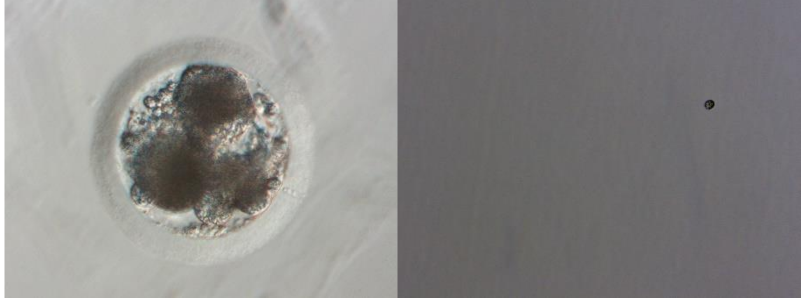

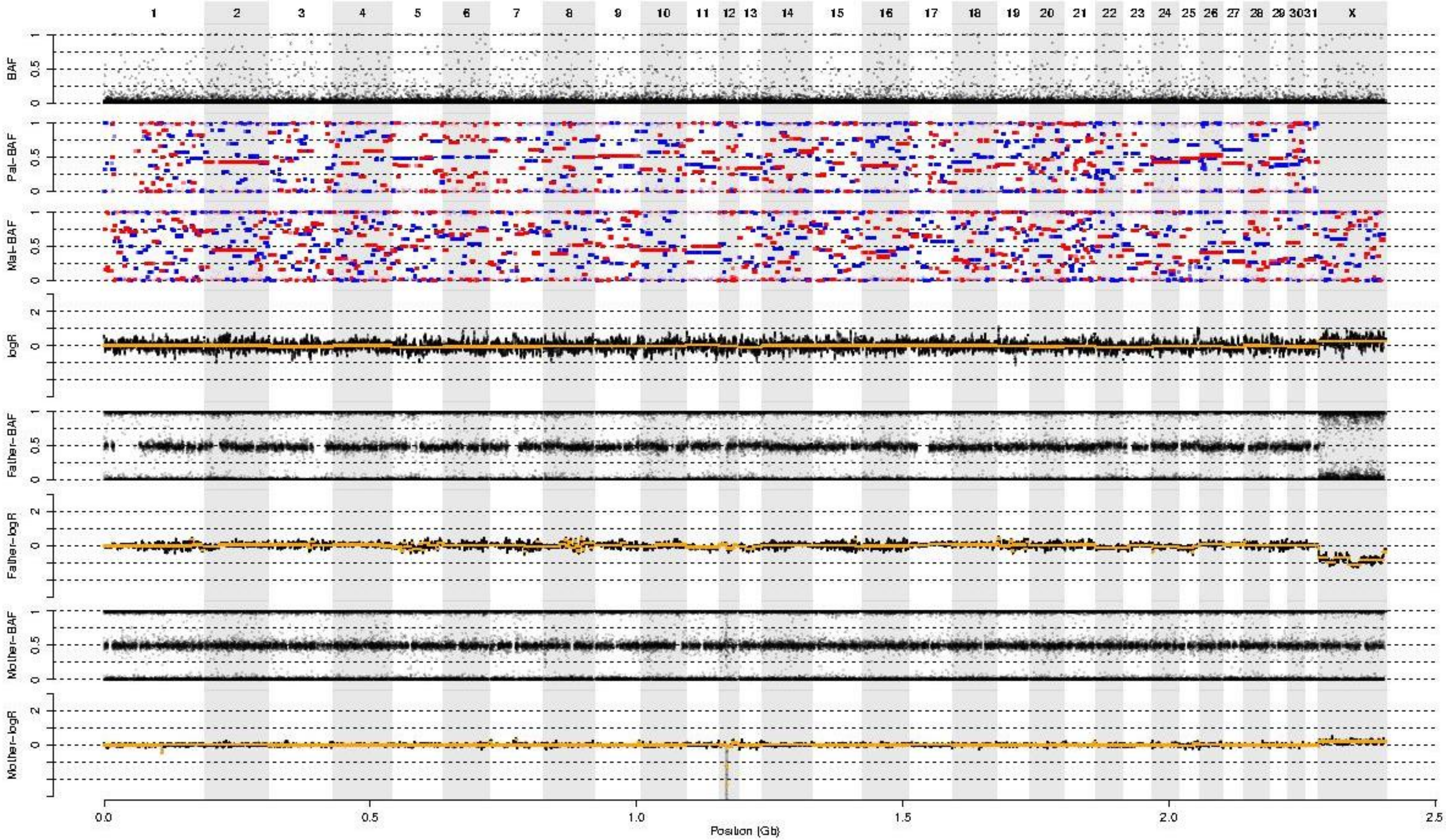

Mare04_ Embryo01_Cell1

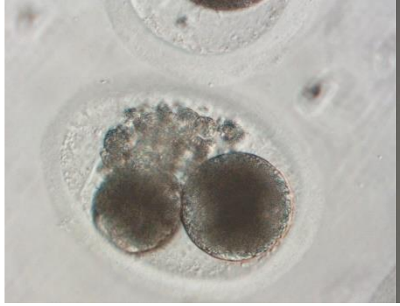

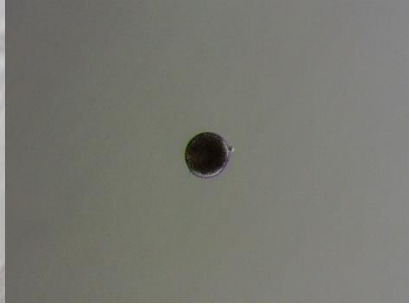

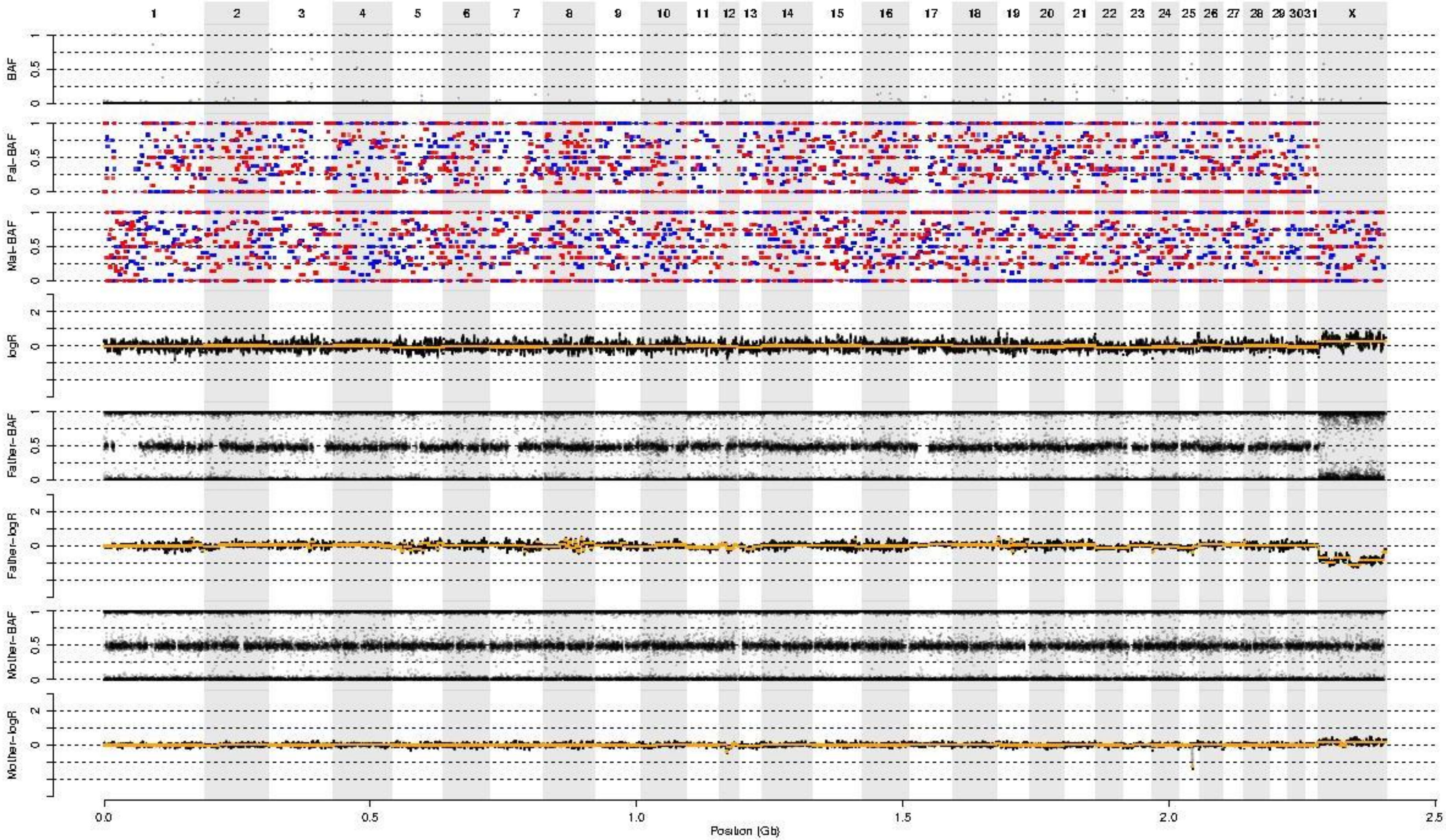

Mare04_ Embryo01_Cell2

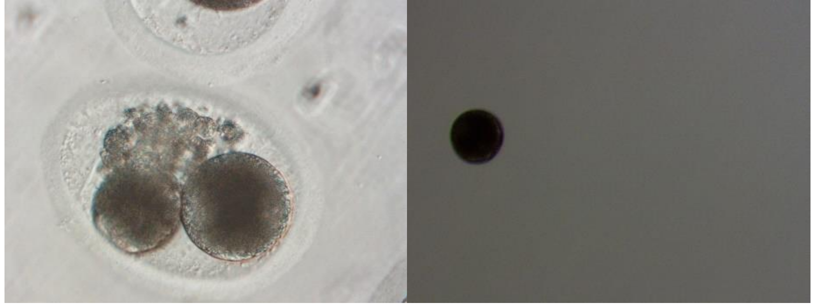

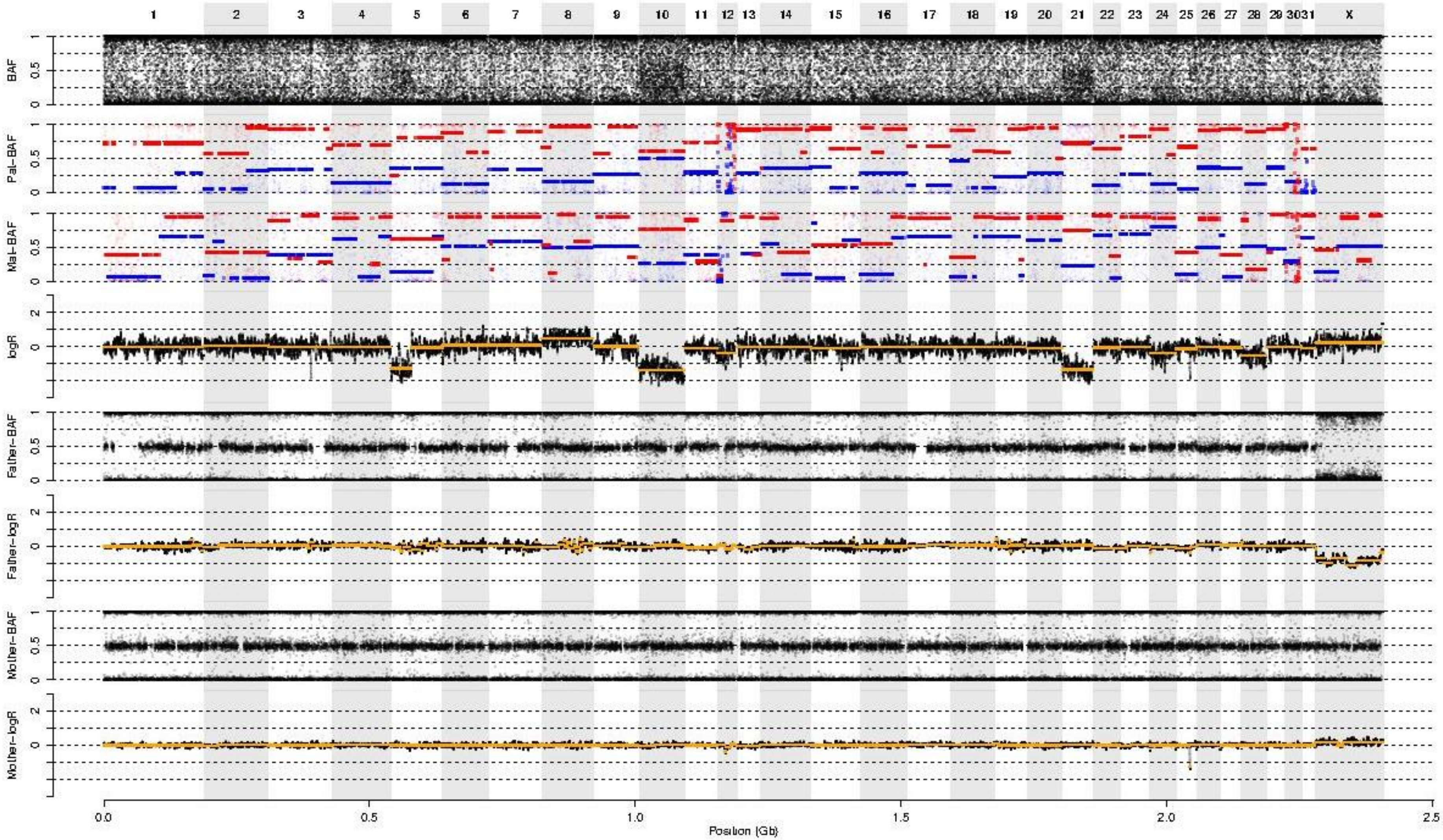

Supplement: Supplementary file 2 — Supplementary Information 2. [file 41598_2023_48103_MOESM2_ESM.pdf]
